# Radical Addition of Dihydroquinoxalin-2-ones to Trifluoromethyl Ketones under Visible-Light Photoredox Catalysis

**DOI:** 10.1021/acs.joc.2c01139

**Published:** 2022-07-05

**Authors:** Jaume Rostoll-Berenguer, María Martín-López, Gonzalo Blay, José R. Pedro, Carlos Vila

**Affiliations:** Departament de Química Orgànica, Facultat de Química, Universitat de València, Dr. Moliner 50, 46100 Burjassot, València, Spain

## Abstract

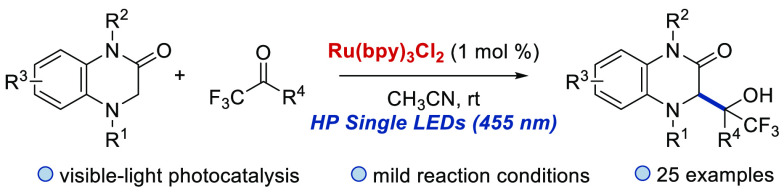

A visible-light photocatalytic radical addition reaction of dihydroquinoxalin-2-ones to trifluoromethyl ketones has been established using Ru(bpy)_3_Cl_2_ as photocatalyst, acetonitrile as solvent, and HP Single Blue LED as the source of light. The reaction provides a straightforward approach to the synthesis of dihydroquinoxalin-2-ones bearing a trifluoromethyl-substituted tertiary alcohol moiety in moderate to good yields under mild conditions.

## Introduction

**Scheme 1:**
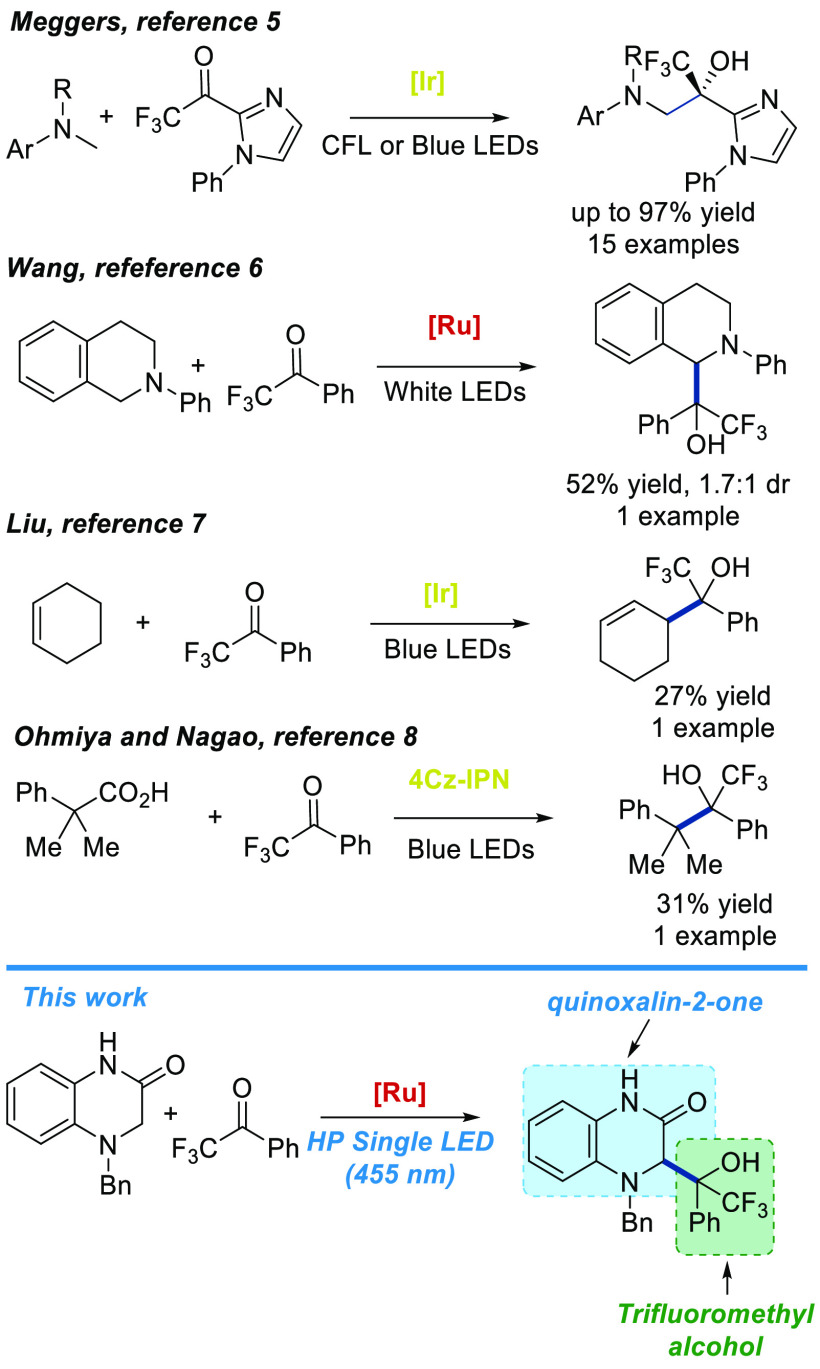
Examples of Photocatalytic Radical Reactions Using Trifluoromethyl Ketones

The synthesis of fluorinated molecules is a fundamental task for synthetic organic chemistry, due to the presence of fluorine atoms in a vast number of pharmaceuticals, agrochemicals, and materials.^1^ In this context, the trifluoromethyl group (CF_3_) has received a significant amount of attention and is often used in medicinal chemistry to replace the methyl group to prevent its metabolic oxidation, to adjust the steric and electronic properties or to increase the lipophilicity of biological active compounds.^2^ Therefore, the organic synthesis of building blocks bearing a trifluoromethyl moiety is very attractive. One of the most efficient and direct ways to incorporate a trifluoromethyl group into organic molecules is the use of trifluoromethyl ketones as reagents.^3^ So, a wide range of synthetic methodologies have been described using nucleophilic addition reactions with trifluoromethyl ketones as electrophiles. However, the radical–radical coupling or radical addition reactions using trifluoromethyl ketones are less studied, and relatively few examples are known. The radical–radical coupling and radical addition reactions are powerful C–C bond formation processes that have been recently established using visible-light photocatalysis, and several synthesis of secondary and tertiary alcohols have been reported.^4^ In this context, very few examples have been described using radical reactions for the synthesis of trifluoromethyl carbinols (Scheme 1). Meggers, in 2016, described an elegant photocatalytic enantio- and diastereoselective synthesis of 1,2-amino alcohols from tertiary amines and trifluoromethyl ketones using a chiral iridium photocatalyst. These authors described 15 examples with good yields with excellent stereoselectivity.^5^ In 2018, Wang and co-workers presented a coupling reaction of tertiary *N*-arylamines and aldehydes, ketones, and imines using visible-light photocatalysis, showing one example with trifluoroacetophenone.^6^ Later in 2019, Liu and co-workers reported one example (27% yield) of a radical–radical coupling of trifluoroacetophenone and cyclohexene using *fac*-Ir(ppy)_3_ as photocatalyst.^7^ Finally in 2021, Ohmiya and Nagao described one example of the photocatalytic synthesis of a tertiary trifluoromethyl alcohol from the reaction of 2-phenylisobutyric acid and trifluoroacetophenone.^8^ Herein, we present the reaction of trifluoromethyl ketones^9^ and dihydroquinoxalin-2-ones using visible-light photoredox catalysis leading to the synthesis of trifluoromethyl alcohols bearing a dihydroquinoxalin-2-one moiety. Dihydroquinoxalin-2-ones are privileged nitrogen heterocycles that are present in a broad assortment of biologically active compounds such as antiviral, antibiotic, anticancer, or anti-inflammatory drugs.^10^ Consequently, the functionalization of this class of nitrogen heterocycles is significant for medicinal and pharmaceutical chemistry. Many methodologies have been established, with the visible-light photocatalytic functionalization being one of the most straightforward and sustainable approaches.^11^ Continuing with our interest in the photocatalytic functionalization of dihydroquinoxalin-2-ones,^12^ we hypothesized that this class of heterocycles could be an appropriate precursor of α-amino radicals^13^ to perform the radical addition reaction to trifluoromethyl ketones under visible-light photocatalysis.

## Results and Discussion

We started our studies with the reaction of 4-benzyl-3,4-dihydroquinoxalin-2(1*H*)-one (**1a**) with 2,2,2-trifluoroacetophenone (**2a**) in the presence of different visible-light photocatalysts in acetonitrile as a solvent at room temperature and under HP (High Power) Single Blue LED irradiation (Table 1). 4-Benzyl-3,4-dihydroquinoxalin-2(1*H*)-one is a challenging molecule because of the possible formation of two α-amino radicals at the α-position to the amide or at the benzylic position. The initial raction using 1 mol % Ru(bpy)_3_Cl_2_ under irradiation of HP Single Blue LED (455 nm) afforded the corresponding trifluoromethyl alcohol **3aa** (diastereoisomers mixture) in 73% yield after 2.5 h reaction time (entry 1), although without diastereoselectivity. Other photocatalysts such Eosin Y or 4-CzIPN (2,4,5,6-tetrakis(9*H*-carbazol-9-yl) isophthalonitrile) were unsuccessful, and the formation of alcohol **3aa** was not observed. Unexpectedly, when Ru(bpy)_3_(PF_6_)_2_ was used as photocatalyst, product **3aa** was obtained with only 23% yield after 24 h (entry 4). A solvent screening (entries 5–7) with Ru(bpy)_3_Cl_2_ photocatalyst did not improve the results obtained with acetonitrile. Increasing the amount of trifluoroacetophenone was detrimental for the conversion to product **3aa** (entries 8–10), and it was isolated with lower yield (56–66%). As we described before for a photocatalytic Giese addition of **1a**,^12c^ in order to improve the conversion, we decided to use (PhO)_2_PO_2_H as a Brønsted acid additive. Unfortunately, the obtained yield for **3aa** was lower (26%). We could perform the reaction at the 0.2 mmol scale obtaining the same yield (entry 12). Finally, several control experiments were carried out showing that the model reaction did not occur without the presence of the Ru(bpy)_3_Cl_2_ photocatalyst (entry 13) or without visible-light irradiation (entry 14).

**Table 1:**
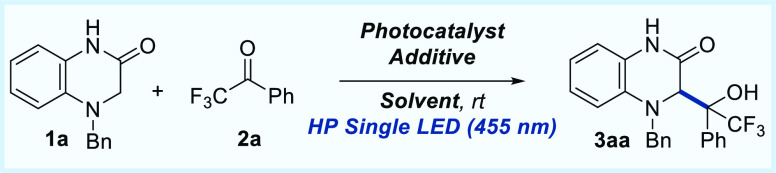
Optimization of the Reaction Conditions^a^

Entry	Photocatalyst	Solvent	Additive	*t* (h)	dr^b^	Yield (%)^c^
1	Ru(bpy)_3_Cl_2_·H_2_O (1%)	CH_3_CN	-	2.5	1:1	73
2	Eosin Y (5%)	CH_3_CN	-	24	-	-
3	4-CzIPN (2%)	CH_3_CN	-	24	-	-
4	Ru(bpy)_3_(PF_6_)_2_ (1%)	CH_3_CN	-	23	1:1	23
5	Ru(bpy)_3_Cl_2_·H_2_O (1%)	DMF	-	5	1:1	63
6	Ru(bpy)_3_Cl_2_·H_2_O (1%)	CH_2_Cl_2_	-	24	1:1	>5^b^
7	Ru(bpy)_3_Cl_2_·H_2_O (1%)	THF	-	24	1:1	>5^b^
8^d^	Ru(bpy)_3_Cl_2_·H_2_O (1%)	CH_3_CN	-	2.5	1:1	66
9^e^	Ru(bpy)_3_Cl_2_·H_2_O (1%)	CH_3_CN	-	2.5	1:1	56
10^f^	Ru(bpy)_3_Cl_2_·H_2_O (1%)	CH_3_CN	-	2.5	1:1	58
11	Ru(bpy)_3_Cl_2_·H_2_O (1%)	CH_3_CN	(PhO)_2_PO_2_H (10%)	24	1:1	26
12^g^	Ru(bpy)_3_Cl_2_·H_2_O (1%)	CH_3_CN	-	2.5	1:1	72
13	**-**	CH_3_CN	-	24	-	-
14^h^	Ru(bpy)_3_Cl_2_·H_2_O (1%)	CH_3_CN	-	24	-	-

a Reaction conditions: 0.13 mmol of **1a**, 0.1 mmol **2a**, *x* mol % of photocatalyst in 1 mL of solvent at rt under an Ar atmosphere and HP Blue LED (450 nm) irradiation.

b Determined by ^1^H NMR.

c Isolated yield of **3aa**.

d Reaction was performed with 0.1 mmol of **1a** and 0.3 mmol **2a**.

e Reaction was performed with 0.1 mmol of **1a** and 0.2 mmol **2a**.

f Reaction was performed with 0.1 mmol of **1a** and 0.13 mmol **2a**.

g Reaction performed with 0.26 mmol of **1a**, 0.2 mmol of **2a** in 2 mL of CH_3_CN at rt under HP Blue LED (455 nm) irradiation.

h Reaction performed under darkness.

**Scheme 2:**
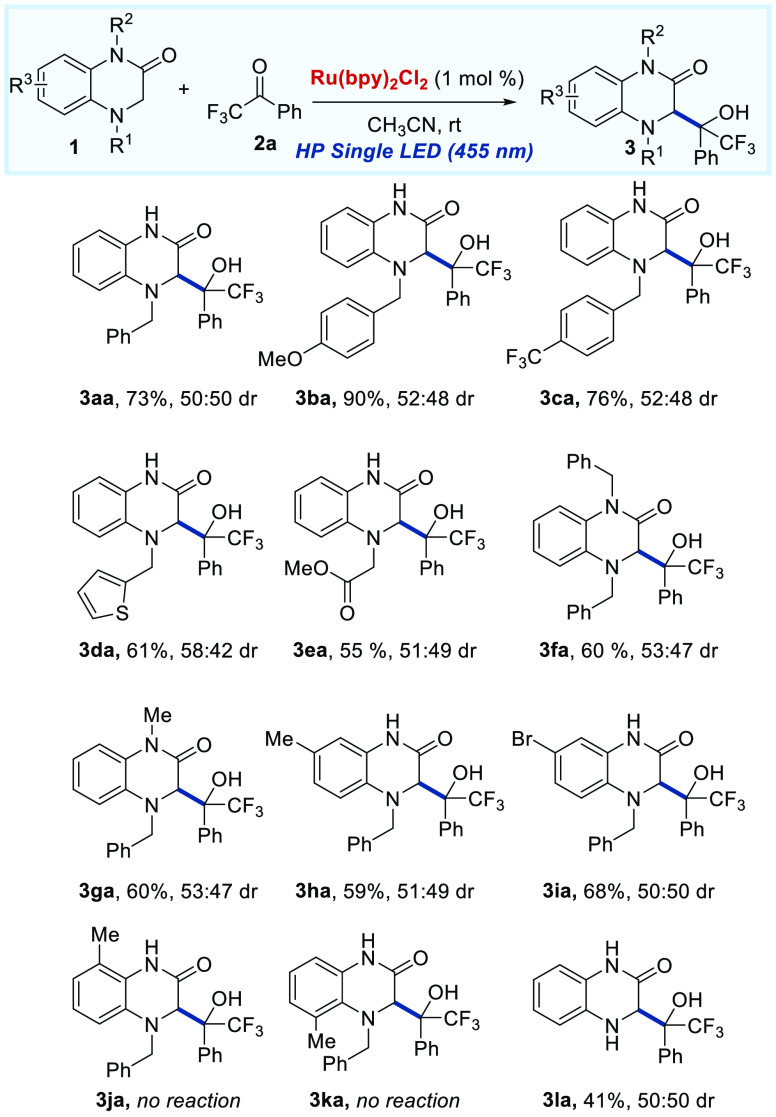
Scope of the Radical Addition Reaction Regarding the Dihydroquinoxalin-2-one Derivatives **1** Reaction conditions: **1** (0.26 mmol), **2a** (0.2 mmol), and Ru(bpy)_3_Cl_2_·H_2_O (1%) in 2 mL of CH_3_CN and stirred at rt under an Ar atmosphere and irradiation of a HP single LED (450 nm). Isolated yields after column chromatography. Diastereomeric ratio determined by ^1^H NMR.

Under the above optimized reaction conditions (entry 1, Table 1), the reaction scope of 1,4-dihydroquinoxalin-2-one derivatives with trifluoroacetophenone **2a** was first studied (Scheme 2). A range of dihydroquinoxalin-2-ones were suitable for this reaction obtaining good yields, although without diastereoselectivity (almost 1:1 dr for all the examples). Initially, we evaluated the effect of the protecting group at the nitrogen of the amine of dihydroquinoxalin-2-one **1**. The reaction tolerates different benzylic substituents, affording the corresponding trifluoromethyl carbinols **3ba** and **3ca** with good yields. Moreover, dihydroquinoxalin-2-one **1d** bearing a heteroaromatic benzyl moiety furnished product **3da** in good yield. Additionally, the group CH_2_CO_2_Me is allowed giving the corresponding quinoxalin-2-one **3ea**, although with lower yield (55%). Moreover, 1,4-disubstituted-3,4-dihydroquinoxalin-2-ones could be used under the optimized reaction conditions giving the corresponding products **3fa** and **3ga** with good yields (60% for both examples). The substitution in the parent aromatic ring of 3,4-dihydroquinoxalin-2-one was also examined under the optimal reaction conditions. To our delight, 3,4-dihydroquinoxalin-2-one bearing an electron-donating (Me) or electron-withdrawing (Br) group at the 7-position on the aromatic ring furnished the corresponding tertiary alcohols **3ha** and **3ia** in good yields (59% and 68%, respectively). Nevertheless, 3,4-dihydroquinoxalin-2-ones with a methyl substituent at either the 5 or the 8 position were not suitable substrates for our methodology. Interestingly, the less electron-rich substrate **1l** bearing a secondary amine was found to be competent under the reaction conditions furnishing the product **3la** in moderate yield.

**Scheme 3:**
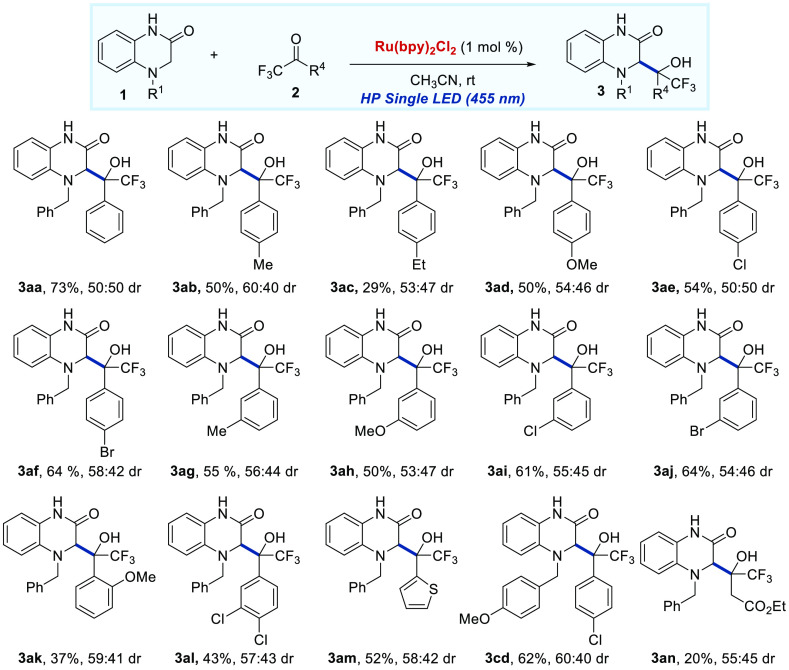
Scope of the Radical Addition Reaction Regarding the Trifluoromethyl Aryl Ketones **2** Reaction conditions: **1** (0.26 mmol), **2** (0.2 mmol), and Ru(bpy)_3_Cl_2_·H_2_O (1%) in 2 mL of CH_3_CN and stirred at rt under an Ar atmosphere with irradiation of a HP single LED (455 nm). Isolated yields after column chromatography. Diastereomeric ratio determined by ^1^H NMR.

Subsequently, the scope and limitation of various trifluoromethyl aryl ketones **2** were explored (Scheme 3). The incorporation of either electron-donating groups (Me, Et, or MeO) or electron-withdrawing groups (Cl or Br) on the benzene ring of trifluoromethyl ketones **2** had no obvious impact on the reaction, and the corresponding products (**3aa**–**3al**) were obtained in 40–70% yields. The presence of a MeO group in the *ortho* position to the carbonyl group of **2** had a slight influence on obtaining the trifluoromethyl alcohol **3ak** with lower yield (37%), but somewhat higher diastereoselectivity (59:41 dr). Furthermore, trifluoromethyl ketones with two substituents at the aromatic ring or bearing a heteroaromatic ring were tested in the radical addition reaction, affording the products **3al** and **3am** with moderate yields. Besides, non-aromatic trifluoromethyl ketone **2n** was found to be able to react under the optimized conditions but provide the expected product (**3an**) in low yield.

**Scheme 4:**
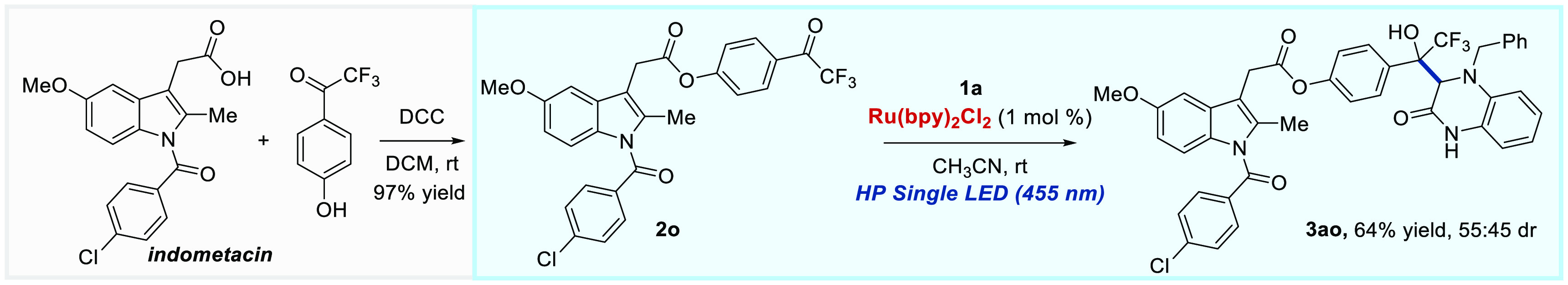
Synthesis of Indometacin-Derived Trifluoroacetophenone **2o** and Its Subsequent Radical Addition Reaction with Dihydroquinoxalin-2-one **1a** Reaction conditions: **1a** (0.26 mmol), **2o** (0.2 mmol), and Ru(bpy)_3_Cl_2_·H_2_O (1%) in 2 mL of CH_3_CN and stirred at rt under an Ar atmosphere and irradiation of a HP Single LED (455 nm). Isolated yield after column chromatography. Diastereomeric ratio determined by ^1^H NMR.

Finally, the utility of our protocol was further applied to trifluoroacetophenone **2o** resulting in the incorporation of the indometacin core, a nonsteroidal anti-inflammatory drug (Scheme 4). Hence, indometacin was coupled with *p*-hydroxytrifluoroacetophenone in the presence of DCC, obtaining the corresponding ester **2o** in 97% yield. This derivative was subjected to our photoredox radical addition protocol furnishing the desired dihydroquinoxalin-2-one derivative bearing the indometacin scaffold (**3ao**) in 64% yield.

**Scheme 5:**
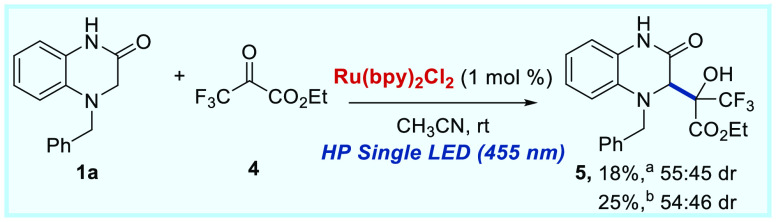
Scope of the Radical Addition Reaction Regarding the 4-Benzyl-3,4-dihydroquinoxalin-2(1*H*)-one **1a** with Ethyl 3,3,3-trifluoropyruvate **4**. Reaction conditions: **1a** (0.13 mmol), **4** (0.1 mmol), and Ru(bpy)_3_Cl_2_·H_2_O (1%) in 1 mL of CH_3_CN and stirred at rt under an Ar atmosphere and irradiation of a HP single LED (450 nm). ^*b*^0.1 mmol of **1a** and 0.13 mmol of **4** were used. Isolated yields after column chromatography. Diastereomeric ratio determined by ^1^H NMR.

To further expand the substrate scope of this reaction, other trifluoromethyl ketones were used as sources of trifluoromethyl ketyl radicals. As disclosed in Scheme 5, ethyl 3,3,3-trifluoropyruvate **4** proved to be a suitable substrate for this transformation, even though the corresponding alcohol product **5** was isolated in low yield.

**Scheme 6:**
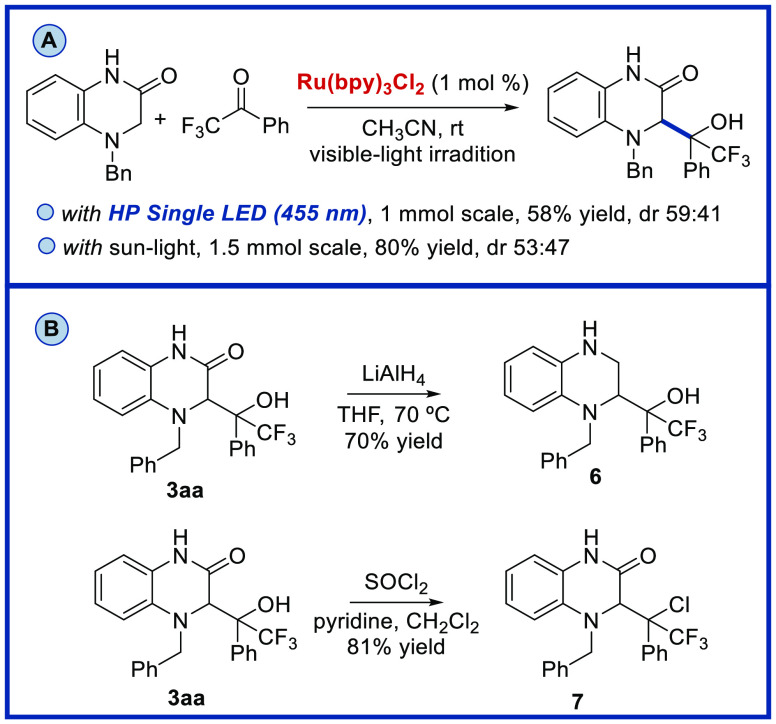
(A) 1 Mmol Scale Reactions Using HP Single Blue LED or Sunlight Irradiation an Ar Atmosphere. (B) Synthetic Transformations. Isolated Yields after Column Chromatography

To demonstrate the utility of our photocatalytic protocol for the synthesis of dihydroquinoxalin-2-ones bearing a trifluoromethyl alcohol, we also performed the reaction of **1a** and trifluoroacetophenone **2a** at 1 mmol scale under HP Single Blue LED or sunlight irradiation (Scheme 6A). Interestingly, when the reaction was performed under sunlight irradiation, we obtained the product **3aa** with higher yield (80%). Finally, we carried out the reduction of the amide group present in the dihydroquinoxalin-2-one derivative **3** with LiAlH_4_ in THF at 70 °C, obtaining the corresponding dihydroquinoxaline **6** with 70% yield (Scheme 6B). Moreover, we attempted dehydration of the product **3aa** using SOCl_2_/pyridine;^14^ however, we obtained the quinoxalin-2-one derivative **7** in 81% yield from the nucleophilic substitution of the OH group by Cl.

**Figure 1:**
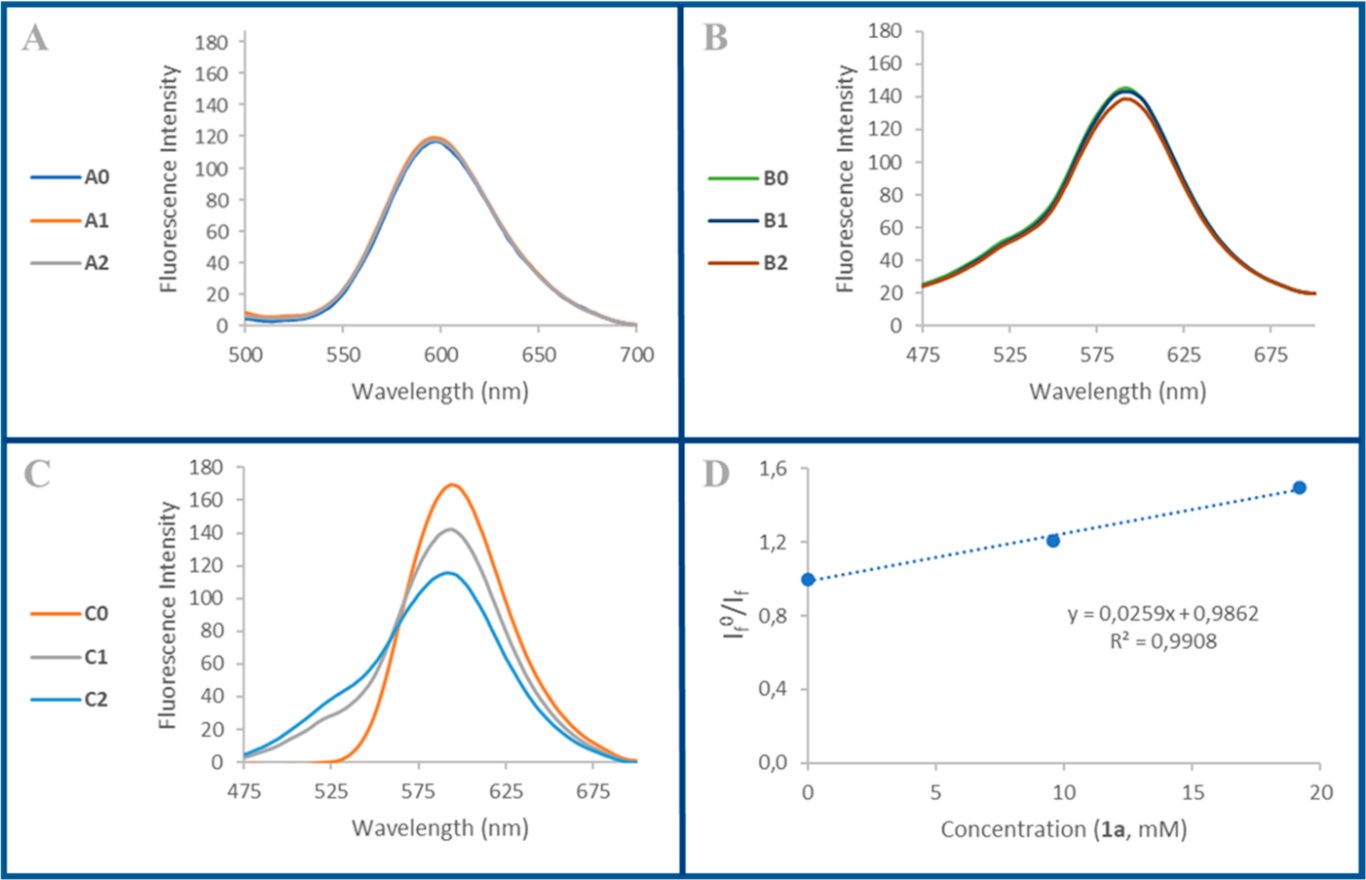
Emission spectrum of Ru(bpy)_3_Cl_2_·6H_2_O (0.02 mM) for (A) solutions of increasing concentration of trifluoroacetophenone **2a** (A0 = 0 mM; A1 = 9.6 mM; A2 = 19.2 mM); (B) solutions of constant concentration of quinoxalin-2-one **1a** (9.6 mM) and increasing concentration of trifluoroacetophenone **2a** (B0 = 0 mM; B1 = 9.6 mM; B2 = 19.2 mM); and (C) solutions of constant concentration of trifluoroacetophenone **2a** (9.6 mM) and increasing concentration of quinoxalin-2-one **1a** (C0 = 0 mM; C1 = 9.6 mM; C2 = 19.2 mM). (D) Stern–Volmer plot for the emission spectrum (at 600 nm) depicted in (C).^17^

To gain insight into the mechanism of the reaction, we first examined the reduction potential values of each component in the reaction mixture. Ru(bpy)_3_Cl_2_ potentials in MeCN are well stablished, and this complex can act either as an oxidant with *E_1/2_ = +0.77 V vs SCE or as a reductant with *E_1/2_= −0.81 V vs SCE.^15^ Reduction potentials of several substituted 2,2,2-trifluoroacetophenones were reported in 1990 by Liu.^16^ This authors examined the effect of several substituents at the aromatic ring and found that the parent 2,2,2-trifluoroacetophenone (**2a**) has a reduction potential of −1.40 V vs SCE. Besides, we have previously reported the reduction potential of 4-benzylquinoxalin-2-one **1a** in an earlier work (+0.80 V vs SCE).^12c^ Based on the thermodynamics of canonical photoredox reactivity, we can exclude a Single Electron Transfer (SET) event between the excited state of the ruthenium catalyst and either the trifluoroacetophenone **2a** (via an oxidative quenching pathway) or 4-benzylquinoxalin-2-one **1a** (via a reductive quenching pathyway). This assumption is further confirmed by luminescence quenching studies in which both trifluoroacetophenone **2a** and 4-benzylquinoxalin-2-one **1a** were unable to independently deactivate the excited state of Ru(bpy)_3_Cl_2_ (Figure 1A).

These findings led us to explore other pathways dictating this reactivity. First, we performed a Stern–Volmer quenching study maintaining the amount of both the 4-benzylquinoxalin-2-one **1a** and Ru(bpy)_3_Cl_2_ in each solution and varying the amount of trifluoroacetophenone **2a**. After recording the emission spectrum of each sample, only a modest change was observed, which can be attributed to experimental errors (Figure 1B). Then, we repeated the same experiment but now maintain constant the amount of trifluoroacetophenone **2a** and Ru(bpy)_3_Cl_2_ and vary the concentration of 4-benzylquinoxalin-2-one **1a**. This time we obtained a set of emission spectra consistent with a Stern–Volmer relationship (Figure 1C), and therefore we can establish a Stern–Volmer constant (*K*_SV_) of 25.9 M^–1^ (Figure 1D).^17^ This study revealed that the excited state of Ru(bpy)_3_Cl_2_ can be quenched (presumably via a SET) by 4-benzylquinoxalin-2-one **1a** only if trifluoroacetophenone **2a** is present. These finding can be explained by admitting an interaction between **1a** and **2a** that makes **1a** more prone to oxidation.

At this point, we wanted to explore the interaction between **1a** and **2a**. We envisioned that a solution of 4-benzylquinoxalin-2-one **1a** in MeCN-*d*^*3*^ could be titrated with trifluoroacetophenone **2a** while monitoring the process by NMR.^17^ Unfortunately, we did not observe any NMR change that could be attributed to an interaction between **1a** and **2a** (Figure S1),^17^ especially regarding the amidic N–H bond of **1a** and a possible Proton Coupled Electron Transfer process like those reported by Knowles.^18^

**Figure 2:**
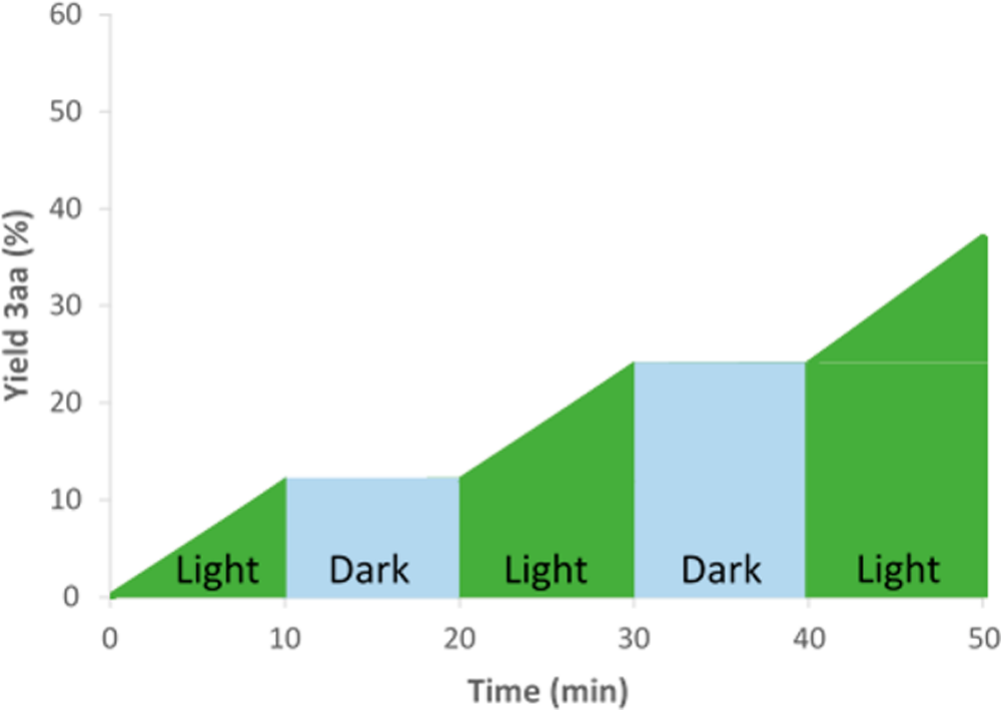
On/off experiment for the radical addition reaction between dihydroquinoxalin-2-one **1a** and trifluoroacetophenone **2a**.

Furthermore, to confirm the participation of a closed photoredox cycle and to exclude a radical chain process, we determined the quantum yield of the process. First, we determined the photon flux of our photochemical setup using standard ferrioxalate actinometry (Figure S3),^17^ and then, we found out that the quantum yield of our methodology is as low as Φ = 0.21 ± 0.02, showing that the participation of a chain mechanism is unlikely (Figure S4).^17^ We have also performed a light/off experiment (Figure 2) for the reaction between **1a** and **2a**, showing as well that the mechanism should be a closed photoredox cycle.

**Scheme 7:**
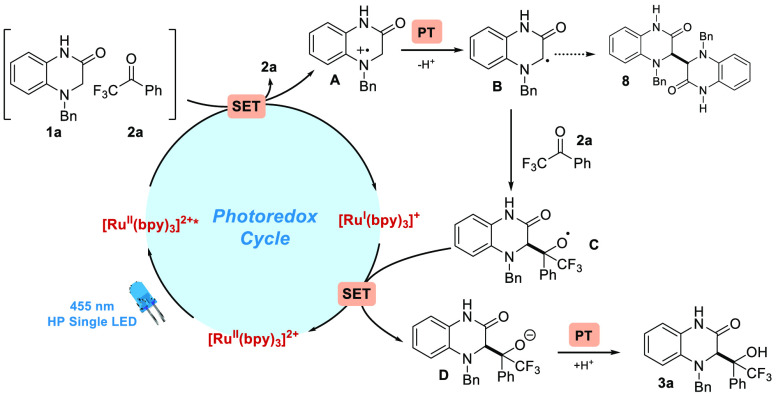
Mechanistic Hypothesis for the Generation of **3aa** from **1a** and **2a** under Photoredox Conditions

With all this information, we were able to postulate a plausible reaction mechanism for our photochemical protocol (Scheme 7). The absorption of a 455 nm photon promotes Ru(bpy)_3_Cl_2_ to its excited state. Then, a sort of aggregate between **1a** and **2a** facilitated the SET from the excited photocatalyst to **1a**, yielding the corresponding radical cation **A** as well as the Ru^I^ form of the catalyst.^19^ The radical cation **A** can experience Proton Transfer (PT) to form the α-amino radical **B**, which has a nucleophilic character and can react with trifluoroacetophenone **2a** to generate *O*-centered radical **C**. This radical **B** can react with itself through an unproductive pathway to form the dimeric compound **8**.^12c^ The Ru^I^ species, which has a strong reductive behavior (*E*^II/I^_1/2_ = −1.33 V vs SCE), is able to reduce radical **C** to its corresponding alkoxide anion **D**. Finally, another PT event over alkoxide **D** furnishes the desired product **3aa**.

## Conclusion

In summary, we have described the synthesis of trifluoromethyl tertiary alcohols bearing a dihydroquinoxalin-2-one framework (25 examples) through a photocatalytic radical addition of dihydroquinoxalin-2-ones to trifluoromethyl ketones enabled by a reductive quenching cycle of Ru(bpy)_3_Cl_2_. Our protocol provides rapid and efficient access to synthetic useful dihydroquinoxalin-2-ones bearing trifluoromethyl and hydroxyl groups under mild reaction conditions and simple operational protocol using HP Single LED of 455 nm. It is also important to note that our protocol is operative in the late-stage functionalization of a value-added indometacin-derived trifluoroacetophenone substrate. In addition, the reaction can be scaled up to 1 mmol using HP Single LED (455 nm) as well as sunlight irradiation. Moreover, several synthetic transformations have been performed, and a plausible reaction mechanism has been postulated.

## Experimental Section

### General Methods

Reactions were carried out in Schlenk tubes oven-dried overnight at 120 °C. Commercial reagents were used as purchased. Reactions were monitored by TLC analysis using Merck Silica Gel 60 F-254 thin layer plates. Flash column chromatography was performed on Merck silica gel 60, 0.040–0.063 mm and visualized using both a UV lamp (254 nm) and then a CAM solution (an aqueous solution of ceric ammonium molybdate). Melting points were determined in capillary tubes. NMR spectra were run at 300 MHz for ^1^H and 75 MHz for ^13^C using residual nondeuterated solvent as internal standard (CHCl_3_: δ 7.26 and 77.00 ppm, respectively). Chemical shifts are given in ppm. The carbon type was determined by DEPT experiments. High resolution mass spectra (ESI) were recorded on a AB SCIEX Triple TOF spectrometer equipped with an electrospray source with a capillary voltage of 4.5 kV (ESI). MeCN was degassed by three freeze–pump–thaw cycles and stored over 3 Å MS for 48 h at least. Prior to use, MeCN was bubbled with Ar for 10 min. Commercially available High Power Single LEDs manufactured by Intelligent LED Solutions (purchased from Farnell, internal reference 3583117) with an emission band centered at 455 nm were used as a light source. These LEDs lay on an aluminum block to ensure proper heat dissipation. Photochemical reactions were conducted in conventional borosilicate glass Schlenk flasks situated at 2 cm to the HP Single LED. Ru(bpy)_4_Cl_2_·6H_2_O and Eosin Y were purchased by Merck-Aldrich. 4-CzIPN^20^ and dihydroquinoxalinones^12c^**1** were known compounds and were synthesized according to literature-reported procedures.

### Specific Procedure for the Synthesis of Indometacin-Derived Trifluoroacetophenone 2o

To a stirred solution of commercially available indometacin (196.8 mg, 0.55 mmol, 1.1 equiv) in DCM (5 mL) were added *p*-hydroxytrifluoroacetophenone (95.1 mg, 0.5 mmol, 1 equiv) and DCC (155 mg, 0.75 mmol, 1.5 equiv), and the resulting mixture was stirred at room temperature for 16 h. Then, the crude reaction mixture was filtered through a pad of Celite eluting with Et_2_O. This yellow solution was concentrated under reduced pressure, and the residue was purified by column chromatography using hexane:EtOAc as eluent to afford the desired product (257 mg, 0.485 mmol, 97% yield) as a white solid.

^1^H NMR (300 MHz, CDCl_3_) δ 8.29–7.93 (m, 2H), 7.68 (d, *J* = 8.6 Hz, 2H), 7.48 (d, *J* = 8.7 Hz, 2H), 7.28 (d, *J* = 9.0 Hz, 2H), 7.04 (d, *J* = 2.5 Hz, 1H), 6.88 (d, *J* = 9.0 Hz, 1H), 6.71 (dd, *J* = 9.0, 2.5 Hz, 1H), 3.95 (s, 2H), 3.84 (s, 3H), 2.47 (s, 3H); ^19^F{^1^H} NMR (282 MHz, CDCl_3_) δ −71.87; ^13^C{^1^H} NMR (75 MHz, CDCl_3_) δ 179.2 (q, *J*_C–F_ = 35.4 Hz, C), 168.3 (C), 168.2 (C), 156.1 (C), 139.4 (C), 136.4 (C), 133.6 (C), 131.9 (q, *J*_C–F_ = 2.0 Hz, CH), 131.2 (CH), 130.8 (C), 130.3 (C), 129.2 (C+CH), 127.4 (C), 122.3 (CH), 116.5 (q, *J*_C–F_ = 290.8 Hz, C), 115.0 (CH), 111.7 (CH), 111.2 (C), 101.2 (CH), 55.7 (CH_3_), 30.5 (CH_2_), 13.4 (CH_3_); HRMS (ESI/Q-TOF) *m*/*z* [M + H]^+^ C_27_H_20_ClF_3_NO_5_^+^ Calcd for 530.0977; Found 530.0984.

### General Procedure for the Photocatalytic Radical Addition of Quinoxalin-2-ones to Trifluoroacetophenone (GP-1)

In an oven-dried Schlenk tube, the corresponding quinoxalin-2-one **1** (0.26 mmol, 0.13 equiv) and Ru(bpy)_3_Cl_2_·6H_2_O (1.5 mg, 1 mol %) were placed and the flask was evacuated and backfilled with Ar (×3). Then, anhydrous and degassed CH_3_CN (2 mL), as well as the corresponding trifluoroacetophenone **2** (0.2 mmol, 0.1 equiv), was added via syringe. The reaction mixture was stirred under the irradiation of a High-Power Blue LED (455 nm) while being cooled with a fan to keep the temperature at 20 °C. Once the reaction was finished (TLC), the mixture was purified by column chromatography using hexane:EtOAc or hexane:Et_2_O mixtures to afford compound **3**.

### Specific Procedure for the Photocatalytic Radical Addition of 4-Benzyl-3,4-dihydroquinoxalin-2(1*H*)-one (1a) to Ethyl 3,3,3-trifluoropyruvate (4) (SP-1)

In an oven-dried Schlenk tube, the corresponding 4-benzyl-3,4-dihydroquinoxalin-2(1*H*)-one (**1a**, 47.6 mg, 0.2 mmol, 0.1 equiv) and Ru(bpy)_3_Cl_2_ (1.5 mg, 1 mol %) were placed, and the flask was evacuated and backfilled with Ar (×3). Then, anhydrous and degassed CH_3_CN (2 mL), as well as 3,3,3-trifluoropyruvate (**4**, 34 μL, 0.26 mmol, 1.3 equiv), was added via syringe. The reaction mixture was stirred under the irradiation of a High-Power Blue LED (455 nm) while being cooled with a fan to keep the temperature at 20 °C. Once the reaction was finished (TLC), the mixture was purified by column chromatography using hexane:EtOAc mixtures to afford compound **5**.

### Specific Procedure for the Photocatalytic Radical Addition of 4-Benzyl-3,4-dihydroquinoxalin-2(1*H*)-one (1a) to 2,2,2-Trifluoroacetophenone (2a) 1 mmol Scale Reaction (SP-2)

In an oven-dried Schlenk tube, 4-benzyl-3,4-dihydroquinoxalin-2(1*H*)-one (**1a**, 312 mg, 1.3 mmol, 1.3 equiv) and Ru(bpy)_3_Cl_2_·6H_2_O (5.0 mg, 1 mol %) were placed, and the flask was evacuated and backfilled with Ar (×3). Then, anhydrous and degassed CH_3_CN (7 mL), as well as 2,2,2-trifluoroacetophenone (**2a**, 212 μL, 1.0 mmol 1 equiv) was added via syringe. The reaction mixture was stirred under the irradiation of several High-Power Blue LEDs (455 nm) while being cooled with a fan to keep the temperature at 20 °C. Once the reaction was finished (TLC), the mixture was purified by column chromatography using hexane:EtOAc mixtures to afford compound **3aa** (240 mg, 0.58 mmol, 58% yield) as a mixture of diastereoisomers (**3aa′** and **3aa″**, 59:41 dr).

### Specific Procedure for the photocatalytic radical addition of 4-benzyl-3,4-dihydroquinoxalin-2(1*H*)-one (1a) to 2,2,2-trifluoroacetophenone (2a) under sunlight irradiation (SP-3)

In an oven-dried Schlenk tube, 4-benzyl-3,4-dihydroquinoxalin-2(1*H*)-one (**1a**, 465 mg, 1.95 mmol, 1.3 equiv) and Ru(bpy)_3_Cl_2_·6H_2_O (7.5 mg, 1 mol %) were placed and the flask was evacuated and backfilled with Ar (×3). Then, anhydrous and degassed CH_3_CN (10 mL), as well as 2,2,2-trifluoroacetophenone (**2a**, 316 μL, 1.5 mmol 1 equiv) was added via syringe. The reaction mixture placed at the upper part of the building in sunny hours and was stirred for 2.5 h. Once the reaction was finished (TLC), the mixture was purified by column chromatography using hexane:EtOAc mixtures to afford compound **3aa** (495 mg, 1.2 mmol, 80% yield) as a mixture of diastereoisomers (**3aa′** and **3aa″**, 53:47 dr).

### Specific Procedure for the reduction of 3a (SP-4)

In a 50 mL round bottomed flask equipped with a condenser, compound **3aa** (78.4 mg, 0.19 mmol, 1 equiv) was placed. The flask was purged with N_2_ and then dry THF (5 mL) was added. The solution was cooled to 0 °C and LiAlH_4_ (125 μL, 0.76 mmol, 4 equiv., 4 M in THF) was added dropwise. The reaction mixture was progressively warmed up and heated (in an oil bath) at reflux temperature for 2 h. After this period, the reaction mixture was cooled again to 0 °C and the excess LiAlH_4_ was quenched with sat. aq. NH_4_Cl (5 mL) and the organics were extracted with DCM (×3). The combined organic layers were washed with brine (×1) and dried over anhydrous MgSO_4_. After evaporating the solvent, the residue was purified by column chromatography using hexane:EtOAc mixtures, obtaining quinoxaline derivative **6**.

### Specific Procedure for the Chlorination of 3aa (SP-5)

In a 10 mL round bottomed flask equipped with a condenser, compound **3aa** (26.9 mg, 0.07 mmol, 1 equiv) was placed. The flask was purged with N_2_ and then DCM (2 mL) was added. SOCl_2_ (10 μL, 0.13 mmol, 2 equiv) and pyridine (11 μL, 0.13 mmol, 2 equiv) were successively added and the reaction mixture was stirred at room temperature under N_2_ for 2 h. The reaction mixture was directly purified by column chromatography using hexane:Et_2_O mixture to afford compound **7**.

### 4-Benzyl-3-(2,2,2-trifluoro-1-hydroxy-1-phenylethyl)-3,4-dihydroquinoxalin-2(1*H*)-one (3aa)

Using 4-benzyl-3,4-dihydroquinoxalin-2(1*H*)-one (**1a**, 62 mg, 0.26 mmol, 1.3 equiv) and 2,2,2-trifluoroacetophenone (**2a**, 28.1 μL, 0.2 mmol, 1 equiv), according to GP-1, compound **3aa** was obtained as a mixture of diastereoisomers (50:50 dr) that were separated by column chromatography using hexane:EtOAc mixtures (from 9:1 to 7:3): **3aa′** (29.7 mg, 0.07 mmol, 36% yield, brown oil) and **3aa″** (30.1 mg, 0.07 mmol, 36% yield, brown oil).

#### Characterization of **3aa′**

^1^H NMR (300 MHz, CDCl_3_) δ 9.64 (s, 1H), 7.64 (dd, *J* = 6.6, 2.9 Hz, 2H), 7.46–7.36 (m, 3H), 7.22 (tdd, *J* = 4.5, 3.6, 1.5 Hz, 3H), 7.01 (ddd, *J* = 8.6, 7.3, 1.4 Hz, 1H), 6.97–6.90 (m, 3H), 6.82 (td, *J* = 7.5, 1.4 Hz, 1H), 6.63 (dd, *J* = 7.8, 1.3 Hz, 1H), 4.82 (s, 1H), 4.59 (d, *J* = 15.8 Hz, 1H), 4.38 (s, 1H), 3.48 (d, *J* = 15.8 Hz, 1H); ^19^F{^1^H} NMR (282 MHz, CDCl_3_) δ −73.17; ^13^C{^1^H} NMR (75 MHz, CDCl_3_) δ 165.7 (C), 136.3 (C), 134.7 (C), 133.1 (C), 128.9 (CH), 128.8 (CH), 128.3 (CH), 127.8 (CH), 127.4 (CH), 126.6 (C), 126.5 (q, *J* = 1.8 Hz, CH), 125.19 (q, *J* = 287.2 Hz, CF_3_), 124.7 (CH), 120.8 (CH), 116.9 (CH), 116.0 (CH), 79.4 (q, *J* = 28.2 Hz, C), 67.2 (CH), 57.4 (CH_2_); HRMS (ESI/Q-TOF) *m*/*z* [M + H]^+^ C_23_H_20_F_3_N_2_O_2_^+^ Calcd for 413.1471; Found 413.1465.

#### Characterization of **3aa″**

^1^H NMR (300 MHz, CDCl_3_) δ 8.95 (s, 1H), 7.47 (d, *J* = 7.6 Hz, 2H), 7.24–7.14 (m, 4H), 7.13–7.02 (m, 4H), 6.92 (ddd, *J* = 8.2, 7.4, 1.4 Hz, 1H), 6.79 (d, *J* = 7.7 Hz, 1H), 6.63 (td, *J* = 7.7, 1.2 Hz, 1H), 6.38 (dd, *J* = 7.8, 1.3 Hz, 1H), 4.81 (d, *J* = 16.0 Hz, 1H), 4.73 (s, 1H), 4.66 (s, 1H), 4.21 (d, *J* = 16.0 Hz, 1H). ^19^F{^1^H} NMR (282 MHz, CDCl_3_) δ −74.24. ^13^C{^1^H} NMR (75 MHz, CDCl_3_) δ 164.8 (C), 136.6 (C), 134.2 (C), 133.6 (C), 128.82 (CH), 128.79 (CH), 127.8 (CH), 127.7 (CH), 127.3 (CH), 126.9 (q, *J* = 1.8 Hz, CH), 125.7 (C), 124.72 (CH), 124.68 (q, *J* = 265.9 Hz, CF_3_), 120.0 (CH), 116.4 (CH), 115.5 (CH), 78.6 (q, *J* = 27.1 Hz, C), 66.4 (CH), 56.5 (CH_2_); HRMS (ESI/Q-TOF) *m*/*z* [M + H]^+^ C_23_H_20_F_3_N_2_O_2_^+^ Calcd for 413.1471; Found 413.1462.

### 4-(4-Methoxybenzyl)-3-(2,2,2-trifluoro-1-hydroxy-1-phenylethyl)-3,4-dihydroquinoxalin-2(1*H*)-one (3ba)

Using 4-(4-methoxybenzyl)-3,4-dihydroquinoxalin-2(1*H*)-one (**1b**, 69.8 mg, 0.26 mmol, 1.3 equiv) and 2,2,2-trifluoroacetophenone (**2a**, 28.1 μL, 0.2 mmol, 1 equiv), according to GP-1, compound **3ba** was obtained as a mixture of diastereoisomers (52:48 dr) that cannot be separated by column chromatography using hexane:EtOAc mixtures (from 9:1 to 8:2): **3ba′** + **3ba″** (74.5 mg, 0.16 mmol, 90% yield, brown oil).

^1^H NMR (300 MHz, CDCl_3_) δ 9.42 (s, 1H), 9.03 (s, 1H), 7.61 (dd, *J* = 6.6, 2.9 Hz, 2H), 7.48 (d, *J* = 7.6 Hz, 2H), 7.41–7.32 (m, 3H), 7.22–7.14 (m, 1H), 7.13–7.04 (m, 2H), 7.04–6.90 (m, 5H), 6.88–6.71 (m, 9H), 6.68–6.57 (m, 1H), 6.33 (dd, *J* = 7.8, 1.4 Hz, 1H), 4.90–4.69 (m, 3H), 4.65 (s, 1H), 4.51 (d, *J* = 15.3 Hz, 1H), 4.34 (s, 1H), 4.14 (d, *J* = 15.6 Hz, 1H), 3.75–3.67 (m, 6H), 3.42 (d, *J* = 15.3 Hz, 1H). ^19^F{^1^H} NMR (282 MHz, CDCl_3_) δ −73.26, −74.23. ^13^C{^1^H} NMR (75 MHz, CDCl_3_) δ 165.8 (C), 165.0 (C), 159.3 (C), 159.2 (C), 134.8 (C), 134.3 (C), 133.78 (C), 133.3 (C), 128.9 (CH), 128.8 (CH), 128.7 (CH), 128.6 (C), 128.3 (CH), 128.2 (C), 127.7 (CH), 127.0 (q, *J* = 1.7 Hz, CH), 126.8 (C), 126.6 (q, *J* = 1.7 Hz, CH), 125.9 (C), 124.8 (CH), 124.7 (CH), 120.9 (CH), 120.0 (CH), 117.3 (CH), 116.8 (CH), 115.9 (CH), 115.6 (CH), 114.22 (CH), 114.19 (CH), 79.3 (q, *J* = 28.2 Hz, C), 78.5 (q, *J* = 27.1 Hz, C), 66.6 (CH), 66.0 (CH), 57.2 (CH_2_), 56.4 (CH_2_), 55.4 (CH_3_), 55.2 (CH_3_); HRMS (ESI/Q-TOF) *m*/*z* [M + H]^+^ C_24_H_22_F_3_N_2_O_3_^+^ Calcd for 443.1577; Found 443.1583.

### 3-(2,2,2-Trifluoro-1-hydroxy-1-phenylethyl)-4-(4-(trifluoromethyl)benzyl)-3,4-dihydroquinoxalin-2(1*H*)-one (3ca)

Using 4-(4-(trifluoromethyl)benzyl)-3,4-dihydroquinoxalin-2(1*H*)-one (**1c**, 79.6 mg, 0.26 mmol, 1.3 equiv) and 2,2,2-trifluoroacetophenone (**2a**, 28.1 μL, 0.2 mmol, 1 equiv), according to GP-1, compound **3ca** was obtained as a mixture of diastereoisomers (52:48 dr) that cannot be separated by column chromatography using hexane:EtOAc mixtures (from 9:1 to 7:3): **3ca′** + **3ca″** (73.0 mg, 0.152 mmol, 76% yield, brown oil).

^1^H NMR (300 MHz, CDCl_3_) δ 9.61 (s, 1H), 9.19 (s, 1H), 7.63 (dd, *J* = 6.6, 2.8 Hz, 2H), 7.52–7.37 (m, 9H), 7.24–7.06 (m, 5H), 7.08–6.95 (m, 3H), 6.91 (td, *J* = 8.2, 1.4 Hz, 1H), 6.87–6.78 (m, 2H), 6.76–6.69 (m, 2H), 6.65 (td, *J* = 7.7, 1.1 Hz, 1H), 6.38 (dd, *J* = 7.8, 1.3 Hz, 1H), 4.82 (d, *J* = 16.4 Hz, 1H), 4.72–4.55 (m, 4H), 4.36 (s, 1H), 4.24 (d, *J* = 16.5 Hz, 1H), 3.53 (d, *J* = 16.4 Hz, 1H). ^19^F{^1^H} NMR (282 MHz, CDCl_3_) δ −62.58, −62.61, −73.09, −74.16. ^13^C{^1^H} NMR (75 MHz, CDCl_3_) δ 165.5 (C), 164.7 (C), 140.8 (q, *J* = 1.1 Hz, C), 140.6 (q, *J* = 1.1 Hz, C), 134.6 (C), 134.0 (C), 132.9 (C), 132.5 (C), 130.00 (q, *J* = 32.5 Hz, C), 129.98 (q, *J* = 32.4 Hz, C), 129.1 (CH), 129.0 (CH), 128.4 (CH), 127.8 (CH), 127.44 (CH), 127.41 (CH), 126.8 (q, *J* = 1.6 Hz, CH), 126.5 (q, *J* = 1.7 Hz, CH), 126.4 (C), 125.8 (q, *J* = 2.6 Hz, CH), 125.7 (q, *J* = 2.6 Hz, CH), 125.0 (CH), 124.8 (CH), 120.9 (CH), 120.3 (CH), 116.2 (CH), 116.1 (CH), 116.0 (CH), 115.7 (CH), 79.7 (q, *J* = 27.6 Hz, C), 78.8 (q, *J* = 27.6 Hz, C), 67.9 (CH), 66.9 (CH), 56.3 (CH_2_), 55.7 (CH_2_); HRMS (ESI/Q-TOF) *m*/*z* [M + H]^+^ C_24_H_19_F_6_N_2_O_2_^+^ Calcd for 481.1345; Found 481.1341.

### 4-(Thiophen-2-ylmethyl)-3-(2,2,2-trifluoro-1-hydroxy-1-phenylethyl)-3,4-dihydroquinoxalin-2(1*H*)-one (3da)

Using 4-(thiophen-2-ylmethyl)-3,4-dihydroquinoxalin-2(1*H*)-one (**1d**, 63.5 mg, 0.26 mmol, 1.3 equiv) and 2,2,2-trifluoroacetophenone (**2a**, 28.1 μL, 0.2 mmol, 1 equiv), according to GP-1, compound **3da** was obtained as a mixture of diastereoisomers (58:42 dr) that cannot be separated by column chromatography using hexane:EtOAc mixtures (from 9:1 to 8:2): **3da′** + **3da″** (51.0 mg, 0.12 mmol, 61% yield, brown oil).

^1^H NMR (300 MHz, CDCl_3_) δ 8.73 (s, 1H), 8.31 (s, 1H), 7.69–7.60 (m, 2H), 7.48 (d, *J* = 7.6 Hz, 2H), 7.44–7.36 (m, 3H), 7.20–6.80 (m, 13H), 6.73–6.62 (m, 3H), 6.30 (dd, *J* = 7.8, 1.2 Hz, 1H), 5.02–4.92 (m, 2H), 4.88 (s, 1H), 4.69–4.59 (m, 2H), 4.40 (d, *J* = 16.2 Hz, 1H), 4.35–4.29 (m, 1H), 3.49 (d, *J* = 16.1 Hz, 1H); ^19^F{^1^H} NMR (282 MHz, CDCl_3_) δ −73.61, −74.77; ^13^C NMR (75 MHz, CDCl_3_) δ 165.4 (C), 164.8 (C), 139.3 (C), 138.8 (C), 134.7 (C), 134.0 (C), 132.8 (C), 132.3 (C), 129.0 (CH), 128.8 (CH), 128.3 (CH), 127.6 (CH), 127.0 (C), 127.0 (C), 127.0 (CH), 126.8 (CH), 126.7 (CH), 126.6 (CH), 126.6 (CH), 126.6 (CH), 125.7 (CH), 125.6 (CH), 124.8 (CH), 124.8 (CH), 121.5 (CH), 120.8 (CH), 117.8 (CH), 117.5 (CH), 115.9 (CH), 115.5 (CH), 79.1 (q, *J*_C–F_ = 26.2 Hz, C), 78.1 (q, *J*_C–F_ = 25.9 Hz, C), 66.6 (CH), 65.7 (CH), 53.1 (CH_2_), 52.6 (CH_2_); HRMS (ESI/Q-TOF) *m*/*z* [M + H]^+^ C_21_H_18_F_3_N_2_O_2_S^+^ Calcd for 419.1036; Found 419.1037.

### Methyl 2-(3-Oxo-2-(2,2,2-trifluoro-1-hydroxy-1-phenylethyl)-3,4-dihydroquinoxalin-1(2*H*)yl) acetate (3ea)

Using methyl 2-(3-oxo-3,4-dihydroquinoxalin-1(2*H*)-yl)acetate (**1e**, 57.3 mg, 0.26 mmol, 1.3 equiv) and 2,2,2-trifluoroacetophenone (**2a**, 28.1 μL, 0.2 mmol, 1 equiv), according to GP-1, compound **3ea** was obtained as a mixture of diastereoisomers (51:49 dr) that cannot be separated by column chromatography using hexane:Et_2_O mixtures (from 5:5 to 2:8): **3ea′** + **3ea″** (43.2 mg, 0.11 mmol, 55% yield, brown oil).

^1^H NMR (300 MHz, CDCl_3_) δ 9.55 (s, 1H), 9.16 (s, 1H), 7.63 (dd, *J* = 6.8, 2.9 Hz, 2H), 7.56–7.47 (m, 2H), 7.38–7.30 (m, 3H), 7.19–7.04 (m, 3H), 7.02–6.80 (m, 4H), 6.80–6.73 (m, 1H), 6.73–6.64 (m, 2H), 6.39 (dd, *J* = 7.8, 1.4 Hz, 1H), 5.56 (s, 1H), 5.27 (s, 1H), 4.53 (s, 1H), 4.38 (d, *J* = 18.5 Hz, 1H), 4.23 (s, 1H), 4.07 (d, *J* = 18.5 Hz, 1H), 3.93 (d, *J* = 18.5 Hz, 1H), 3.64 (s, 3H), 3.63 (s, 3H), 3.09 (d, *J* = 18.5 Hz, 1H). ^19^F{^1^H} NMR (282 MHz, CDCl_3_) δ −73.38, −74.14. ^13^C{^1^H} NMR (75 MHz, CDCl_3_) δ 171.2 (C), 170.9 (C), 165.2 (C), 164.5 (C), 134.4 (C), 133.8 (C), 132.7 (C), 132.3 (C), 128.9 (CH), 128.8 (CH), 128.1 (CH), 127.6 (CH), 127.1 (C), 127.0 (q, *J* = 1.8 Hz, CH), 126.6 (C), 126.60 (q, *J* = 1.8 Hz, CH), 125.1 (q, *J* = 281.9 Hz, CF_3_), 124.9 (q, *J* = 286.9 Hz, CF_3_), 124.6 (CH), 124.5 (CH), 121.9 (CH), 121.3 (CH), 117.3 (CH), 116.1 (CH), 115.9 (CH), 79.0 (q, *J* = 27.6 Hz, C), 77.9 (q, *J* = 26.5 Hz, C), 69.0 (CH), 67.9 (CH), 56.9 (CH_2_), 56.3 (CH_2_), 52.4 (CH_3_), 52.3 (CH_3_); HRMS (ESI/Q-TOF) *m*/*z* [M + H]^+^ C_19_H_18_F_3_N_2_O_4_^+^ Calcd for 395.1213; Found 395.1217.

### 1,4-Dibenzyl-3-(2,2,2-trifluoro-1-hydroxy-1-phenylethyl)-3,4-dihydroquinoxalin-2(1*H*)-one (3fa)

Using 1,4-dibenzyl-3,4-dihydroquinoxalin-2(1*H*)-one (**1f**, 85.4 mg, 0.26 mmol, 1.3 equiv) and 2,2,2-trifluoroacetophenone (**2a**, 28.1 μL, 0.2 mmol, 1 equiv), according to GP-1, compound **3fa** was obtained as a mixture of diastereoisomers (53:47 dr) that cannot be separated by column chromatography using hexane:EtOAc mixtures (from 9:1 to 7:3): **3fa′** + **3fa″** (60.8 mg, 0.12 mmol, 60% yield, brown oil).

^1^H NMR (400 MHz, CDCl_3_) δ 7.64–7.58 (m, 2H), 7.49 (d, *J* = 7.9 Hz, 2H), 7.42–7.34 (m, 3H), 7.32–7.17 (m, 13H), 7.17–7.03 (m, 5H), 7.02–6.86 (m, 5H), 6.85–6.74 (m, 4H), 6.59 (t, *J* = 6.9 Hz, 1H), 6.47 (d, *J* = 8.1 Hz, 1H), 5.24 (d, *J* = 16.0 Hz, 1H), 5.11–4.76 (m, 6H), 4.71–4.58 (m, 2H), 4.56 (s, 1H), 4.32 (d, *J* = 15.9 Hz, 1H), 3.60 (d, *J* = 15.6 Hz, 1H); ^19^F{^1^H} NMR (282 MHz, CDCl_3_) δ −73.67, −74.55. ^13^C{^1^H} NMR (75 MHz, CDCl_3_) δ 164.4 (C), 163.9 (C), 136.7 (C), 136.3 (C), 135.9 (C), 135.5 (C), 134.9 (C), 134.7 (C), 134.4 (C), 134.3 (C), 129.6 (C), 128.86 (CH), 128.79 (CH), 128.75 (CH), 128.70 (CH), 128.64 (CH), 128.59 (CH), 128.1 (CH), 127.9 (CH), 127.8 (CH), 127.7 (CH), 127.6 (CH), 127.4 (CH), 127.3 (CH), 127.2 (CH), 126.9 (CH), 126.5 (q, *J* = 1.8 Hz, CH), 126.2 (CH), 124.8 (q, *J* = 286.4 Hz, CF_3_), 124.5 (CH), 124.4 (CH), 121.1 (CH), 120.1 (CH), 117.9 (CH), 117.2 (CH), 115.9 (CH), 115.7 (CH), 78.33 (q, *J* = 27.1 Hz, C), 78.33 (q, *J* = 27.1 Hz, C), 67.4 (CH), 66.8 (CH), 57.9 (CH_2_), 56.9 (CH_2_), 46.2 (CH_2_), 45.7 (CH_2_); HRMS (ESI/Q-TOF) *m*/*z* [M + H]^+^ C_30_H_26_F_3_N_2_O_2_^+^ Calcd for 503.1941; Found 503.1937.

### 4-Benzyl-1-methyl-3-(2,2,2-trifluoro-1-hydroxy-1-phenylethyl)-3,4-dihydroquinoxalin-2(1*H*)-one (3ga)

Using 4-benzyl-1-methyl-3,4-dihydroquinoxalin-2(1*H*)-one (**1g**, 65.6 mg, 0.26 mmol, 1.3 equiv) and 2,2,2-trifluoroacetophenone (**2a**, 28.1 μL, 0.2 mmol, 1 equiv), according to GP-1, compound **3ga** was obtained as a mixture of diastereoisomers (53:47 dr) that cannot be separated by column chromatography using hexane:EtOAc mixtures (from 9:1 to 7:3): **3ga′** + **3ga″** (50.4 mg, 0.12 mmol, 60% yield, colorless oil). Representative NMR signals for either the major and the minor diastereoisomer are labeled with one or two asterisks, respectively.

^1^H NMR (300 MHz, CDCl_3_) δ 7.56–7.49 (m, 2H*), 7.40–7.30 (m, 6H), 7.26–7.17 (m, 5H), 7.12–6.99 (m, 5H), 6.98–6.85 (m, 6H), 6.77 (ddd, *J* = 12.9, 8.1, 1.4 Hz, 2H), 6.65 (ddd, *J* = 8.1, 7.3, 1.5 Hz, 1H**), 6.42 (dd, *J* = 8.1, 1.4 Hz, 1H**), 5.07 (s, 1H*), 4.96 (s, 1H**), 4.87 (d, *J* = 16.0 Hz, 1H**), 4.72 (s, 1H**), 4.48 (d, *J* = 15.4 Hz, 1H*), 4.37–4.32 (m, 2H), 3.52 (d, *J* = 15.4 Hz, 1H*), 3.26 (s, 3H*), 3.13 (s, 3H**). ^19^F{^1^H} NMR (282 MHz, CDCl_3_) δ −73.88*, −74.11**. ^13^C{^1^H} NMR (75 MHz, CDCl_3_) δ 164.3 (C*), 163.9 (C**), 136.5 (C), 136.0 (C), 134.9 (C), 134.3 (C), 134.2 (C), 133.9 (C), 130.4 (C), 128.73 (CH), 128.71 (CH), 128.7 (CH), 128.6 (CH), 128.0 (CH), 127.9 (CH), 127.72 (CH), 127.70 (CH), 127.65 (CH), 127.3 (CH), 126.8 (q, *J* = 1.7 Hz, CH), 126.5 (q, *J* = 2.2 Hz, CH), 125.1 (q, *J* = 286.9 Hz, CF_3_), 124.9 (q, *J* = 293.0 Hz, CF_3_), 124.3 (CH), 124.2 (CH), 121.3 (CH), 119.7 (CH), 117.8 (CH), 116.2 (CH), 114.7 (CH*), 114.4 (CH**), 78.4 (q, *J* = 28.2 Hz, C), 77.9 (q, *J* = 27.1 Hz, C), 67.0 (CH), 66.8 (CH), 58.1 (CH_2_), 56.23 (q, *J* = 1.6 Hz, CH_2_), 29.0 (CH_3_), 28.9 (CH_3_); HRMS (ESI/Q-TOF) *m*/*z* [M + H]^+^ C_24_H_22_F_3_N_2_O_2_^+^ Calcd for 427.1628; Found 427.1629.

### 4-Benzyl-7-methyl-3-(2,2,2-trifluoro-1-hydroxy-1-phenylethyl)-3,4-dihydroquinoxalin-2(1*H*)-one (3ha)

Using 4-benzyl-7-methyl-3,4-dihydroquinoxalin-2(1*H*)-one (**1h**, 65.6 mg, 0.26 mmol, 1.3 equiv) and 2,2,2-trifluoroacetophenone (**2a**, 28.1 μL, 0.2 mmol, 1 equiv), according to GP-1, compound **3ha** was obtained as a mixture of diastereoisomers (51:49 dr) that were separated by column chromatography using hexane:EtOAc mixtures (from 9:1 to 7:3): **3ha′** (25.7 mg, 0.06 mmol, 30% yield, colorless oil) and **3ha″** (24.7 mg, 0.06 mmol, 29% yield, colorless oil).

#### Characterization of **3ha′**

^1^H NMR (300 MHz, CDCl_3_) δ 9.25 (s, 1H), 7.61 (dd, *J* = 6.8, 3.0 Hz, 2H), 7.41–7.34 (m, 3H), 7.24–7.18 (m, 3H), 7.00–6.91 (m, 2H), 6.87–6.79 (m, 2H), 6.42 (d, *J* = 1.6 Hz, 1H), 4.81 (s, 1H), 4.53 (d, *J* = 15.6 Hz, 1H), 4.33 (d, *J* = 0.9 Hz, 1H), 3.48 (d, *J* = 15.6 Hz, 1H), 2.24 (s, 3H); ^19^F{^1^H} NMR (282 MHz, CDCl_3_) δ −73.34; ^13^C{^1^H} NMR (75 MHz, CDCl_3_) δ 165.6 (C), 136.4 (C), 134.8 (C), 130.9 (C), 130.5 (C), 128.8 (CH), 128.7 (CH), 128.2 (CH), 127.8 (CH), 127.5 (CH), 126.7 (C), 126.5 (d, *J* = 1.7 Hz, CH), 125.4 (CH), 117.4 (CH), 116.5 (CH), 79.2 (q, *J* = 27.8 Hz, C), 67.1 (CH), 57.9 (CH_2_), 20.6 (CH_3_); HRMS (ESI/Q-TOF) *m*/*z* [M + H]^+^ C_24_H_22_F_3_N_2_O_2_^+^ Calcd for 427.1628; Found 427.1633.

#### Characterization of **3ha″**

^1^H NMR (300 MHz, CDCl_3_) δ 8.83 (s, 1H), 7.40 (d, *J* = 7.6 Hz, 2H), 7.20–7.07 (m, 4H), 7.07–7.02 (m, 2H), 7.02–6.93 (m, 2H), 6.67–6.56 (m, 2H), 6.12 (s, 1H), 4.72–4.60 (m, 2H), 4.53 (s, 1H), 4.08 (d, *J* = 15.9 Hz, 1H), 2.06 (s, 3H); ^19^F{^1^H} NMR (282 MHz, CDCl_3_) δ −74.21; ^13^C{^1^H} NMR (75 MHz, CDCl_3_) δ 165.0 (C), 136.8 (C), 135.5 (C), 134.4 (C), 131.1 (C), 128.7 (CH), 128.7 (CH), 127.7 (CH), 127.7 (CH), 127.4 (CH), 126.9 (q, *J* = 2.3 Hz, CH), 125.8 (C), 125.3 (CH), 116.7 (CH), 116.1 (CH), 78.6 (q, *J* = 27 Hz, C), 66.5 (CH), 57.0 (CH_2_), 20.4 (CH_3_); HRMS (ESI/Q-TOF) *m*/*z* [M + H]^+^ C_24_H_22_F_3_N_2_O_2_^+^ Calcd for 427.1628; Found 427.1619.

### 4-Benzyl-7-bromo-3-(2,2,2-trifluoro-1-hydroxy-1-phenylethyl)-3,4-dihydroquinoxalin-2(1*H*)-one (3ia)

Using 4-benzyl-7-bromo-3,4-dihydroquinoxalin-2(1*H*)-one (**1i**, 82.5 mg, 0.26 mmol, 1.3 equiv) and 2,2,2-trifluoroacetophenone (**2a**, 28.1 μL, 0.2 mmol, 1 equiv), according to GP-1, compound **3ia** was obtained as a mixture of diastereoisomers (50:50 dr) that cannot be separated by column chromatography using hexane:EtOAc mixtures (from 9:1 to 7:3): **3ia′** + **3ai″** (66.8 mg, 0.136 mmol, 68% yield, colorless oil). Representative NMR signals for either the major and the minor diastereoisomer are labeled with one or two asterisks, respectively.

^1^H NMR (300 MHz, CDCl_3_) δ 9.73 (s, 1H**), 9.23 (s, 1H*), 7.57–7.45 (m, 2H**), 7.37 (d, *J* = 7.5 Hz, 2H*), 7.33–7.24 (m, 1H*+2H**), 7.16–7.05 (m, 8H), 7.00 (dd, *J* = 8.6, 2.1 Hz, 1H**), 6.97–6.83 (m, 6H), 6.72–6.61 (m, 2H**), 6.50 (d, *J* = 8.6 Hz, 1H*), 6.38 (d, *J* = 2.2 Hz, 1H*), 4.63–4.53 (m, 2H*), 4.48 (d, *J* = 15.8 Hz, 1H**) 4.32–4.35 (m, 1H*+1H**), 4.25 (s, 1H**), 4.03 (d, *J* = 15.9 Hz, 1H*), 3.53 (d, *J* = 15.8 Hz, 1H**); ^19^F{^1^H} NMR (282 MHz, CDCl_3_) δ −73.17**, −74.01*; ^13^C{^1^H} NMR (75 MHz, CDCl_3_) δ 165.1 (C), 164.4 (C), 136.1 (C), 135.9 (C), 134.4 (C), 134.0 (C), 132.7 (C), 132.3 (C), 129.2 (CH), 129.0 (CH), 128.9 (CH), 128.4 (CH), 128.0 (CH), 128.0 (CH), 127.9 (CH), 127.8 (C), 127.3 (CH), 127.2 (CH), 127.1 (C), 126.7 (q, *J* = 1.8 Hz, CH), 126.3 (q, *J* = 1.7 Hz, CH), 118.6 (CH), 118.2 (CH), 117.7 (CH), 117.5 (CH), 112.3 (C), 111.5 (C), 79.8 (q, *J* = 27.9 Hz, C), 78.9 (q, *J* = 27.4 Hz, C), 67.2 (CH), 66.5 (CH), 57.0 (CH_2_), 56.6 (CH_2_); HRMS (ESI/Q-TOF) *m*/*z* [M + H]^+^ C_23_H_19_BrF_3_N_2_O_2_^+^ Calcd for 491.0577; Found 491.0582.

### 3-(2,2,2-Trifluoro-1-hydroxy-1-phenylethyl)-3,4-dihydroquinoxalin-2(1*H*)-one (3la)

Using 3,4-dihydroquinoxalin-2(1*H*)-one (**1l**, 38.5 mg, 0.26 mmol, 1.3 equiv) and 2,2,2-trifluoroacetophenone (**2a**, 28.1 μL, 0.2 mmol, 1 equiv), according to GP-1, compound **3la** was obtained as a mixture of diastereoisomers (50:50 dr) that cannot be separated by column chromatography using hexane:EtOAc mixtures (from 9:1 to 7:3): **3la′** + **3la″** (26.4 mg, 0.082 mmol, 41% yield, colorless oil).

^1^H NMR (300 MHz, DMSO-*d*^*6*^) δ 10.43 (s, 1H), 10.31 (s, 1H), 7.60–7.50 (m, 2H), 7.49–7.41 (m, 2H), 7.35–7.26 (m, 3H), 7.25–7.16 (m, 3H), 6.82 (s, 1H), 6.74–6.66 (m, 1H), 6.66–6.60 (m, 3H), 6.56 (d, *J* = 7.1 Hz, 1H), 6.51 (dd, *J* = 7.8, 1.4 Hz, 1H), 6.49–6.35 (m, 3H), 6.16 (d, *J* = 2.6 Hz, 1H), 5.87 (d, *J* = 3.3 Hz, 1H), 4.56 (d, *J* = 3.3 Hz, 1H), 4.46 (d, *J* = 2.5 Hz, 1H); ^19^F{^1^H} NMR (282 MHz, DMSO-*d*^*6*^) δ −72.18, −72.44; ^13^C NMR (75 MHz, DMSO-*d*^*6*^) δ 162.7 (C), 162.1 (C), 136.1 (C), 135.7 (C), 133.0 (C), 132.6 (C), 128.3 (CH), 128.0 (CH), 127.8 (CH), 127.4 (CH), 126.7 (CH), 126.4 (CH), 125.4 (C), 124.8 (C), 122.8 (CH), 122.4 (CH), 117.1 (CH), 117.0 (CH), 114.3 (CH), 114.0 (CH), 112.9 (CH), 112.9 (CH), 79.4 (q, *J*_C–F_ = 26.0 Hz, C), 79.3 (q, *J*_C–F_ = 25.7 Hz, C), 60.9 (CH), 60.3 (CH); HRMS (ESI/Q-TOF) *m*/*z* [M + H]^+^ C_16_H_14_F_3_N_2_O_2_^+^ Calcd for 323.1002; Found 323.1004.

### 4-Benzyl-3-(2,2,2-trifluoro-1-hydroxy-1-(*p*-tolyl)ethyl)-3,4-dihydroquinoxalin-2(1*H*)-one (3ab)

Using 4-benzyl-3,4-dihydroquinoxalin-2(1*H*)-one (**1a**, 62 mg, 0.26 mmol, 1.3 equiv) and 2,2,2-trifluoro-1-(*p*-tolyl)ethan-1-one (**2b**, 31 μL, 0.2 mmol, 1 equiv), according to GP-1, compound **3ab** was obtained as a mixture of diastereoisomers (60:40 dr) that were separated by column chromatography using hexane:EtOAc mixtures (from 95:5 to 75:25): **3ab′** (28.0 mg, 0.06 mmol, 30% yield, brown oil) and **3ab″** (18.7 mg, 0.04 mmol, 20% yield, brown oil).

#### Characterization of **3ab′**

^1^H NMR (300 MHz, CDCl_3_) δ 9.21 (s, 1H), 7.50 (d, *J* = 8.3 Hz, 2H), 7.24–7.15 (m, 5H), 7.06–6.86 (m, 4H), 6.81 (td, *J* = 7.6, 1.4 Hz, 1H), 6.62 (dd, *J* = 7.8, 1.3 Hz, 1H), 4.65 (s, 1H), 4.59 (d, *J* = 15.8 Hz, 1H), 4.35 (s, 1H), 3.50 (d, *J* = 15.8 Hz, 1H), 2.38 (s, 3H). ^19^F{^1^H} NMR (282 MHz, CDCl_3_) δ −73.34. ^13^C{^1^H} NMR (75 MHz, CDCl_3_) δ 165.5 (C), 138.8 (C), 136.4 (C), 133.2 (C), 131.7 (C), 129.0 (CH), 128.7 (CH), 127.8 (CH), 127.4 (CH), 126.5 (C), 126.4 (q, *J* = 1.8 Hz, CH), 125.2 (d, *J* = 286.9 Hz, CF_3_), 124.7 (CH), 120.7 (CH), 116.7 (CH), 115.8 (CH), 79.4 (q, *J* = 27.9 Hz, C), 67.3 (CH), 57.3 (CH_2_), 21.1 (CH_3_); HRMS (ESI/Q-TOF) *m*/*z* [M + H]^+^ C_24_H_22_F_3_N_2_O_2_^+^ Calcd for 427.1628; Found 427.1621.

#### Characterization of **3ab″**

^1^H NMR (300 MHz, CDCl_3_) δ 8.85 (s, 1H), 7.34 (d, *J* = 8.2 Hz, 2H), 7.24–7.16 (m, 3H), 7.06 (dd, *J* = 7.2, 2.2 Hz, 2H), 6.97–6.85 (m, 3H), 6.78 (d, *J* = 7.8 Hz, 1H), 6.65 (td, *J* = 7.6, 1.2 Hz, 1H), 6.39 (dd, *J* = 7.7, 1.3 Hz, 1H), 4.79 (d, *J* = 16.0 Hz, 1H), 4.63 (s, 1H), 4.59 (s, 1H), 4.19 (d, *J* = 16.0 Hz, 1H), 2.22 (s, 3H). ^13^C{^1^H} NMR (282 MHz, CDCl_3_) δ −74.38. ^13^C{^1^H} NMR (75 MHz, CDCl_3_) δ 164.8 (C), 138.6 (C), 136.7 (C), 133.6 (C), 131.2 (C), 128.8 (CH), 128.4 (CH), 127.7 (CH), 127.3 (CH), 126.8 (d, *J* = 1.8 Hz, CH), 125.8 (C), 124.6 (CH), 119.8 (CH), 116.4 (CH), 115.5 (CH), 78.6 (q, *J* = 27.1, 26.5 Hz, C), 66.4 (CH), 56.5 (CH_2_), 20.9 (CH_3_); HRMS (ESI/Q-TOF) *m*/*z* [M + H]^+^ C_24_H_22_F_3_N_2_O_2_^+^ Calcd for 427.1628; Found 427.1624.

### 4-Benzyl-3-(1-(4-ethylphenyl)-2,2,2-trifluoro-1-hydroxyethyl)-3,4-dihydroquinoxalin-2(1*H*)-one (3ac)

Using 4-benzyl-3,4-dihydroquinoxalin-2(1*H*)-one (**1a**, 62 mg, 0.26 mmol, 1.3 equiv) and 1-(4-ethylphenyl)-2,2,2-trifluoroethan-1-one (**2c**, 33 μL, 0.2 mmol, 1 equiv), according to GP-1, compound **3ac** was obtained as a mixture of diastereoisomers (53:47 dr) that were separated by column chromatography using hexane:EtOAc mixtures (from 95:5 to 75:25): **3ac′** (13.6 mg, 0.03 mmol, 15% yield, yellow oil) and **3ac″** (12.1 mg, 0.03 mmol, 14% yield, yellow oil).

#### Characterization of **3ac′**

^1^H NMR (300 MHz, CDCl_3_) δ 8.80 (s, 1H), 7.51 (d, *J* = 8.2 Hz, 2H), 7.24–7.17 (m, 5H), 6.99 (ddd, *J* = 8.6, 7.3, 1.4 Hz, 1H), 6.95–6.86 (m, 3H), 6.80 (td, *J* = 7.5, 1.4 Hz, 1H), 6.62 (dd, *J* = 7.8, 1.4 Hz, 1H), 4.69 (s, 1H), 4.56 (d, *J* = 15.7 Hz, 1H), 4.33 (s, 1H), 3.45 (d, *J* = 15.8 Hz, 1H), 2.68 (q, *J* = 7.6 Hz, 2H), 1.25 (t, *J* = 7.6 Hz, 3H). ^13^C{^1^H} NMR (282 MHz, CDCl_3_) δ −73.29. ^13^C{^1^H} NMR (101 MHz, CDCl_3_) δ 165.4 (C), 145.1 (C), 136.4 (C), 133.2 (C), 132.0 (C), 128.8 (CH), 127.81 (CH), 127.77(CH), 127.4 (CH), 126.5 (CH), 124.7 (CH), 120.6 (CH), 116.9 (CH), 115.7 (CH), 79.4 (d, *J* = 26.3 Hz, C), 67.3 (CH), 57.3 (CH_2_), 28.5 (CH_2_), 15.4 (CH_3_); HRMS (ESI/Q-TOF) *m*/*z* [M + H]^+^ C_25_H_24_F_3_N_2_O_2_^+^ Calcd for 441.1784; Found 441.1791.

#### Characterization of **3ac″**

^1^H NMR (300 MHz, CDCl_3_) δ 8.32 (s, 1H), 7.35 (d, *J* = 8.1 Hz, 2H), 7.24–7.21 (m, 3H), 7.06 (dd, *J* = 7.4, 2.1 Hz, 2H), 6.93–6.86 (m, 3H), 6.76 (d, *J* = 8.1 Hz, 1H), 6.61 (td, *J* = 7.6, 1.3 Hz, 1H), 6.34 (dd, *J* = 7.8, 1.9 Hz, 1H), 4.80 (d, *J* = 16.0 Hz, 1H), 4.62 (s, 1H), 4.58 (s, 1H), 4.21 (d, *J* = 15.9 Hz, 1H), 2.51 (q, *J* = 7.6 Hz, 2H), 1.13 (t, *J* = 7.6 Hz, 3H). ^13^C{^1^H} NMR (282 MHz, CDCl_3_) δ −74.38. ^13^C{^1^H} NMR (101 MHz, CDCl_3_) δ 164.7 (C), 144.9 (C), 136.7 (C), 133.6 (C), 131.2 (C), 128.8 (CH), 127.7 (CH), 127.3 (CH), 127.2 (CH), 126.9 (q, *J* = 2.5 Hz, CH), 125.8 (C), 124.6 (CH), 119.8 (CH), 116.4 (CH), 115.4 (CH), 78.5 (q, *J* = 27.1 Hz, C), 66.5 (CH), 56.5 (CH_2_), 28.3 (CH_2_), 15.4 (CH_3_); HRMS (ESI/Q-TOF) *m*/*z* [M + H]^+^ C_25_H_24_F_3_N_2_O_2_^+^ Calcd for 441.1784; Found 441.1793.

### 4-Benzyl-3-(2,2,2-trifluoro-1-hydroxy-1-(4-methoxyphenyl)ethyl)-3,4-dihydroquinoxalin-2(1*H*)-one (3ad)

Using 4-benzyl-3,4-dihydroquinoxalin-2(1*H*)-one (**1a**, 62 mg, 0.26 mmol, 1.3 equiv) and 2,2,2-trifluoro-1-(4-methoxyphenyl)ethan-1-one (**2d**, 31 μL, 0.2 mmol, 1 equiv), according to GP-1, compound **3ad** was obtained as a mixture of diastereoisomers (54:46 dr) that were separated by column chromatography using hexane:EtOAc mixtures (from 95:5 to 75:25): **3ad′** (22.7 mg, 0.05 mmol, 27% yield, yellow oil) and **3ad″** (20.2 mg, 0.05 mmol, 23% yield, yellow oil).

#### Characterization of **3ad′**

^1^H NMR (300 MHz, CDCl_3_) δ 8.92 (s, 1H), 7.52 (d, *J* = 8.8 Hz, 2H), 7.23–7.18 (m, 3H), 7.04–6.89 (m, 6H), 6.81 (td, *J* = 7.5, 1.4 Hz, 1H), 6.63 (dd, *J* = 7.8, 1.4 Hz, 1H), 4.68 (s, 1H), 4.59 (d, *J* = 15.8 Hz, 1H), 4.32 (s, 1H), 3.81 (s, 3H), 3.52 (d, *J* = 15.8 Hz, 1H). ^13^C{^1^H} NMR (282 MHz, CDCl_3_) δ −73.56. ^13^C{^1^H} NMR (75 MHz, CDCl_3_) δ 165.4 (C), 160.0 (C), 136.4 (C), 133.1 (C), 128.8 (CH), 127.9 (CH), 127.8 (CH), 127.4 (CH), 126.7 (C), 126.5 (C), 125.2 (d, *J* = 287.5 Hz, CF_3_), 124.7 (CH), 120.7 (CH), 116.9 (CH), 115.8 (CH), 113.6 (CH), 79.19 (d, *J* = 27.6 Hz, C), 67.3 (CH), 57.4 (CH_2_), 55.2 (CH_3_); HRMS (ESI/Q-TOF) *m*/*z* [M + H]^+^ C_24_H_22_F_3_N_2_O_3_^+^ Calcd for 443.1577; Found 443.1574.

#### Characterization of **3ad″**

^1^H NMR (300 MHz, CDCl_3_) δ 8.50 (s, 1H), 7.37 (d, *J* = 8.8 Hz, 2H), 7.24–7.19 (m, 3H), 7.07 (dd, *J* = 7.3, 2.2 Hz, 2H), 6.92 (ddd, *J* = 8.1, 7.3, 1.4 Hz, 1H), 6.80 (d, *J* = 8.6 Hz, 1H), 6.70–6.57 (m, 3H), 6.38 (dd, *J* = 7.8, 1.4 Hz, 1H), 4.83 (d, *J* = 16.0 Hz, 1H), 4.66 (s, 1H), 4.62 (s, 1H), 4.24 (d, *J* = 16.0 Hz, 1H), 3.70 (s, 3H). ^13^C{^1^H} NMR (282 MHz, CDCl_3_) δ −74.63. ^13^C{^1^H} NMR (75 MHz, CDCl_3_) δ 164.9 (C), 159.8 (C), 136.7 (C), 133.6 (C), 130.9 (d, *J* = 283.6 Hz, CF_3_), 128.8 (CH), 128.3 (d, *J* = 2.2 Hz, CH), 127.8 (CH), 127.3 (CH), 125.9 (C), 125.7 (C), 124.7 (CH), 119.9 (CH), 116.4 (CH), 115.4 (CH), 113.0 (CH), 78.2 (d, *J* = 27.6 Hz, C), 66.5 (CH), 56.5 (CH_2_), 55.2 (CH_3_); HRMS (ESI/Q-TOF) *m*/*z* [M + H]^+^ C_24_H_22_F_3_N_2_O_3_^+^ Calcd for 443.1577; Found 443.1582.

### 4-Benzyl-3-(1-(4-chlorophenyl)-2,2,2-trifluoro-1-hydroxyethyl)-3,4-dihydroquinoxalin-2(1*H*)-one (3ae)

Using 4-benzyl-3,4-dihydroquinoxalin-2(1*H*)-one (**1a**, 62 mg, 0.26 mmol, 1.3 equiv) and 2,2,2-trifluoro-1-(4-chlorophenyl)ethan-1-one (**2e**, 30 μL, 0.2 mmol, 1 equiv), according to GP-1, compound **3ae** was obtained as a mixture of diastereoisomers (50:50 dr) that were separated by column chromatography using hexane:EtOAc mixtures (from 95:5 to 75:25): **3ae′** (24.1 mg, 0.05 mmol, 27% yield, yellow oil) and **3ae″** (24.4 mg, 0.05 mmol, 27% yield, yellow oil).

#### Characterization of **3ae′**

^1^H NMR (300 MHz, CDCl_3_) δ 8.90 (s, 1H), 7.53 (d, *J* = 8.7 Hz, 2H), 7.34 (d, *J* = 8.9 Hz, 2H)., 7.24–7.21 (M, 3H), 7.04–6.91 (m, 4H), 6.84 (ddd, *J* = 7.8, 7.1, 1.6 Hz, 1H), 6.57 (dd, *J* = 7.8, 1.4 Hz, 1H), 4.65 (s, 1H), 4.60 (d, *J* = 15.5 Hz, 1H), 4.32 (s, 1H), 3.60 (d, *J* = 15.6 Hz, 1H). ^13^C{^1^H} NMR (282 MHz, CDCl3) δ −73.70. ^13^C{^1^H} NMR (75 MHz, CDCl_3_) δ 165.0 (C), 136.0 (C), 135.1 (C), 133.2 (C), 132.8 (C), 128.9 (CH), 128.4 (CH), 128.0 (CH), 127.5 (CH), 126.8 (C), 124.9 (CH), 124.6 (q, *J* = 283.3 Hz, CF3), 121.4 (CH), 117.6 (CH), 115.9 (CH), 79.0 (d, *J* = 28.2 Hz, C), 66.9 (CH), 58.1 (CH_2_); HRMS (ESI/Q-TOF) *m*/*z* [M + H]^+^ C_23_H_19_ClF_3_N_2_O_2_^+^ Calcd for 447.1082; Found 447.1088.

#### Characterization of **3ae″**

^1^H NMR (300 MHz, CDCl_3_) δ 8.41 (s, 1H), 7.39 (d, *J* = 8.7 Hz, 2H), 7.25 7.20 (m, 3H), 7.10–7.07 (m, 2H), 7.02 (d, *J* = 8.9 Hz, 2H), 6.94 (ddd, *J* = 8.1, 7.4, 1.4 Hz, 1H), 6.83 (d, *J* = 7.7 Hz, 1H), 6.68 (td, *J* = 7.7, 1.3 Hz, 1H), 6.35 (dd, *J* = 7.8, 1.4 Hz, 1H), 4.96–4.81 (m, 1H), 4.63 (s, 1H), 4.31 (d, *J* = 15.8 Hz, 1H). ^13^C{^1^H} NMR (282 MHz, CDCl_3_) δ −74.60. ^13^C{^1^H} NMR (75 MHz, CDCl_3_) δ 164.7 (C), 136.4 (C), 135.0 (C), 133.3 (C), 132.6 (C), 128.9 (CH), 128.6 (d, *J* = 2.2 Hz, CH), 127.9 (CH), 127.7 (CH), 127.4 (CH), 125.5 (C), 125.0 (CH), 124.7 (q, *J* = 286.4 Hz, CF_3_), 120.3 (CH), 116.9 (CH), 115.5 (CH), 77.8 (d, *J* = 27.6 Hz, C), 66.3 (CH), 56.9 (CH_2_); HRMS (ESI/Q-TOF) *m*/*z* [M + H]^+^ C_23_H_19_ClF_3_N_2_O_2_^+^ Calcd for 447.1082; Found 447.1085.

### 4-Benzyl-3-(1-(4-bromophenyl)-2,2,2-trifluoro-1-hydroxyethyl)-3,4-dihydroquinoxalin-2(1*H*)-one (3af)

Using 4-benzyl-3,4-dihydroquinoxalin-2(1*H*)-one (**1a**, 62 mg, 0.26 mmol, 1.3 equiv) and 1-(4-bromophenyl)-2,2,2-trifluoroethan-1-one (**2f**, 30 μL, 0.2 mmol, 1 equiv), according to GP-1, compound **3af** was obtained as a mixture of diastereoisomers (58:42 dr) that were separated by column chromatography using hexane:EtOAc mixtures (from 95:5 to 75:25): **3af′** (36.3 mg, 0.08 mmol, 37% yield, yellow oil) and **3af″** (26.4 mg, 0.05 mmol, 27% yield, yellow oil).

#### Characterization of **3af′**

^1^H NMR (300 MHz, CDCl_3_) δ 9.10 (s, 1H), 7.63–7.38 (m, 4H), 7.24–7.21 (m, 3H), 7.07–6.92 (m, 4H), 6.87 (td, *J* = 7.5, 1.6 Hz, 1H), 6.58 (dd, *J* = 7.8, 1.4 Hz, 1H), 4.66 (s, 1H), 4.63 (d, *J* = 15.4 Hz, 1H), 4.34 (s, 1H), 3.63 (d, *J* = 15.5 Hz, 1H). ^13^C{^1^H} NMR (282 MHz, CDCl_3_) δ −73.68. ^13^C{^1^H} NMR (75 MHz, CDCl_3_) δ 165.0 (C), 136.0 (C), 133.7 (C), 132.8 (C), 131.3 (CH), 128.9 (CH), 128.3 (q, *J* = 1.8 Hz, CH), 128.0 (CH), 127.5 (CH), 126.9 (C), 124.9 (d, *J* = 286.9 Hz, CF_3_), 124.86 (CH), 123.4 (C), 121.5 (CH), 117.6 (CH), 115.9 (CH), 79.1 (q, *J* = 28.2 Hz, C), 66.8 (CH), 58.1 (CH_2_); HRMS (ESI/Q-TOF) *m*/*z* [M + H]^+^ C_23_H_19_BrF_3_N_2_O_2_^+^ Calcd for 491.0577; Found 491.0570.

#### Characterization of **3af″**

^1^H NMR (500 MHz, CDCl_3_) δ 7.55 (s, 1H), 7.32 (d, *J* = 8.7 Hz, 2H), 7.27–7.21 (m, 3H), 7.17 (d, *J* = 8.7 Hz, 2H), 7.08 (d, *J* = 8.1 Hz, 2H), 6.94 (t, *J* = 7.8 Hz, 1H), 6.84 (d, *J* = 8.0 Hz, 1H), 6.69 (t, *J* = 7.6 Hz, 1H), 6.32 (d, *J* = 7.8 Hz, 1H), 4.90 (d, *J* = 16.0 Hz, 1H), 4.85 (s, 1H), 4.63 (s, 1H), 4.34 (d, *J* = 15.9 Hz, 1H). ^13^C{^1^H} NMR (282 MHz, CDCl_3_) δ −74.60. ^13^C{^1^H} NMR (75 MHz, CDCl_3_) δ 164.6 (C), 136.4 (C), 133.3 (C), 133.1 (C), 130.7 (CH), 128.9 (CH), 127.9 (CH), 127.4 (CH), 125.5 (C), 125.0 (CH), 123.4 (C), 120.4 (CH), 117.0 (CH), 115.5 (CH), 77.8 (d, *J* = 27.1 Hz), 66.3 (CH), 57.0 (CH_2_); HRMS (ESI/Q-TOF) *m*/*z* [M + H]^+^ C_23_H_19_BrF_3_N_2_O_2_^+^ Calcd for 491.0577; Found 491.0572.

### 4-Benzyl-3-(2,2,2-trifluoro-1-hydroxy-1-(*m*-tolyl)ethyl)-3,4-dihydroquinoxalin-2(1*H*)-one (3ag)

Using 4-benzyl-3,4-dihydroquinoxalin-2(1*H*)-one (**1a**, 62 mg, 0.26 mmol, 1.3 equiv) and 2,2,2-trifluoro-1-(*m*-tolyl)ethan-1-one (**2g**, 31 μL, 0.2 mmol, 1 equiv), according to GP-1, compound **3ag** was obtained as a mixture of diastereoisomers (58:42 dr) that were separated by column chromatography using hexane:EtOAc mixtures (from 95:5 to 75:25): **3ag′** (24.8 mg, 0.06 mmol, 31% yield, yellow oil) and **3ag″** (19.6 mg, 0.05 mmol, 24% yield, yellow oil).

#### Characterization of **3ag′**

^1^H NMR (300 MHz, CDCl_3_) δ 8.89 (s, 1H), 7.40 (d, *J* = 10.2 Hz, 2H), 7.32–7.25 (m, 1H), 7.26–7.15 (m, 4H), 7.00 (td, *J* = 7.8, 7.4, 1.5 Hz, 1H), 6.96–6.87 (m, 3H), 6.81 (td, *J* = 7.6, 1.3 Hz, 1H), 6.62 (dd, *J* = 7.8, 1.1 Hz, 1H), 4.73 (s, 1H), 4.56 (d, *J* = 15.7 Hz, 1H), 4.34 (s, 1H), 3.45 (d, *J* = 15.8 Hz, 1H), 2.36 (s, 3H). ^13^C{^1^H} NMR (282 MHz, CDCl_3_) δ −73.13. ^13^C{^1^H} NMR (75 MHz, CDCl_3_) δ 165.5 (C), 138.0 (C), 136.3 (C), 134.7 (C), 133.2 (C), 129.7 (CH), 128.8 (CH), 128.1 (CH), 127.8 (CH), 127.4 (CH), 127.2 (q, *J* = 1.8 Hz, CH), 126.5 (C), 124.7 (CH), 123.6 (q, *J* = 2.8 Hz, CH), 120.7 (CH), 116.8 (CH), 115.8 (CH), 79.4 (q, *J* = 27.9 Hz, C), 67.3 (CH), 57.3 (CH_2_), 21.6 (CH_3_); HRMS (ESI/Q-TOF) *m*/*z* [M + H]^+^ C_24_H_22_F_3_N_2_O_2_^+^ Calcd for 427.1628; Found 427.1633.

#### Characterization of **3ag″**

^1^H NMR (300 MHz, CDCl_3_) δ 8.32 (s, 1H), 7.41–7.17 (m, 5H), 7.14–7.04 (m, 2H), 7.03–6.89 (m, 3H), 6.83 (d, *J* = 7.8 Hz, 1H), 6.64 (td, *J* = 7.6, 1.3 Hz, 1H), 6.37 (dd, *J* = 7.8, 1.4 Hz, 1H), 4.85 (d, *J* = 16.0 Hz, 1H), 4.72 (s, 1H), 4.65 (s, 1H), 4.25 (d, *J* = 16.0 Hz, 1H), 2.08 (s, 3H). ^13^C{^1^H} NMR (282 MHz, CDCl_3_) δ −74.36. ^13^C{^1^H} NMR (75 MHz, CDCl_3_) δ 164.8 (C), 137.5 (C), 136.6 (C), 134.0 (C), 133.7 (C), 129.5 (CH), 128.8 (CH), 127.8 (CH), 127.6 (CH), 127.3 (CH), 126.1 (q, *J* = 281.4 Hz, CF_3_), 125.6 (C), 124.7 (CH), 123.9 (q, *J* = 1.8 Hz, CH), 119.9 (CH), 116.3 (CH), 115.3 (CH), 78.5 (q, *J* = 27.6 Hz, C), 66.5 (CH), 56.4 (q, *J* = 1.7 Hz, CH_2_), 21.3 (CH_3_); HRMS (ESI/Q-TOF) *m*/*z* [M + H]^+^ C_24_H_22_F_3_N_2_O_2_^+^ Calcd for 427.1628; Found 427.1621.

### 4-Benzyl-3-(2,2,2-trifluoro-1-hydroxy-1-(3-methoxyphenyl)ethyl)-3,4-dihydroquinoxalin-2(1*H*)-one (3ah)

Using 4-benzyl-3,4-dihydroquinoxalin-2(1*H*)-one (**1a**, 62 mg, 0.26 mmol, 1.3 equiv) and 2,2,2-trifluoro-1-(3-methoxyphenyl)ethan-1-one (**2h**, 32 μL, 0.2 mmol, 1 equiv), according to GP-1, compound **3ah** was obtained as a mixture of diastereoisomers (53:47 dr) that were separated by column chromatography using hexane:EtOAc mixtures (from 95:5 to 75:25): **3ah′** (23.1 mg, 0.05 mmol, 26% yield, yellow oil) and **3ah″** (20.6 mg, 0.05 mmol, 24% yield, yellow oil).

#### Characterization of **3ah′**

^1^H NMR (300 MHz, CDCl_3_) δ 9.26 (s, 1H), 7.31 (t, *J* = 8.2 Hz, 1H), 7.23–7.17 (m, 5H), 7.10–6.87 (m, 5H), 6.81 (td, *J* = 7.5, 1.4 Hz, 1H), 6.64 (dd, *J* = 7.8, 1.4 Hz, 1H), 4.83 (s, 1H), 4.59 (d, *J* = 15.7 Hz, 1H), 4.35 (s, 1H), 3.78 (s, 3H), 3.49 (d, *J* = 15.7 Hz, 1H). ^13^C{^1^H} NMR (282 MHz, CDCl_3_) δ −73.15. ^13^C{^1^H}NMR (75 MHz, CDCl_3_) δ 165.6 (C), 159.6 (C), 136.3 (C), 133.1 (C), 129.3 (CH), 128.8 (CH), 127.9 (CH), 127.4 (CH), 126.5 (C), 125.1 (q, *J* = 287.5 Hz, CF_3_), 124.8 (CH), 120.8 (CH), 118.9 (q, *J* = 2.2 Hz, CH), 116.9 (CH), 115.9 (CH), 114.6 (CH), 112.2 (q, *J* = 2.0 Hz, CH), 79.3 (q, *J* = 27.6 Hz, C), 67.2 (CH), 57.4 (CH_2_), 55.2 (CH_3_); HRMS (ESI/Q-TOF) *m*/*z* [M + H]^+^ C_24_H_22_F_3_N_2_O_3_^+^ Calcd for 443.1577; Found 443.1579.

#### Characterization of **3ah″**

^1^H NMR (300 MHz, CDCl_3_) δ 8.97 (s, 1H), 7.25–7.15 (m, 3H), 7.09–7.00 (m, 5H), 6.92 (ddd, *J* = 8.7, 7.3, 1.5 Hz, 1H), 6.80 (dd, *J* = 8.1, 1.3 Hz, 1H), 6.74 (ddd, *J* = 7.7, 2.5, 1.4 Hz, 1H), 6.65 (td, *J* = 7.6, 1.3 Hz, 1H), 6.44 (dd, *J* = 7.8, 1.4 Hz, 1H), 4.81 (d, *J* = 16.0 Hz, 1H), 4.74 (s, 1H), 4.66 (s, 1H), 4.19 (d, *J* = 16.0 Hz, 1H), 3.60 (s, 3H). ^13^C{^1^H} NMR (282 MHz, CDCl_3_) δ −74.18. ^13^C{^1^H} NMR (75 MHz, CDCl_3_) δ 164.7 (C), 159.1 (C), 136.6 (C), 135.9 (C), 133.7 (C), 128.8 (CH), 128.8 (CH), 127.8 (CH), 127.3 (CH), 125.8 (C), 124.7 (CH), 119.9 (CH), 119.3 (q, *J* = 1.7 Hz, CH), 116.2 (CH), 115.5 (CH), 114.8 (CH), 112.5 (q, *J* = 1.7 Hz, CH), 78.7 (q, *J* = 27.6 Hz, C), 66.4 (CH), 56.4 (CH_2_), 55.0 (CH_3_); HRMS (ESI/Q-TOF) *m*/*z* [M + H]^+^ C_24_H_22_F_3_N_2_O_3_^+^ Calcd for 443.1577; Found 443.1583.

### 4-Benzyl-3-(1-(3-chlorophenyl)-2,2,2-trifluoro-1-hydroxyethyl)-3,4-dihydroquinoxalin-2(1*H*)-one (3ai)

Using 4-benzyl-3,4-dihydroquinoxalin-2(1*H*)-one (**1a**, 62 mg, 0.26 mmol, 1.3 equiv) and 1-(3-chlorophenyl)-2,2,2-trifluoroethan-1-one (**2i**, 29 μL, 0.2 mmol, 1 equiv), according to GP-1, compound **3ai** was obtained as a mixture of diastereoisomers (55:45 dr) that were separated by column chromatography using hexane:EtOAc mixtures (from 95:5 to 75:25): **3ai′** (30.0 mg, 0.07 mmol, 33% yield, yellow oil) and **3ai″** (24.6 mg, 0.05 mmol, 28% yield, yellow oil).

#### Characterization of **3ai′**

^1^H NMR (300 MHz, CDCl_3_) δ 8.38 (s, 1H), 7.48 (t, *J* = 2.0 Hz, 1H), 7.37 (d, *J* = 7.9 Hz, 1H), 7.26–7.20 (m, 3H), 7.17–7.05 (m, 3H), 7.01 (d, *J* = 8.0 Hz, 1H), 6.98–6.91 (m, 1H), 6.86 (d, *J* = 7.3 Hz, 1H), 6.66 (td, *J* = 7.7, 1.4 Hz, 1H), 6.37 (dd, *J* = 7.8, 1.3 Hz, 1H), 4.94 (s, 1H), 4.90 (d, *J* = 16.0 Hz, 1H), 4.64 (s, 1H), 4.31 (d, *J* = 16.0 Hz, 1H); ^19^F{^1^H} NMR (282 MHz, CDCl_3_) δ −74.58; ^13^C{^1^H} NMR (75 MHz, CDCl_3_) δ 164.6 (C), 136.4 (C), 136.1 (C), 135.5 (C), 134.0 (C), 133.3 (C), 129.0 (CH), 128.9 (CH), 128.8 (CH), 127.9 (CH), 127.7 (d, *J* = 2.2 Hz, CH), 127.4 (CH), 125.4 (C), 125.3 (q, *J* = 1.6 Hz, CH), 125.1 (CH), 124.6 (d, *J* = 285.8 Hz, CF_3_), 120.5 (CH), 117.0 (CH), 115.5 (CH), 77.8 (d, *J* = 27.6 Hz, C), 66.2 (CH), 57.0 (q, *J* = 1.7 Hz, CH_2_); HRMS (ESI/Q-TOF) *m*/*z* [M + H]^+^ C_23_H_19_ClF_3_N_2_O_2_^+^ Calcd for 447.1082; Found 447.1085.

#### Characterization of **3ai″**

^1^H NMR (300 MHz, CDCl_3_) δ 8.97 (s, 1H), 7.60 (t, *J* = 1.7 Hz, 1H), 7.49 (d, *J* = 7.6 Hz, 1H), 7.37 (dt, *J* = 8.0, 1.5 Hz, 1H), 7.32 (d, *J* = 7.9 Hz, 1H), 7.26–7.19 (m, 3H), 7.03 (ddd, *J* = 8.5, 7.3, 1.4 Hz, 1H), 6.99–6.91 (m, 3H), 6.88–6.80 (m, 1H), 6.63 (dd, *J* = 7.8, 1.3 Hz, 1H), 4.78 (s, 1H), 4.61 (d, *J* = 15.5 Hz, 1H), 4.33 (s, 1H), 3.56 (d, *J* = 15.5 Hz, 1H);^19^F{^1^H} NMR (282 MHz, CDCl_3_) δ −73.52; ^13^C{^1^H} NMR (75 MHz, CDCl_3_) δ 165.1 (C), 136.7 (C), 135.9 (C), 134.5 (C), 132.8 (C), 129.4 (CH), 129.1 (CH), 128.9 (CH), 128.0 (CH), 127.5 (CH), 127.1 (d, *J* = 2.2 Hz, CH), 127.02 (d, *J* = 295.8 Hz, CF_3_), 126.7 (C), 124.9 (CH), 124.7 (d, *J* = 2.2 Hz, CH), 121.4 (CH), 117.6 (CH), 115.9 (CH), 78.9 (d, *J* = 28.2 Hz, C), 66.9 (CH), 58.1 (CH_2_); HRMS (ESI/Q-TOF) *m*/*z* [M + H]^+^ C_23_H_19_ClF_3_N_2_O_2_^+^ Calcd for 447.1082; Found 447.1090.

### 4-Benzyl-3-(1-(3-bromophenyl)-2,2,2-trifluoro-1-hydroxyethyl)-3,4-dihydroquinoxalin-2(1*H*)-one (3aj)

Using 4-benzyl-3,4-dihydroquinoxalin-2(1*H*)-one (**1a**, 62 mg, 0.26 mmol, 1.3 equiv) and 1-(3-bromophenyl)-2,2,2-trifluoroethan-1-one (**2j**, 30 μL, 0.2 mmol, 1 equiv), according to GP-1, compound **3aj** was obtained as a mixture of diastereoisomers (54:46 dr) that were separated by column chromatography using hexane:EtOAc mixtures (from 95:5 to 75:25): **3aj′** (33.9 mg, 0.07 mmol, 35% yield, yellow oil) and **3aj″** (28.8 mg, 0.06 mmol, 29% yield, yellow oil).

#### Characterization of **3aj′**

^1^H NMR (300 MHz, CDCl_3_) δ 8.96 (s, 1H), 7.76 (t, *J* = 1.9 Hz, 1H), 7.53 (tdd, *J* = 7.9, 1.9, 1.0 Hz, 2H), 7.30–7.17 (m, 4H), 7.03 (ddd, *J* = 8.5, 7.2, 1.4 Hz, 1H), 6.99–6.92 (m, 3H), 6.85 (td, *J* = 7.5, 1.5 Hz, 1H), 6.62 (dd, *J* = 7.8, 1.4 Hz, 1H), 4.80 (s, 1H), 4.61 (d, *J* = 15.5 Hz, 1H), 4.32 (d, *J* = 1.0 Hz, 1H), 3.55 (d, *J* = 15.6 Hz, 1H); ^13^C{^1^H} NMR (282 MHz, CDCl_3_) δ −73.48; ^13^C{^1^H} NMR (75 MHz, CDCl_3_) δ 165.1 (C), 136.9 (C), 135.9 (C), 132.8 (C), 132.1 (CH), 130.0 (q, *J* = 2.2 Hz, CH), 129.7 (CH), 128.9 (CH), 128.0 (CH), 127.6 (CH), 126.8 (C), 125.2 (q, *J* = 2.2 Hz, CH), 125.0 (CH), 122.6 (C), 121.4 (CH), 117.6 (CH), 115.9 (CH), 78.8 (q, *J* = 28.2 Hz, C), 66.9 (CH), 58.2 (CH_2_); HRMS (ESI/Q-TOF) *m*/*z* [M + H]^+^ C_23_H_19_BrF_3_N_2_O_2_^+^ Calcd for 491.0577; Found 491.0580.

#### Characterization of **3aj″**

^1^H NMR (300 MHz, CDCl_3_) δ 8.81 (s, 1H), 7.64 (d, *J* = 1.9 Hz, 1H), 7.43 (d, *J* = 7.9 Hz, 1H), 7.30 (dd, *J* = 1.9, 1.0 Hz, 1H), 7.25–7.20 (m, 4H), 7.08 (dd, *J* = 7.2, 2.3 Hz, 1H), 6.98–6.92 (m, 2H), 6.87 (dd, *J* = 7.9, 1.3 Hz, 1H), 6.67 (td, *J* = 7.5, 1.4 Hz, 1H), 6.38 (dd, *J* = 7.8, 1.4 Hz, 1H), 4.97 (s, 1H), 4.90 (d, *J* = 15.8 Hz, 1H), 4.64 (s, 1H), 4.30 (d, *J* = 15.9 Hz, 1H); ^13^C{^1^H} NMR (282 MHz, CDCl_3_) δ −74.53; ^13^C NMR (75 MHz, CDCl_3_) δ 164. (C), 136.39 (C), 136.36 (C), 133.3 (C), 131.9 (CH), 130.5 (q, *J* = 2.2 Hz, CH), 129.0 (CH), 128.9 (CH), 127.9 (CH), 127.4 (CH), 125.7 (q, *J* = 1.8 Hz, CH), 125.5 (C), 125.2 (CH), 124.6 (q, *J* = 286.4 Hz, CF_3_), 122.1 (C), 120.5 (CH), 116.9 (CH), 115.6 (CH), 77.7 (q, *J* = 27.6 Hz, C), 66.1 (CH), 57.0 (CH_2_); HRMS (ESI/Q-TOF) *m*/*z* [M + H]^+^ C_23_H_19_BrF_3_N_2_O_2_^+^ Calcd for 491.0577; Found 491.05781.

### 4-Benzyl-3-(2,2,2-trifluoro-1-hydroxy-1-(2-methoxyphenyl)ethyl)-3,4-dihydroquinoxalin-2(1*H*)-one (3ak)

Using 4-benzyl-3,4-dihydroquinoxalin-2(1*H*)-one (**1a**, 62 mg, 0.26 mmol, 1.3 equiv) and 2,2,2-trifluoro-1-(2-methoxyphenyl)ethan-1-one (**2k**, 32 μL, 0.2 mmol, 1 equiv), according to GP-1, compound **3ak** was obtained as a mixture of diastereoisomers (59:41 dr) that cannot be separated by column chromatography using hexane:EtOAc mixtures (from 95:5 to 75:25): **3ak′** + **3ak″** (32.7 mg, 0.07 mmol, 37% yield, yellow oil). Representative NMR signals for either the major and the minor diastereoisomer are labeled with one or two asterisks, respectively.

^1^H NMR (300 MHz, CDCl_3_) δ 9.36 (s, 1H*), 9.17 (s, 1H**), 7.49–7.46 (m, 2H), 7.37 (ddd, *J* = 8.8, 7.4, 1.6 Hz, 1H), 7.33–7.10 (m, 10H), 7.05–6.79 (m, 10H), 6.76–6.68 (m, 2H), 6.63 (dd, *J* = 7.7, 1.5 Hz, 1H*), 6.61–6.51 (m, 2H), 6.02 (s, 1H**), 4.96 (d, *J* = 15.8 Hz, 1H**), 4.75 (s, 1H*), 4.46 (d, *J* = 16.0 Hz, 1H*), 4.40 (d, *J* = 15.5 Hz, 1H**), 3.84 (d, *J* = 15.5 Hz, 1H*), 3.72 (s, 3H*), 3.63 (s, 3H**); ^13^C{^1^H} NMR (282 MHz, CDCl_3_) δ −72.60 *, −74.25**; ^13^C{^1^H} NMR (75 MHz, CDCl_3_) δ 163.8 (C**), 163.2 (C*), 158.0 (C*), 157.7 (C*), 137.0 (C**), 136.8 (C*), 135.8 (C*), 134.9 (C**), 134.1 (C**), 133.9 (C*), 130.3 (CH), 130.2 (CH), 128.7 (CH), 128.6 (CH), 128.54 (CH), 128.51 (CH), 128.46 (CH), 127.53 (CH), 127.48 (CH), 127.4 (CH), 123.9 (CH), 123.5 (CH), 122.9 (C), 121.5 (CH), 121.0 (CH), 120.7 (C), 119.40 (CH), 119.38 (CH), 115.3 (CH), 115.2 (CH), 112.4 (CH), 112.2 (CH), 83.7 (d, *J* = 27.1 Hz, C*), 83.3 (d, *J* = 26.5 Hz, C**), 66.51 (CH*), 66.48 (CH**), 55.96 (CH_2_), 55.94 (CH_3_**), 55.86 (CH_3_*); HRMS (ESI/Q-TOF) *m*/*z* [M + H]^+^ C_24_H_22_F_3_N_2_O_3_^+^ Calcd for 443.1577; Found 443.1589.

### 4-Benzyl-3-(1-(3,4-dichlorophenyl)-2,2,2-trifluoro-1-hydroxyethyl)-3,4-dihydroquinoxalin-2(1*H*)-one (3al)

Using 4-benzyl-3,4-dihydroquinoxalin-2(1*H*)-one (**1a**, 62 mg, 0.26 mmol, 1.3 equiv) and 1-(3,4-dichlorophenyl)-2,2,2-trifluoroethan-1-one (**2l**, 32 μL, 0.2 mmol, 1 equiv), according to GP-1, compound **3al** (41.2 mg, 0.09 mmol, 43% yield, yellow oil) was obtained as a mixture of diastereoisomers (57:43 dr) that were separated by column chromatography using hexane:EtOAc mixtures (from 95:5 to 75:25): **3al′** (23.4 mg, 0.05 mmol, 25% yield, yellow oil) and **3al″** (17.8 mg, 0.04 mmol, 18% yield, yellow oil).

#### Characterization of **3al′**

^1^H NMR (300 MHz, CDCl_3_) δ 8.84 (s, 1H), 7.68 (t, *J* = 1.2 Hz, 1H), 7.42 (t, *J* = 1.1 Hz, 2H), 7.25–7.23 (m, 3H), 7.09–6.95 (m, 4H), 6.88 (ddd, *J* = 7.8, 7.0, 1.8 Hz, 1H), 6.60 (dd, *J* = 7.8, 1.4 Hz, 1H), 4.69 (s, 1H), 4.64 (d, *J* = 15.4 Hz, 1H), 4.33 (s, 1H), 3.71 (d, *J* = 15.4 Hz, 1H); ^13^C{^1^H} NMR (282 MHz, CDCl_3_) δ −73.87; ^13^C{^1^H} NMR (75 MHz, CDCl_3_) δ 164.7 (C), 135.7 (C), 134.8 (C), 133.3 (C), 132.6 (C), 132.6 (C), 130.0 (CH), 129.0 (q, *J* = 1.9 Hz, CH), 128.9 (CH), 128.2 (CH), 127.6 (CH), 127.0 (C), 125.94 (q, *J* = 1.8 Hz, CH), 125.91 (q, *J* = 274.5 Hz, CF_3_), 125.0 (CH), 121.9 (CH), 118.1 (CH), 115.9 (CH), 78.5 (q, *J* = 28.2 Hz, C), 66.7 (CH), 58.7 (CH_2_); HRMS (ESI/Q-TOF) *m*/*z* [M + H]^+^ C_23_H_18_Cl_2_F_3_N_2_O_2_^+^ Calcd for 481.0692; Found 481.0699.

#### Characterization of **3al″**

^1^H NMR (300 MHz, CDCl_3_) δ 8.00 (s, 1H), 7.57 (d, *J* = 2.2 Hz, 1H), 7.31 (dd, *J* = 8.6, 2.3 Hz, 1H), 7.26–7.21 (m, 3H), 7.14–7.06 (m, 3H), 6.97 (ddd, *J* = 8.6, 7.2, 1.4 Hz, 1H), 6.91–6.86 (m, 1H), 6.70 (td, *J* = 7.6, 1.4 Hz, 1H), 6.36 (dd, *J* = 7.8, 1.4 Hz, 1H), 5.02 (s, 1H), 4.93 (d, *J* = 15.9 Hz, 1H), 4.63 (s, 1H), 4.37 (d, *J* = 15.8 Hz, 1H); ^13^C{^1^H} NMR (282 MHz, CDCl_3_) δ −74.83; ^13^C{^1^H} NMR (75 MHz, CDCl_3_) δ 164.4 (C), 136.3 (C), 134.2 (C), 133.2 (C), 133.2 (C), 132.1 (C), 129.7 (q, *J* = 2.2 Hz, CH), 129.4 (CH), 128.9 (CH), 128.0 (CH), 127.4 (CH), 126.6 (q, *J* = 2.2 Hz, CH), 125.31 (CH), 125.28 (C), 120.7 (CH), 117.3 (CH), 115.4 (CH), 77.2 (q, *J* = 32.1 Hz, C), 66.1 (CH), 57.3 (d, *J* = 1.7 Hz, CH_2_); HRMS (ESI/Q-TOF) *m*/*z* [M + H]^+^ C_23_H_18_Cl_2_F_3_N_2_O_2_^+^ Calcd for 481.0692; Found 481.0697.

### 4-Benzyl-3-(2,2,2-trifluoro-1-hydroxy-1-(thiophen-2-yl)ethyl)-3,4-dihydroquinoxalin-2(1*H*)-one (3am)

Using 4-benzyl-3,4-dihydroquinoxalin-2(1*H*)-one (**1a**, 62 mg, 0.26 mmol, 1.3 equiv) and 2,2,2-trifluoro-1-(thiophen-2-yl)ethan-1-one (**2m**, 26 μL, 0.2 mmol, 1 equiv), according to GP-1, compound **3am** was obtained as a mixture of diastereoisomers (58:42 dr) that were separated by column chromatography using hexane:EtOAc mixtures (from 95:5 to 75:25): **3am′** (25.1 mg, 0.06 mmol, 30% yield, yellow oil) and **3am″** (18.4 mg, 0.04 mmol, 22% yield, yellow oil).

#### Characterization of **3am′**

^1^H NMR (300 MHz, CDCl_3_) δ 8.74 (s, 1H), 7.38 (dd, *J* = 5.1, 1.2 Hz, 1H), 7.24–7.17 (m, 4H), 7.06 (dd, *J* = 5.1, 3.7 Hz, 1H), 7.03–6.92 (m, 4H), 6.84 (ddd, *J* = 7.8, 6.8, 1.9 Hz, 1H), 6.67 (dd, *J* = 7.7, 1.3 Hz, 1H), 5.37 (s, 1H), 4.64 (d, *J* = 15.7 Hz, 1H), 4.34 (s, 1H), 3.47 (d, *J* = 15.7 Hz, 1H); ^13^C{^1^H} NMR (282 MHz, CDCl_3_) δ −75.61; ^13^C{^1^H} NMR (101 MHz, CDCl_3_) δ 166.0 (C), 138.8 (C), 136.2 (C), 133.0 (C), 128.80 (CH), 127.96 (CH), 127.5 (CH), 126.7 (CH), 126.53 (q, *J* = 2.2 Hz, CH), 126.4 (C), 124.9 (CH), 124.5 (q, *J* = 286.9 Hz, CF_3_), 121.2 (CH), 117.9 (CH), 115.8 (CH), 78.5 (q, *J* = 29.3 Hz, C), 67.4 (CH), 58.2 (CH_2_); HRMS (ESI/Q-TOF) *m*/*z* [M + H]^+^ C_21_H_18_F_3_N_2_O_2_S^+^ Calcd for 419.1036; Found 419.1039.

#### Characterization of **3am″**

^1^H NMR (300 MHz, CDCl_3_) δ 8.17 (s, 1H), 7.29–7.18 (m, 4H), 7.16–7.05 (m, 3H), 7.02–6.87 (m, 1H), 6.82 (dt, *J* = 3.6, 1.1 Hz, 1H), 6.73–6.61 (m, 2H), 6.39 (dd, *J* = 7.8, 1.3 Hz, 1H), 5.41 (s, 1H), 4.98 (d, *J* = 16.1 Hz, 1H), 4.66 (s, 1H), 4.39 (d, *J* = 16.0 Hz, 1H); ^13^C{^1^H} NMR (282 MHz, CDCl_3_) δ −76.67; ^13^C{^1^H} NMR (101 MHz, CDCl_3_) δ 165.1 (C), 138.0 (C), 137.9 (C), 136.6 (C), 133.5 (C), 129.1 (CH), 128.9 (CH), 127.9 (CH), 127.3 (CH), 127.1 (CH), 126.7 (CH), 125.0 (CH), 120.1 (CH), 116.9 (CH), 115.4 (CH), 78.5 (q, *J* = 29.6 Hz, C), 66.2 (CH), 56.7 (CH_2_); HRMS (ESI/Q-TOF) *m*/*z* [M + H]^+^ C_21_H_18_F_3_N_2_O_2_S^+^ Calcd for 419.1036; Found 419.1037.

### 3-(1-(4-Chlorophenyl)-2,2,2-trifluoro-1-hydroxyethyl)-4-(4-methoxybenzyl)-3,4-dihydroquinoxalin-2(1*H*)-one (3cd)

Using 4-(4-methoxybenzyl)-3,4-dihydroquinoxalin-2(1*H*)-one (**1c**, 69.8 mg, 0.26 mmol, 1.3 equiv) and 1-(4-chlorophenyl)-2,2,2-trifluoroethan-1-one (**2d**, 30 μL, 0.2 mmol, 1 equiv), according to GP-1, compound **3cd** was obtained as a mixture of diastereoisomers (60:40 dr) that were separated by column chromatography using hexane:EtOAc mixtures (from 95:5 to 75:25): **3cd′** (35.3 mg, 0.07 mmol, 37% yield, yellow oil) and **3cd″** (23.7 mg, 0.05 mmol, 25% yield, yellow oil).

#### Characterization of **3cd′**

^1^H NMR (300 MHz, CDCl_3_) δ 8.90 (s, 1H), 7.50 (d, *J* = 8.7 Hz, 2H), 7.32 (d, *J* = 8.9 Hz, 1H), 7.07–6.95 (m, 2H), 6.92–6.84 (m, 3H), 6.75 (d, *J* = 8.7 Hz, 1H), 6.56 (dd, *J* = 7.7, 1.3 Hz, 1H), 4.71 (s, 1H), 4.54 (d, *J* = 15.2 Hz, 1H), 4.31 (s, 1H), 3.73 (s, 3H), 3.58 (d, *J* = 15.2 Hz, 1H); ^13^C{^1^H} NMR (282 MHz, CDCl_3_) δ −73.81; ^13^C{^1^H} NMR (75 MHz, CDCl_3_) δ 165.1 (C), 159.3 (C), 135.0 (C), 133.2 (C), 132.9 (C), 129.0 (CH), 128.3 (CH), 127.8 (C), 127.1 (C), 124.9 (q, *J* = 272.0 Hz, CF_3_), 124.8 (CH), 121.5 (CH), 118.0 (CH), 115.8 (CH), 114.2 (CH), 78.8 (q, *J* = 28.2 Hz, C), 66.3 (CH), 58.0 (CH_2_), 55.2 (CH_3_); HRMS (ESI/Q-TOF) *m*/*z* [M + H]^+^ C_24_H_21_ClF_3_N_2_O_3_^+^ Calcd for 477.1187; Found 477.1192.

#### Characterization of **3cd″**

^1^H NMR (300 MHz, CDCl_3_) δ 8.24 (s, 1H), 7.38 (d, *J* = 8.6 Hz, 2H), 7.02 (d, *J* = 6.5 Hz, 2H), 6.99 (d, *J* = 6.0 Hz, 2H), 6.97–6.81 (m, 2H), 6.77 (d, *J* = 8.7 Hz, 2H), 6.69 (td, *J* = 7.6, 1.3 Hz, 1H), 6.32 (dd, *J* = 7.8, 1.4 Hz, 1H), 4.88 (s, 1H), 4.80 (d, *J* = 15.5 Hz, 1H), 4.60 (s, 1H), 4.23 (d, *J* = 15.6 Hz, 1H), 3.74 (s, 3H); ^19^F{^1^H} NMR (282 MHz, CDCl_3_) δ −72.34; ^13^C{^1^H} NMR (75 MHz, CDCl_3_) δ 164.7 (C), 159.3 (C), 135.0 (C), 133.5 (C), 132.6 (C), 128.8 (CH), 128.6 (q, *J* = 2.2 Hz, CH), 128.3 (C), 127.7 (CH), 125.7 (C), 124.9 (CH), 120.4 (CH), 117.3 (CH), 115.4 (CH), 114.2 (CH), 77.72 (q, *J* = 27.6 Hz, C), 65.9 (CH), 56.8 (CH_2_), 55.2 (CH_3_); HRMS (ESI/Q-TOF) *m*/*z* [M + H]^+^ C_24_H_21_ClF_3_N_2_O_3_^+^ Calcd for 477.1187; Found 477.1189.

### Ethyl 3-(1-benzyl-3-oxo-1,2,3,4-tetrahydroquinoxalin-2-yl)-4,4,4-trifluoro-3-hydroxybutanoate (3an)

Using 4-benzyl-3,4-dihydroquinoxalin-2(1*H*)-one (**1a**, 62 mg, 0.26 mmol, 1.3 equiv) and ethyl 4,4,4-trifluoro-3-oxobutanoate (**2n**, 29 uL, 0.2 mmol, 1 equiv), according to GP-1, compound **3an** was obtained as a mixture of diastereoisomers (55:45 dr) that cannot be separated by column chromatography using hexane:EtOAc mixtures (from 95:5 to 75:22): **3an′** + **3an″** (16.9 mg, 0.04 mmol, 20% yield, yellow oil).

^1^H NMR (300 MHz, CDCl_3_) δ 8.27 (s, 1H), 8.25 (s, 1H), 7.32–7.19 (m, 6H), 7.17–7.12 (m, 4H), 7.00–6.95 (m, 2H), 6.93–6.86 (m, 2H), 6.85–6.79 (m, 2H), 6.74–6.69 (m, 2H), 5.80 (s, 1H), 5.46 (s, 1H), 4.91–4.84 (m, 2H), 4.58–4.43 (m, 3H), 4.34 (s, 1H), 4.26–4.02 (m, 4H), 3.07 (d, *J* = 16.5 Hz, 1H), 2.88 (d, *J* = 16.4 Hz, 1H), 2.76 (d, *J* = 16.3 Hz, 1H), 2.66 (d, *J* = 16.4 Hz, 1H), 1.29–1.21 (m, 6H); ^19^F{^1^H} NMR (282 MHz, CDCl_3_) δ −77.50, −77.68; ^13^C{^1^H} NMR (75 MHz, CDCl_3_) δ 171.7 (C), 171.5 (C), 163.8 (C), 163.2 (C), 136.7 (C), 136.5 (C), 133.8 (C), 133.0 (C), 128.7 (CH), 128.7 (CH), 127.8 (CH), 127.7 (CH), 127.7 (CH), 127.5 (CH), 127.1 (C), 127.0 (C), 124.4 (CH), 124.4 (CH), 120.6 (CH), 120.2 (CH), 117.2 (CH), 116.6 (CH), 115.3 (CH), 115.1 (CH), 65.1 (CH), 64.8 (CH), 61.7 (CH_2_), 61.7 (CH_2_), 57.9 (CH_2_), 56.9 (CH_2_), 35.2 (q, *J* = 1.7 Hz, CH_2_), 33.9 (CH_2_), 13.91 (CH_3_), 13.85 (CH_3_); HRMS (ESI/Q-TOF) *m*/*z* [M + H]^+^ C_21_H_22_F_3_N_2_O_4_^+^ Calcd for 423.1526; Found 423.1527.

### 4-(1-(1-Benzyl-3-oxo-1,2,3,4-tetrahydroquinoxalin-2-yl)-2,2,2-trifluoro-1-hydroxyethyl)phenyl 2-(1-(4-chlorobenzoyl)-5-methoxy-2-methyl-1*H*-indol-3-yl)acetate (3ao)

Using 4-benzyl-3,4-dihydroquinoxalin-2(1*H*)-one (**1a**, 62 mg, 0.26 mmol, 1.3 equiv) and 4-(2,2,2-trifluoroacetyl)phenyl 2-(1-(4-chlorobenzoyl)-5-methoxy-2-methyl-1*H*-indol-3-yl)acetate (**2o**, 106 mg, 0.2 mmol, 1 equiv), according to GP-1, compound **3ao** was obtained as a mixture of diastereoisomers (55:45 dr) that were separated by column chromatography using DCM:EtOAc mixtures (from 99:1 to 95:5): **3ao′** (54.1 mg, 0.07 mmol, 35% yield, yellow oil) and **3ao″** (44.2 mg, 0.06 mmol, 29% yield, yellow oil).

#### Characterization of **3ao′**

^1^H NMR (300 MHz, CDCl_3_) δ 8.50 (s, 1H), 7.68 (d, *J* = 8.7 Hz, 2H), 7.59 (d, *J* = 8.8 Hz, 2H), 7.48 (d, *J* = 8.7 Hz, 2H), 7.23–7.16 (m, 3H), 7.09 (d, *J* = 8.9 Hz, 2H), 7.06 (d, *J* = 2.5 Hz, 1H), 7.03–6.95 (m, 1H), 6.94–6.86 (m, 4H), 6.80 (td, *J* = 7.5, 1.5 Hz, 1H), 6.71 (dd, *J* = 9.0, 2.5 Hz, 1H), 6.61 (dd, *J* = 7.8, 1.3 Hz, 1H), 4.82 (s, 1H), 4.56 (d, *J* = 15.6 Hz, 1H), 4.29 (s, 1H), 3.92 (s, 2H), 3.84 (s, 3H), 3.48 (d, *J* = 15.6 Hz, 1H), 2.47 (s, 3H); ^13^C{^1^H} NMR (282 MHz, CDCl_3_) δ −73.95; ^13^C{^1^H} NMR (75 MHz, CDCl_3_) δ 169.0 (C), 168.3 (C), 165.0 (C), 156.1 (C), 151.1 (C), 139.4 (C), 136.3 (C), 136.0 (C), 133.8 (C), 133.0 (C), 132.4 (C), 131.2 (CH), 130.9 (C), 130.5 (C), 129.2 (CH), 128.8 (CH), 127.9 (CH), 127.9 (q, *J*_C–F_ = 1.5 Hz, CH), 127.5 (CH), 126.6 (C), 124.8 (CH), 121.2 (CH), 121.2 (CH), 117.4 (CH), 115.8 (CH), 115.0 (CH), 111.8 (C), 111.7 (CH), 101.3 (CH), 78.9 (q, *J*_C–F_ = 28.2 Hz, C), 67.0 (CH), 57.9 (CH_2_), 55.8 (CH_3_), 30.6 (CH_2_), 13.4 (CH_3_); HRMS (ESI/Q-TOF) *m*/*z* [M + H]^+^ C_42_H_34_ClF_3_N_3_O_6_^+^ Calcd for 768.2083; Found 768.2099.

#### Characterization of **3ao″**

^1^H NMR (300 MHz, CDCl_3_) δ 8.21 (s, 1H), 7.66 (d, *J* = 8.7 Hz, 2H), 7.53–7.36 (m, 4H), 7.25–7.14 (m, 3H), 7.10–7.03 (m, 2H), 7.00 (d, *J* = 2.5 Hz, 1H), 6.94–6.83 (m, 2H), 6.82–6.74 (m, 3H), 6.69 (dd, *J* = 9.0, 2.5 Hz, 1H), 6.60 (td, *J* = 7.6, 1.3 Hz, 1H), 6.35 (dd, *J* = 7.8, 1.3 Hz, 1H), 4.89–4.77 (m, 2H), 4.61 (s, 1H), 4.25 (d, *J* = 15.9 Hz, 1H), 3.85 (s, 2H), 3.83 (s, 3H), 2.42 (s, 3H); ^13^C{^1^H} NMR (282 MHz, CDCl_3_) δ −74.88; ^13^C{^1^H} NMR (75 MHz, CDCl_3_) δ 168.8 (C), 168.3 (C), 164.6 (C), 156.1 (C), 151.0 (C), 139.4 (C), 136.5 (C), 136.2 (C), 133.8 (C), 133.3 (C), 131.8 (C), 131.2 (CH), 130.8 (C), 130.4 (C), 129.2 (CH), 128.8 (CH), 128.3 (d, *J*_C–F_ = 1.4 Hz, CH), 127.8 (CH), 127.3 (CH), 125.6 (C), 124.8 (CH), 120.5 (CH), 120.2 (CH), 116.5 (CH), 115.6 (CH), 115.0 (CH), 111.8 (C), 111.6 (CH), 101.3 (CH), 78.1 (d, *J*_C–F_ = 27.4 Hz, C), 66.5 (CH), 56.6 (CH_2_), 55.7 (CH_3_), 30.5 (CH_2_), 13.4 (CH_3_); HRMS (ESI/Q-TOF) *m*/*z* [M + H]^+^ C_42_H_34_ClF_3_N_3_O_6_^+^ Calcd for 768.2083; Found 768.2102.

### Ethyl 2-(1-Benzyl-3-oxo-1,2,3,4-tetrahydroquinoxalin-2-yl)-3,3,3-trifluoro-2-hydroxypropanoate (5)

Using 4-benzyl-3,4-dihydroquinoxalin-2(1*H*)-one (**1a**, 23.8 mg, 0.1 mmol, 1 equiv) and ethyl 3,3,3-trifluoropyruvate (**4**, 17 μL, 0.13 mmol, 1.3 equiv), according to SP-1, compound **5** was obtained as a mixture of diastereoisomers (54:46 dr) that were separated by column chromatography using hexane:EtOAc mixtures (from 95:5 to 75:25): **5′** (5.5 mg, 0.014 mmol, 14% yield, yellow oil) and **5″** (4.7 mg, 0.011 mmol, 11% yield, yellow oil).

#### Characterization of **5′**

^1^H NMR (300 MHz, CDCl_3_) δ 8.49 (s, 1H), 7.26–7.22 (m, 3H), 7.07 (dd, *J* = 7.2, 2.4 Hz, 2H), 7.01–6.83 (m, 3H), 6.72 (dd, *J* = 7.9, 1.6 Hz, 1H), 4.63 (d, *J* = 15.1 Hz, 1H), 4.57 (s, 1H), 4.53–4.36 (m, 1H), δ 4.29–4.16 (m, 1H), 4.19 (d, *J* = 15.0 Hz, 1H), 3.78 (s, 1H), 1.35 (t, *J* = 7.2 Hz, 3H);^19^F{^1^H} NMR (282 MHz, CDCl_3_) δ −73.97; ^13^C{^1^H} NMR (75 MHz, CDCl_3_) δ 167.95 (q, *J* = 1.1 Hz, C), 161.8 (C), 136.1 (C), 133.2 (C), 129.2 (C), 128.7 (CH), 128.0 (CH), 127.8 (CH), 125.6 (d, *J* = 266.5 Hz, CF_3_), 124.0 (CH), 121.9 (CH), 119.1 (CH), 115.6 (CH), 81.3 (q, *J* = 29.3 Hz, C), 64.3 (CH_2_), 63.6 (CH), 59.3 (CH_2_), 13.9 (CH_3_); HRMS (ESI/Q-TOF) *m*/*z* [M + H]^+^ C_20_H_20_F_3_N_2_O_4_^+^ Calcd for 409.1370; Found 409.1373.

#### Characterization of **5″**

^1^H NMR (300 MHz, CDCl_3_) δ 8.62 (s, 1H), 7.25–7.21 (m, 2H), 7.15 (dd, *J* = 7.9, 1.7 Hz, 2H), 6.99-6.84 (m, 3H), 6.79 (td, *J* = 7.5, 1.4 Hz, 1H), 6.70 (dd, *J* = 7.8, 1.5 Hz, 1H), 4.94 (d, *J* = 15.9 Hz, 1H), 4.77 (s, 1H), 4.50–4.18 (m, 4H), 1.34 (t, *J* = 7.2 Hz, 3H); ^13^C{^1^H} NMR (282 MHz, CDCl_3_) δ −73.71; ^13^C{^1^H} NMR (75 MHz, CDCl_3_) δ 167.7 (C), 163.8 (C), 136.7 (C), 133.2 (C), 128.7 (CH), 127.6 (CH), 127.3 (CH), 127.1 (C), 124.3 (CH), 120.1 (CH), 116.3 (CH), 115.0 (CH), 79.8 (q, *J* = 28.7 Hz, C), 65.4 (CH), 64.6 (CH_2_), 56.0 (q, *J* = 1.7 Hz, CH_2_), 13.7 (CH_3_); HRMS (ESI/Q-TOF) m/z [M + H]^+^ C_20_H_20_F_3_N_2_O_4_^+^ Calcd for 409.1370; Found 409.1378.

### 1-(1-Benzyl-1,2,3,4-tetrahydroquinoxalin-2-yl)-2,2,2-trifluoro-1-phenylethan-1-ol (6)

Using 4-benzyl-3-(2,2,2-trifluoro-1-hydroxy-1-phenylethyl)-3,4-dihydroquinoxalin-2(1*H*)-one (**3aa**, 78.4 mg, 0.19 mmol, 1 equiv), according to SP-4, compound **6** was obtained as a mixture of diastereoisomers (52:48 dr) that were separated by column chromatography using hexane:EtOAc mixtures (from 95:5 to 75:25): **6′** (27.8 mg, 0.068 mmol, 36% yield, yellow oil) and **6″** (25.7 mg, 0.062 mmol, 34% yield, yellow oil).

#### Characterization of **6′**

^1^H NMR (300 MHz, CDCl_3_) δ 7.62 (dd, *J* = 6.4, 2.8 Hz, 2H), 7.52–7.33 (m, 4H), 7.22–7.08 (m, 3H), 6.90 (dd, *J* = 7.4, 2.2 Hz, 2H), 6.79–6.67 (m, 2H), 6.60 (td, *J* = 7.5, 1.2 Hz, 1H), 6.49 (dd, *J* = 8.3, 1.0 Hz, 1H), 4.11 (d, *J* = 17.1 Hz, 1H), 4.07–3.97 (m, 2H), 3.32–3.21 (m, 1H), 3.01 (d, *J* = 17.1 Hz, 1H); ^13^C{^1^H} NMR (282 MHz, CDCl_3_) δ −71.82; ^13^C{^1^H} NMR (75 MHz, CDCl_3_) δ 137.8 (C), 137.5 (C), 135.3 (C), 130.4 (C), 128.4 (CH), 128.3 (CH), 128.2 (CH), 126.90 (CH), 126.88 (CH), 126.3 (CH), 126.2 (q, *J* = 289.7 Hz, CF_3_), 122.2 (CH), 116.7 (CH), 116.0 (CH), 112.9 (CH), 82.5 (d, *J* = 25.4 Hz, C), 60.1 (CH), 54.0 (CH_2_), 42.6 (q, *J* = 2.2 Hz, CH_2_); HRMS (ESI/Q-TOF) m/z [M + H]^+^ C_23_H_22_F_3_N_2_O^+^ Calcd for 399.1679; Found 399.1677.

#### Characterization of **6″**

^1^H NMR (300 MHz, CDCl_3_) δ 7.64 (d, *J* = 7.2 Hz, 2H), 7.53–7.37 (m, 4H), 7.34–7.20 (m, 5H), 6.88–6.78 (m, 2H), 6.74–6.56 (m, 2H), 5.12 (d, *J* = 17.1 Hz, 1H), 4.57 (d, *J* = 17.1 Hz, 1H), 4.19 (dd, *J* = 3.5, 1.7 Hz, 1H), 3.02 (dd, *J* = 11.2, 1.7 Hz, 1H), 2.91 (dd, *J* = 11.2, 3.4 Hz, 1H);^19^F{^1^H} NMR (282 MHz, CDCl_3_) δ −72.58; ^13^C{^1^H} NMR (75 MHz, CDCl_3_) δ 138.4 (C), 137.6 (C), 134.5 (C), 129.8 (C), 128.7 (CH), 128.62 (CH), 128.56 (CH), 127.3 (CH), 127.2 (CH), 126.2 (q, *J* = 1.4 Hz, CH), 122.4 (CH), 117.2 (CH), 116.3 (CH), 113.8 (CH), 81.5 (q, *J* = 27.1 Hz, C), 59.2 (CH), 54.6 (q, *J* = 3.3 Hz, CH_2_), 41.0 (CH_2_); HRMS (ESI/Q-TOF) *m*/*z* [M + H]^+^ C_23_H_22_F_3_N_2_O^+^ Calcd for 399.1679; Found 399.1675.

### 4-Benzyl-3-(1-chloro-2,2,2-trifluoro-1-phenylethyl)-3,4-dihydroquinoxalin-2(1*H*)-one (7)

Using 4-benzyl-3-(2,2,2-trifluoro-1-hydroxy-1-phenylethyl)-3,4-dihydroquinoxalin-2(1*H*)-one (**3aa**, 26.9 mg, 0.05 mmol, 1 equiv), according to SP-5, compound **7** was obtained as a mixture of diastereoisomers (50:50 dr) that were separated by column chromatography using hexane:Et_2_O mixtures (from 5:5 to 2:8): **7′** (11.3 mg, 0.025 mmol, 40% yield, yellow oil) and **7″** (11.5 mg, 0.025 mmol, 40% yield, yellow oil).

#### Characterization of **7′**

^1^H NMR (300 MHz, CDCl_3_) δ 9.11 (s, 1H), 7.67 (d, *J* = 6.5 Hz, 2H), 7.46–7.35 (m, 3H), 7.22–7.12 (m, 3H), 6.98–6.84 (m, 3H), 6.77 (td, *J* = 7.6, 1.2 Hz, 1H), 6.71–6.62 (m, 2H), 4.86 (s, 1H), 4.32 (d, *J* = 15.8 Hz, 1H), 3.42 (d, *J* = 15.9 Hz, 1H); ^13^C{^1^H} NMR (282 MHz, CDCl_3_) δ −67.14; ^13^C{^1^H} NMR (75 MHz, CDCl_3_) δ 161.0 (C), 136.3 (C), 133.4 (C), 133.3 (C), 129.5 (CH), 128.7 (CH), 128.4 (CH), 127.9 (q, *J* = 2.2 Hz, CH), 127.7 (CH), 127.3 (C), 127.2 (CH), 124.1 (CH), 124.0 (q, *J* = 284.7 Hz, CF_3_), 120.1 (CH), 116.1 (CH), 115.4 (CH), 77.1 (q, *J* = 27.7 Hz, C), 67.9 (CH), 56.4 (CH_2_); HRMS (ESI/Q-TOF) m/z [M + H]^+^ C_23_H_19_ClF_3_N_2_O^+^ Calcd for 431.1133; Found 431.1136.

#### Characterization of **7″**

^1^H NMR (300 MHz, CDCl_3_) δ 8.30 (s, 1H), 7.57 (d, *J* = 8.3 Hz, 2H), 7.45–7.20 (m, 6H), 7.14 (dd, *J* = 7.6, 1.7 Hz, 2H), 6.97 (ddd, *J* = 8.3, 7.0, 1.4 Hz, 1H), 6.92 (dd, *J* = 8.0, 1.6 Hz, 1H), 6.82–6.71 (m, 1H), 6.46 (dd, *J* = 7.7, 1.2 Hz, 1H), 5.01 (d, *J* = 15.7 Hz, 1H), 5.00 (s, 1H), 4.48 (d, *J* = 15.7 Hz, 1H); ^13^C{^1^H} NMR (282 MHz, CDCl_3_) δ −69.14; ^13^C{^1^H} NMR (101 MHz, CDCl_3_) δ 161.1 (C), 136.5 (C), 133.3 (C), 132.5 (C), 129.2 (CH), 128.9 (CH), 128.1 (CH), 127.9 (CH), 127.7 (q, *J* = 2.0 Hz, CH), 127.5 (CH), 127.2 (C), 124.2 (CH), 120.3 (CH), 116.4 (CH), 115.0 (CH), 68.2 (CH), 56.9 (CH_2_); HRMS (ESI/Q-TOF) *m*/*z* [M + H]^+^ C_23_H_19_ClF_3_N_2_O^+^ Calcd for 431.1133; Found 431.1132.

### 1,1′-Dibenzyl-1,1′,4,4′-tetrahydro-[2,2′-biquinoxaline]-3,3′(2*H*,2′*H*)-dione (8)

In low-yielding reactions, a large amount of dimeric dihydroquinoxalin-2-one (8) was obtained. It was isolated as a single diasteromer by removing the mother liquor and washing the solid with DCM. The presence of this dimeric specie is consistent with the generation of the α-aminoradical under our photoredox conditions.

^1^H NMR (300 MHz, DMSO-*d*_6_) δ 10.68 (bs, 2H), 7.25–7.11 (m, 6H), 7.07–6.96 (m, 4H), 6.88 (dd, *J* = 7.3, 1.9 Hz, 2H), 6.79–6.63 (m, 4H), 6.41 (dd, *J* = 7.5, 1.7 Hz, 2H), 4.65 (d, *J* = 15.7 Hz, 2H), 4.02 (d, *J* = 15.8 Hz, 2H), 3.94 (s, 2H); ^13^C NMR (75 MHz, DMSO-*d*_6_) δ 164.4 (C), 137.5 (C), 132.4 (C), 128.4 (CH), 127.2 (CH), 127.1 (CH), 127.0 (C), 123.0 (CH), 118.7 (CH), 115.0 (CH), 114.1 (CH), 63.2 (CH), 53.2 (CH_2_); HRMS (ESI-QTOF) *m*/*z* [M + H]^+^ C_30_H_27_N_4_O_2_ Calcd for 475.2129; Found 475.2133.

## Supporting Information Available

^1^H, ^19^F, and ^13^C NMR spectra for all compounds. FAIR Data is available as Supporting Information for Publication and includes the primary NMR FID files for Compounds **3**, **5**, **6**, **7**, and **8** (PDF)
FAIR data, including the primary NMR FID files, for compounds **2o**, **3aa**, **3ab**, **3ac**, **3ad**, **3ae**, **3af**, **3ag**, **3ah**, **3ai**, **3aj**, **3ak**, **3al**, **3am**, **3an**, **3ao**, **3ba**, **3ca**, **3cd**, **3da**, **3ea**, **3fa(1)**, **3ga**, **3ha**, **3ia**, **3la**, **5**, **6**, **7** (ZIP)
