# Supplementary material for: Radical Addition of Dihydroquinoxalin-2-ones to Trifluoromethyl Ketones under Visible-Light Photoredox Catalysis
Source: J Org Chem. 2022 Jul 5;87(14):9343–56. doi: 10.1021/acs.joc.2c01139 (PMC9295130; doi:10.1021/acs.joc.2c01139)

Radical Addition of Dihydroquinoxalin-2-ones to Trifluoromethyl Ketones under Visible-Light Photoredox Catalysis

Jaume Rostoll-Berenguer, María Martín-López, Gonzalo Blay, José R. Pedro* and Carlos Vila*

Departament de Química Orgànica, Facultat de Química, Universitat de València, Dr. Moliner 50, 46100 Burjassot, València (Spain).E-mail: carlos.vila@uv.es, jose.r.pedro@uv.es

Mechanistic Studies S2

1. Luminescence Emission Quenching Studies S2
2. NMR Studies: S3
3. Quantum Yield measurement: S4
4. On/off experiment: S7

Typical reaction setup for the 0.2 mmol scale reaction S8

NMR Spectra S9

Mechanistic Studies

1. Luminescence Emission Quenching Studies

MeCN over 3Å MS was degassed by sonication and N_2 bubbling simultaneously. The measurement solutions were prepared from stock solutions of **1a** (32 mM), **2a** (32 mM), and $Ru(bpy)_3Cl_2$ (**Ru**, 0.4 mM) in anhydrous and degassed MeCN. Table S1 shows the concentration of each analyte in the measurement solutions for all the experiences.

Solution	[1a] (mM)	[2a] (mM)	[Ru] (mM)
A0	-	0,0	0.02
A1	-	9,6	0.02
A2	-	19,2	0.02
B0	9,6	0,0	0.02
B1	9,6	9.6	0.02
B2	9,6	19,2	0.02
C0	0,0	9,6	0.02
C1	9.6	9,6	0.02
C2	19,2	9,6	0.02

Table S1: Concentration of 1a, 2a and $Ru(bpy)_3Cl_2$ in each solution.

All the emission spectra were obtained using a Jasco FP-750 Spectrofluorometer selecting 450 nm as the excitation wavelength.

In a previous work we demonstrated that 4-benzilquinoxalin-2-one **1a** cannot quench the excited state of $Ru(bpy)_3Cl_2$ in MeCN.[1]

The emission spectrum of each series of solutions were plotted in Figures 1A, 1B and 1C.

References:

1. Rostoll-Berenguer, J.; Blay, G.; Pedro, J. R.; Vila, C. *Org. Lett.* **2020**, *22*, 8012-8017.

2. NMR Studies:

A solution of 4-benzylquinoxalin-2-one (**1a**, 5 mg) in MeCN-d^3 (red line) was treated with increasing amounts of a 693 mM solution of trifluoroacetophenone **2a** in MeCN-d^3.

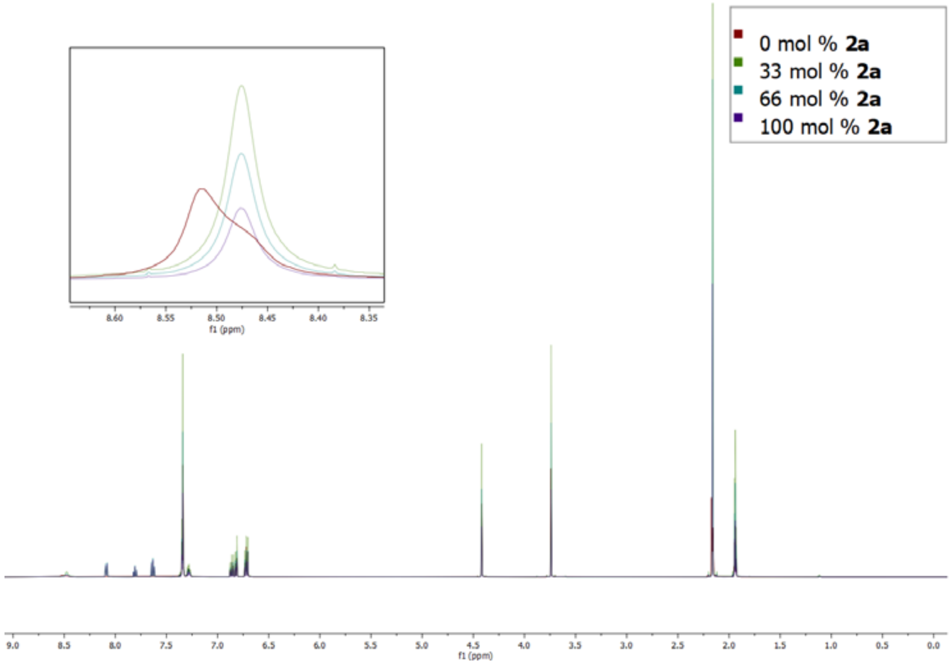

*Figure S1: NMR titration experiment of **1a** with **2a**.*

A potential interaction between Ru(bpy)$_3$Cl$_2$ and trifluoroacetophenone **2a** was also examined by NMR in MeCN-d^3 (Figure S2). Nevertheless, it seems that there is not any interaction between them, considering that there is not any change in the chemical shift of **2a** with and without Ru(bpy)$_3$Cl$_2$.

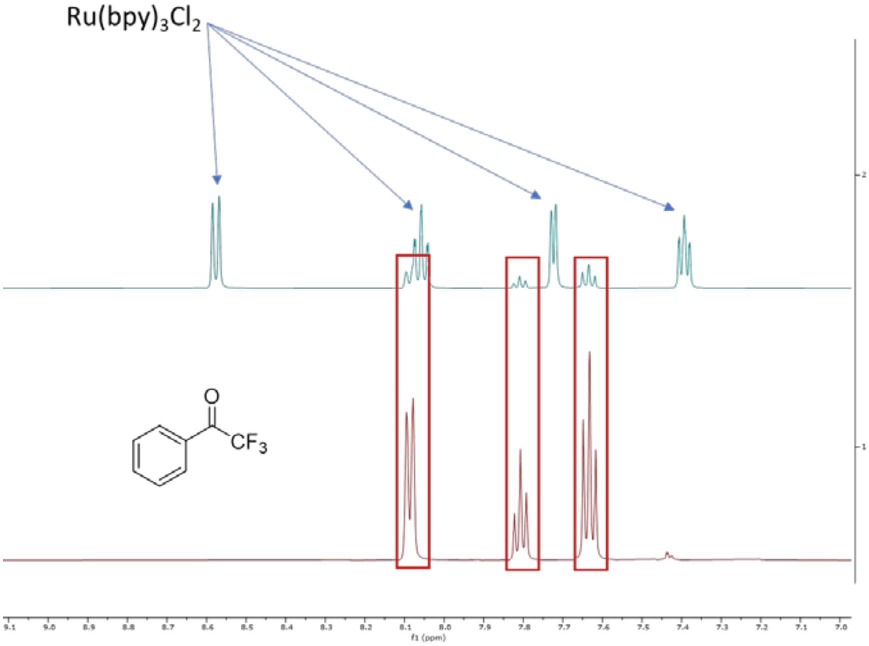

*Figure S2: NMR experiment of Ru(bpy)$_2$Cl$_2$·6H$_2$O with **2a**.*

3. Quantum Yield measurement:

Quantum Yield measurements were determined using standard ferrioxalate chemical actinometry as described by Hatchard and Parker[16] and the modifications implemented by Yoon[17] and Melchiorre.[18]

1. Determination of the photon flux:

The photon flux of the HP Single LED was determined by monitoring the photoreduction of Fe(III) in potassium ferrioxalate to Fe(II), upon complexation with 1,10-phenanthroline:

$$K_3[Fe^{III}(C_2O_4)]_3 \xrightarrow{h\nu} Fe^{II} \xrightarrow{phen} Fe^{II}(phen)_3$$

strong absorption at 510 nm

The photoreduction of potassium ferrioxalate is an extensively studied transformation and its quantum yield is already known for many wavelengths. Therefore, the determination of the rate of this photoreaction by the action of our photoreactor allows us to obtain its photon flux.

The following solutions were prepared:

a. **Actinometer solution**: 294.8 mg of potassium ferrioxalate trihydrate (purchased from Alfa Aesar, catalogue number 31124) and 139 uL of H_2SO_4 96% were added to a 50 mL volumetric flask and filled to the mark with Nanopure water.
b. **Phenanthroline solution**: 100 mg of 1,10-phenanthroline were added to a 50 mL volumetric flask and filled to the mark with Nanopure water.
c. **Buffer solution**: 2.47 g of sodium acetate and 0.5 mL of H_2SO_4 96% were added to a 50 mL volumetric flask and filled to the mark with Nanopure water.

1 mL of the actinometer solution was added to an ovendried Schlenck tube and was irradiated by Blue LEDs for 3 seconds. After the irradiation, the mixture was quantitatively transferred to a 10 mL volumetric flask containing 0.5 mL of the phenanthroline solution and 2 mL of the buffer solution. Then, the flask was filled to the mark with Nanopure water and was left in the dark for 1 h to ensure the quantitative formation of $Fe^{II}(phen)_3^{2+}$ complex. This procedure was repeated two more times changing the irradiation time to 5 and 6 seconds. Additionally, the blank solution was done with a non-irradiate sample, following the same sample treatment.

The absorbance of each solution at 510 nm was measured using a Thermo Scientific Spectronic 200 spectrophotometer, establishing the blank with the non-irradiated sample. According to Lambert-Beer law, the moles of Fe(II) in each sample are related to the absorbance:

$$n(Fe) = \frac{A \cdot V}{l \cdot \varepsilon}$$

Where:

- A is the absorbance of each sample.
- V is the volume (in L) of the measurement sample (10 mL).

- ε is the extinction coefficient of the complex $Fe^{II}(phen)_3^{2+}$ at 510 nm (11100 L mol^{-1} cm^{-1})
- l is the optical path of the sample in the spectrophotometer (1 cm).

The moles of Fe(II) were plot versus irradiation time (Figure S3):

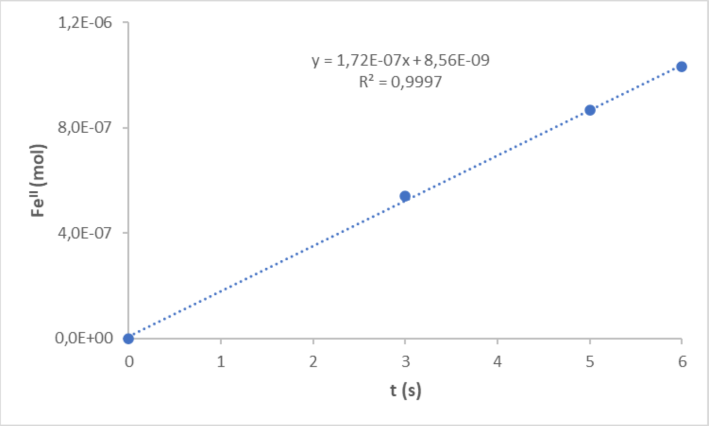

Figure S3: Moles of Fe(II) against the time for the determination of the photon flux.

The slope of the line is the rate of $Fe^{II}(phen)_3^{2+}$ formation: $1.737 \cdot 10^{-7}$ mol/s.

The photon flux can be calculated using the following equation:

$$Photon\ flux\ \left(\frac{einstein}{s}\right) = \frac{Reaction\ rate\ (\frac{mol}{s})}{Quantum\ yield\ \left(\frac{mol}{einstein}\right) \cdot F}$$

Where:

- Reaction rate is the already calculated value: $1.737 \cdot 10^{-7}$ mol/s.
- Quantum yield for the photoreduction of ferrioxalate at 455 nm, which is 1.1[19]
- F is the fraction of light absorbed, and it is calculated with $F = 1 - 10^{-A(455\ nm)} = 0.376$, where A(455 nm) is the absorbance of the actinometer solution at 450 nm (0.205).

$$Photon\ flux\ \left(\frac{einstein}{s}\right) = \frac{1.737 \cdot 10^{-7}\ \left(\frac{mol}{s}\right)}{1.1\ \left(\frac{mol}{einstein}\right) \cdot 0.376} = 4.18 \cdot 10^{-7}\ \frac{einsteins}{s}$$

2. <u>Determination of the Quantum Yield of the photochemical reaction:</u>

Once we have determined the photon flux of the HP Single Blue LED, the same equation must be employed for the determination of the quantum yield of the photochemical Giese addition. For that, the moles of Giese product for a given time must be determined.

Following General Procedure 1 (GP-1), the reaction between **1a** and **2a** in the presence of $Ru(bpy)_3Cl_2$ was carried out. After 10 minutes, a 0.2 mL aliquot was taken out and filtered through silica and eluted with EtOAc. The moles of product **3a** were determined by ^{1}H-NMR using 4-methoxyacetophenone as internal standard. This process was

repeated taking aliquots after 20 and 30 minutes of irradiance. The kinetic profile of the reaction is shown in Figure S4.

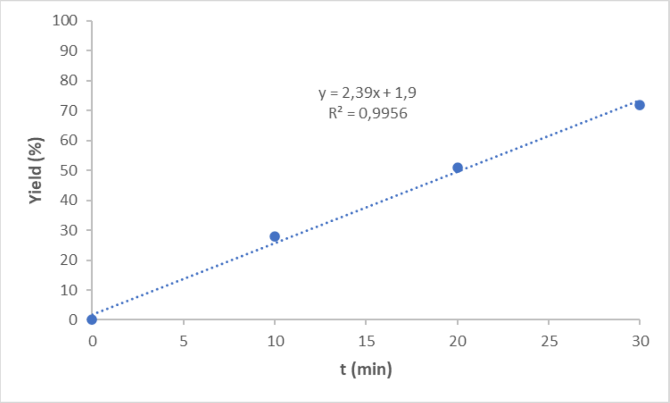

Figure S4: Kinetic profile for the photochemical reaction after 10, 20 and 30 minutes of irradiance

Then, the following equation was used to determine the quantum yield:

$$Quantum\ yield\ \left(\frac{mol}{einstein}\right) = \frac{\mathbf{3}\ (mol)}{Photon\ flux\ \left(\frac{einstein}{s}\right) \cdot time\ (s) \cdot F}$$

Where:

- **3a** (mol) is the amount of **3a** that has been formed during the irradiation time.
- Time (s) is the irradiation time (in seconds).
- F is the fraction of light absorbed, and it is calculated with $F = 1 - 10^{-A(450\ nm)} = 0.9941$, where A(450 nm) is the absorbance of the reaction at 450 nm (2.23).

Run	Irradiation Time (min)	Quantum Yield
1	10	0.2226
2	20	0.2060
3	30	0.1925
		**Avg.**: 0.21±0.02

Table S2: Quantum yield determination to different irradiation times.

4.On/Off experiments:

Following General Procedure 1 (GP-1), the reaction between **1a** and **2a** in the presence of Ru(bpy)3Cl2 was carried out. After 10 minutes of irradiance, a 0.2 mL aliquot was taken out and filtered through silica and eluted with EtOAc. Then, the reaction mixture was stirred in the dark for 10 additional minutes. This process is repeated two more times. The moles of product 3a were determined by ^{1}H-NMR using 4-methoxyacetophenone as internal standard. Then, the yield of the reaction is plotted for each time (Figure S5).

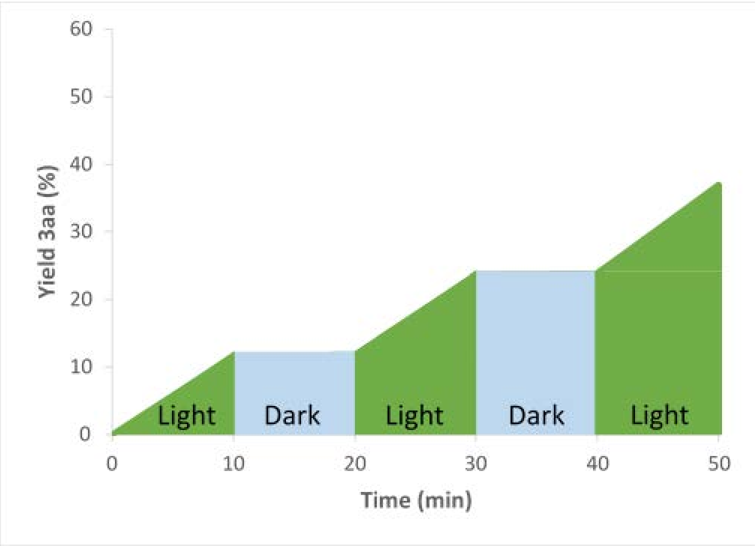

*Figure S5: On/off experiment for the radical addition reaction between dihydroquinoxalin-2-one **1a** and trifluoroacetophenone **2a**.*

Typical reaction setup for the 0.2 mmol scale reaction

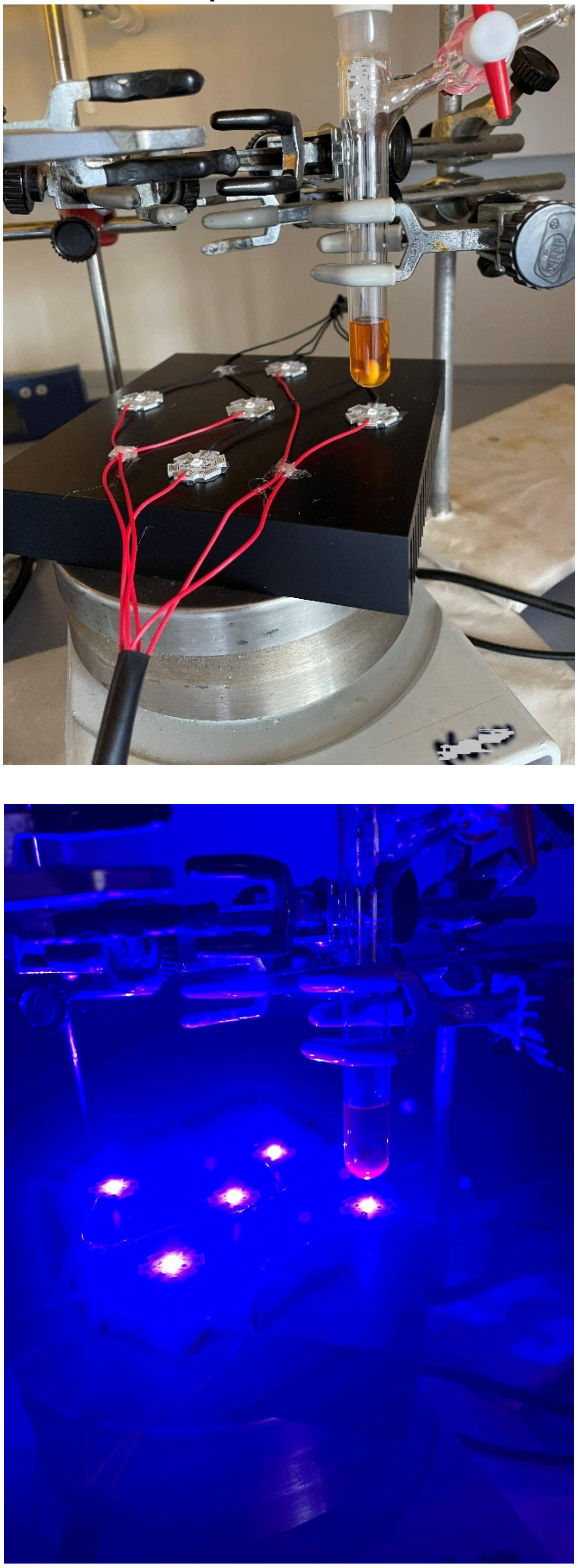

Figure S6: Typical reaction setup for 0.2 mmol-scale additions.

NMR Spectra

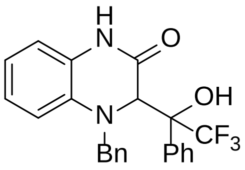

3aa'

^{1}H-NMR ($CDCl_3$, 300 MHz)
^{19}F{^{1}H}-NMR ($CDCl_3$, 282 MHz)
^{13}C{^{1}H}-NMR ($CDCl_3$, 75 MHz)

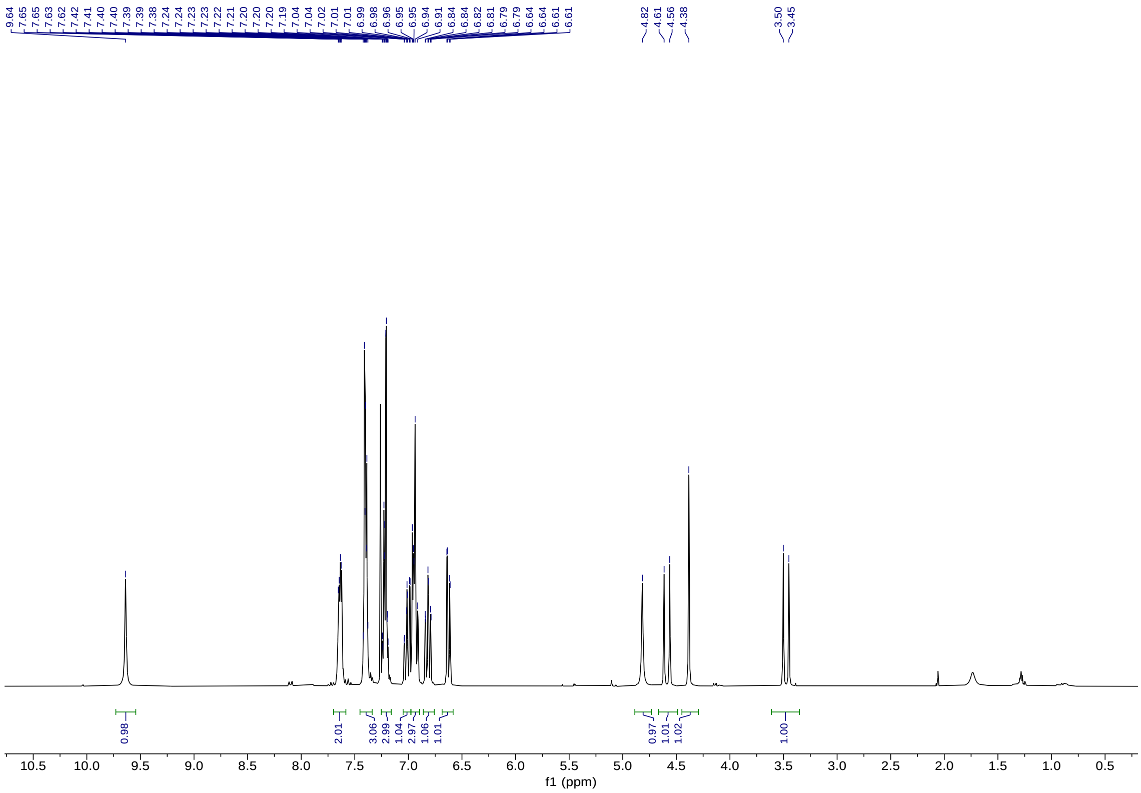

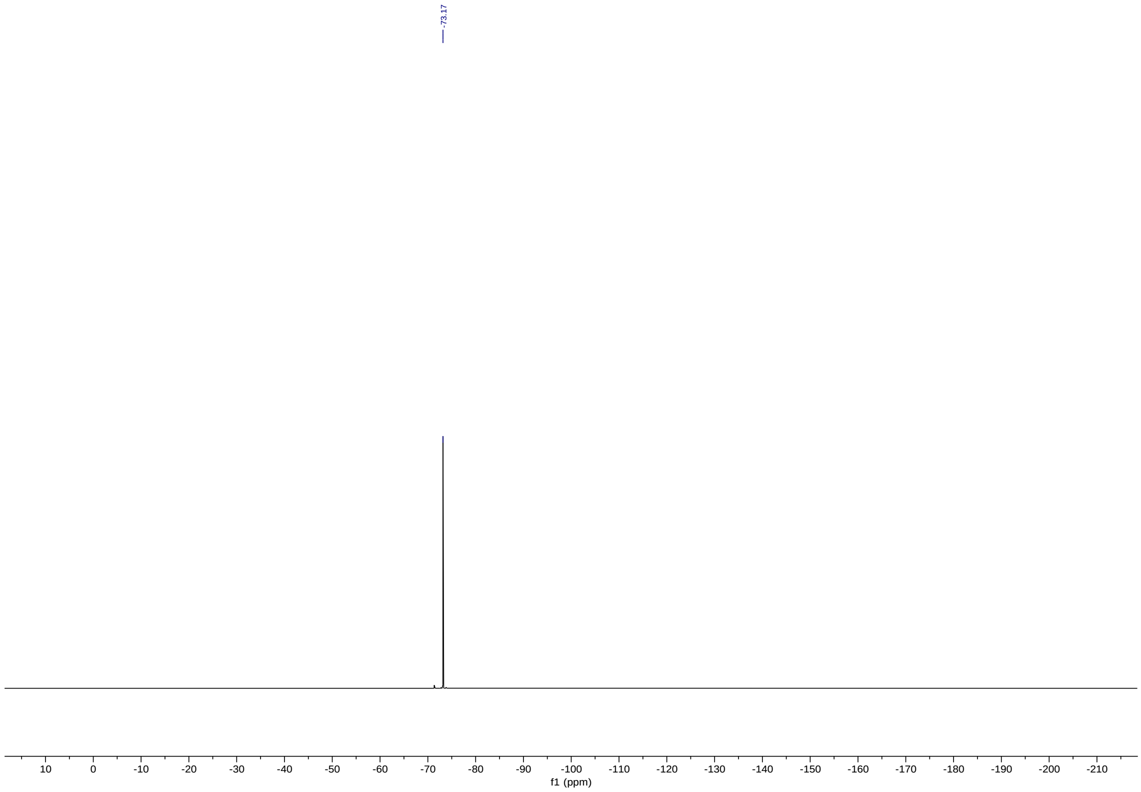

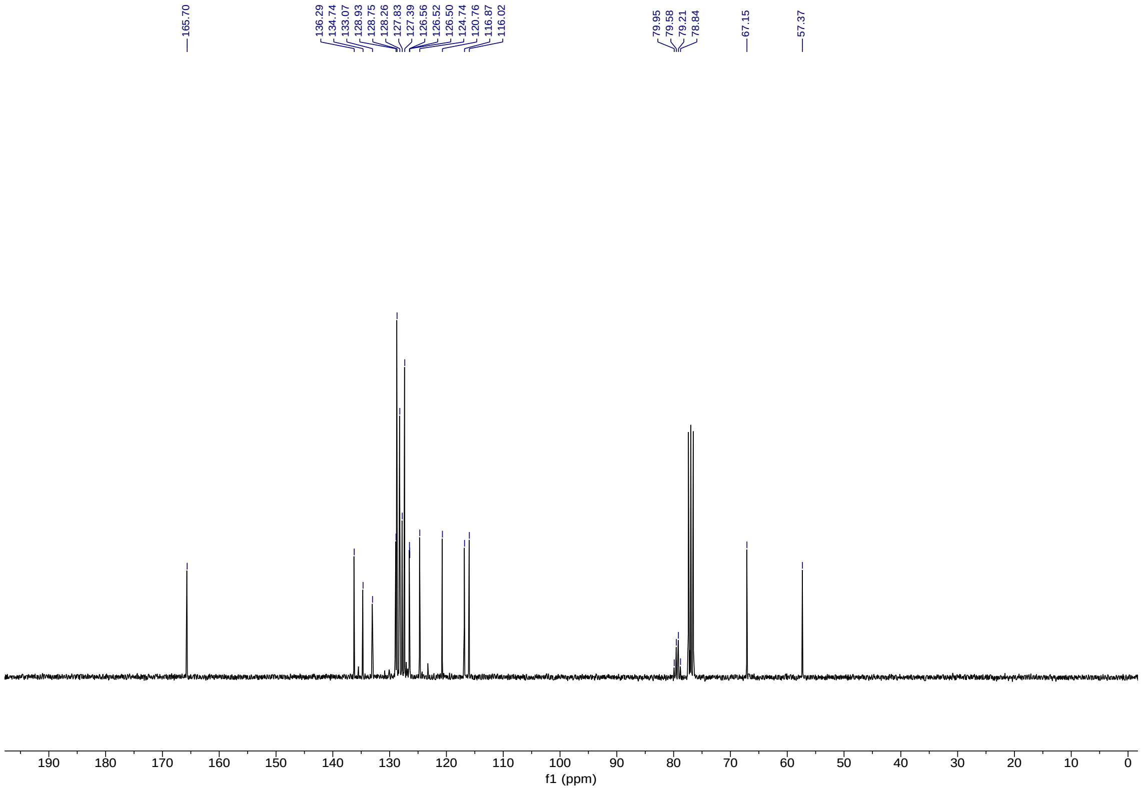
165.70
136.29
134.74
133.07
128.93
128.75
128.26
127.83
127.39
126.56
126.52
126.50
124.74
120.76
116.87
116.02
79.95
79.58
79.21
78.84
67.15
57.37
190 180 170 160 150 140 130 120 110 100 90 80 70 60 50 40 30 20 10 0
f1 (ppm)

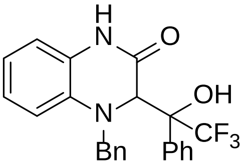

3aa''

^{1}H-NMR ($CDCl_3$, 300 MHz)
^{19}F{^{1}H}-NMR ($CDCl_3$, 282 MHz)
^{13}C{^{1}H}-NMR ($CDCl_3$, 75 MHz)

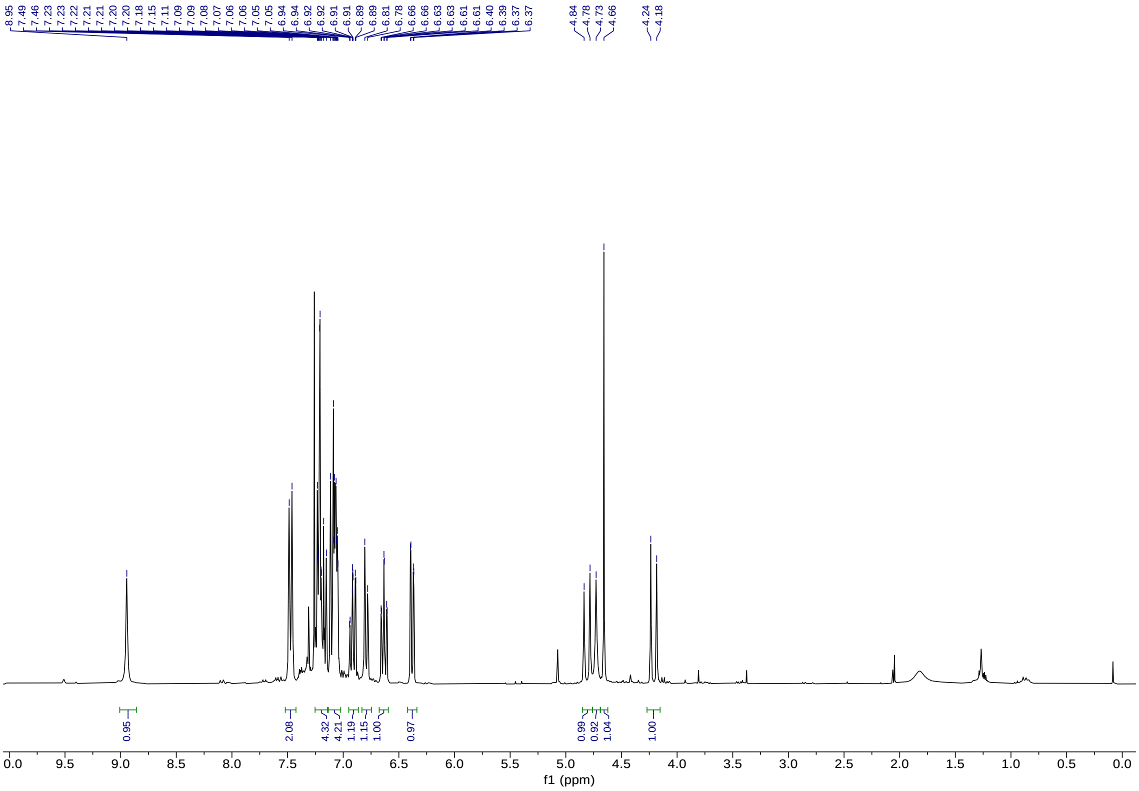

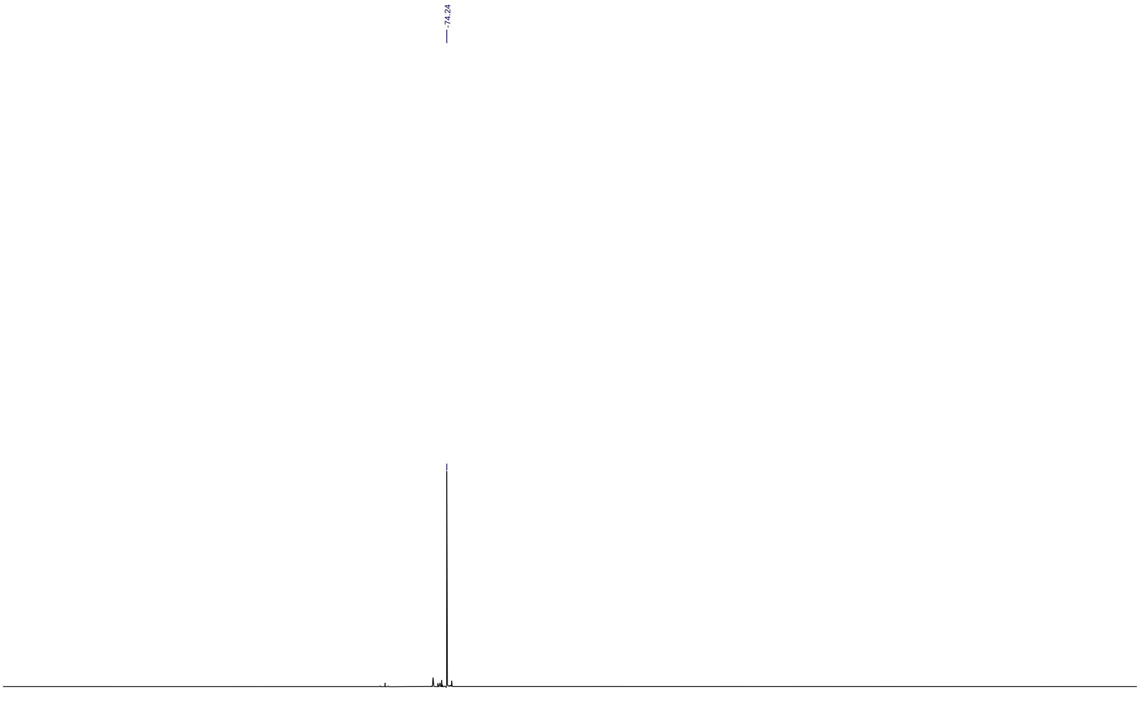

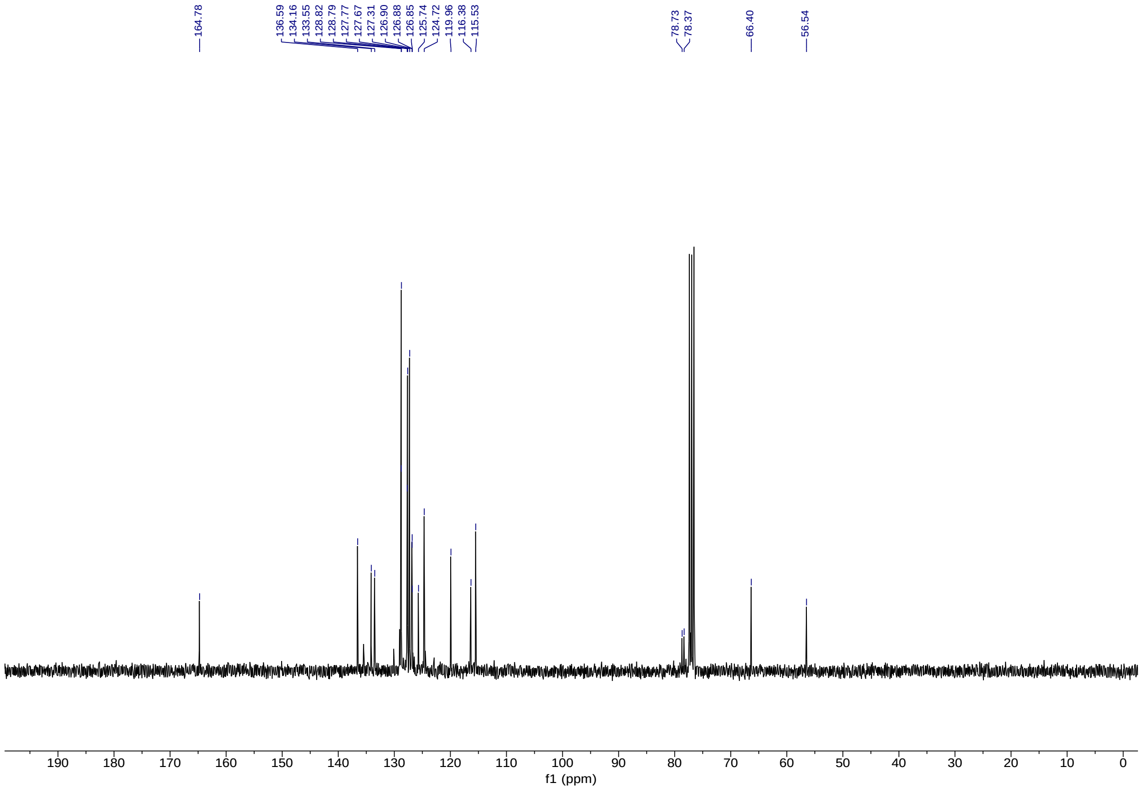
164.78
136.59
134.16
133.55
128.82
128.79
127.77
127.67
127.31
126.90
126.88
126.85
125.74
124.72
119.96
116.38
115.53
78.73
78.37
66.40
56.54
190 180 170 160 150 140 130 120 110 100 90 80 70 60 50 40 30 20 10 0
f1 (ppm)

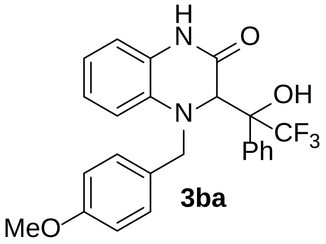

3ba

^{1}H-NMR ($CDCl_3$, 300 MHz)
^{19}F{^{1}H}-NMR ($CDCl_3$, 282 MHz)
^{13}C{^{1}H}-NMR ($CDCl_3$, 75 MHz)

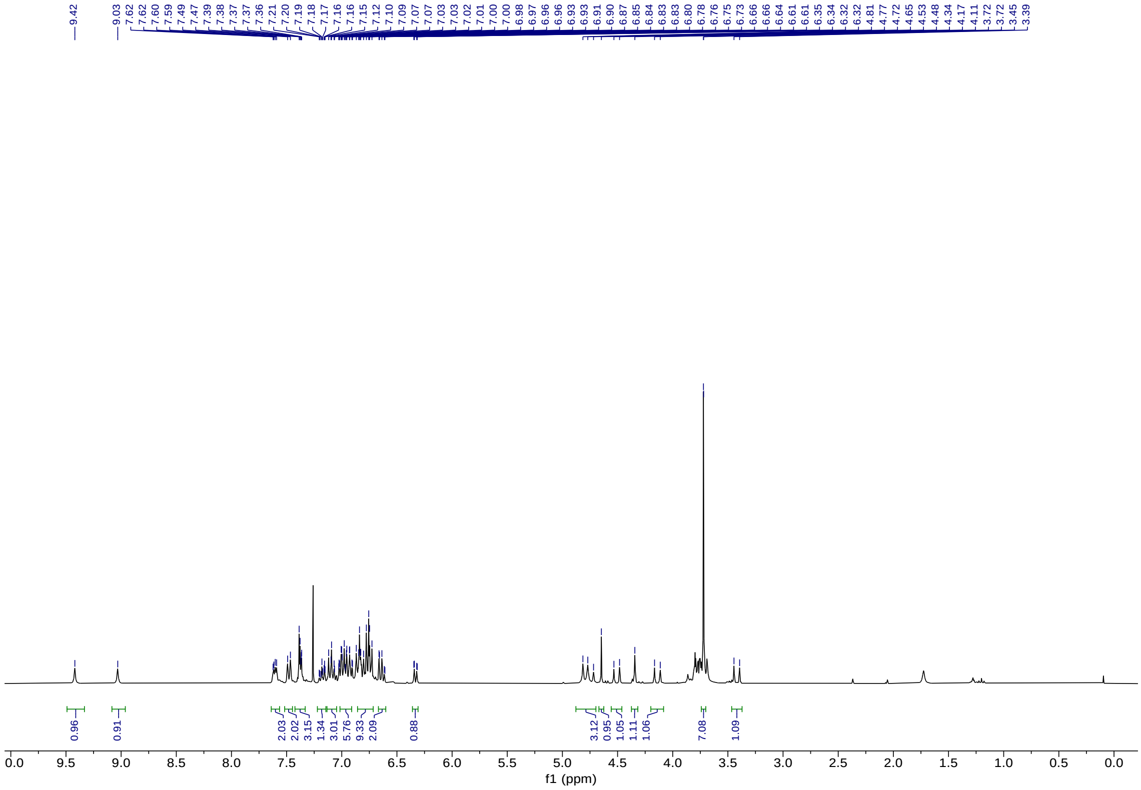

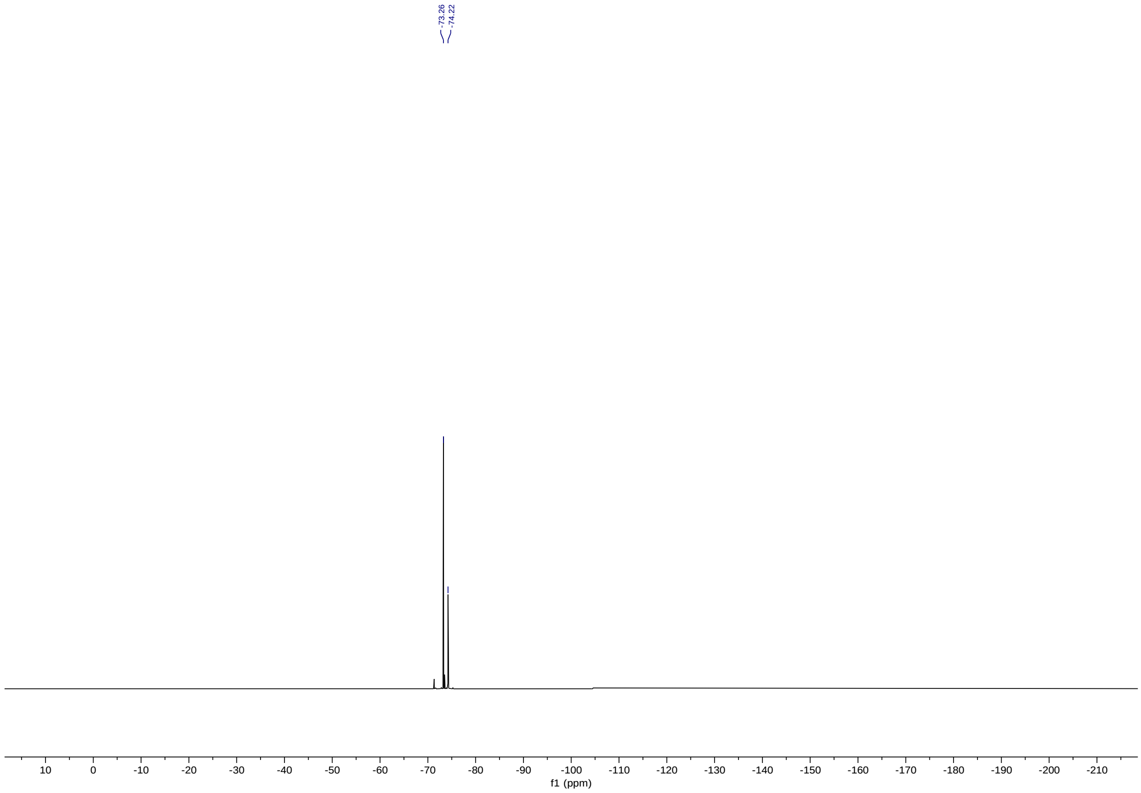

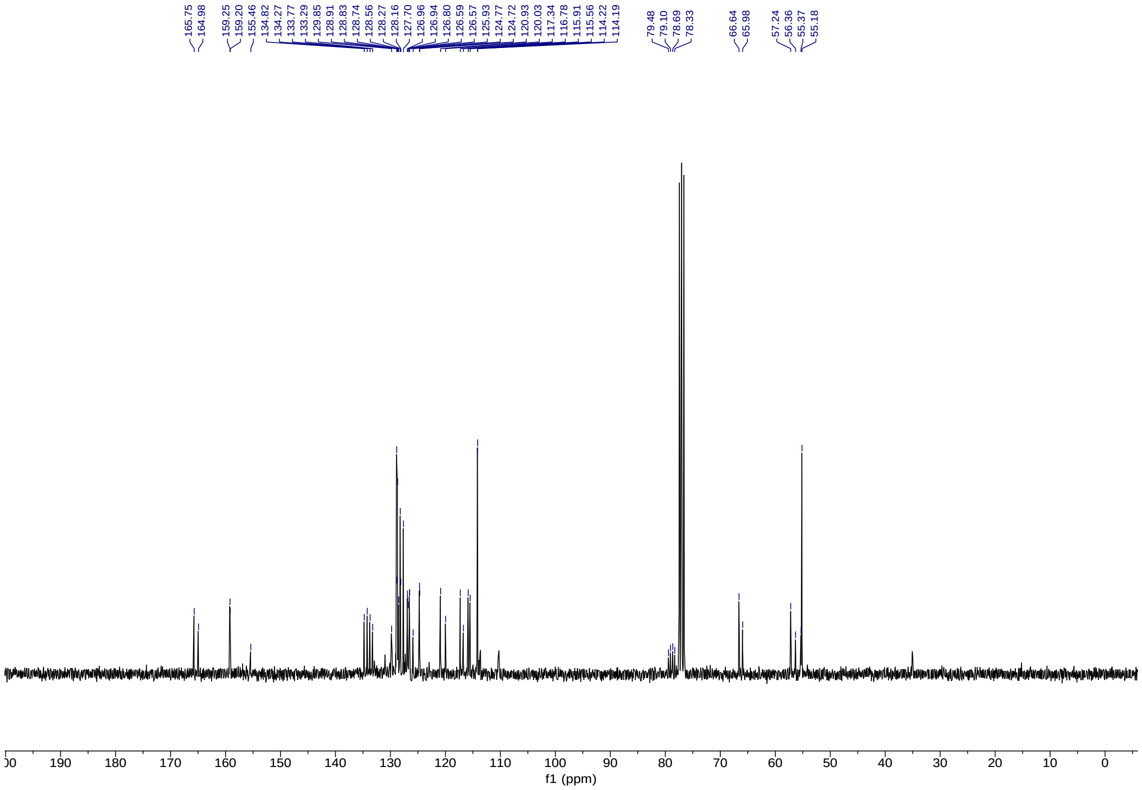
165.75
164.98
159.25
159.20
155.46
134.82
134.27
133.77
133.29
129.85
128.91
128.83
128.74
128.56
128.27
128.16
127.70
126.96
126.94
126.80
126.59
126.57
125.93
124.77
124.72
120.93
120.03
117.34
116.78
115.91
115.56
114.22
114.19
79.48
79.10
78.69
78.33
66.64
65.98
57.24
56.36
55.37
55.18
f1 (ppm)

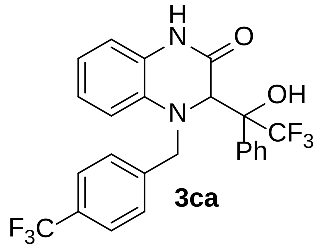

3ca

^{1}H-NMR ($CDCl_3$, 300 MHz)
^{19}F{^{1}H}-NMR ($CDCl_3$, 282 MHz)
^{13}C{^{1}H}-NMR ($CDCl_3$, 75 MHz)

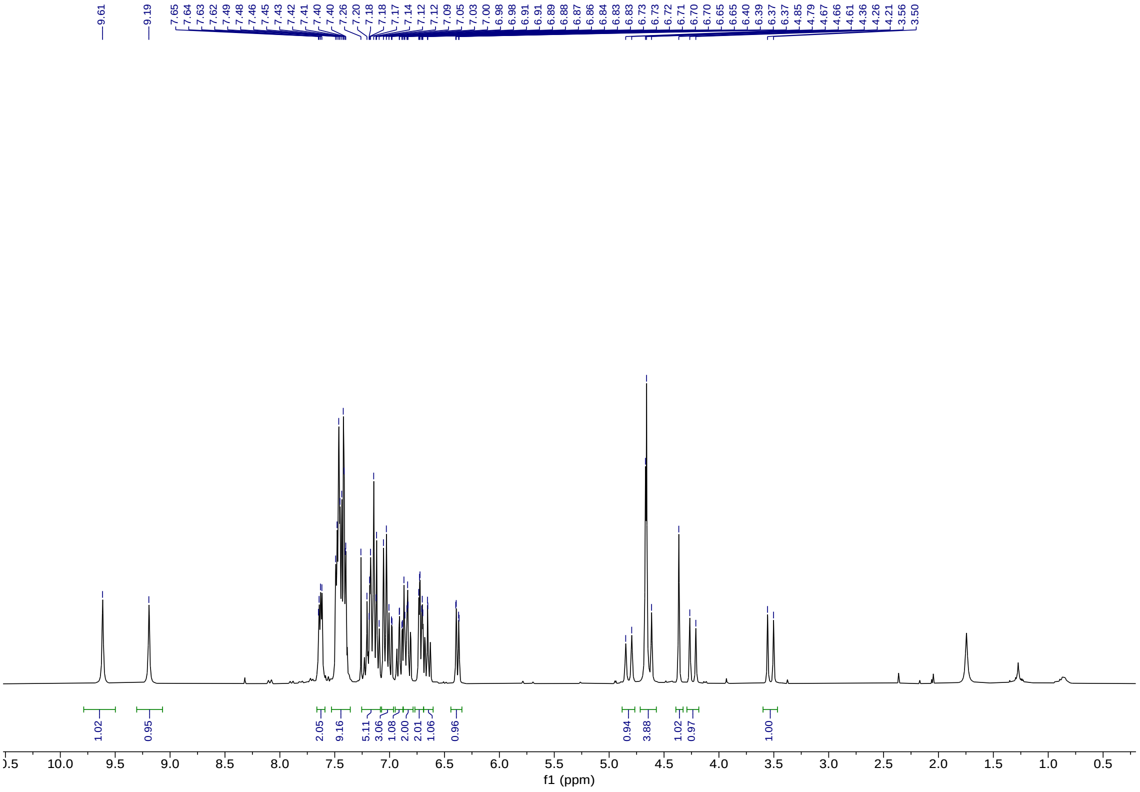

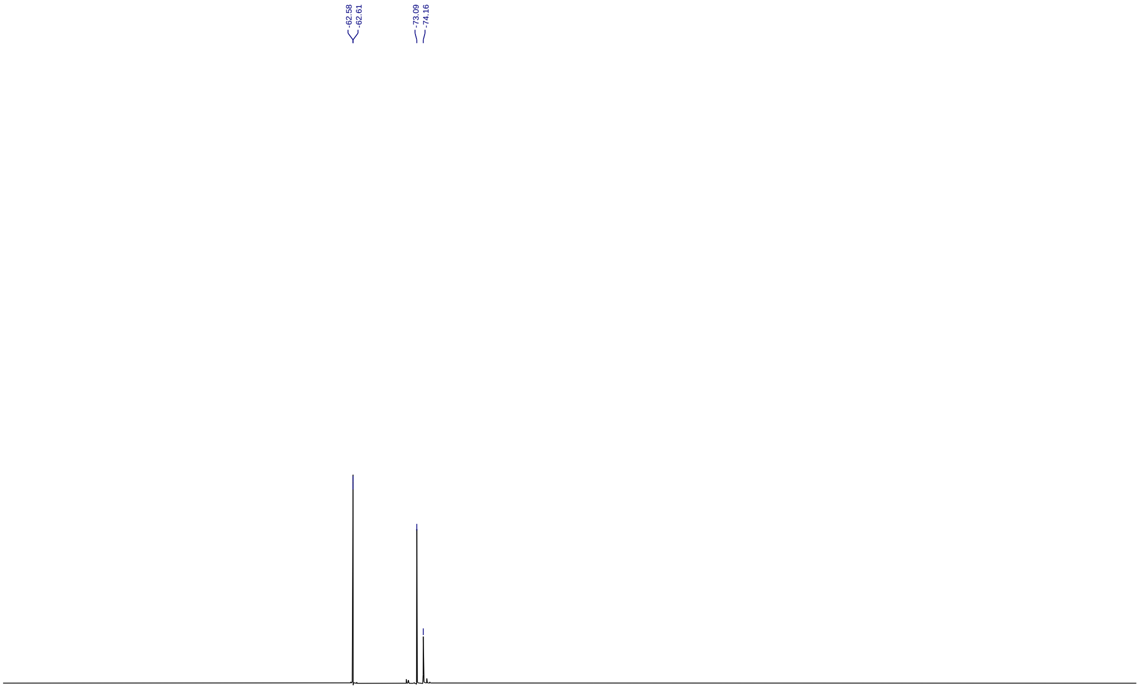

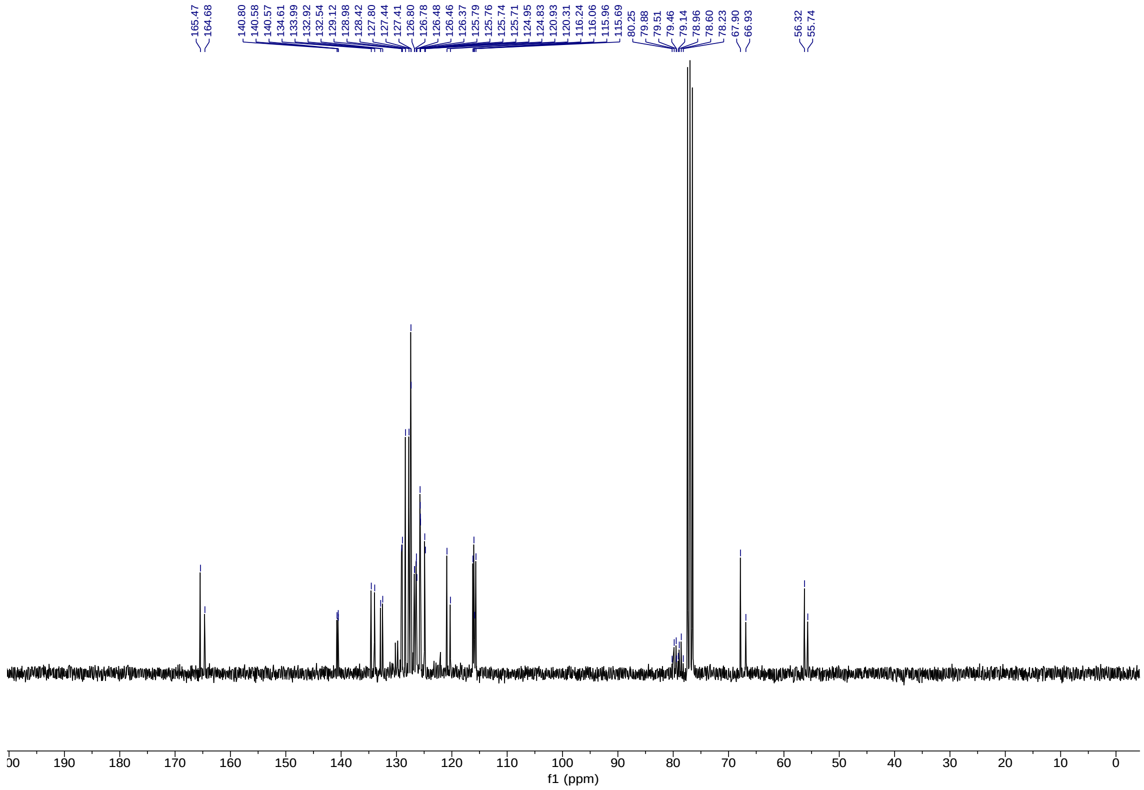
165.47
164.68
140.80
140.58
140.57
134.61
133.99
132.92
132.54
129.12
128.98
128.42
127.80
127.44
127.41
126.80
126.78
126.48
126.46
126.37
125.79
125.76
125.74
125.71
124.95
124.83
120.93
120.31
116.24
116.06
115.96
115.69
80.25
79.88
79.51
79.46
79.14
78.96
78.60
78.23
67.90
66.93
56.32
55.74
f1 (ppm)

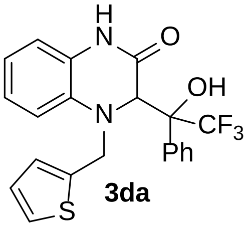

3da

^{1}H-NMR ($CDCl_3$, 300 MHz)
^{19}F{^{1}H} ($CDCl_3$, 282 MHz)
^{13}C{^{1}H}-NMR ($CDCl_3$, 75 MHz)

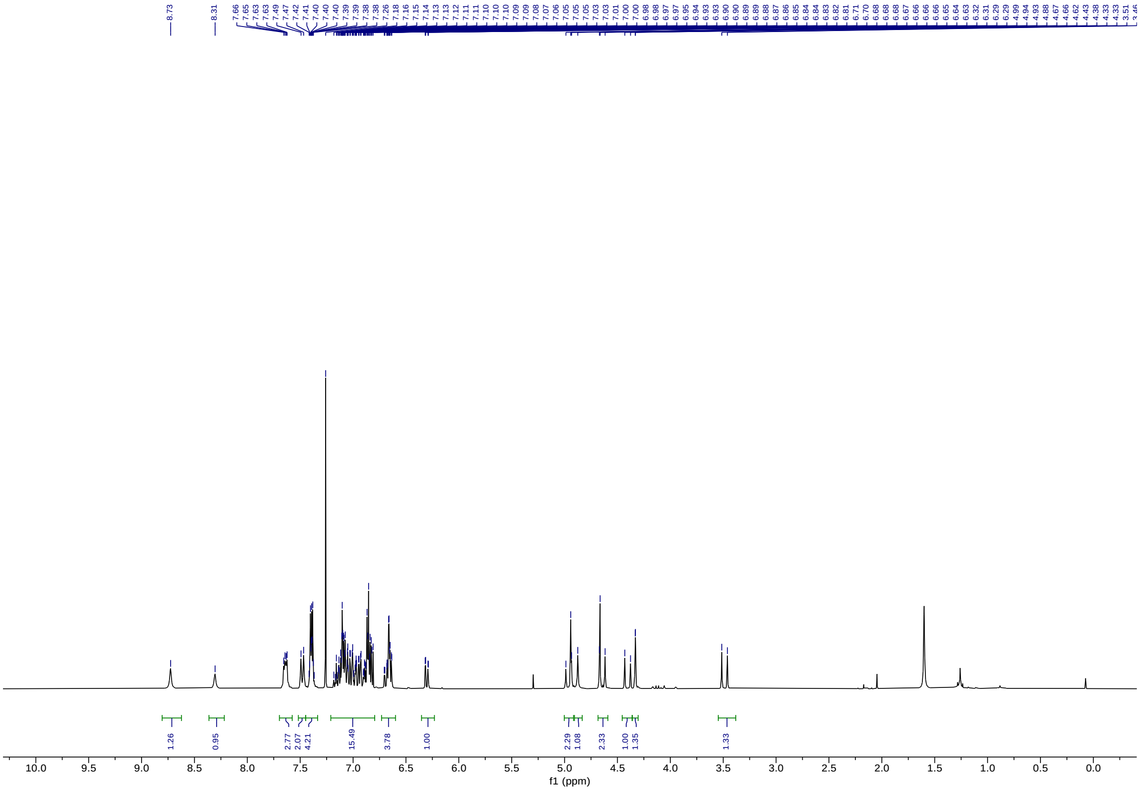

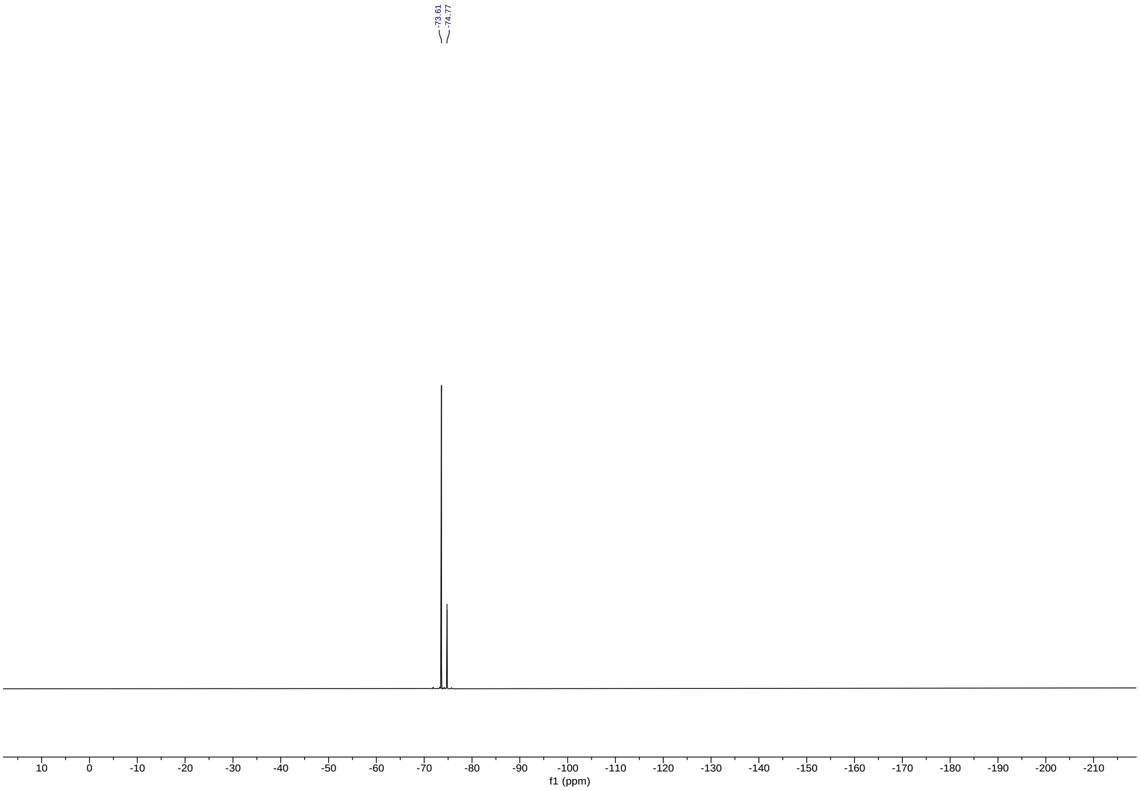

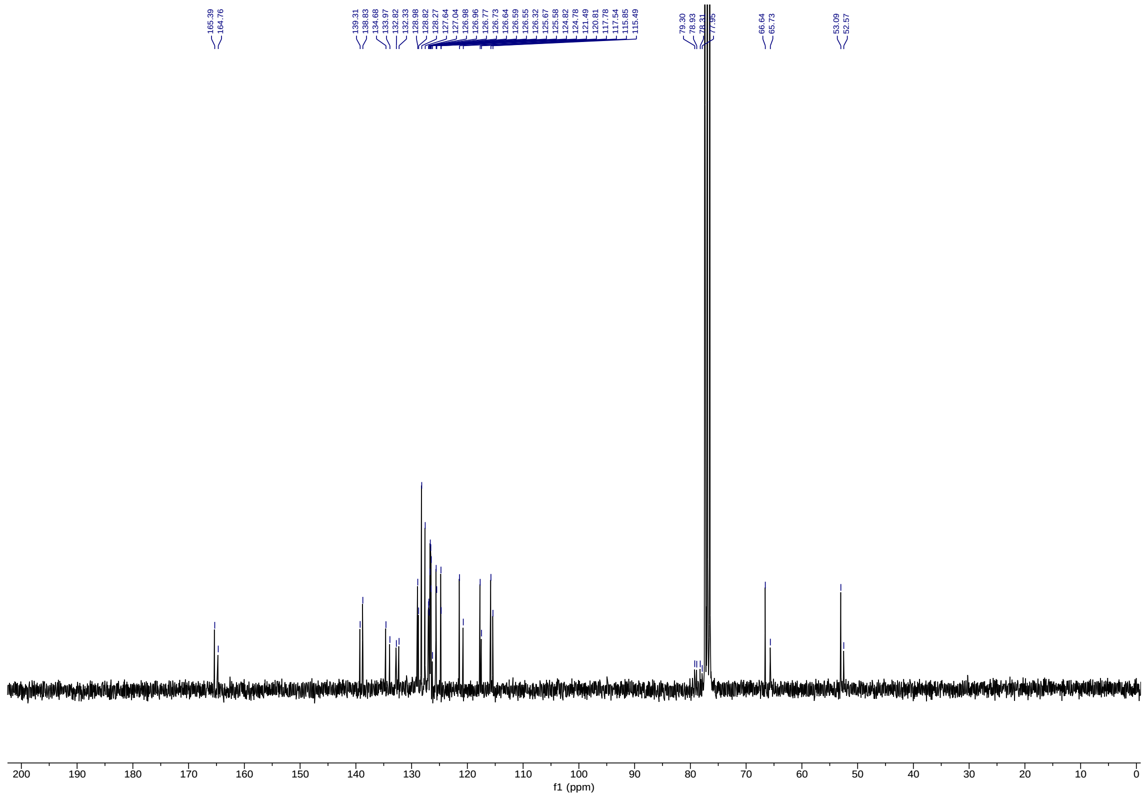

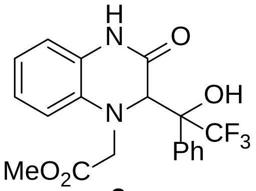

3ea

^{1}H-NMR ($CDCl_3$, 300 MHz)
^{19}F{^{1}H}-NMR ($CDCl_3$, 282 MHz)
^{13}C{^{1}H}-NMR ($CDCl_3$, 75 MHz)

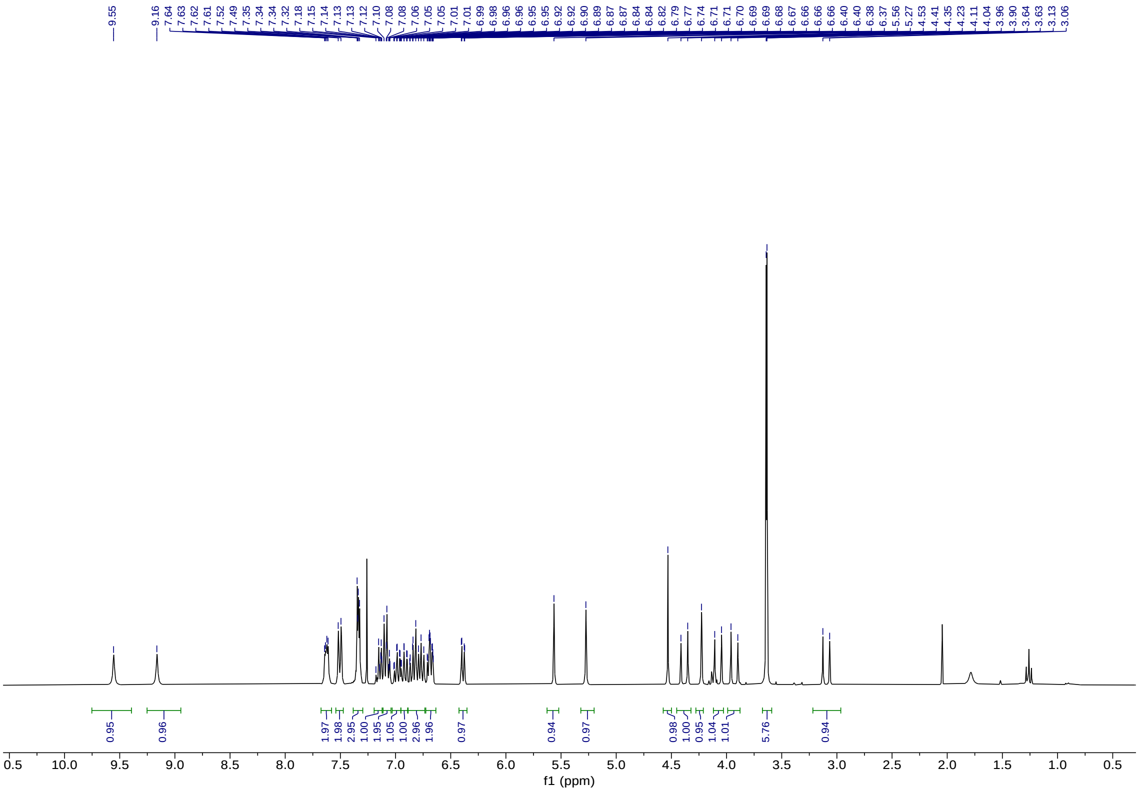

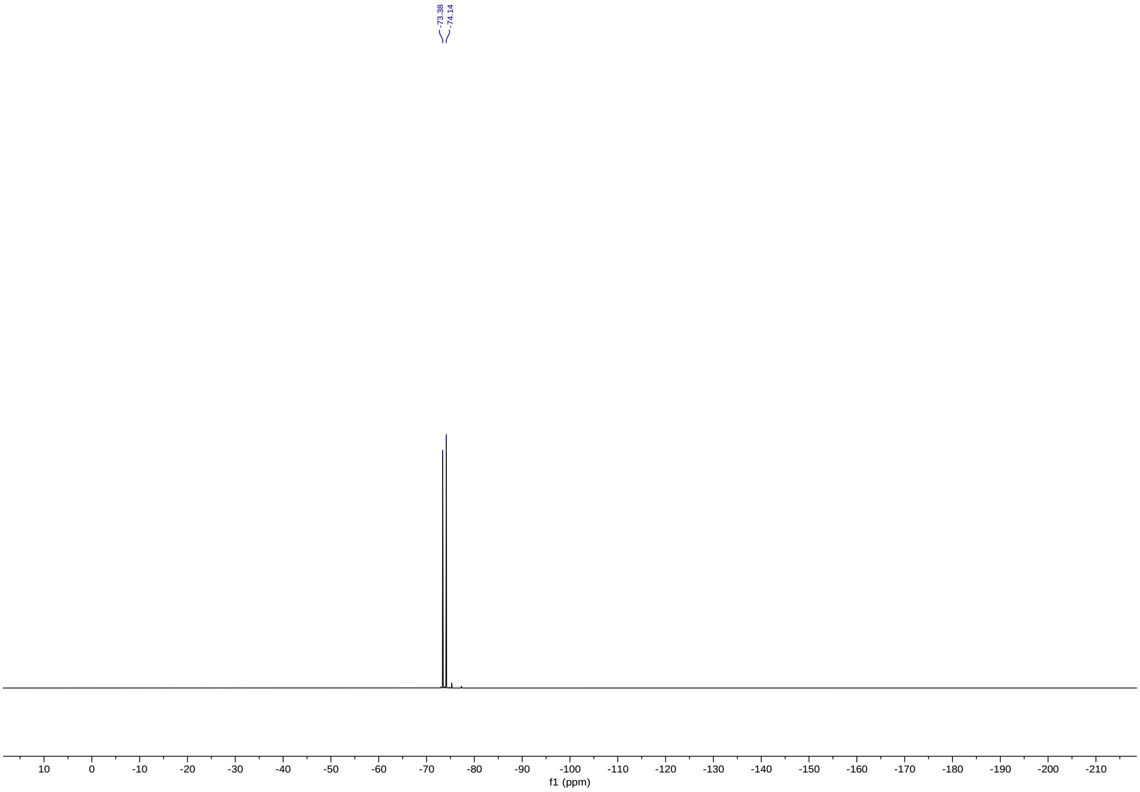

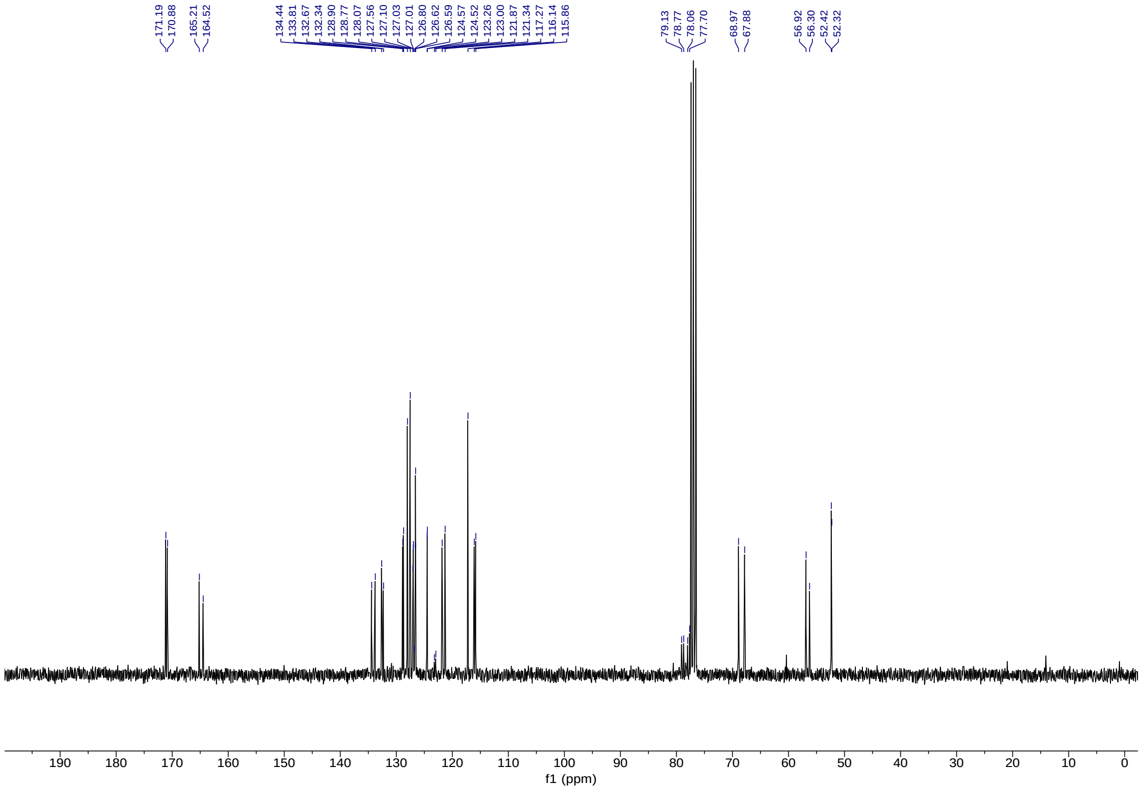
171.19
170.88
165.21
164.52
134.44
133.81
132.67
132.34
128.90
128.77
128.07
127.56
127.10
127.03
127.01
126.80
126.62
126.59
124.57
124.52
123.26
123.00
121.87
121.34
117.27
116.14
115.86
79.13
78.77
78.06
77.70
68.97
67.88
56.92
56.30
52.42
52.32
190
180
170
160
150
140
130
120
110
100
90
80
70
60
50
40
30
20
10
0
f1 (ppm)

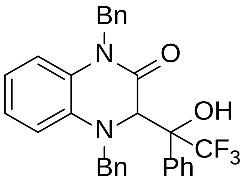

3fa

^{1}H-NMR ($CDCl_3$, 300 MHz)
^{19}F{^{1}H}-NMR ($CDCl_3$, 282 MHz)
^{13}C{^{1}H}-NMR ($CDCl_3$, 75 MHz)

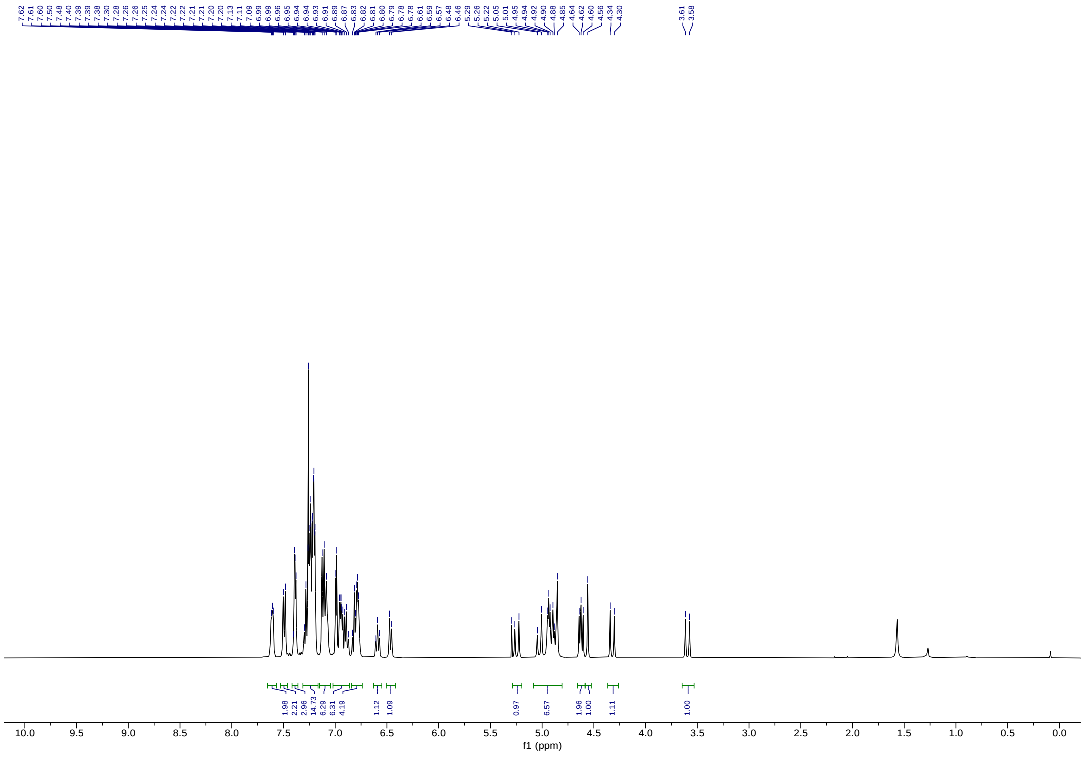

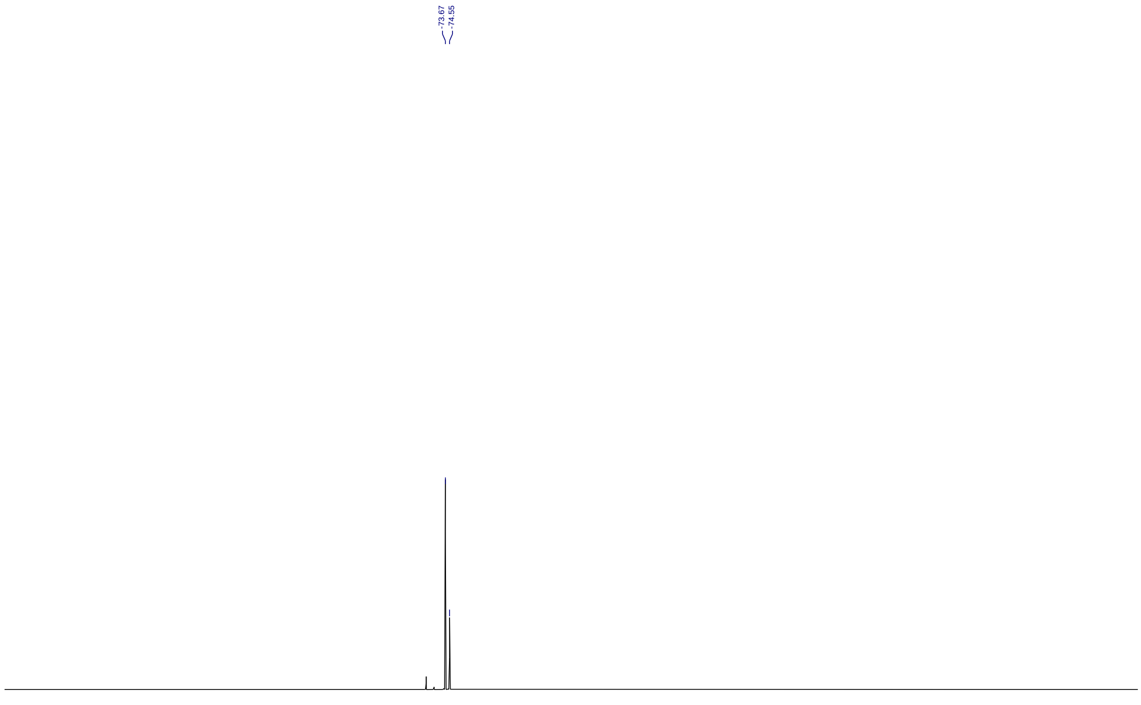

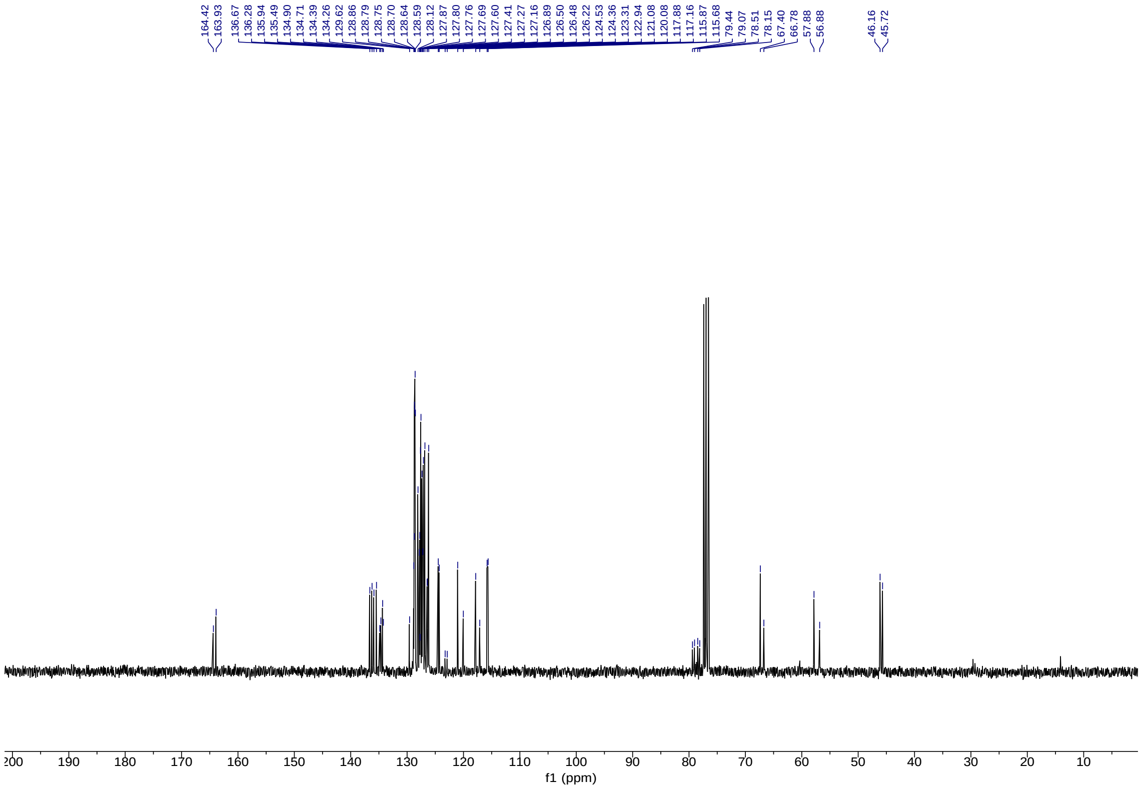

164.42
163.93
136.67
136.28
135.94
135.49
134.90
134.71
134.39
134.26
129.62
128.86
128.79
128.75
128.70
128.64
128.59
128.12
127.87
127.80
127.76
127.69
127.60
127.41
127.27
127.16
126.89
126.50
126.48
126.22
124.53
124.36
123.31
122.94
121.08
120.08
117.88
117.16
115.87
115.68
79.44
79.07
78.51
78.15
67.40
66.78
57.88
56.88
46.16
45.72
200 190 180 170 160 150 140 130 120 110 100 90 80 70 60 50 40 30 20 10
f1 (ppm)

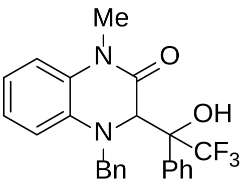

3ga

^{1}H-NMR ($CDCl_3$, 300 MHz)
^{19}F{^{1}H}-NMR ($CDCl_3$, 282 MHz)
^{13}C{^{1}H}-NMR ($CDCl_3$, 75 MHz)

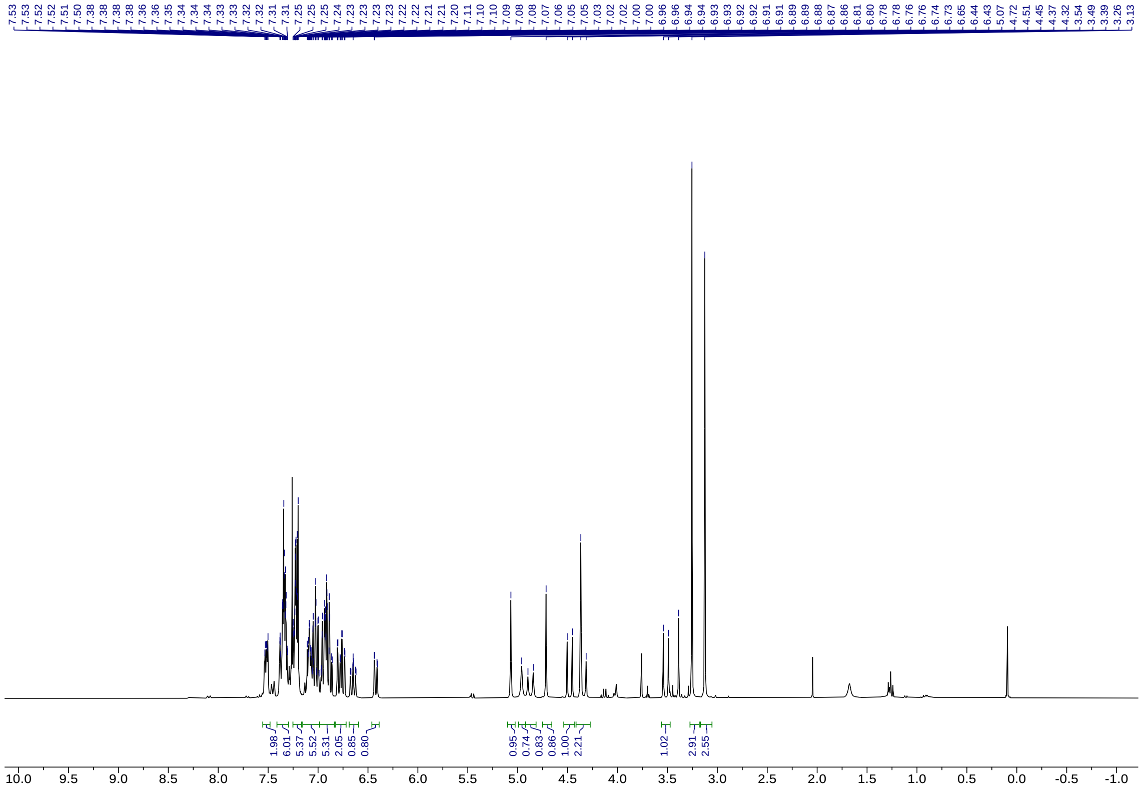

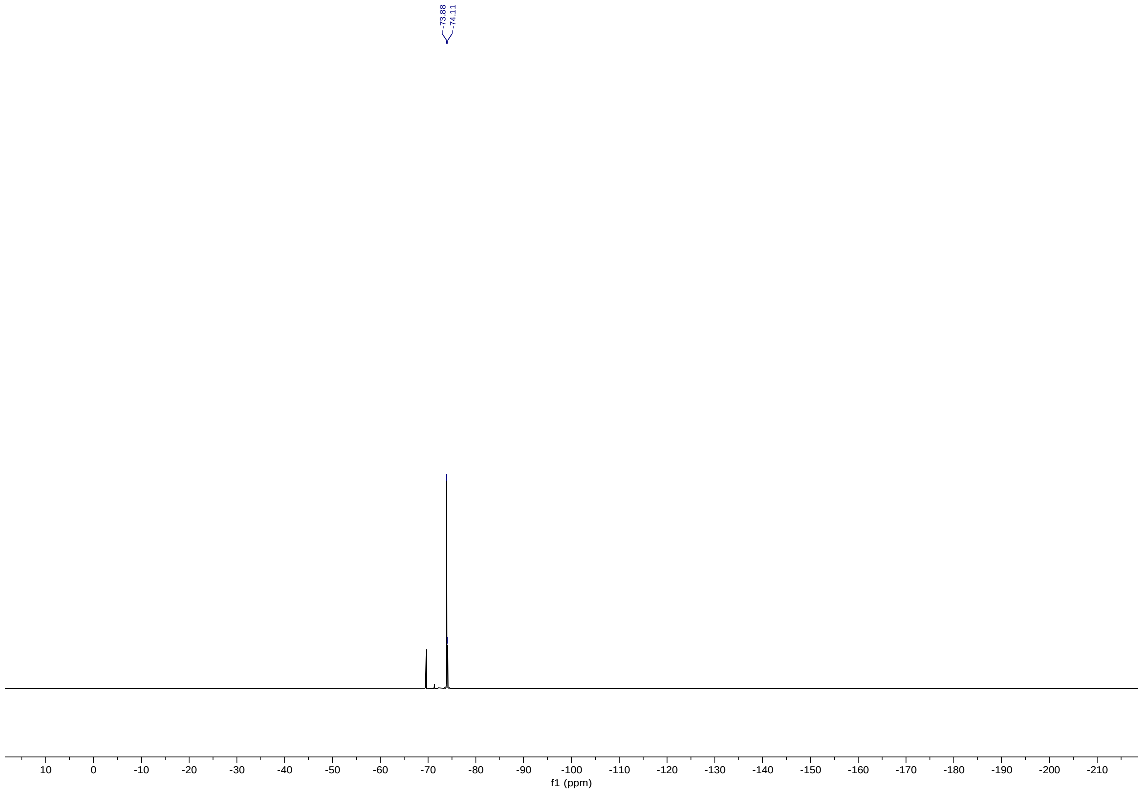

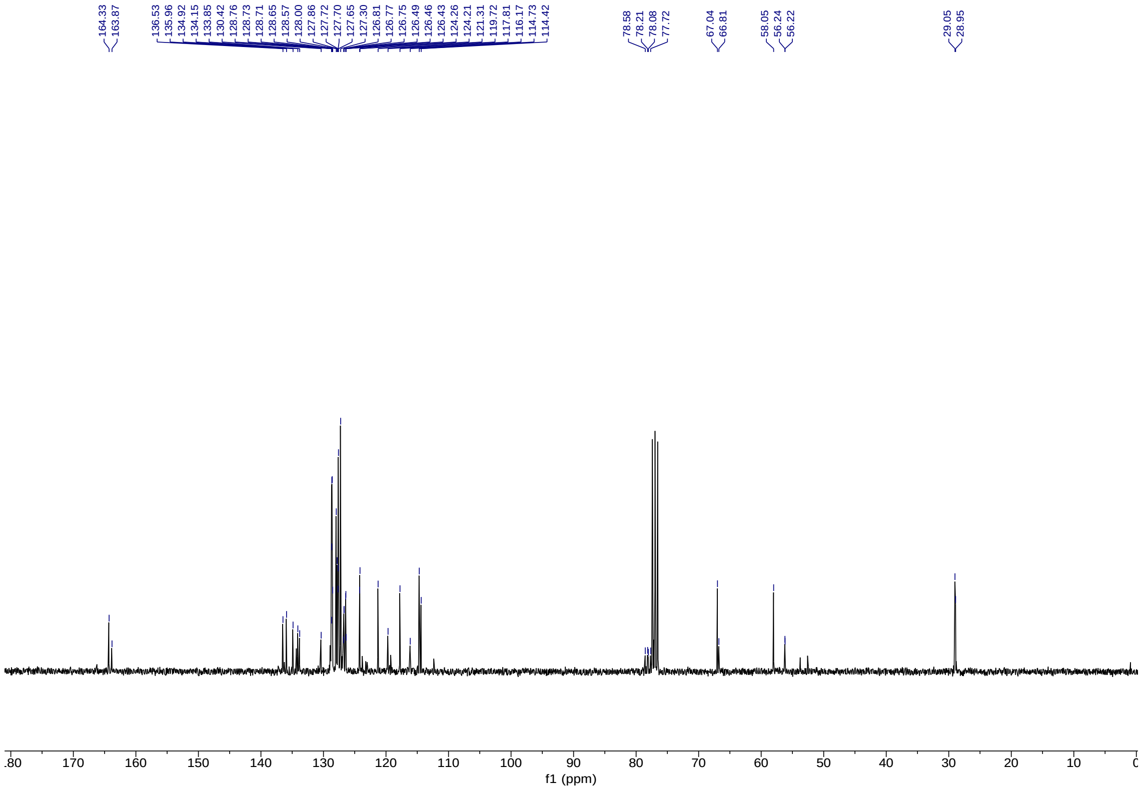
164.33
163.87
136.53
135.96
134.92
134.15
133.85
130.42
128.76
128.73
128.71
128.65
128.57
128.00
127.86
127.72
127.70
127.65
127.30
126.81
126.77
126.75
126.49
126.46
126.43
124.26
124.21
121.31
119.72
117.81
116.17
114.73
114.42
78.58
78.21
78.08
77.72
67.04
66.81
58.05
56.24
56.22
29.05
28.95
f1 (ppm)

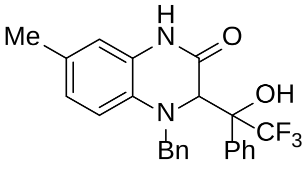

3ha'

^{1}H-NMR ($CDCl_3$, 300 MHz)
^{19}F{^{1}H}-NMR ($CDCl_3$, 282 MHz)
^{13}C{^{1}H}-NMR ($CDCl_3$, 75 MHz)

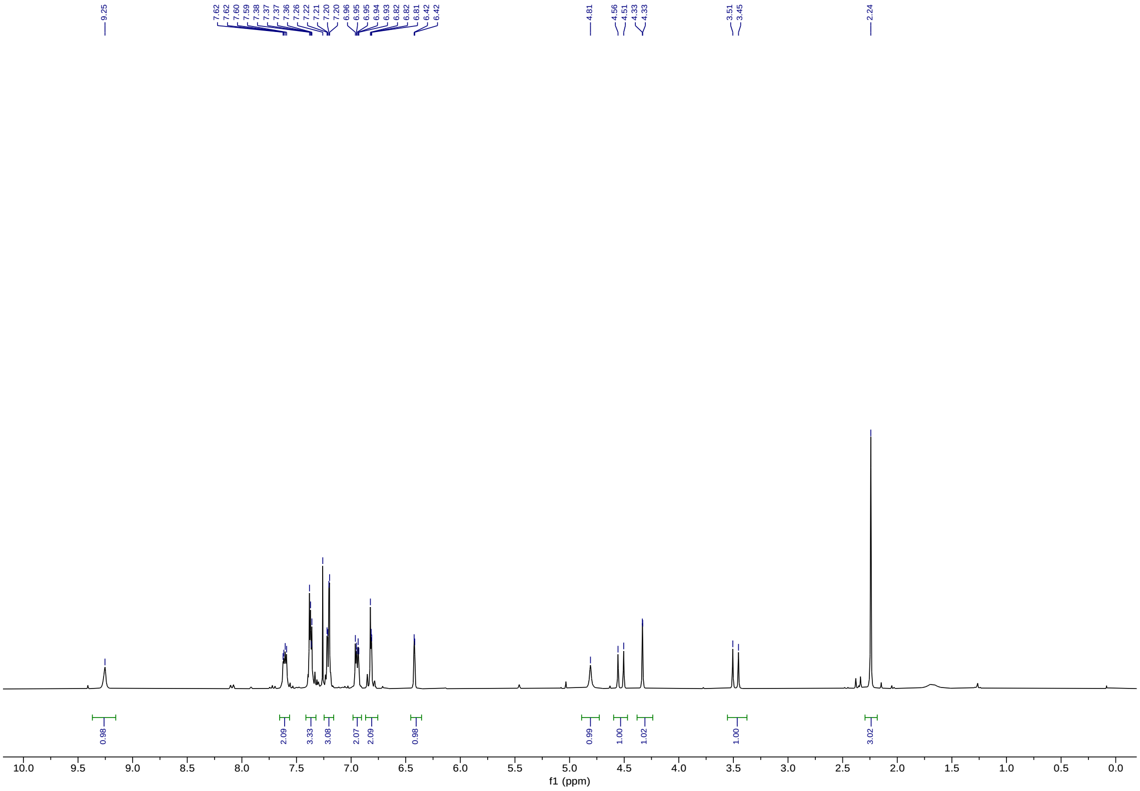

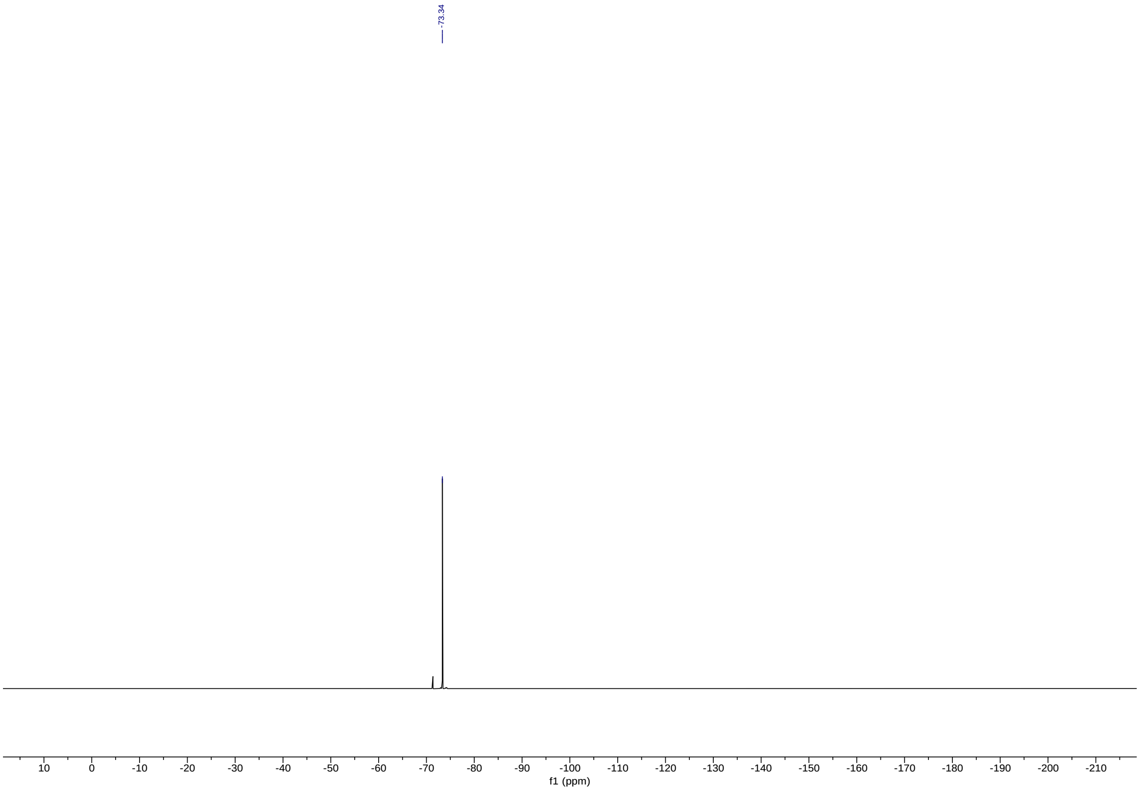

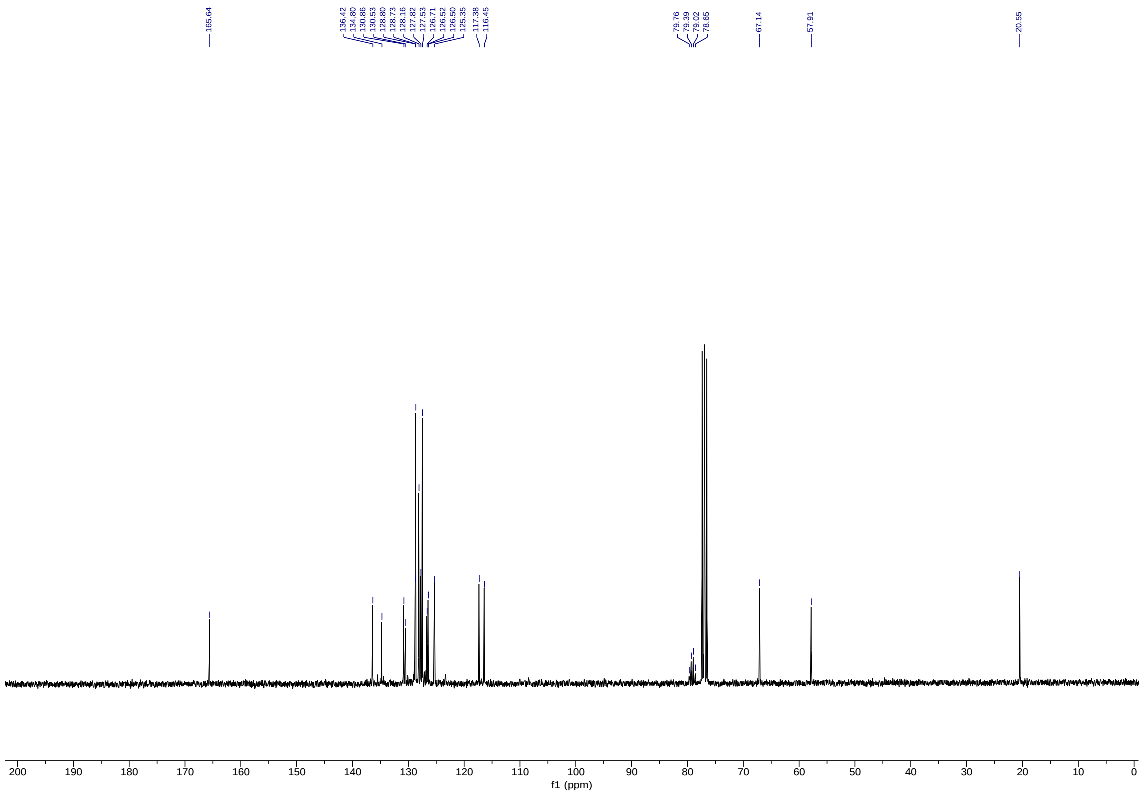
165.64
136.42
134.80
130.86
130.53
128.80
128.73
128.16
127.82
127.53
126.71
126.52
126.50
125.35
117.38
116.45
79.76
79.39
79.02
78.65
67.14
57.91
20.55
200 190 180 170 160 150 140 130 120 110 100 90 80 70 60 50 40 30 20 10 0
f1 (ppm)

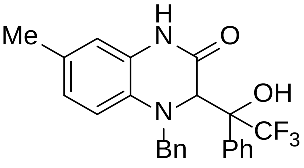

3ha''

^{1}H-NMR ($CDCl_3$, 300 MHz)
^{19}F{^{1}H}-NMR ($CDCl_3$, 282 MHz)
^{13}C{^{1}H}-NMR ($CDCl_3$, 75 MHz)

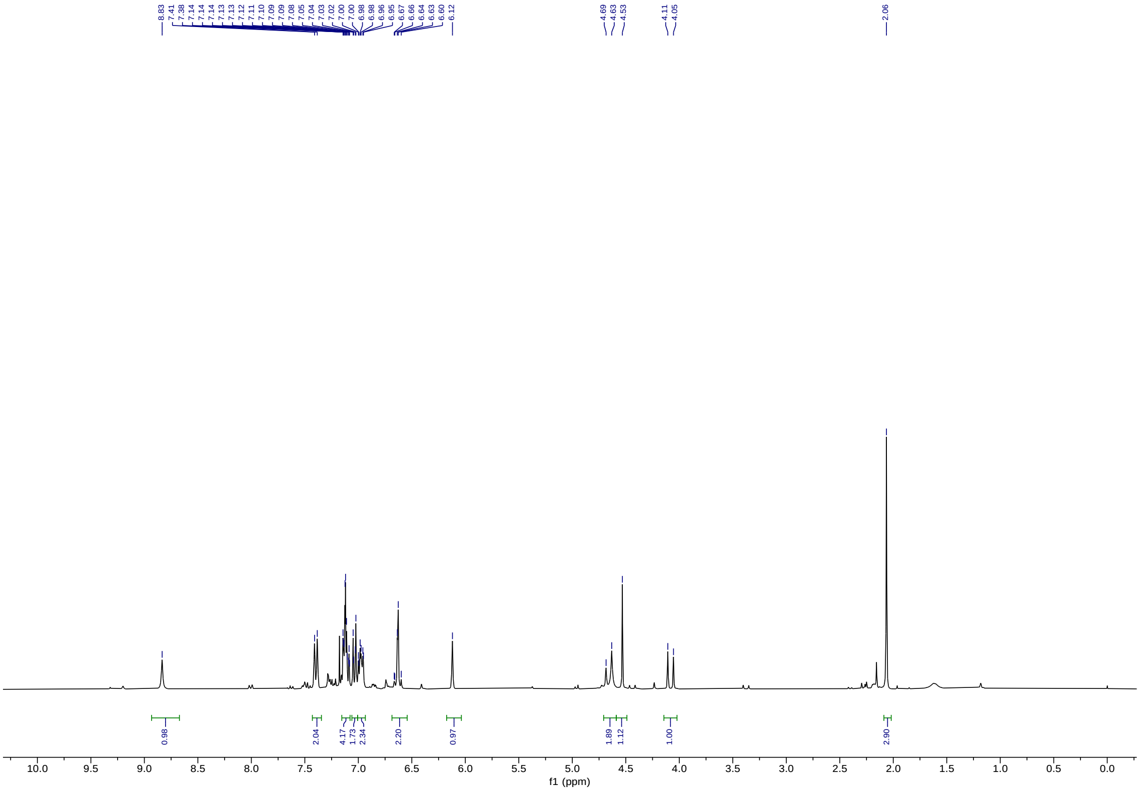

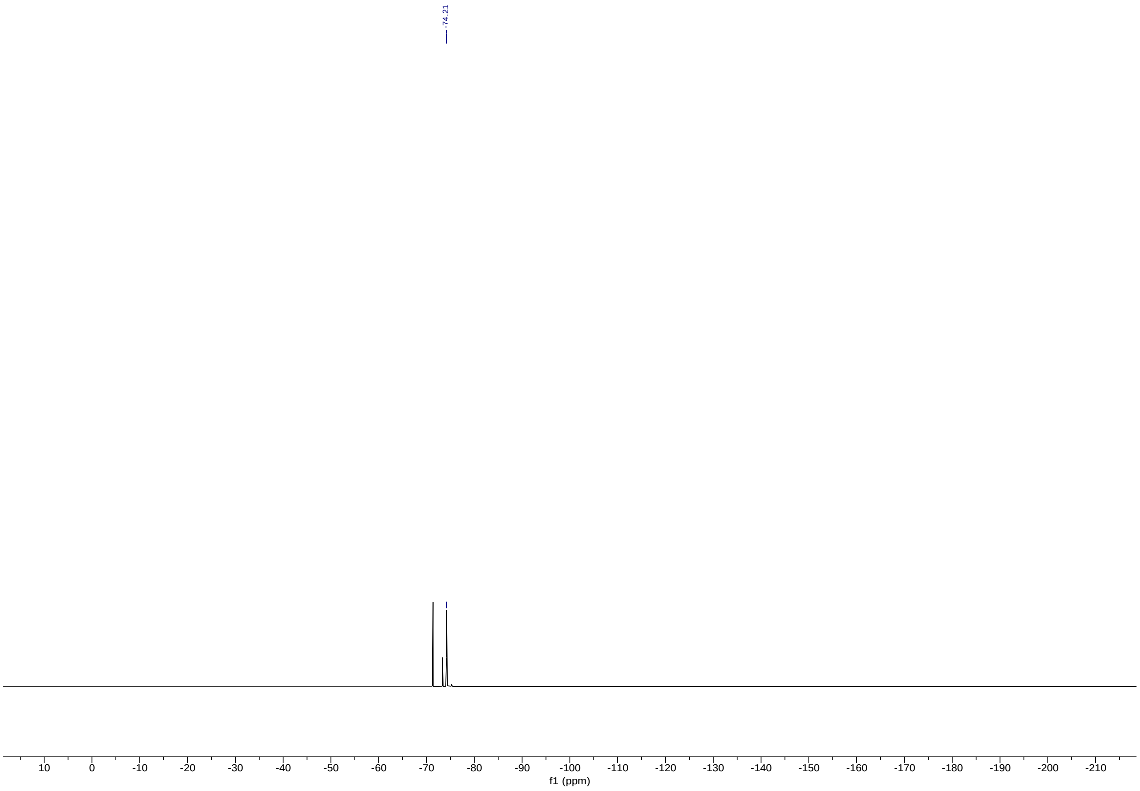

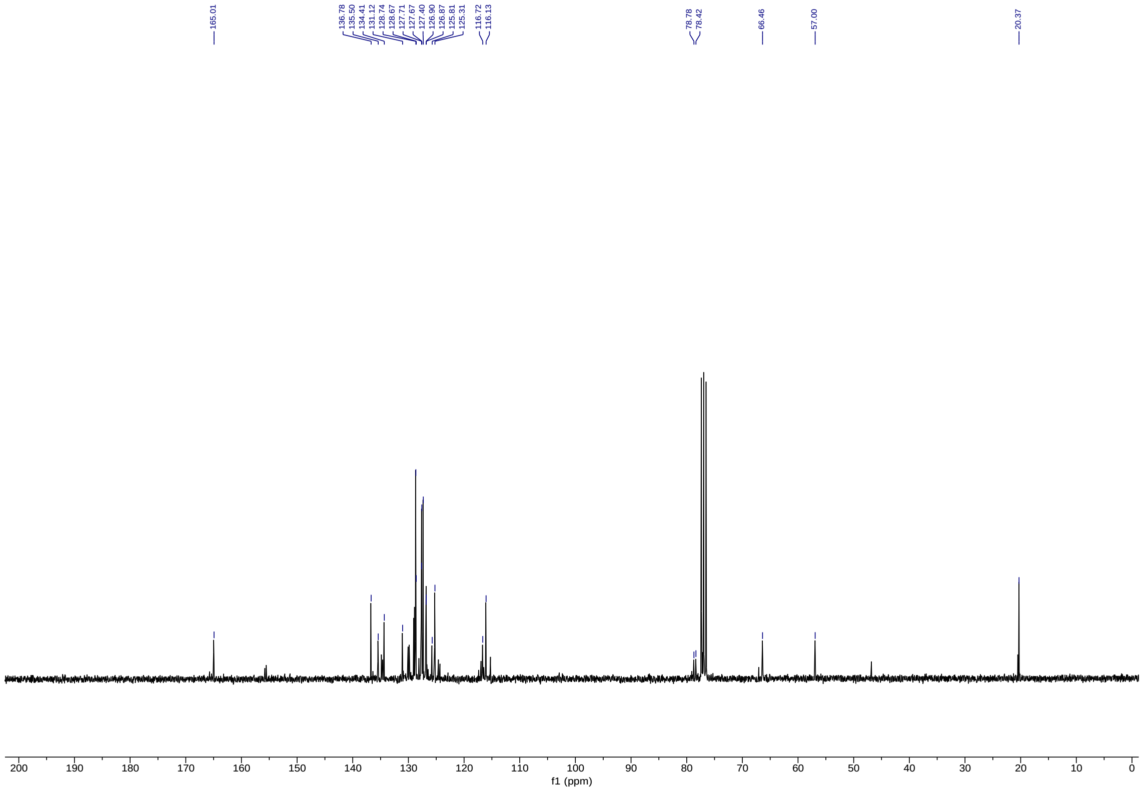
165.01
136.78
135.50
134.41
131.12
128.74
128.67
127.71
127.67
127.40
126.90
126.87
125.81
125.31
116.72
116.13
78.78
78.42
66.46
57.00
20.37
f1 (ppm)

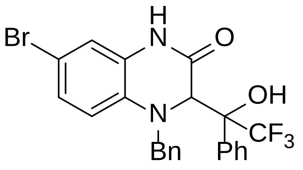

3ia

^{1}H-NMR ($CDCl_3$, 300 MHz)
^{19}F{^{1}H}-NMR ($CDCl_3$, 282 MHz)
^{13}C{^{1}H}-NMR ($CDCl_3$, 75 MHz)

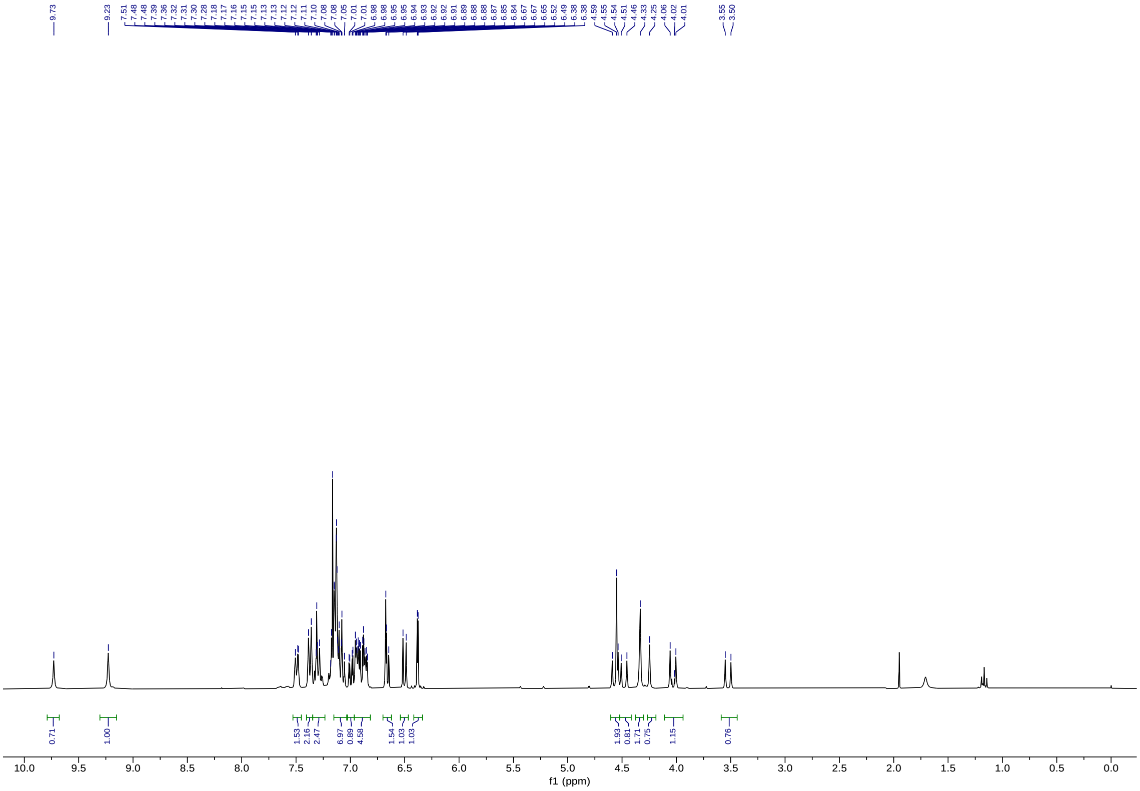

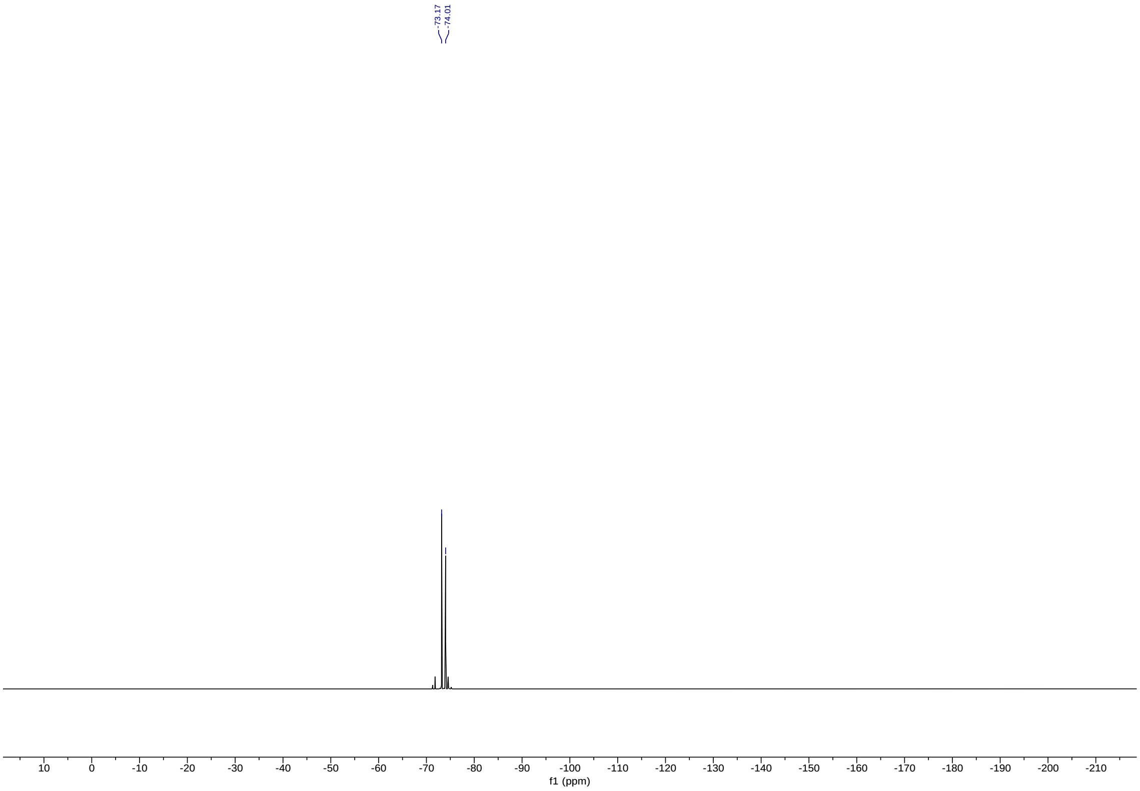

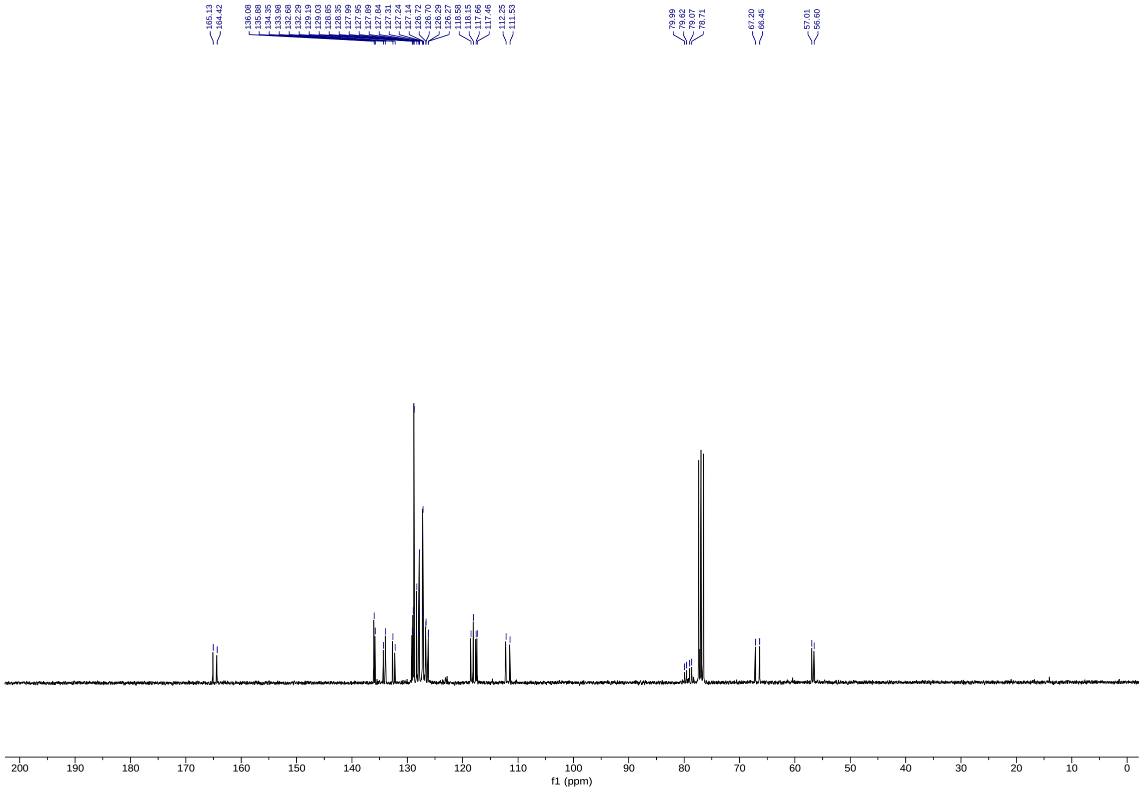
165.13
164.42
136.08
135.88
134.35
133.98
132.68
132.29
129.19
129.03
128.85
128.35
127.99
127.95
127.89
127.84
127.31
127.24
127.14
126.72
126.70
126.29
126.27
118.58
118.15
117.66
117.46
112.25
111.53
79.99
79.62
79.07
78.71
67.20
66.45
57.01
56.60
200
190
180
170
160
150
140
130
120
110
100
90
80
70
60
50
40
30
20
10
0
f1 (ppm)

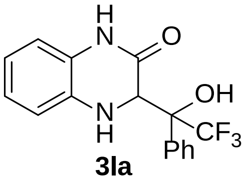

3la

¹H-NMR (DMSO-*d⁶*, 300 MHz)
¹⁹F{¹H}-NMR (DMSO-*d⁶*, 282 MHz)
¹³C{¹H}-NMR (DMSO-*d⁶*, 75 MHz)

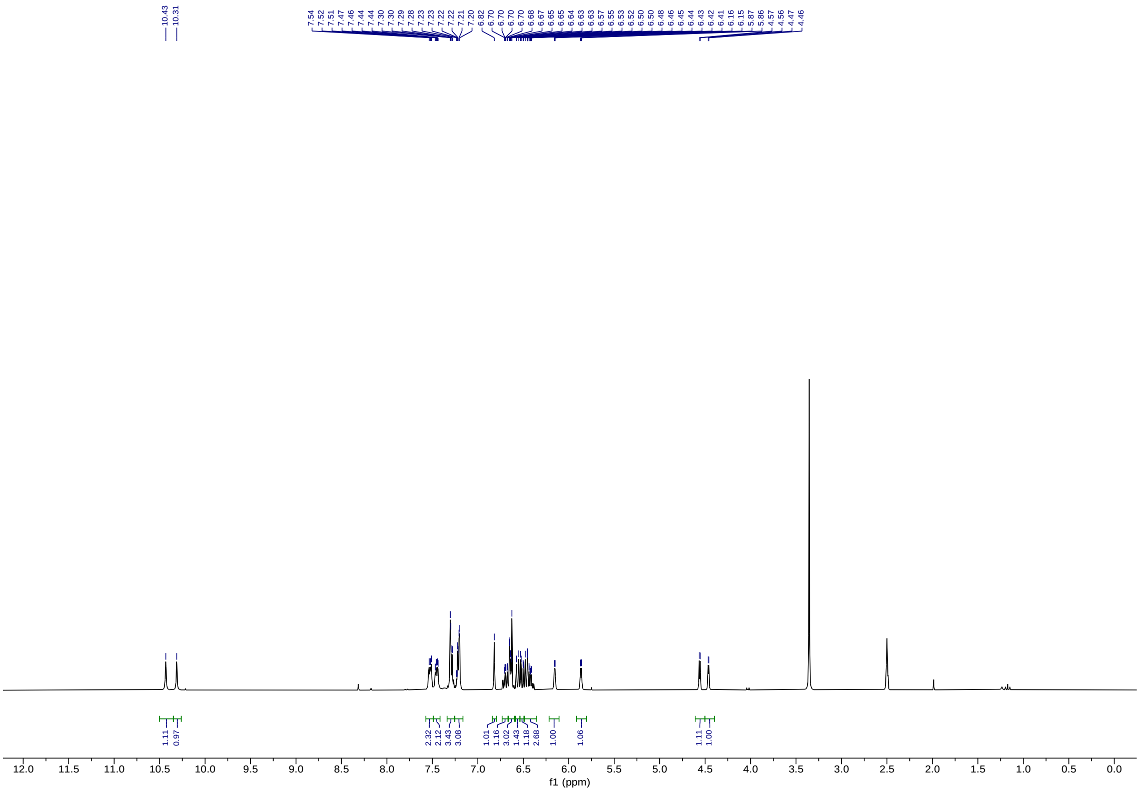

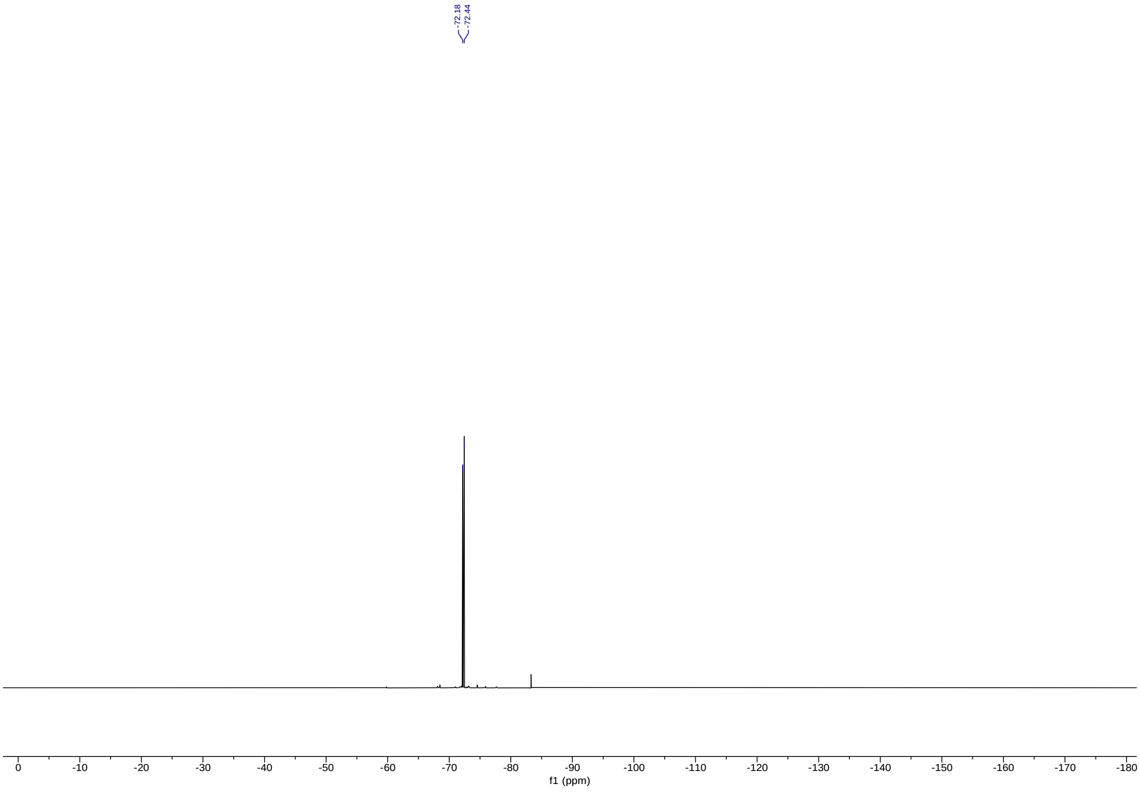

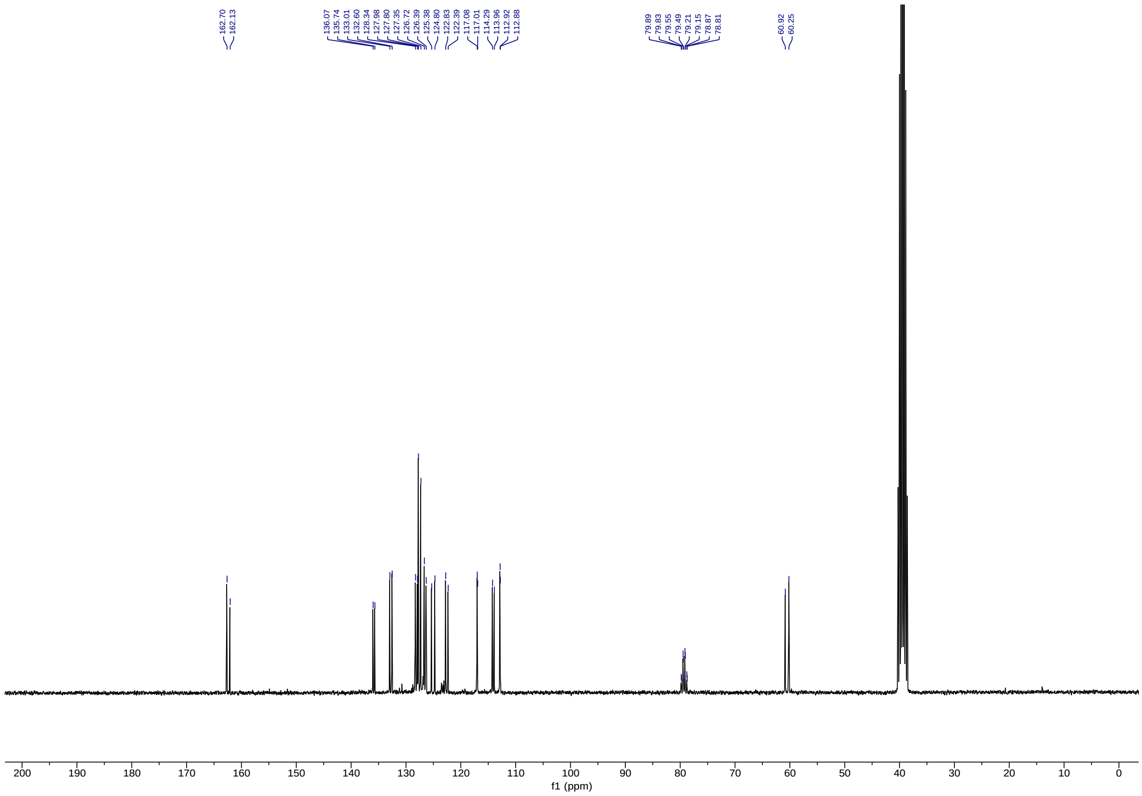
162.70
162.13
136.07
135.74
133.01
132.60
128.34
127.98
127.80
127.35
126.72
126.39
125.38
124.80
122.83
122.39
117.08
117.01
114.29
113.96
112.92
112.88
79.89
79.83
79.55
79.49
79.21
79.15
78.87
78.81
60.92
60.25
f1 (ppm)

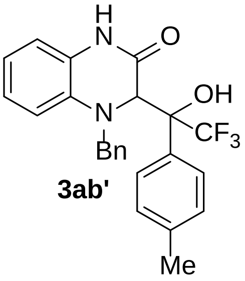

3ab'

^{1}H-NMR ($CDCl_3$, 300 MHz)
$^{19}F\{^{1}H\}$-NMR ($CDCl_3$, 282 MHz)
$^{13}C\{^{1}H\}$-NMR ($CDCl_3$, 75 MHz)

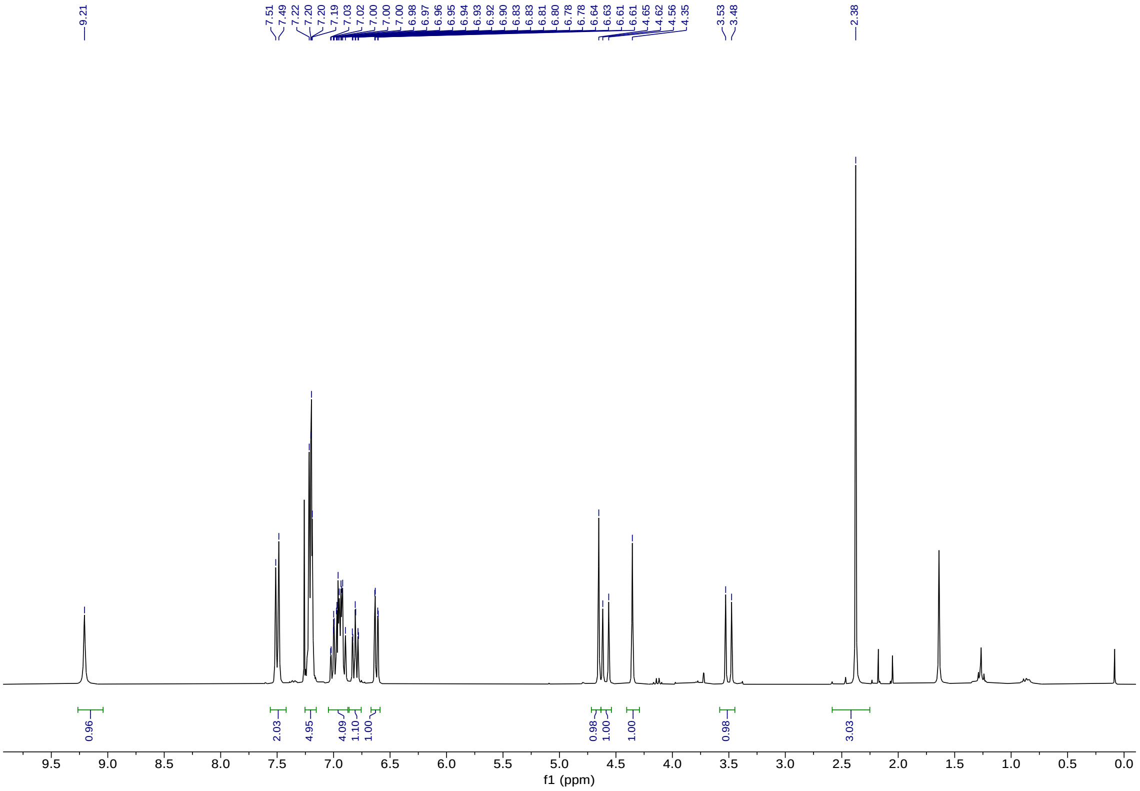

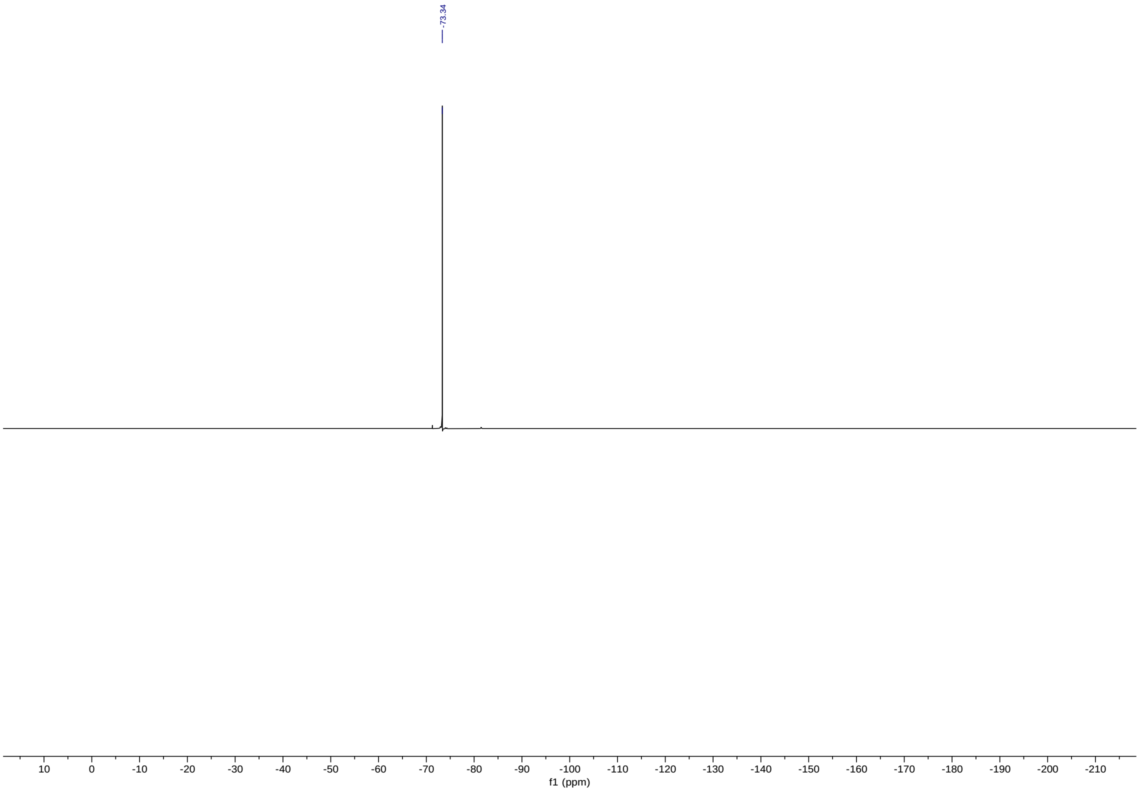

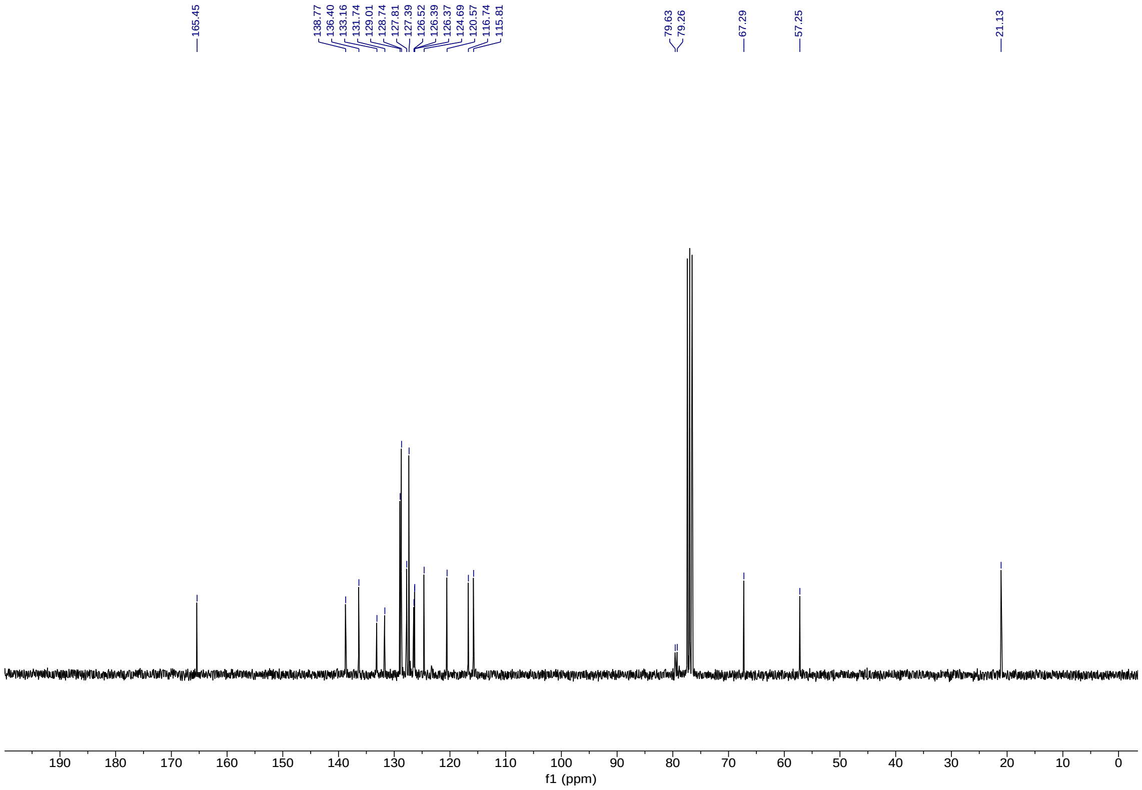
165.45
138.77
136.40
133.16
131.74
129.01
128.74
127.81
127.39
126.52
126.39
126.37
124.69
120.57
116.74
115.81
79.63
79.26
67.29
57.25
21.13
190 180 170 160 150 140 130 120 110 100 90 80 70 60 50 40 30 20 10 0
f1 (ppm)

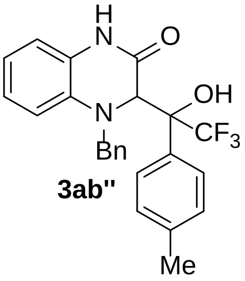

3ab''

^{1}H-NMR ($CDCl_3$, 300 MHz)
^{19}F{^{1}H}-NMR ($CDCl_3$, 282 MHz)
^{13}C{^{1}H}-NMR ($CDCl_3$, 75 MHz)

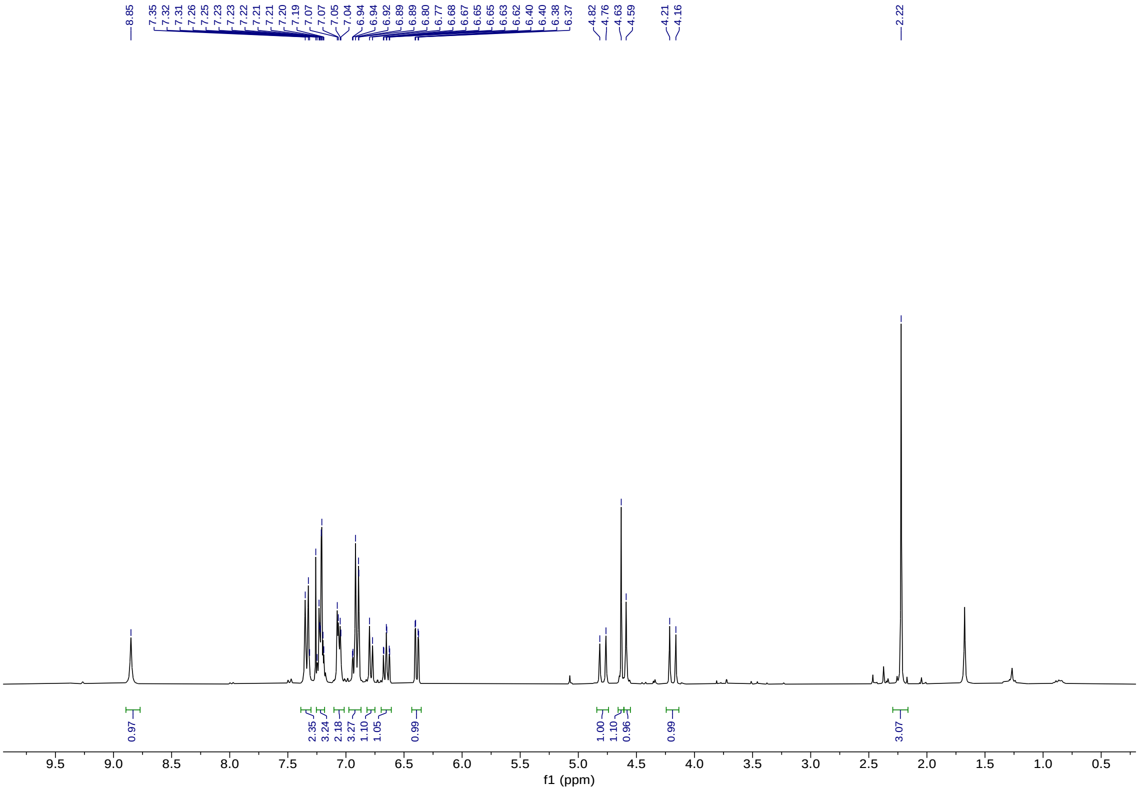

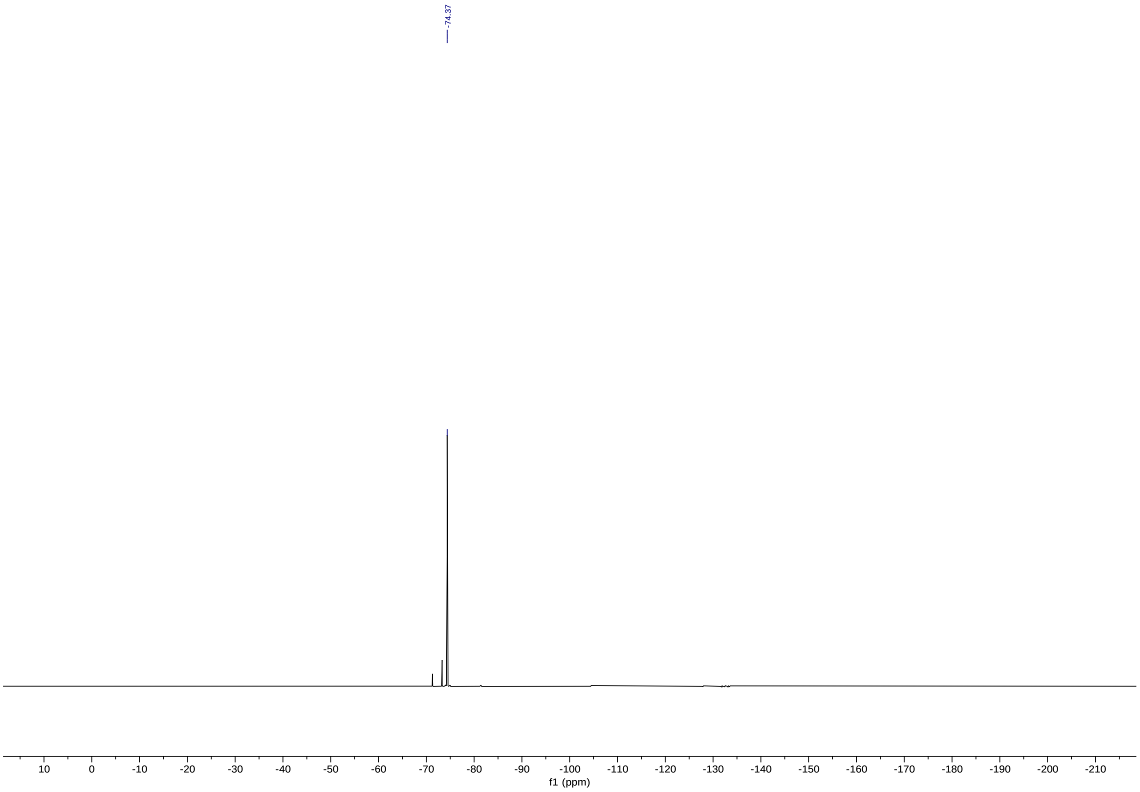

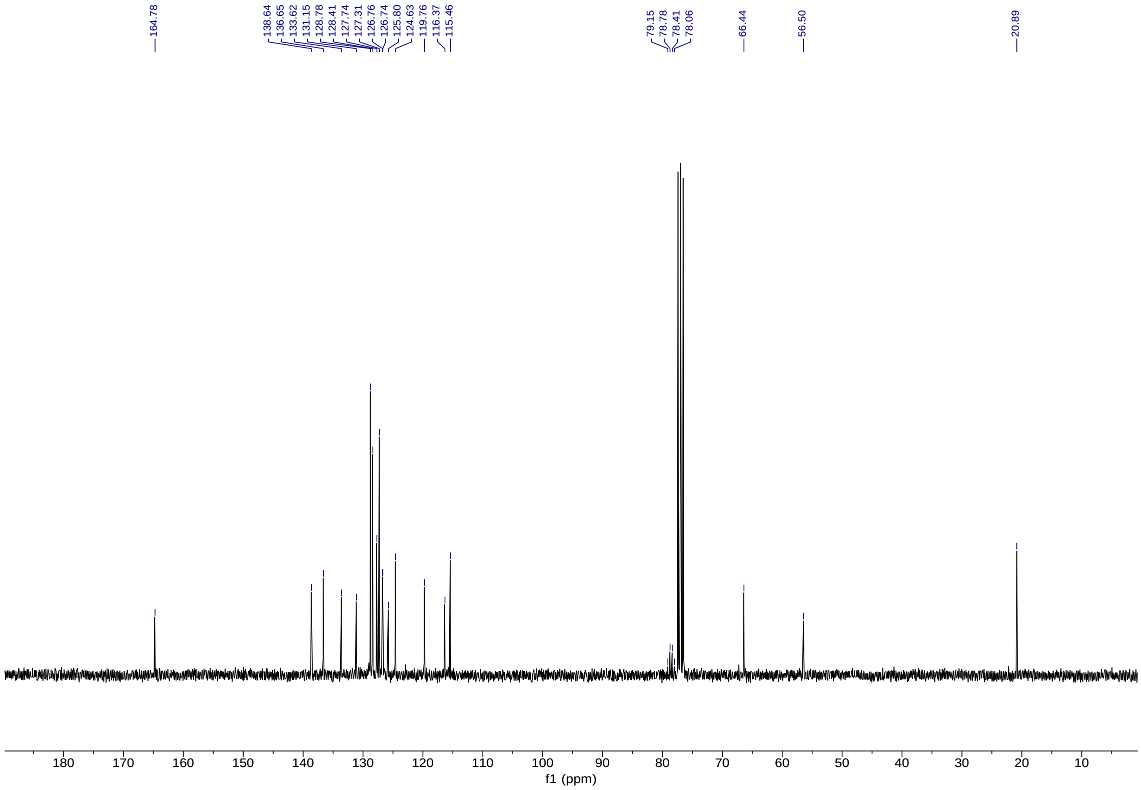
164.78
138.64
136.65
133.62
131.15
128.78
128.41
127.74
127.31
126.76
126.74
125.80
124.63
119.76
116.37
115.46
79.15
78.78
78.41
78.06
66.44
56.50
20.89
180 170 160 150 140 130 120 110 100 90 80 70 60 50 40 30 20 10
f1 (ppm)

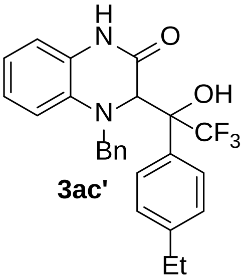

3ac'

^{1}H-NMR ($CDCl_3$, 300 MHz)
^{19}F{^{1}H}-NMR ($CDCl_3$, 282 MHz)
^{13}C{^{1}H}-NMR ($CDCl_3$, 75 MHz)

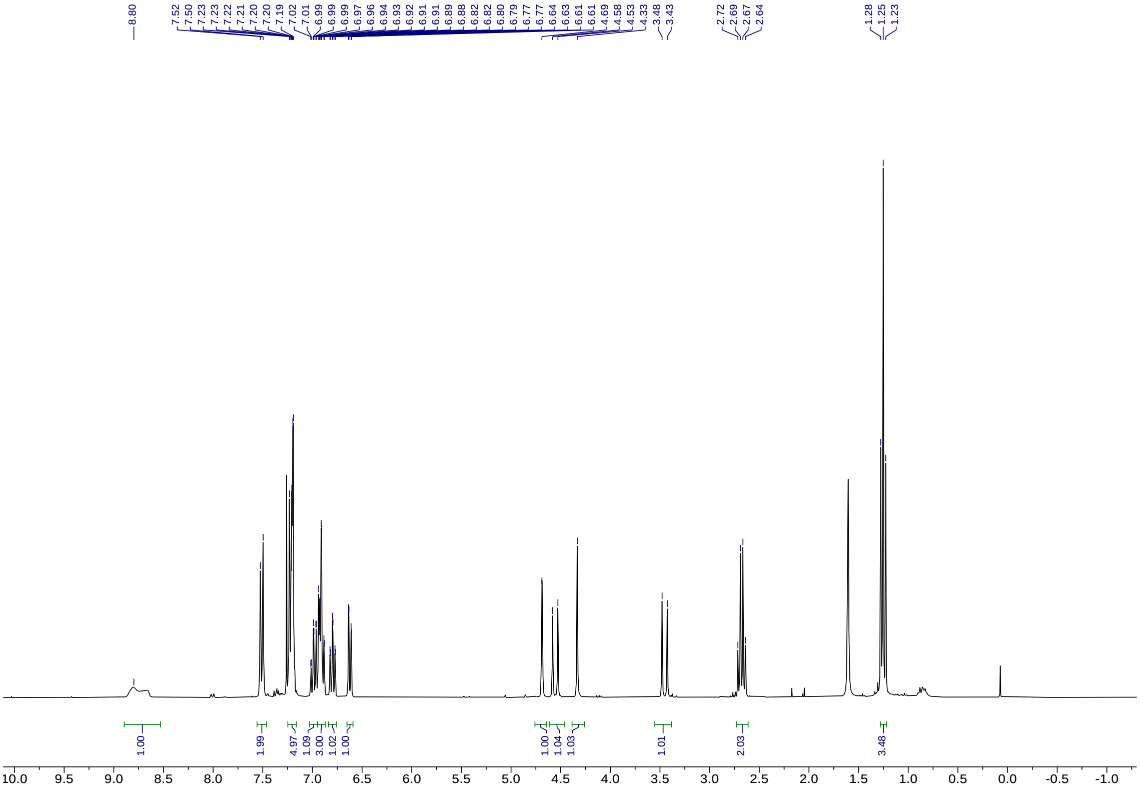

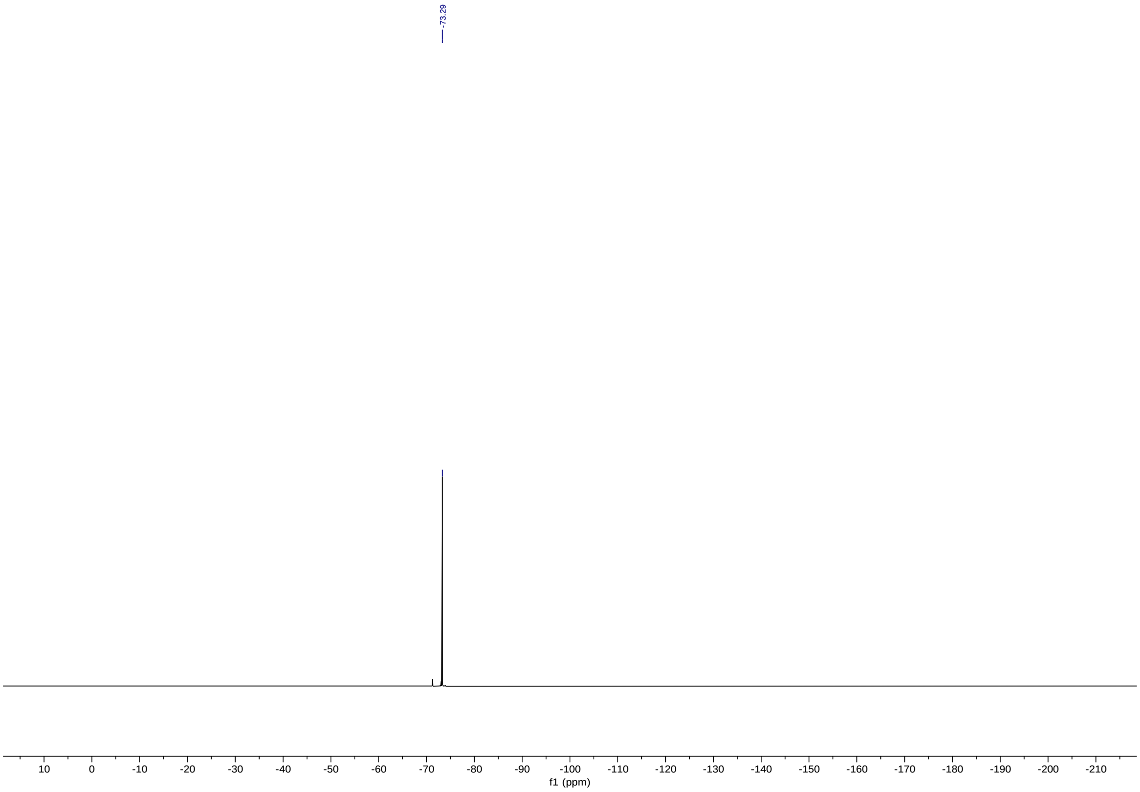

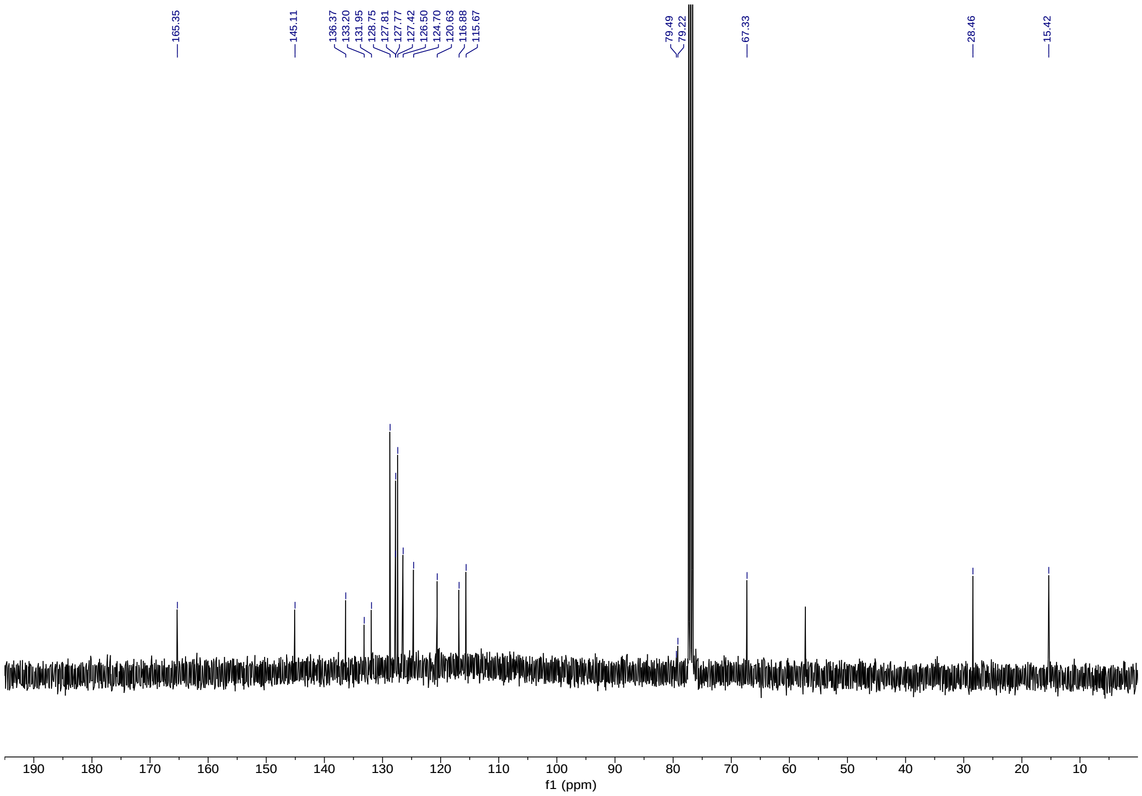
165.35
145.11
136.37
133.20
131.95
128.75
127.81
127.77
127.42
126.50
124.70
120.63
116.88
115.67
79.49
79.22
67.33
28.46
15.42
190 180 170 160 150 140 130 120 110 100 90 80 70 60 50 40 30 20 10
f1 (ppm)

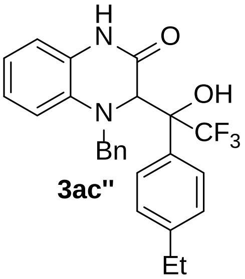

3ac''

^{1}H-NMR ($CDCl_3$, 300 MHz)
^{19}F{^{1}H}-NMR ($CDCl_3$, 282 MHz)
^{13}C{^{1}H}-NMR ($CDCl_3$, 75 MHz)

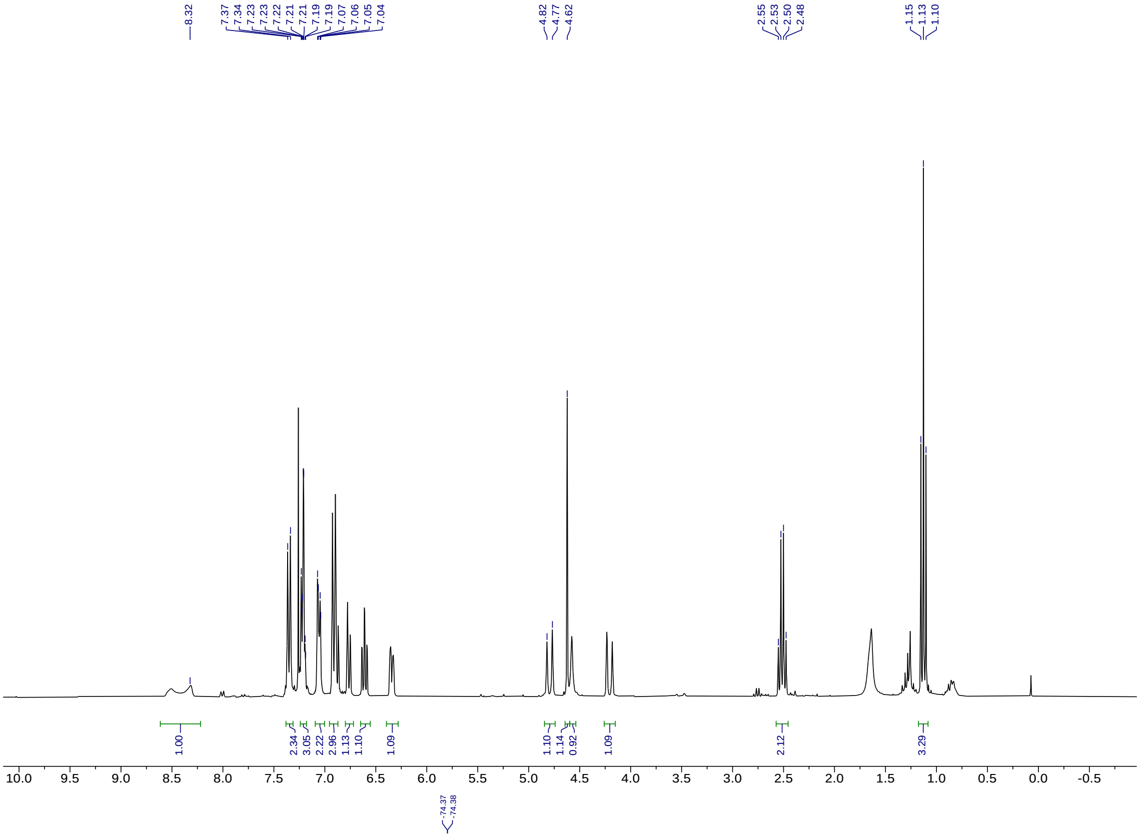

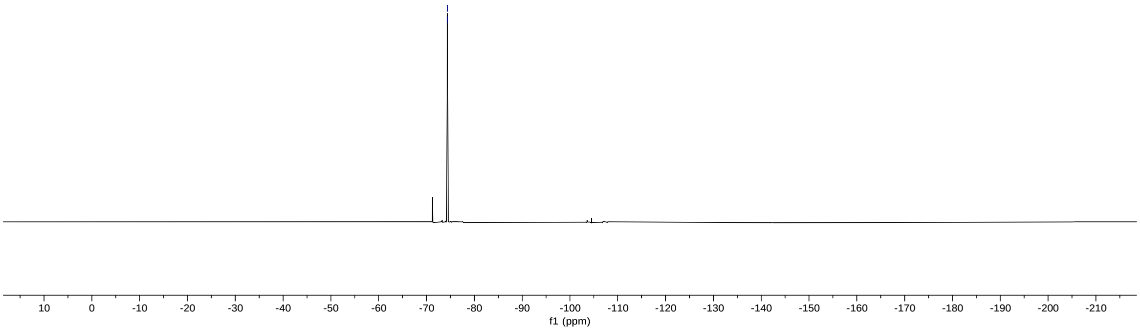

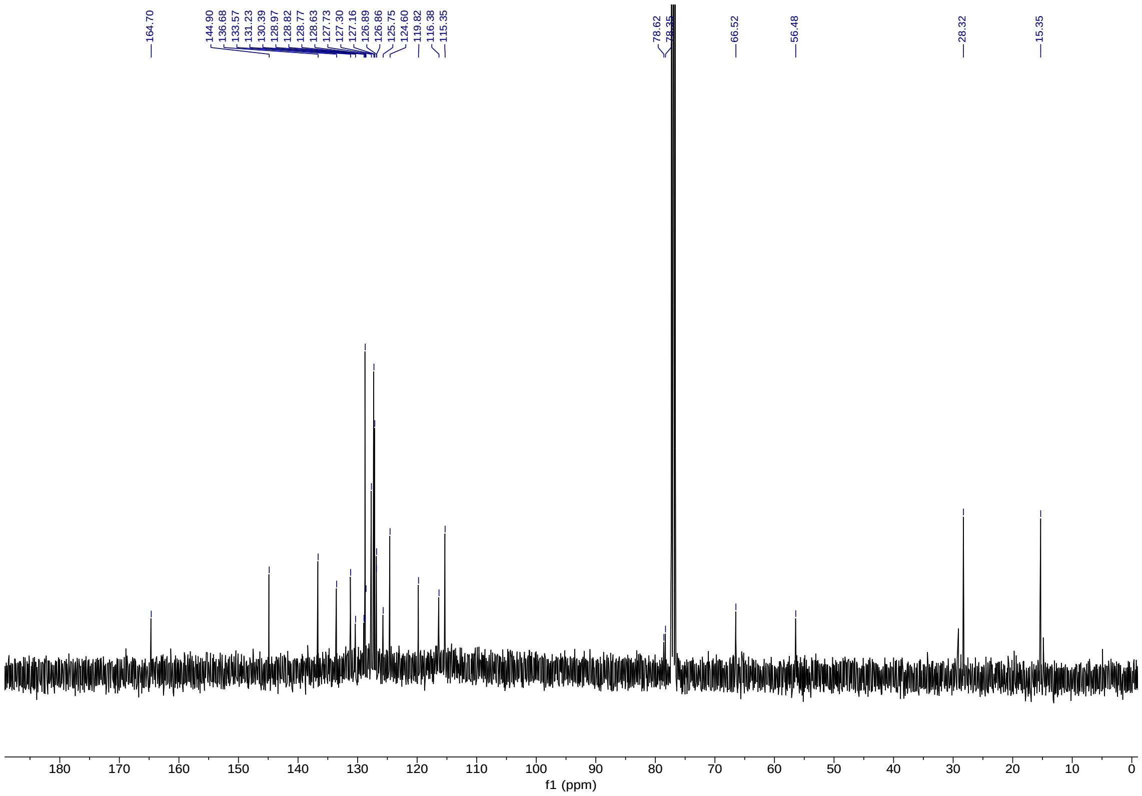
164.70
144.90
136.68
133.57
131.23
130.39
128.97
128.82
128.77
128.63
127.73
127.30
127.16
126.89
126.86
125.75
124.60
119.82
116.38
115.35
78.62
66.52
56.48
28.32
15.35
180
170
160
150
140
130
120
110
100
90
80
70
60
50
40
30
20
10
0
f1 (ppm)

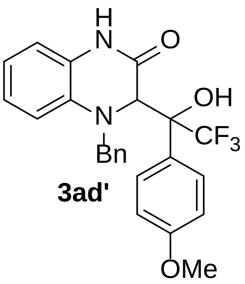

3ad'

^{1}H-NMR ($CDCl_3$, 300 MHz)
^{19}F{^{1}H}-NMR ($CDCl_3$, 282 MHz)
^{13}C{^{1}H}-NMR ($CDCl_3$, 75 MHz)

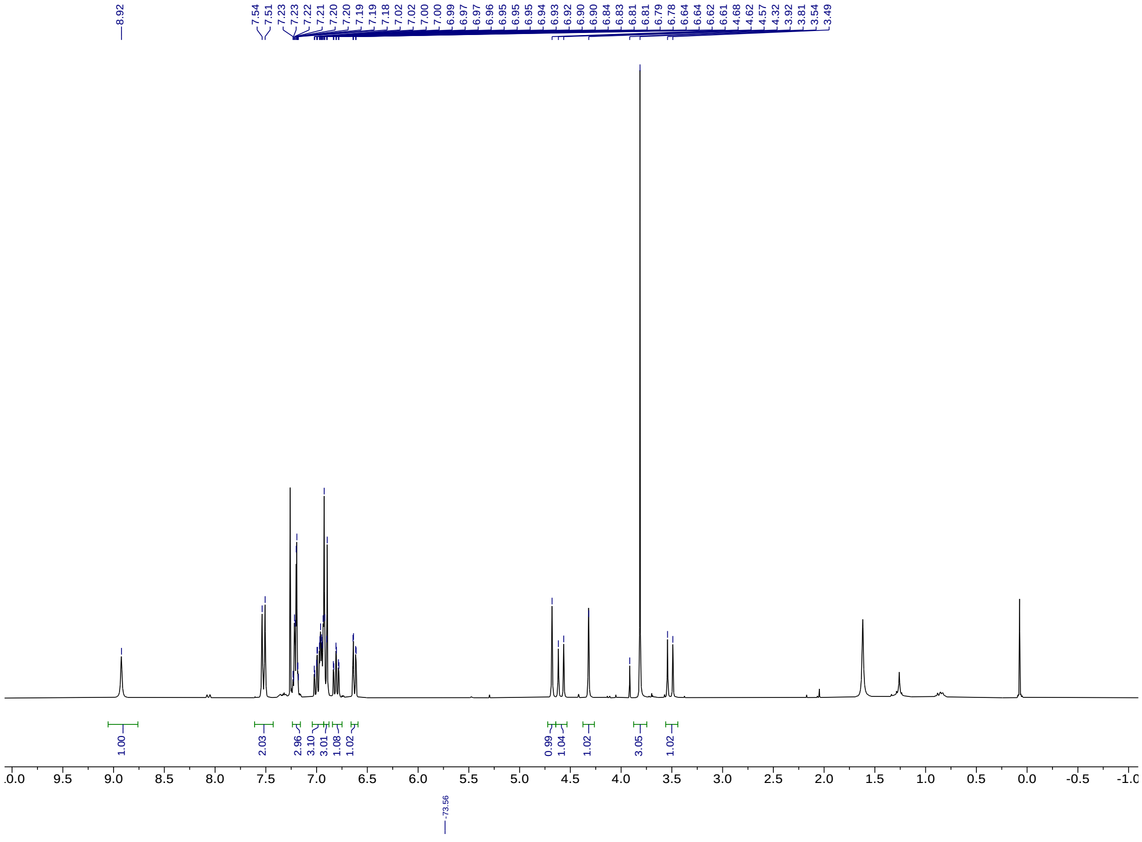

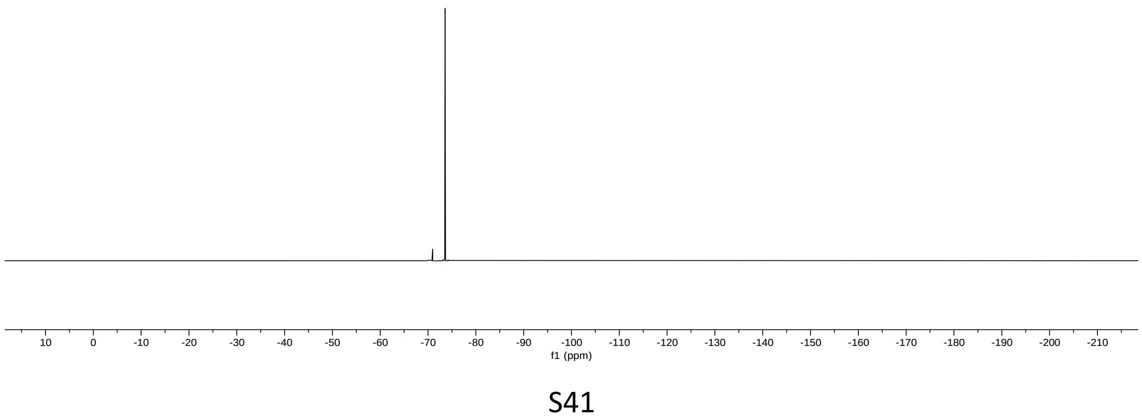

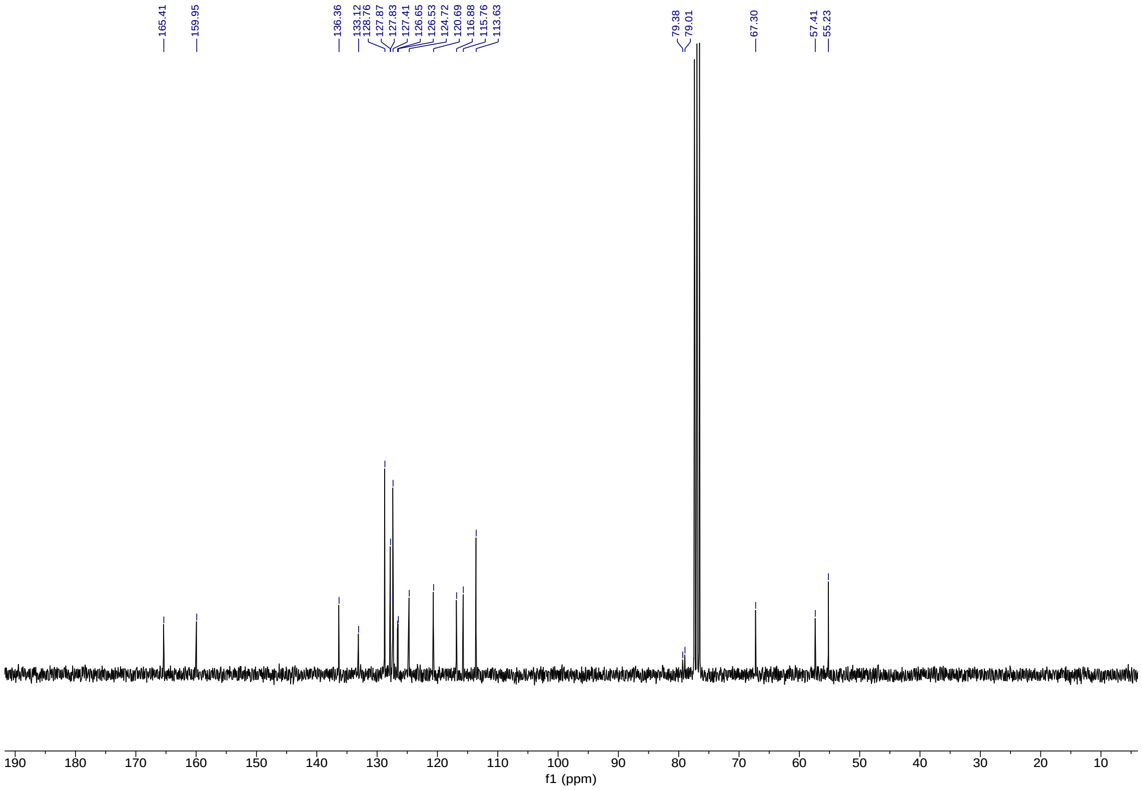

165.41
159.95
136.36
133.12
128.76
127.87
127.83
127.41
126.65
126.53
124.72
120.69
116.88
115.76
113.63
79.38
79.01
67.30
57.41
55.23
190
180
170
160
150
140
130
120
110
100
90
80
70
60
50
40
30
20
10
f1 (ppm)

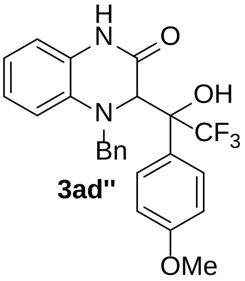

3ad''

^{1}H-NMR (CDCl$_3$, 300 MHz)
^{19}F{^{1}H}-NMR (CDCl$_3$, 282 MHz)
^{13}C{^{1}H}-NMR (CDCl$_3$, 75 MHz)

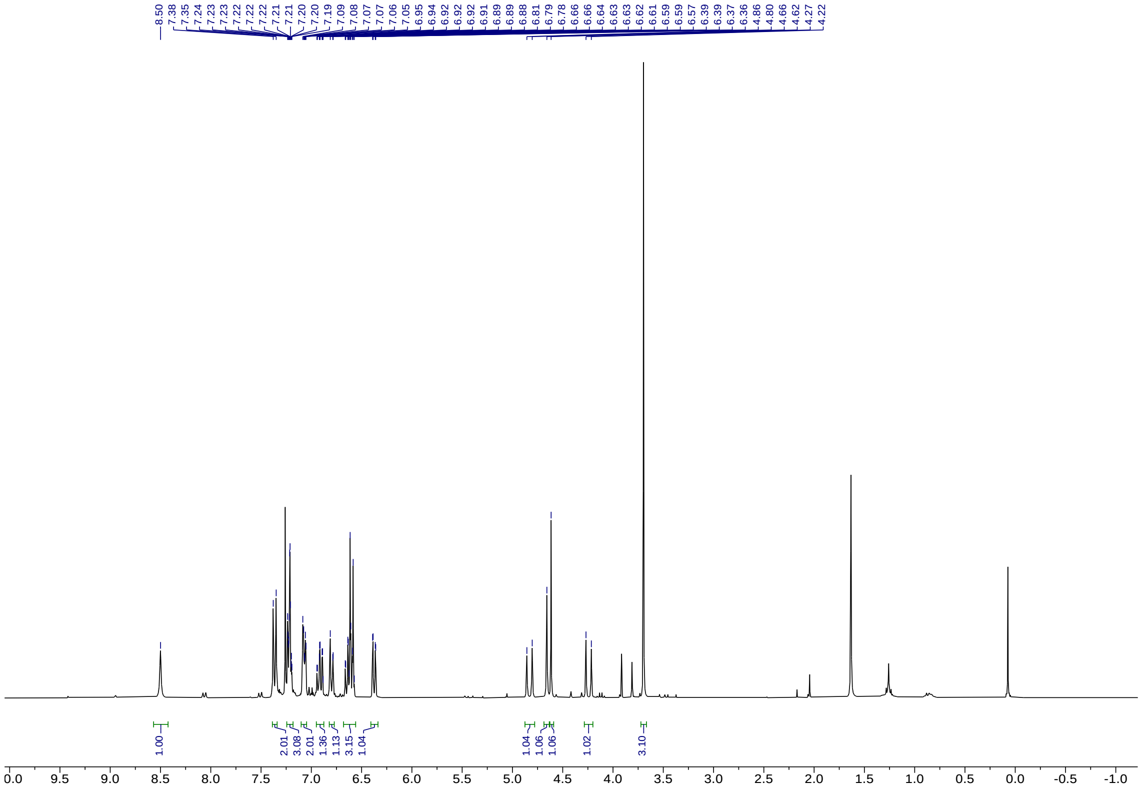

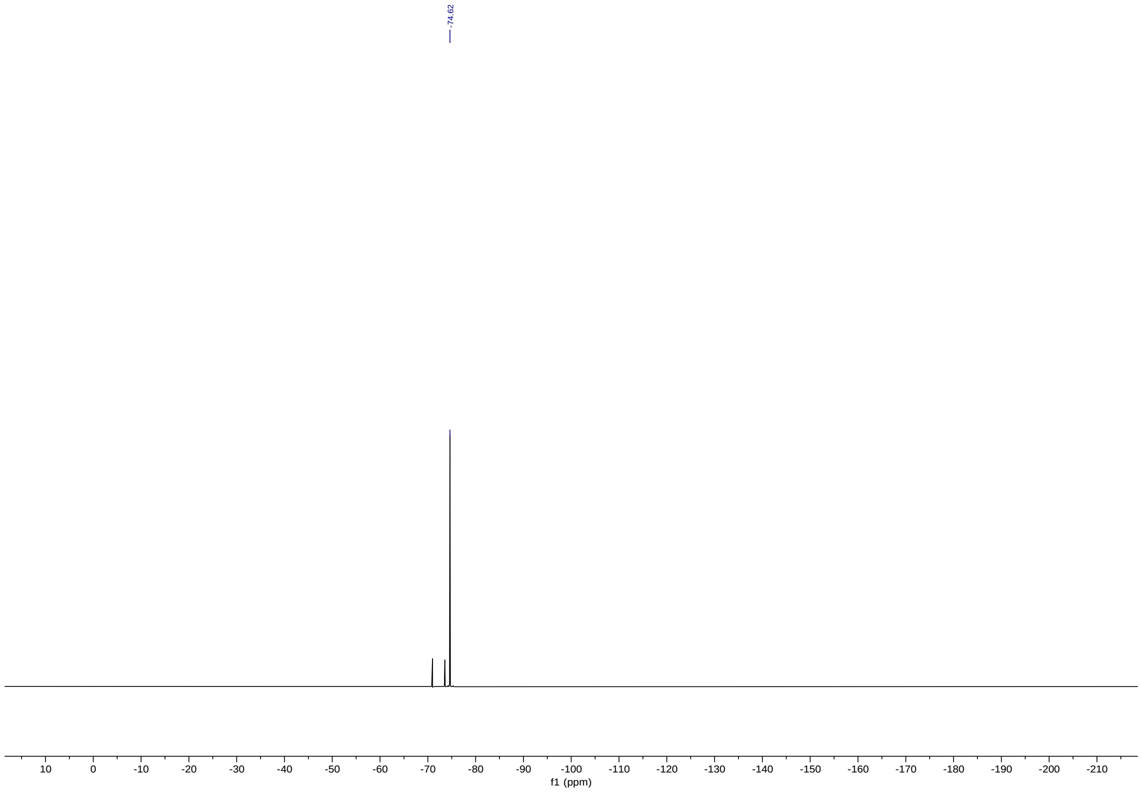

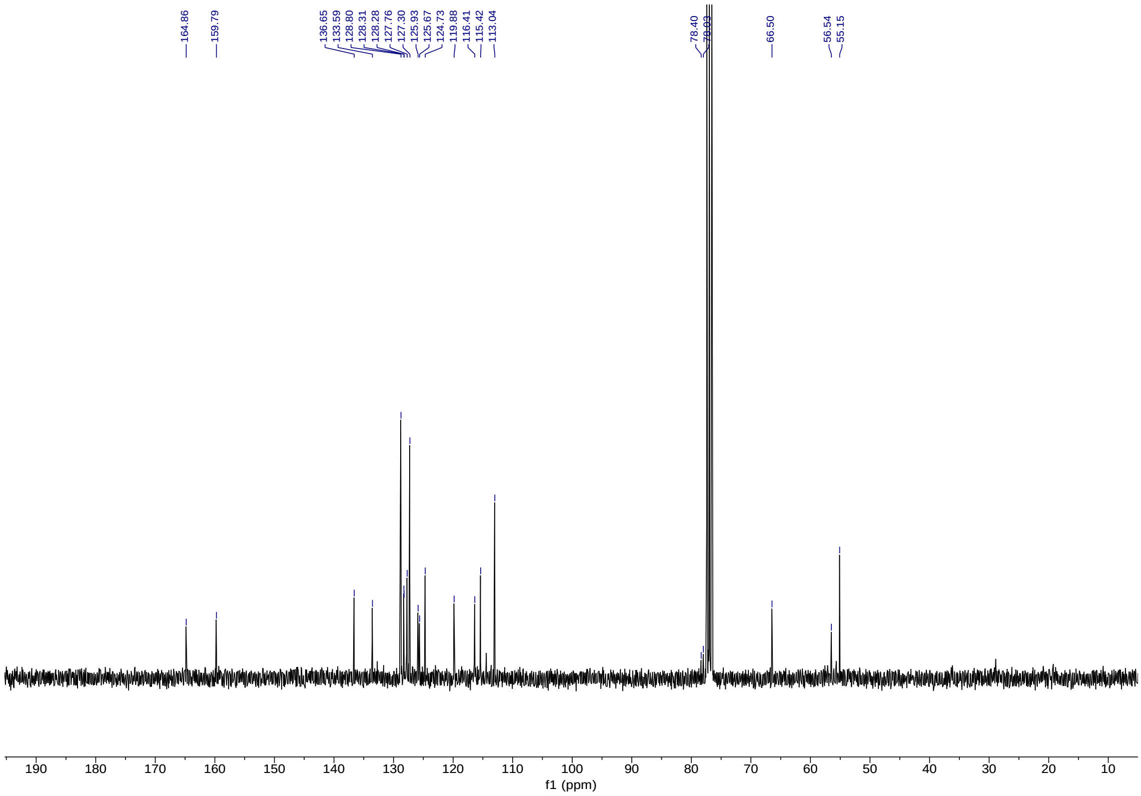
164.86
159.79
136.65
133.59
128.80
128.31
128.28
127.76
127.30
125.93
125.67
124.73
119.88
116.41
115.42
113.04
78.40
66.50
56.54
55.15
190
180
170
160
150
140
130
120
110
100
90
80
70
60
50
40
30
20
10
f1 (ppm)

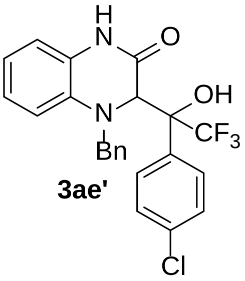

3ae'

^{1}H-NMR (CDCl$_3$, 300 MHz)
^{19}F{^{1}H}-NMR (CDCl$_3$, 282 MHz)
^{13}C{^{1}H}-NMR (CDCl$_3$, 75 MHz)

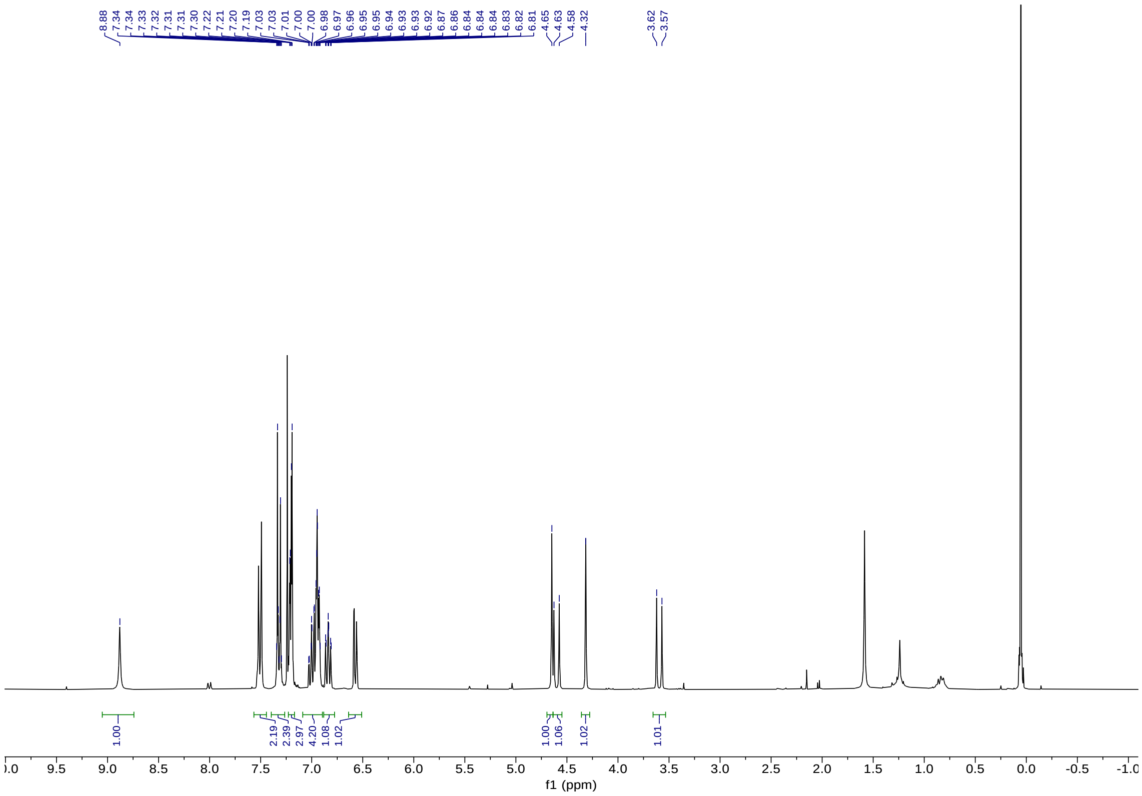

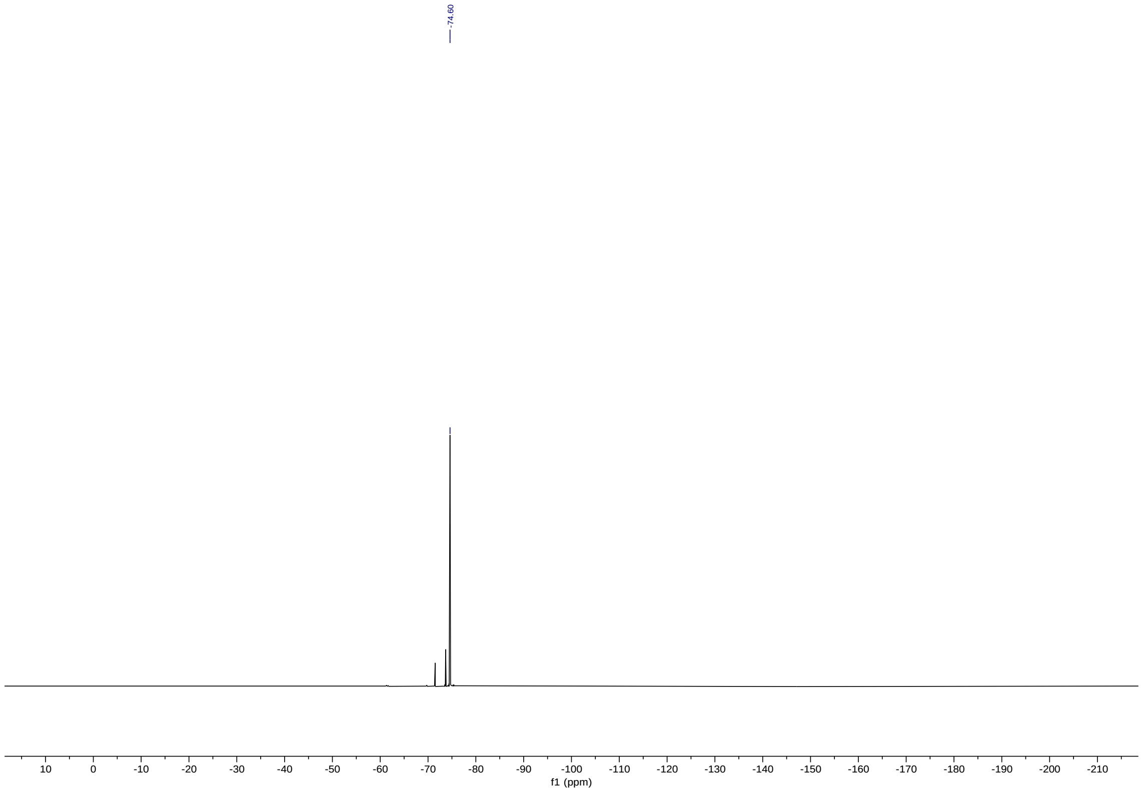

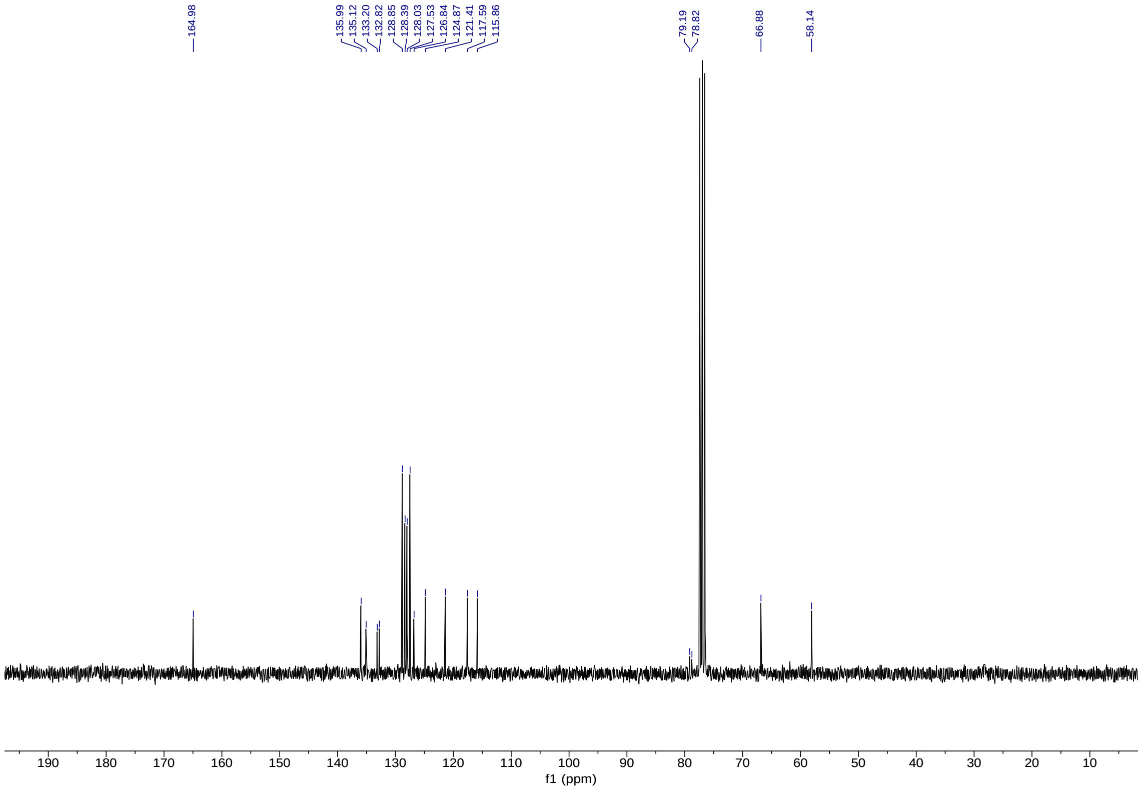
164.98
135.99
135.12
133.20
132.82
128.85
128.39
128.03
127.53
126.84
124.87
121.41
117.59
115.86
79.19
78.82
66.88
58.14
190 180 170 160 150 140 130 120 110 100 90 80 70 60 50 40 30 20 10
f1 (ppm)

3ae''

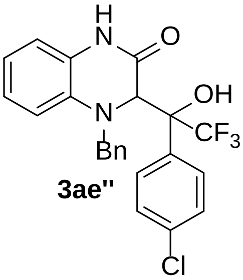

^{1}H-NMR ($CDCl_3$, 300 MHz)
^{19}F{^{1}H}-NMR ($CDCl_3$, 282 MHz)
^{13}C{^{1}H}-NMR ($CDCl_3$, 75 MHz)

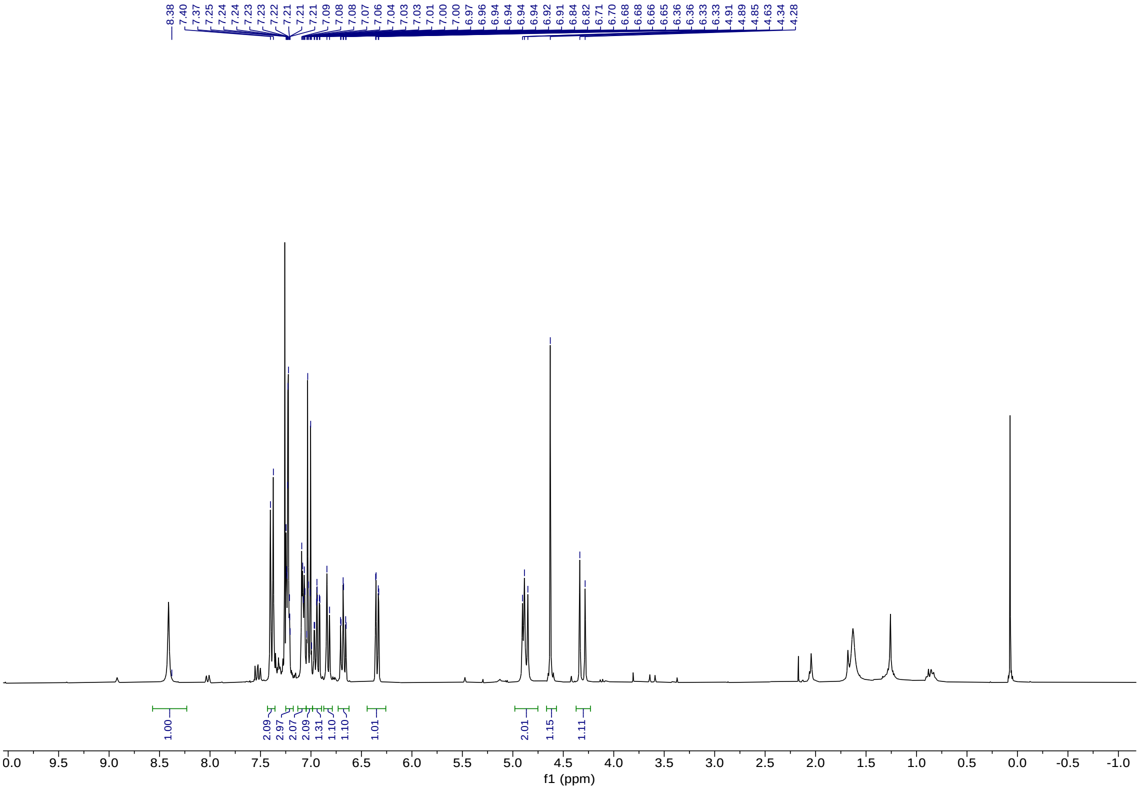

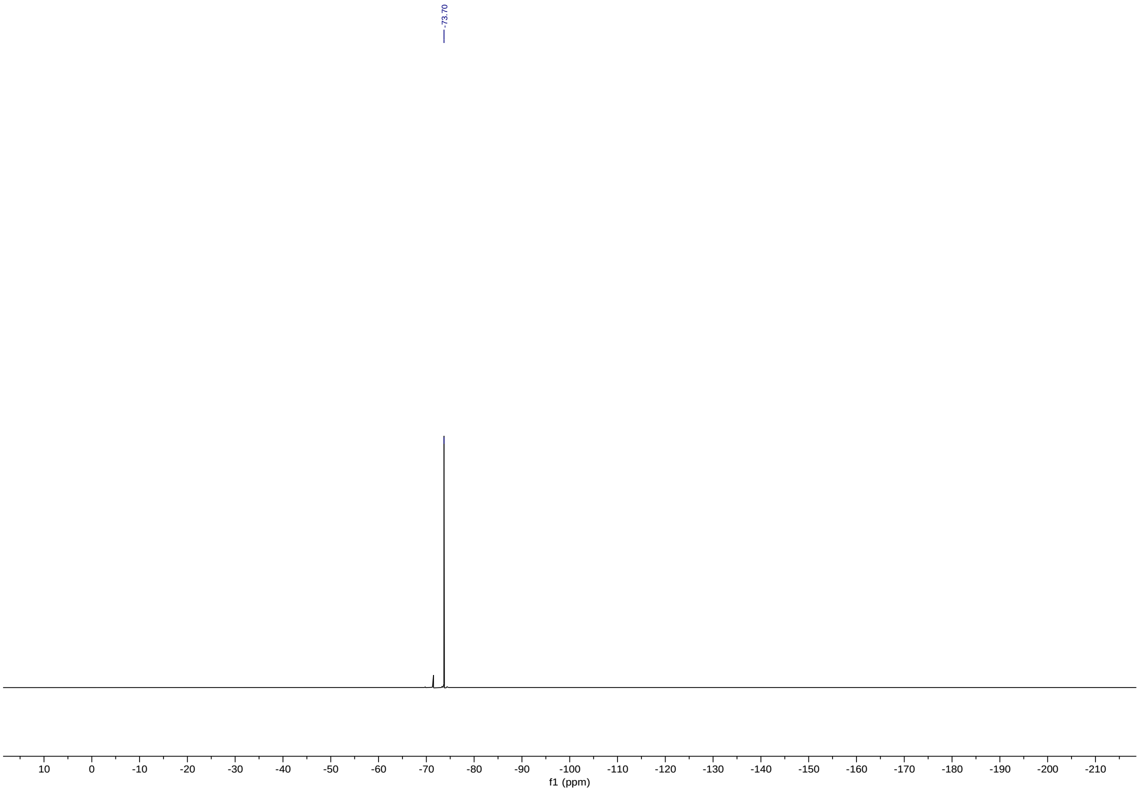

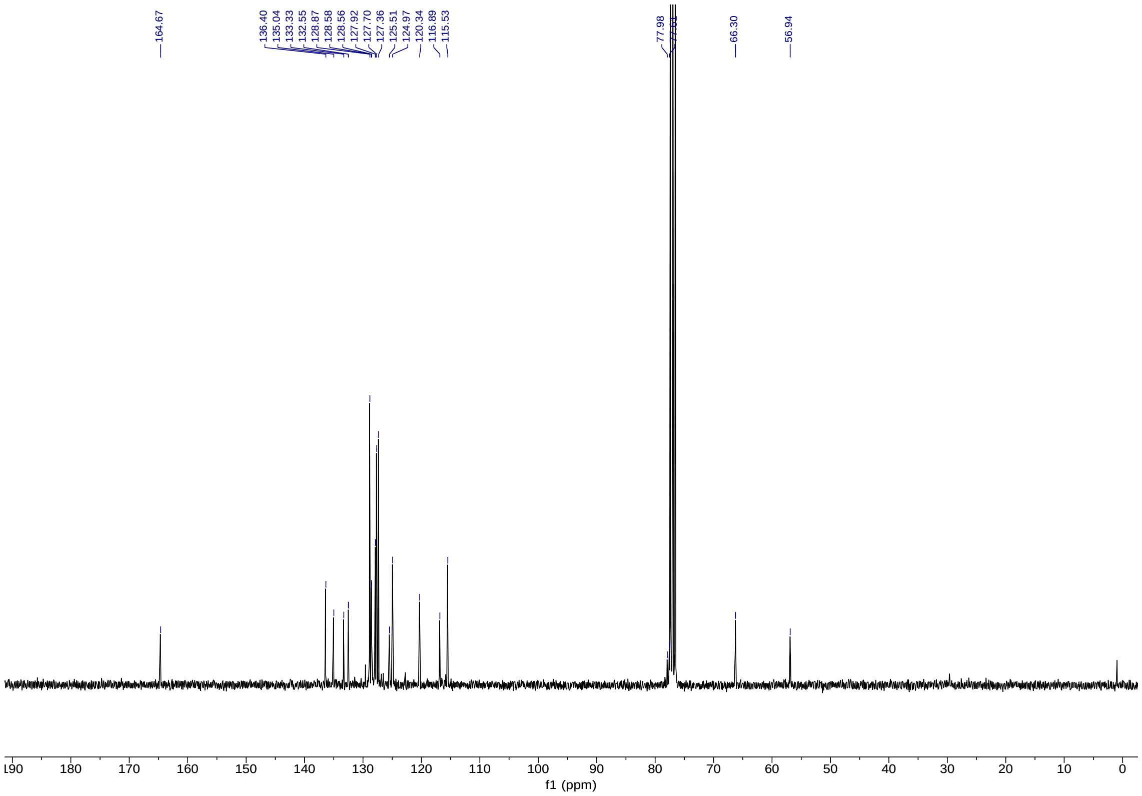
164.67
136.40
135.04
133.33
132.55
128.87
128.58
128.56
127.92
127.70
127.36
125.51
124.97
120.34
116.89
115.53
77.98
77.61
66.30
56.94
190
180
170
160
150
140
130
120
110
100
90
80
70
60
50
40
30
20
10
0
f1 (ppm)

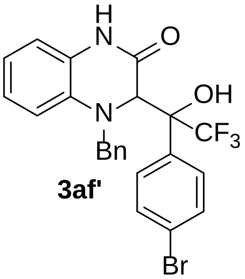

3af'

^{1}H-NMR (CDCl$_3$, 300 MHz)
^{19}F{^{1}H}-NMR (CDCl$_3$, 282 MHz)
^{13}C{^{1}H}-NMR (CDCl$_3$, 75 MHz)

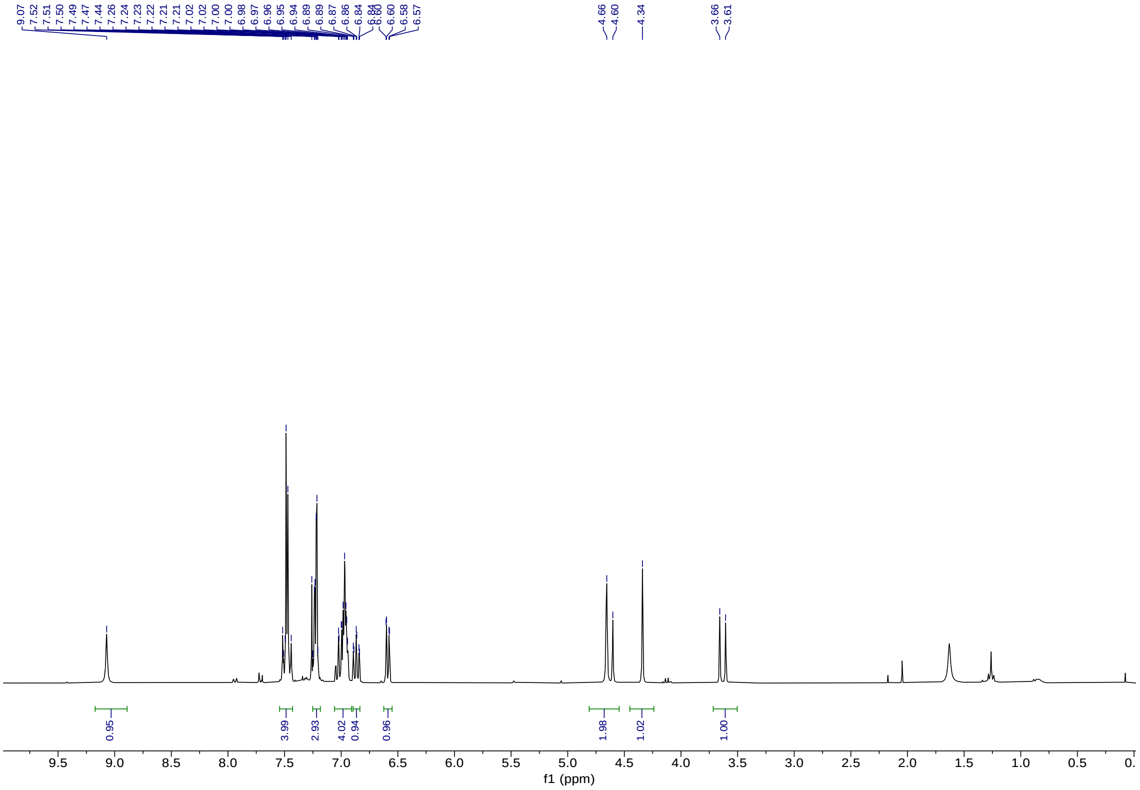

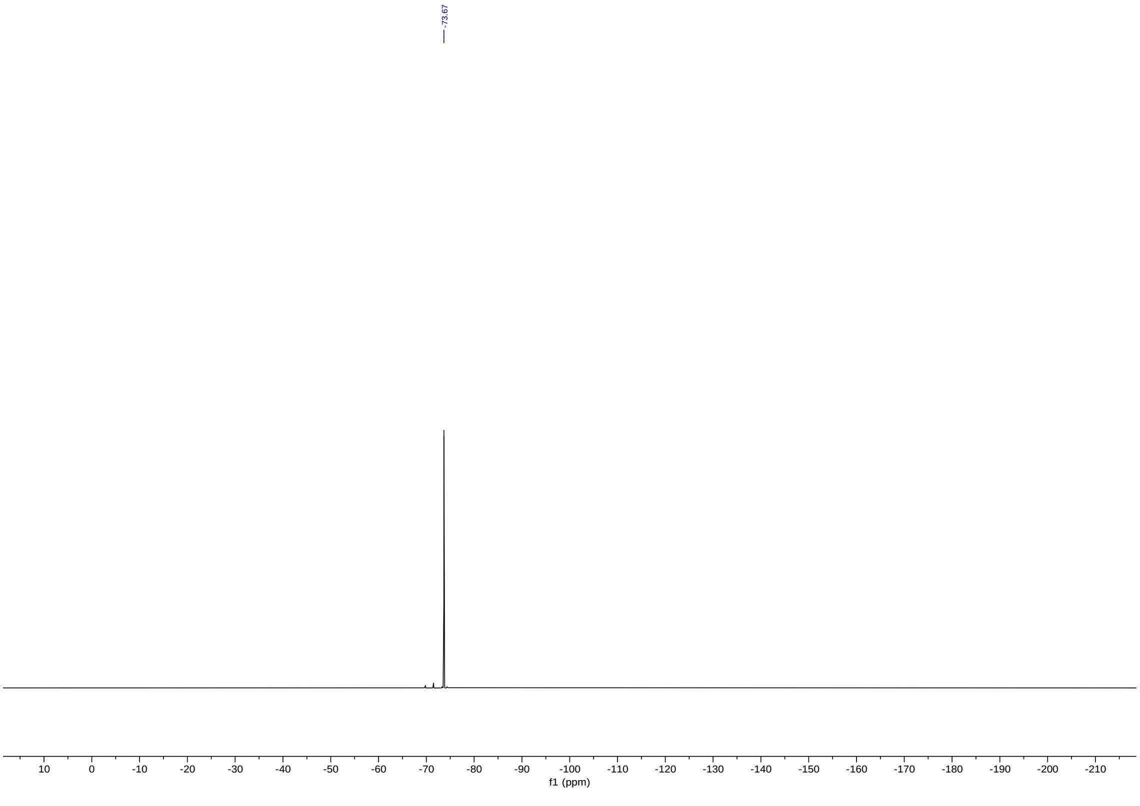

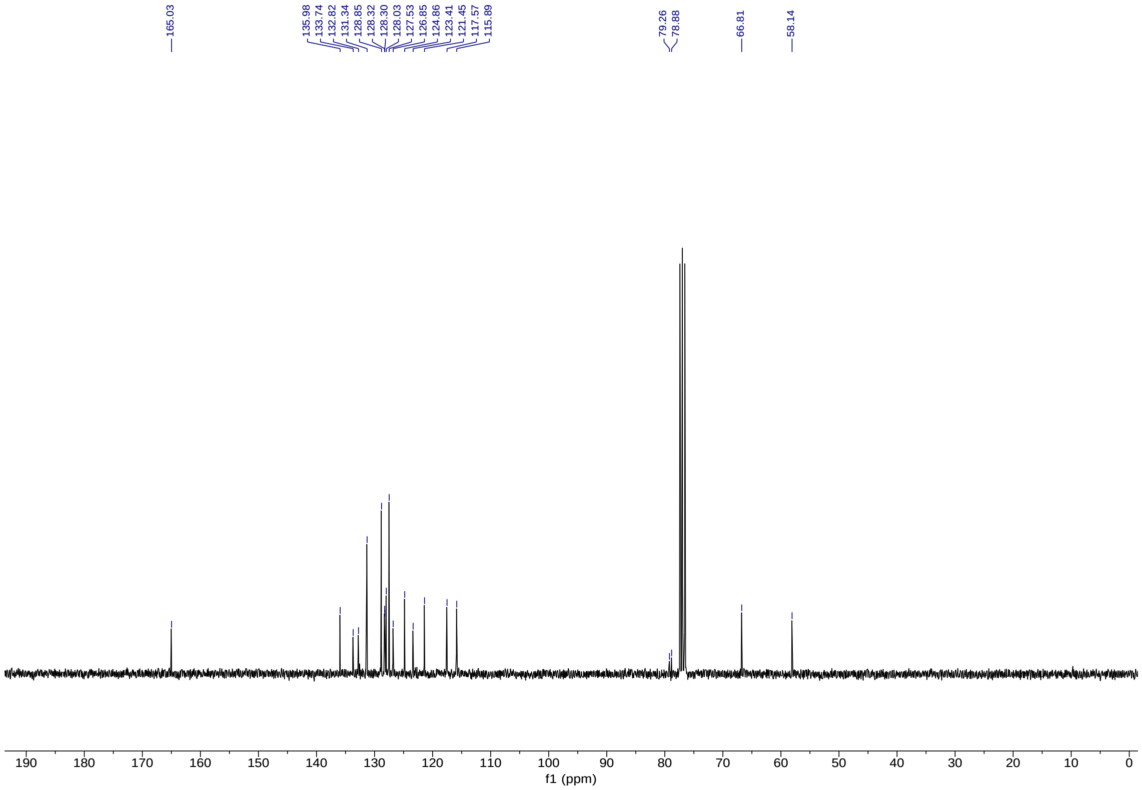
165.03
135.98
133.74
132.82
131.34
128.85
128.32
128.30
128.03
127.53
126.85
124.86
123.41
121.45
117.57
115.89
79.26
78.88
66.81
58.14
190 180 170 160 150 140 130 120 110 100 90 80 70 60 50 40 30 20 10 0
f1 (ppm)

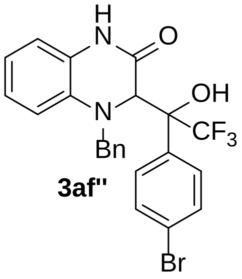

3af''

^{1}H-NMR ($CDCl_3$, 300 MHz)
^{19}F{^{1}H}-NMR ($CDCl_3$, 282 MHz)
^{13}C{^{1}H}-NMR ($CDCl_3$, 75 MHz)

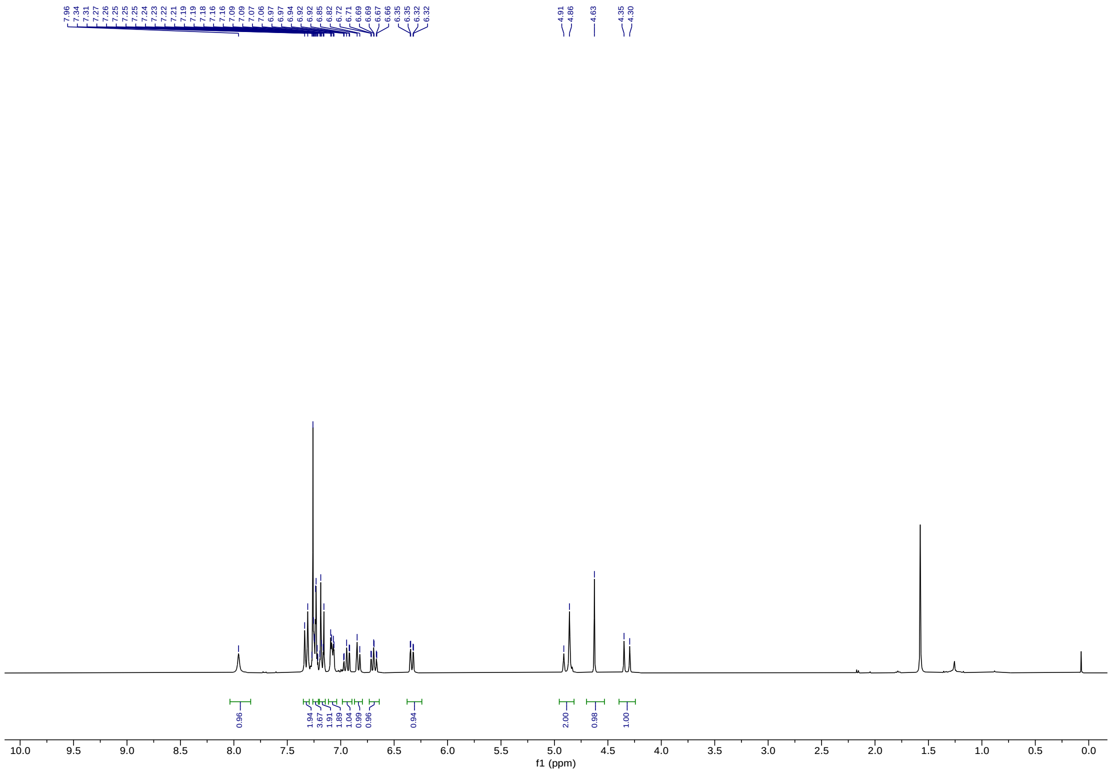

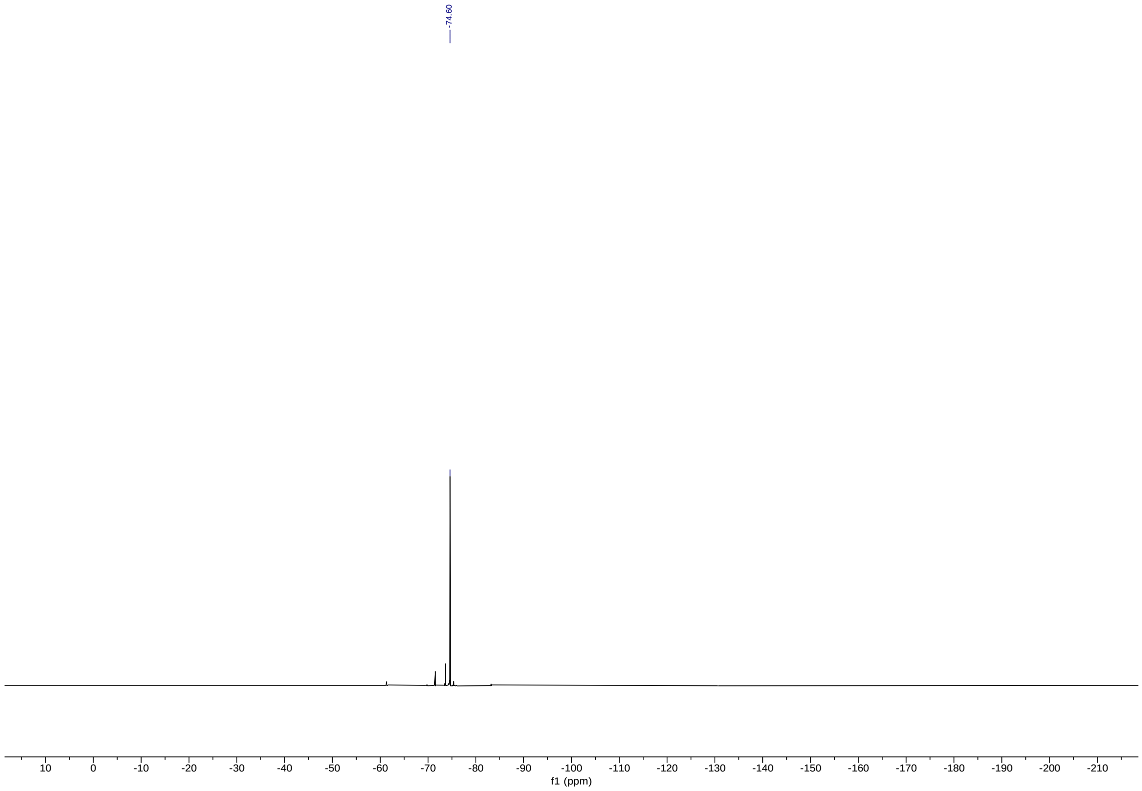

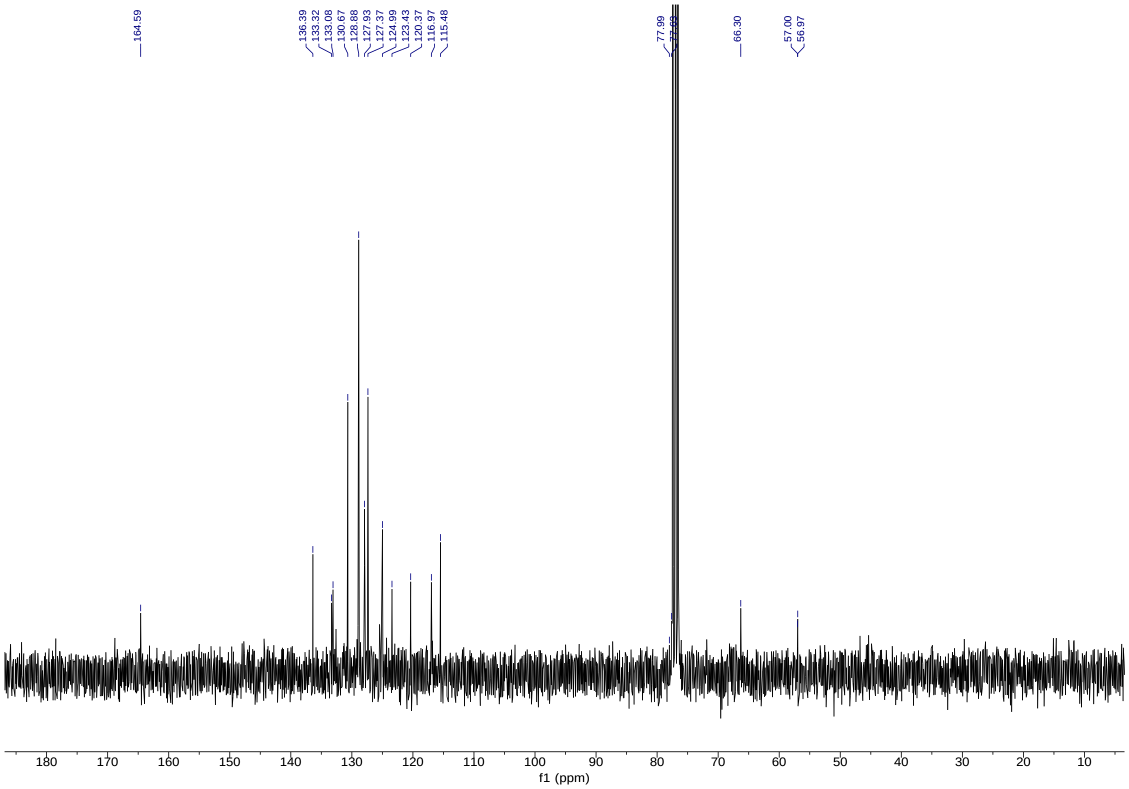
164.59
136.39
133.32
133.08
130.67
128.88
127.93
127.37
124.99
123.43
120.37
116.97
115.48
77.99
66.30
57.00
56.97
180
170
160
150
140
130
120
110
100
90
80
70
60
50
40
30
20
10
f1 (ppm)

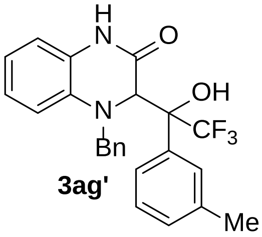

3ag'

^{1}H-NMR ($CDCl_3$, 300 MHz)
^{19}F{^{1}H}-NMR ($CDCl_3$, 282 MHz)
^{13}C{^{1}H}-NMR ($CDCl_3$, 75 MHz)

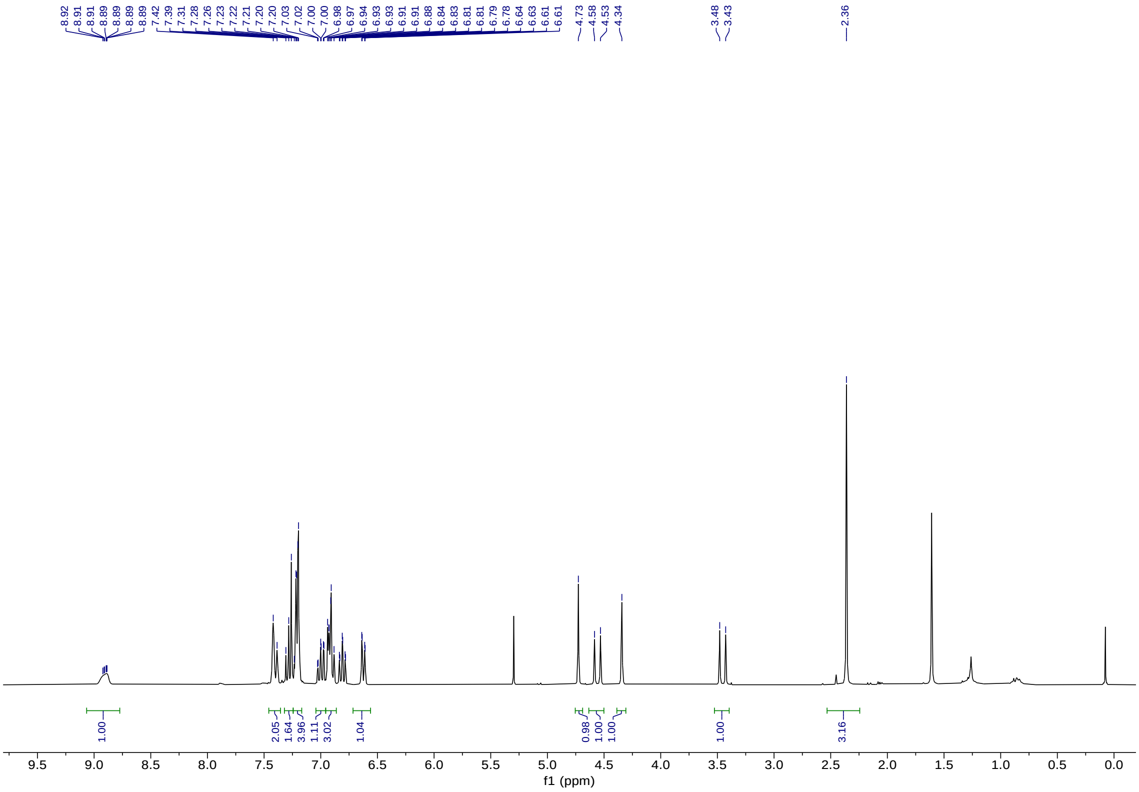

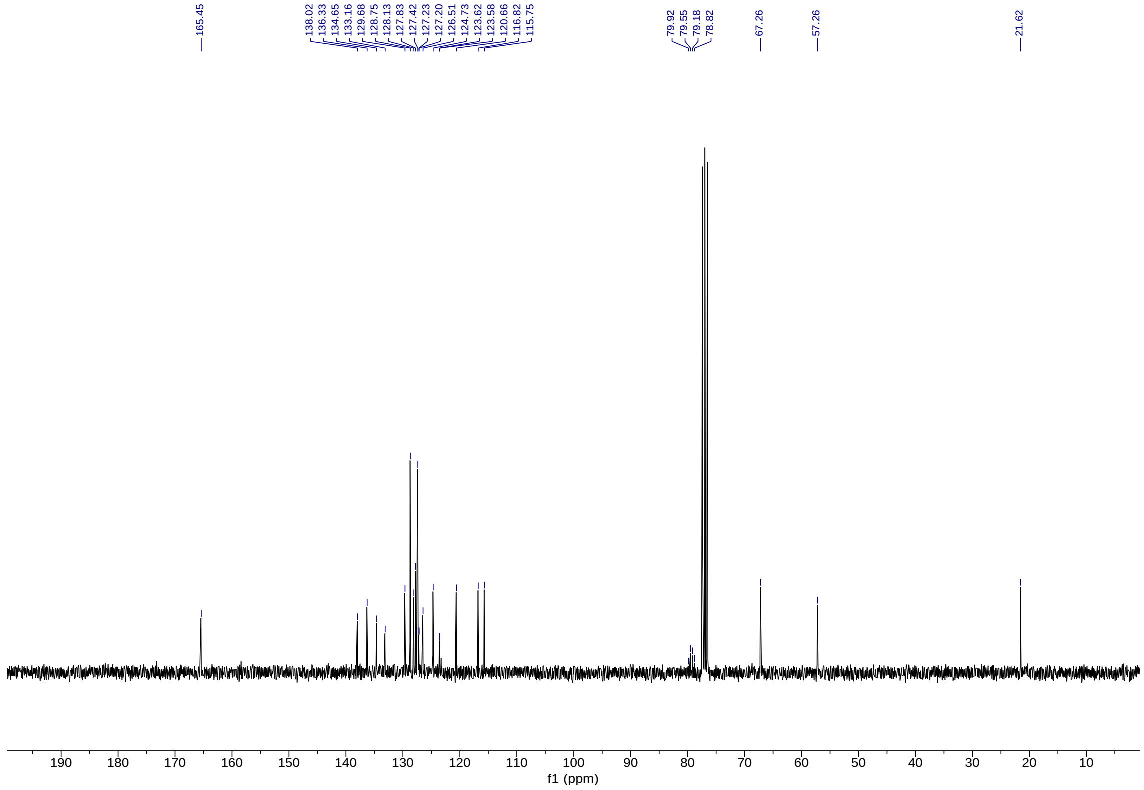
165.45
138.02
136.33
134.65
133.16
129.68
128.75
128.13
127.83
127.42
127.23
127.20
126.51
124.73
123.62
123.58
120.66
116.82
115.75
79.92
79.55
79.18
78.82
67.26
57.26
21.62
190
180
170
160
150
140
130
120
110
100
90
80
70
60
50
40
30
20
10
f1 (ppm)

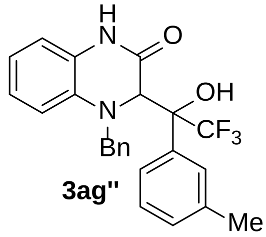

3ag''

^{1}H-NMR ($CDCl_3$, 300 MHz)
^{19}F{^{1}H}-NMR ($CDCl_3$, 282 MHz)
^{13}C{^{1}H}-NMR ($CDCl_3$, 75 MHz)

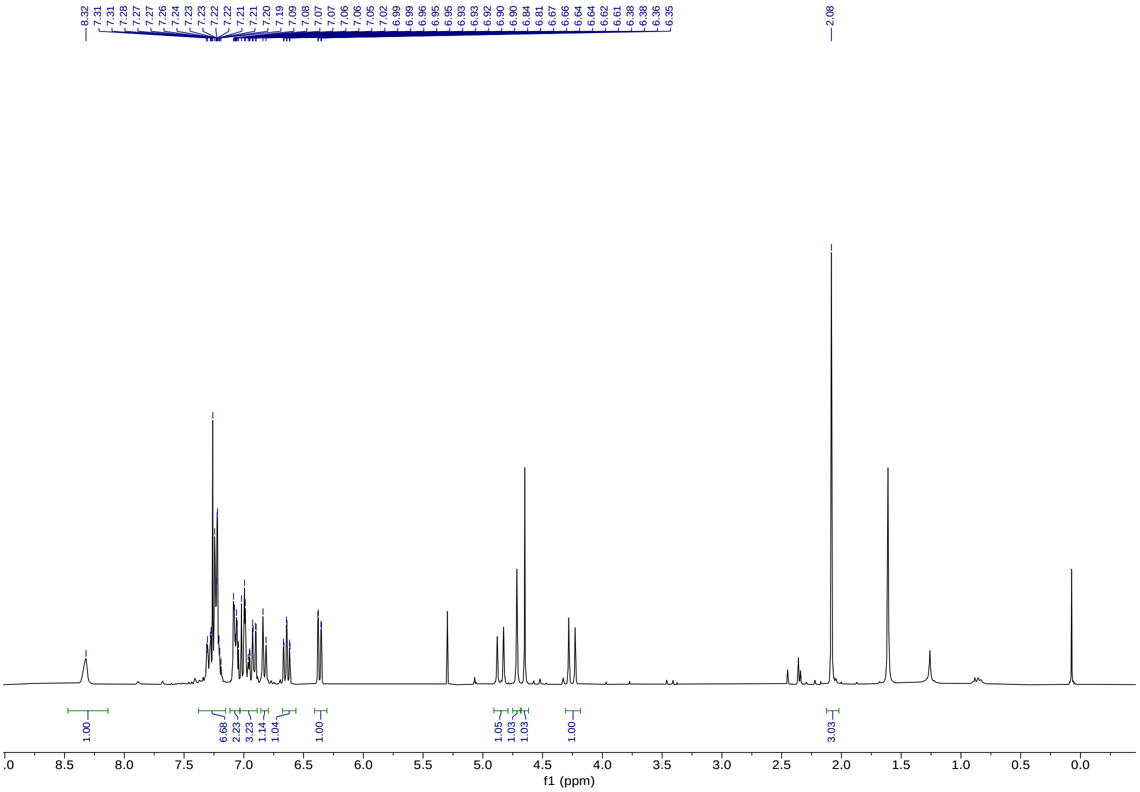

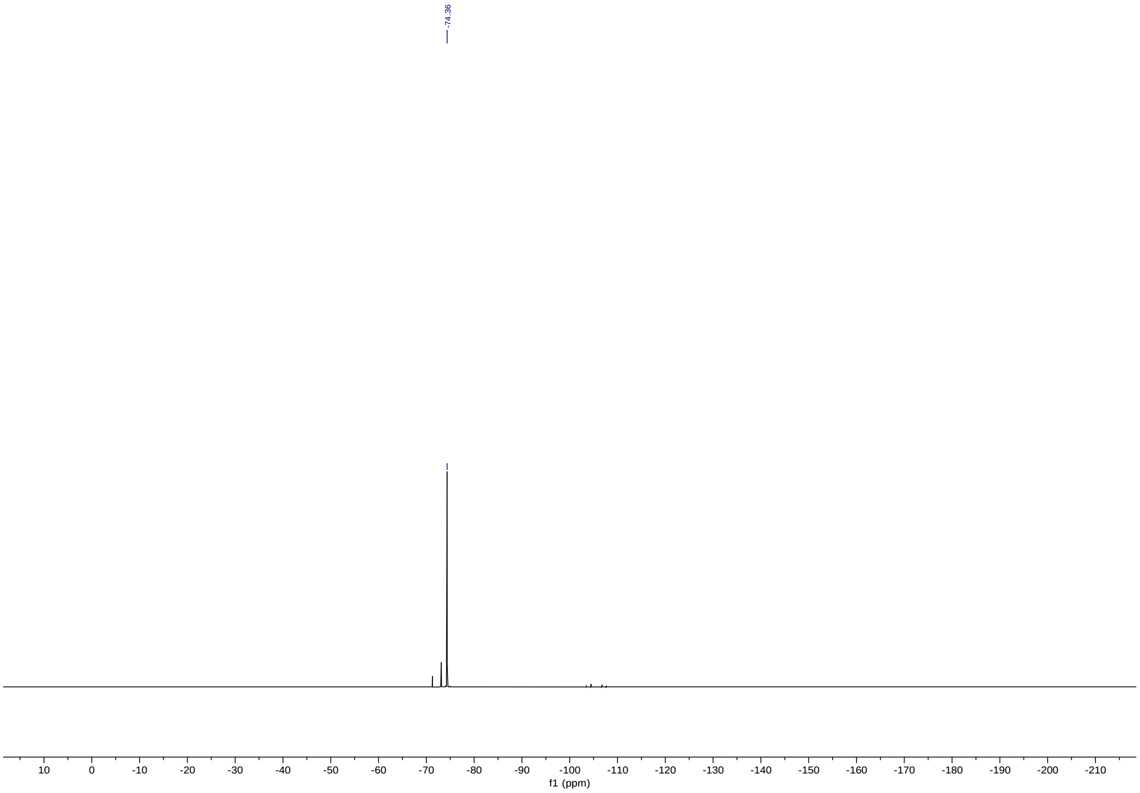

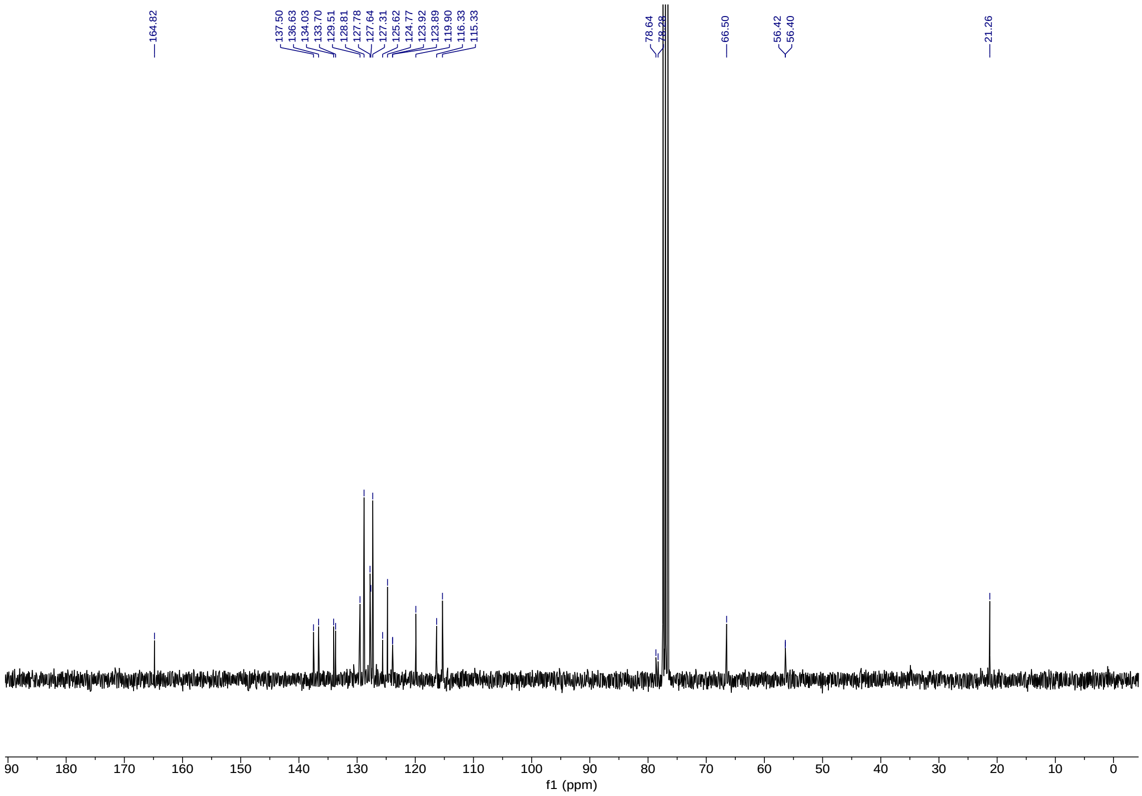
164.82
137.50
136.63
134.03
133.70
129.51
128.81
127.78
127.64
127.31
125.62
124.77
123.92
123.89
119.90
116.33
115.33
78.64
78.28
66.50
56.42
56.40
21.26
f1 (ppm)

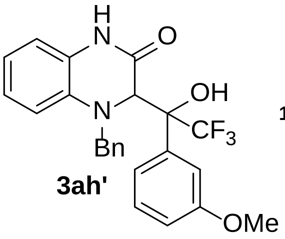

3ah'

^{1}H-NMR ($CDCl_3$, 300 MHz)
^{19}F{^{1}H}-NMR ($CDCl_3$, 282 MHz)
^{13}C{^{1}H}-NMR ($CDCl_3$, 75 MHz)

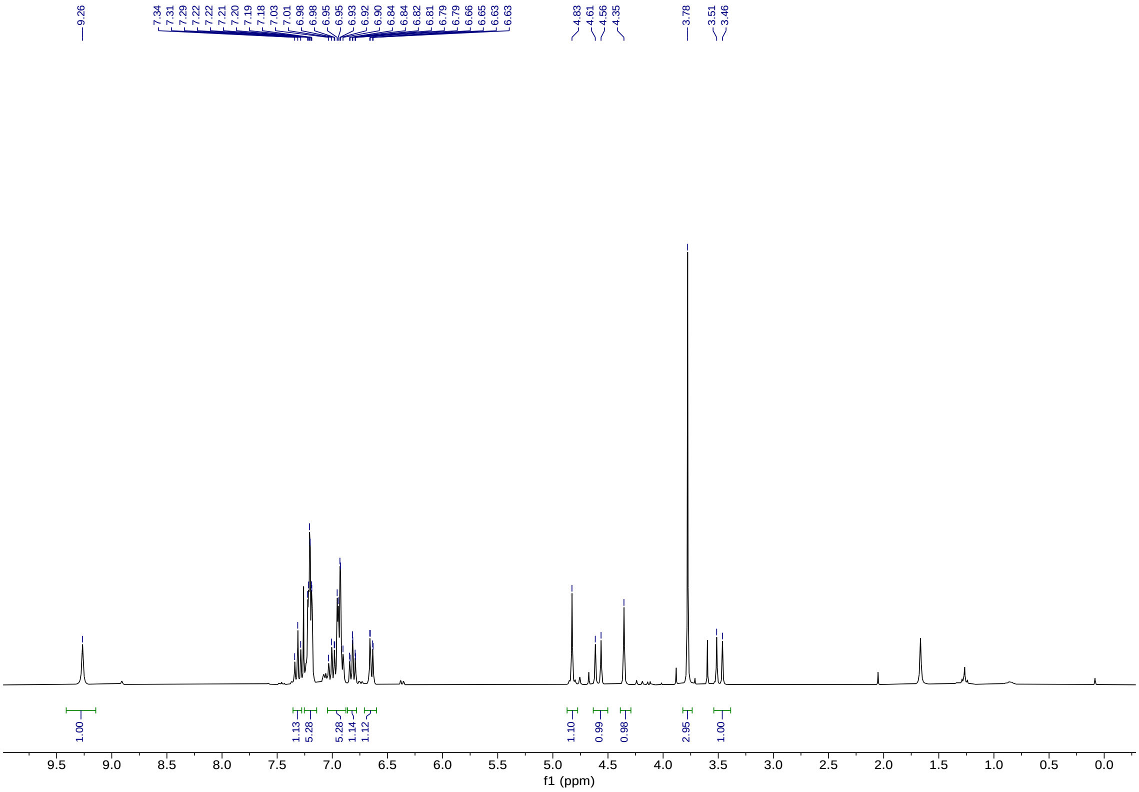

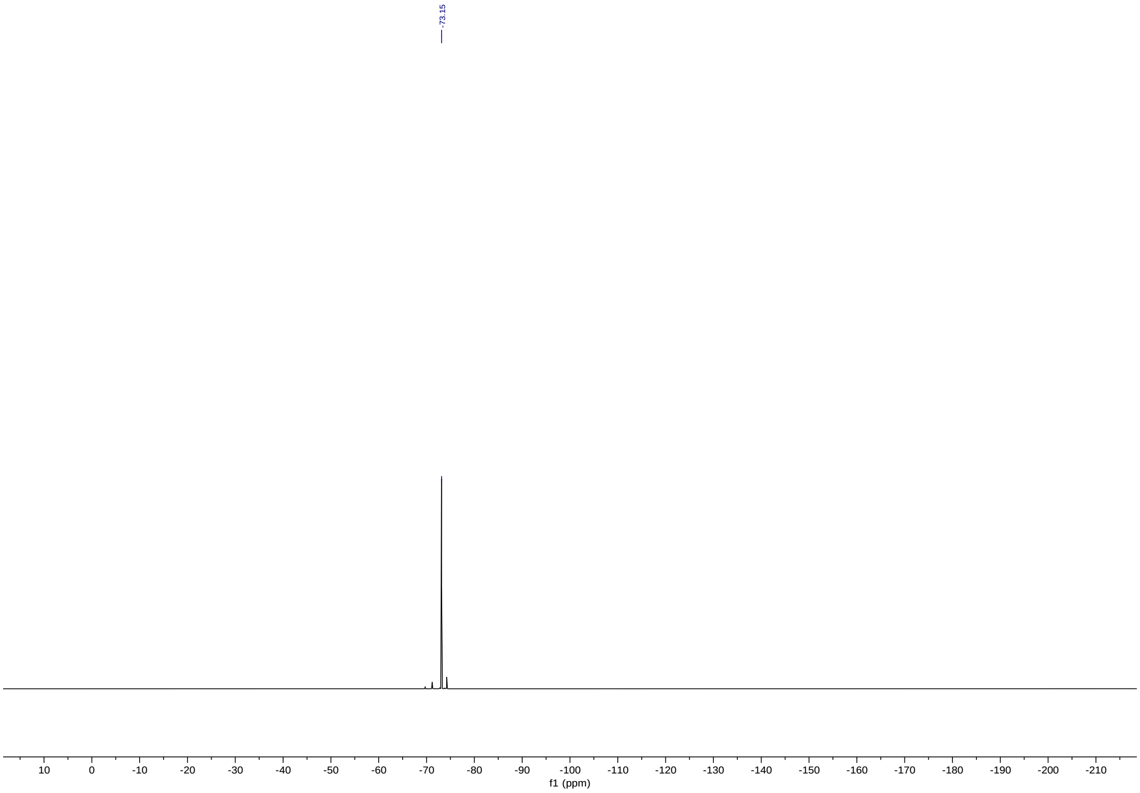

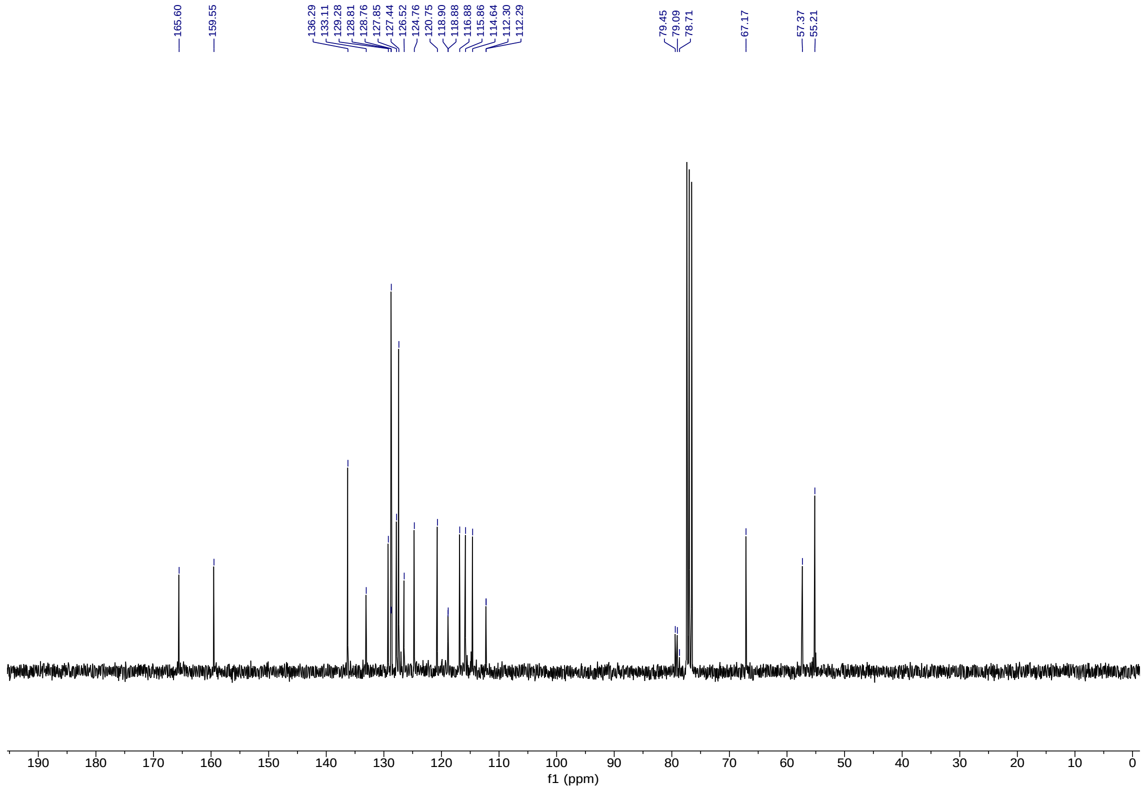
165.60
159.55
136.29
133.11
129.28
128.81
128.76
127.85
127.44
126.52
124.76
120.75
118.90
118.88
116.88
115.86
114.64
112.30
112.29
79.45
79.09
78.71
67.17
57.37
55.21
190
180
170
160
150
140
130
120
110
100
90
80
70
60
50
40
30
20
10
0
f1 (ppm)

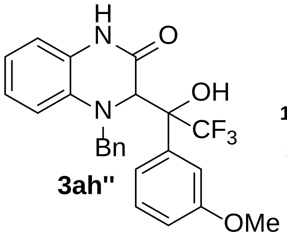

3ah''

^{1}H-NMR ($CDCl_3$, 300 MHz)
^{19}F{^{1}H}-NMR ($CDCl_3$, 282 MHz)
^{13}C{^{1}H}-NMR ($CDCl_3$, 75 MHz)

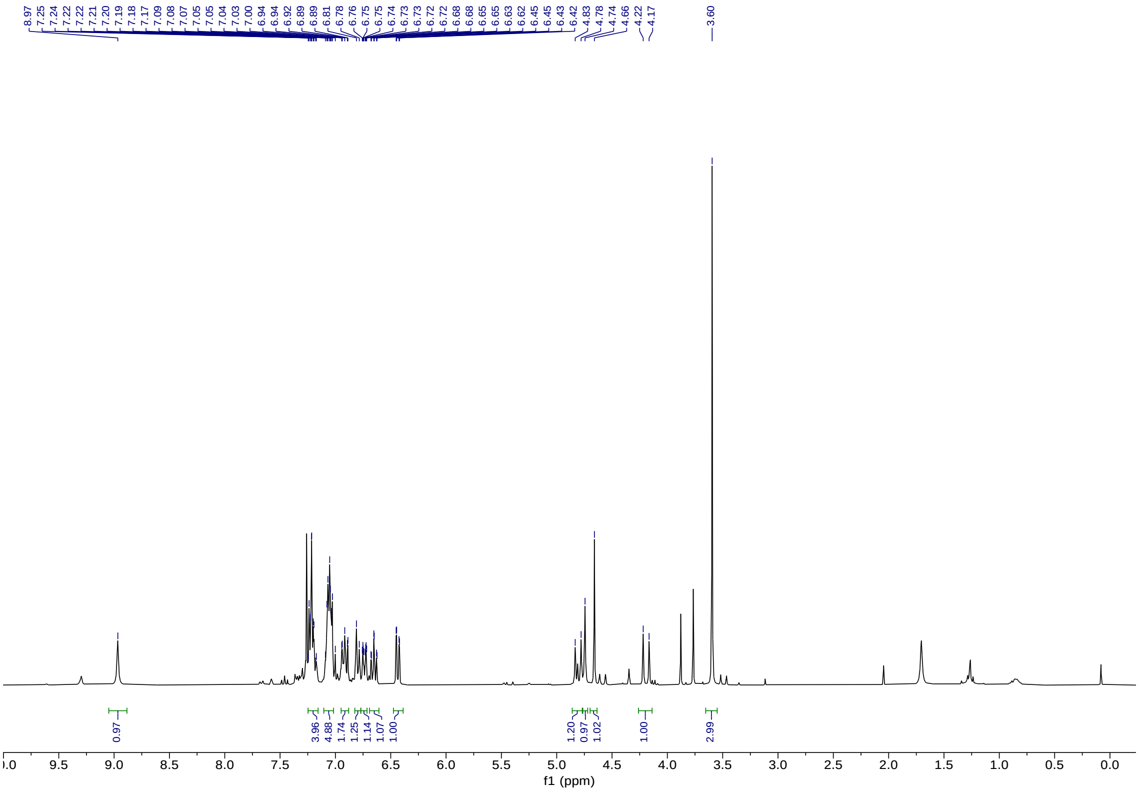

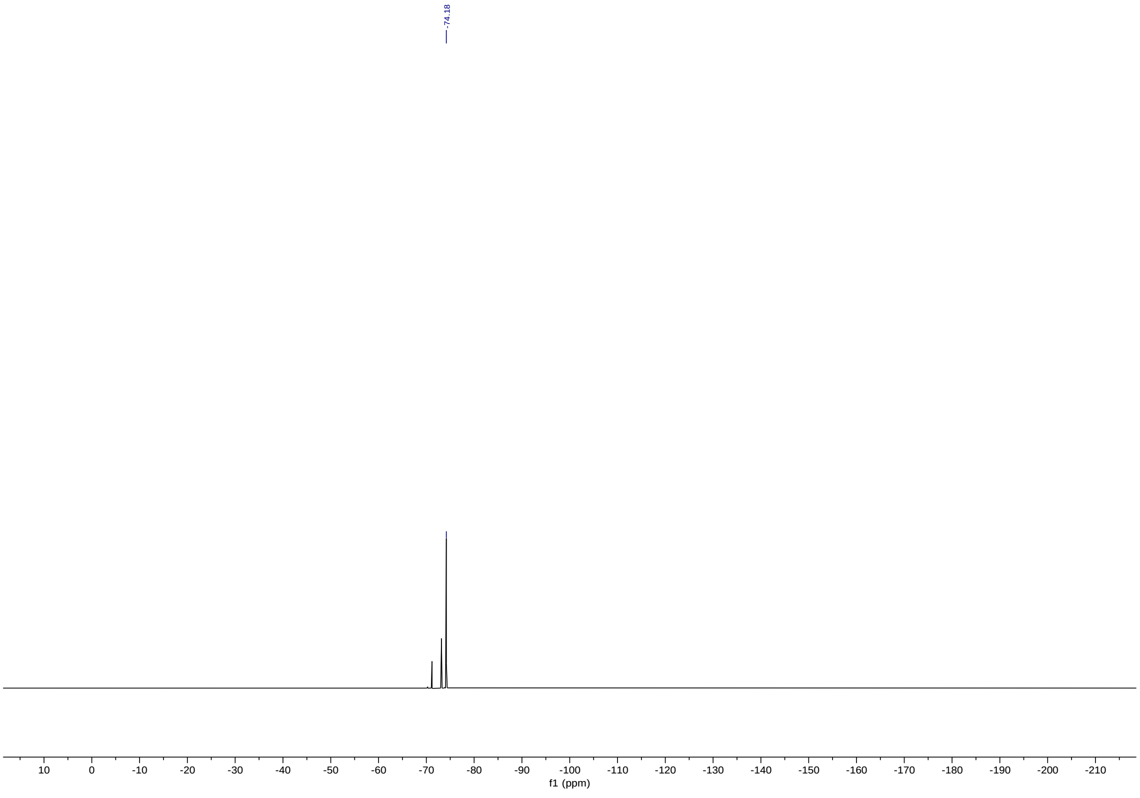

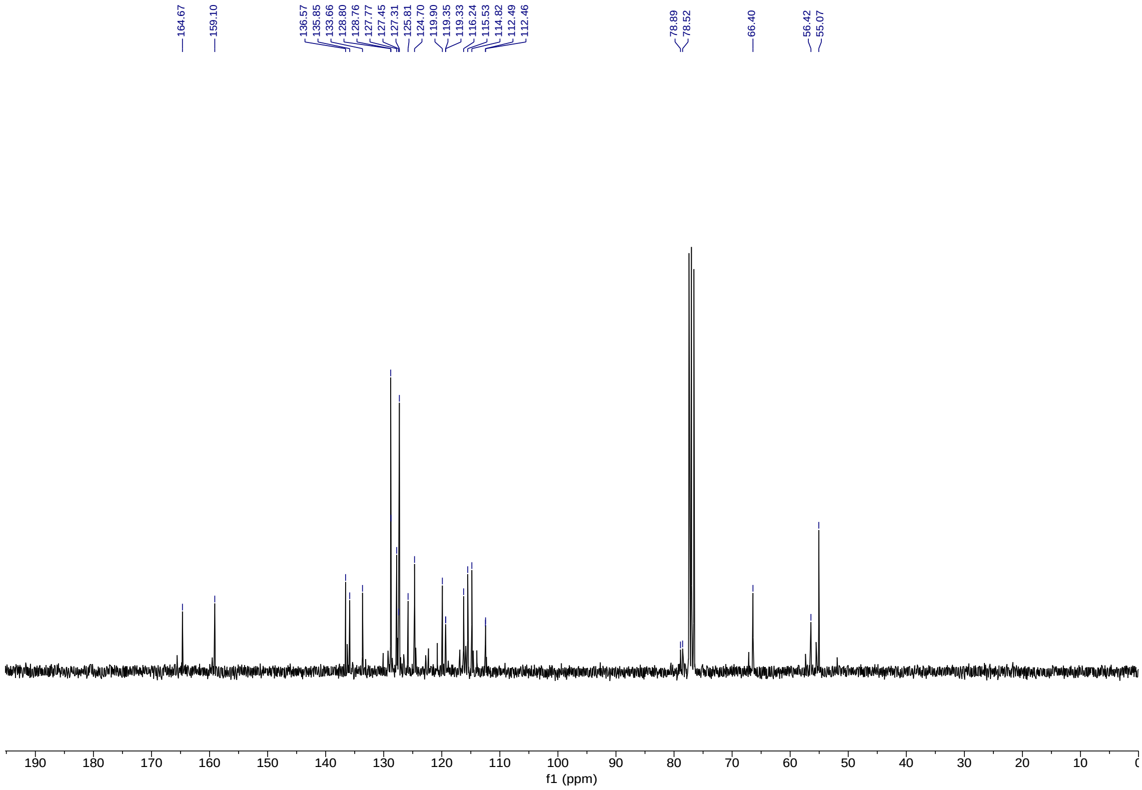
164.67
159.10
136.57
135.85
133.66
128.80
128.76
127.77
127.45
127.31
125.81
124.70
119.90
119.35
119.33
116.24
115.53
114.82
112.49
112.46
78.89
78.52
66.40
56.42
55.07
190 180 170 160 150 140 130 120 110 100 90 80 70 60 50 40 30 20 10 0
f1 (ppm)

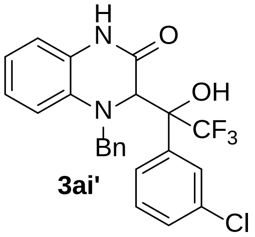

3ai'

^{1}H-NMR ($CDCl_3$, 300 MHz)
^{19}F{^{1}H}-NMR ($CDCl_3$, 282 MHz)
^{13}C{^{1}H}-NMR ($CDCl_3$, 75 MHz)

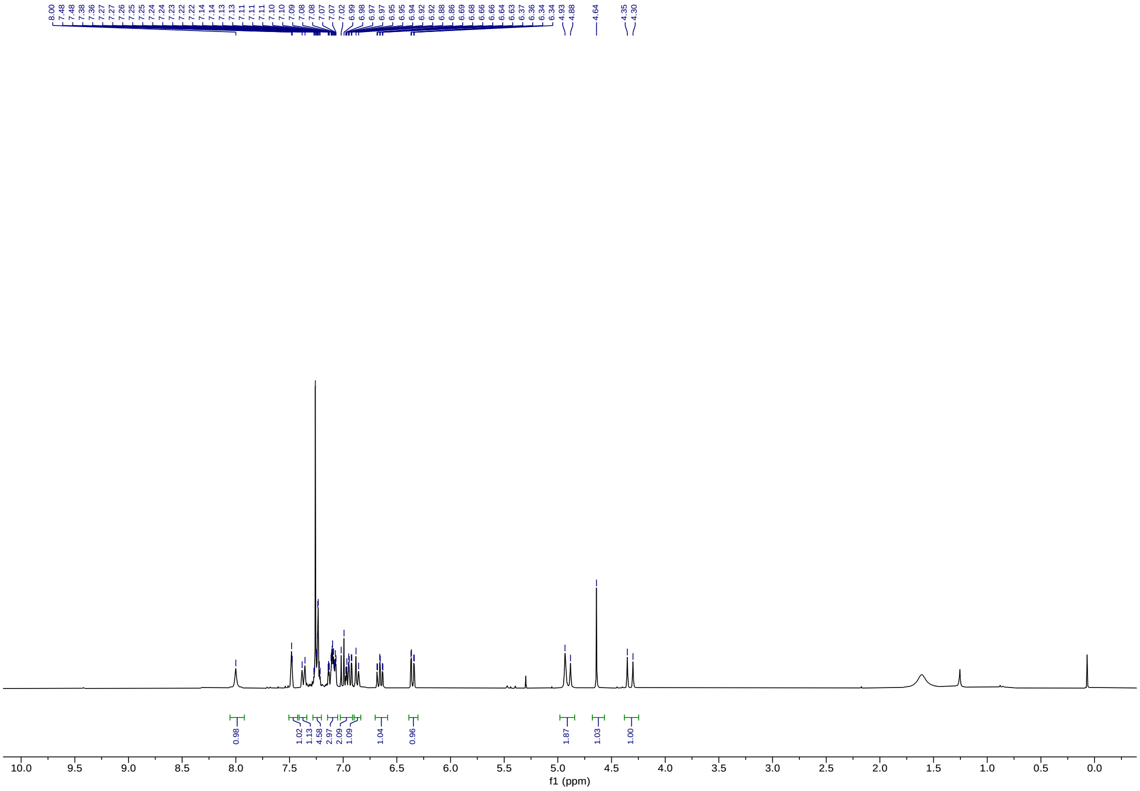

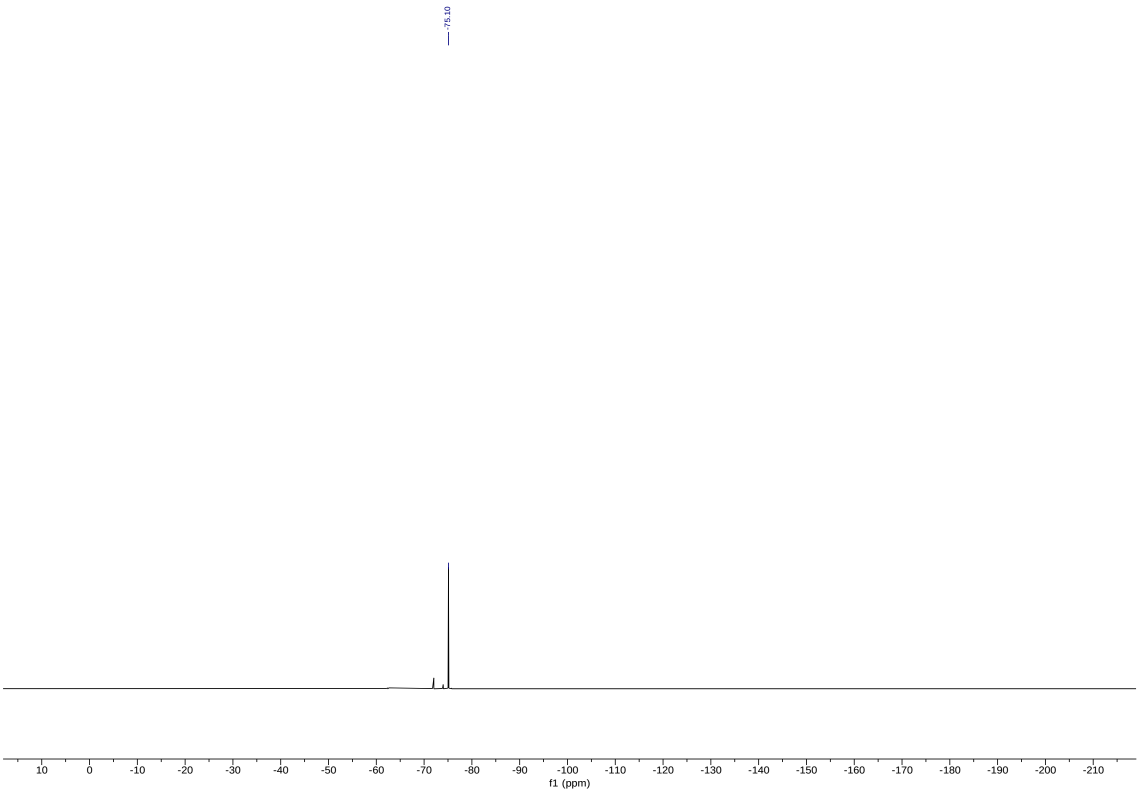

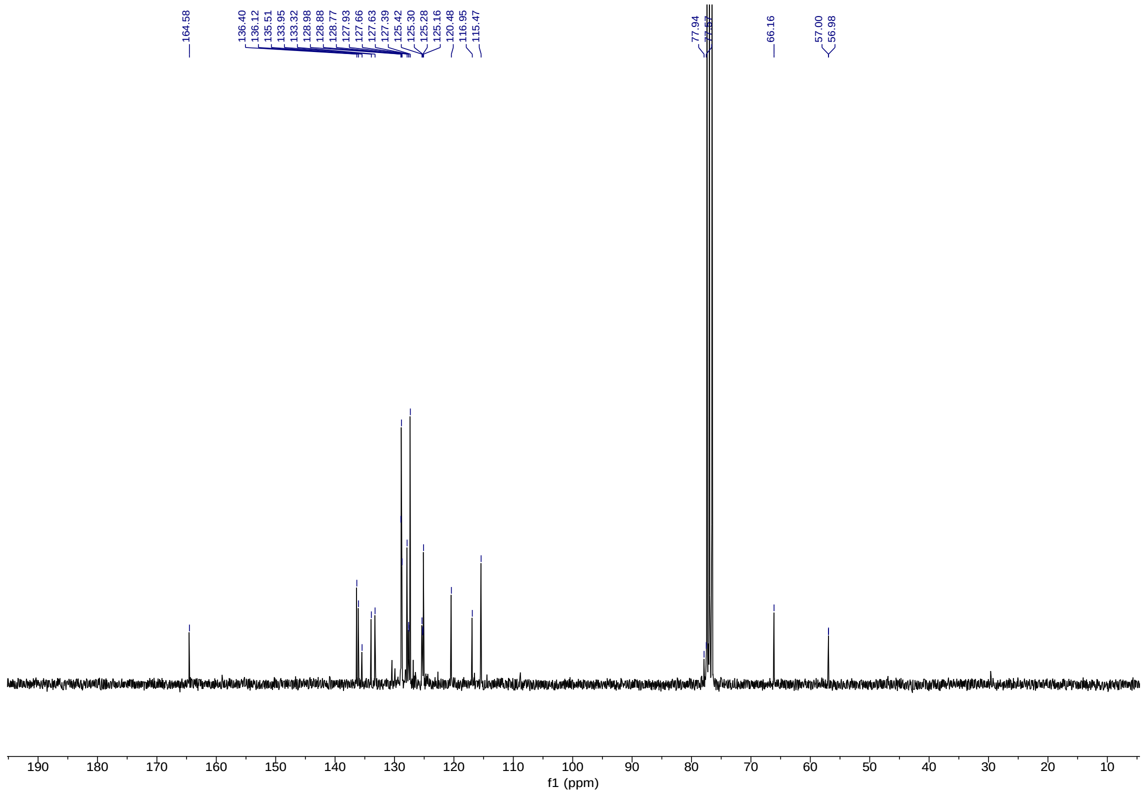
164.58
136.40
136.12
135.51
133.95
133.32
128.98
128.88
128.77
127.93
127.66
127.63
127.39
125.42
125.30
125.28
125.16
120.48
116.95
115.47
77.94
66.16
57.00
56.98
f1 (ppm)

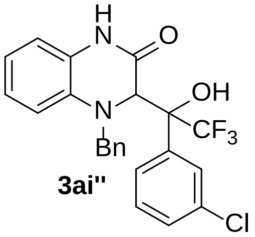

3ai''

^{1}H-NMR ($CDCl_3$, 300 MHz)
$^{19}F\{^{1}H\}$-NMR ($CDCl_3$, 282 MHz)
$^{13}C\{^{1}H\}$-NMR ($CDCl_3$, 75 MHz)

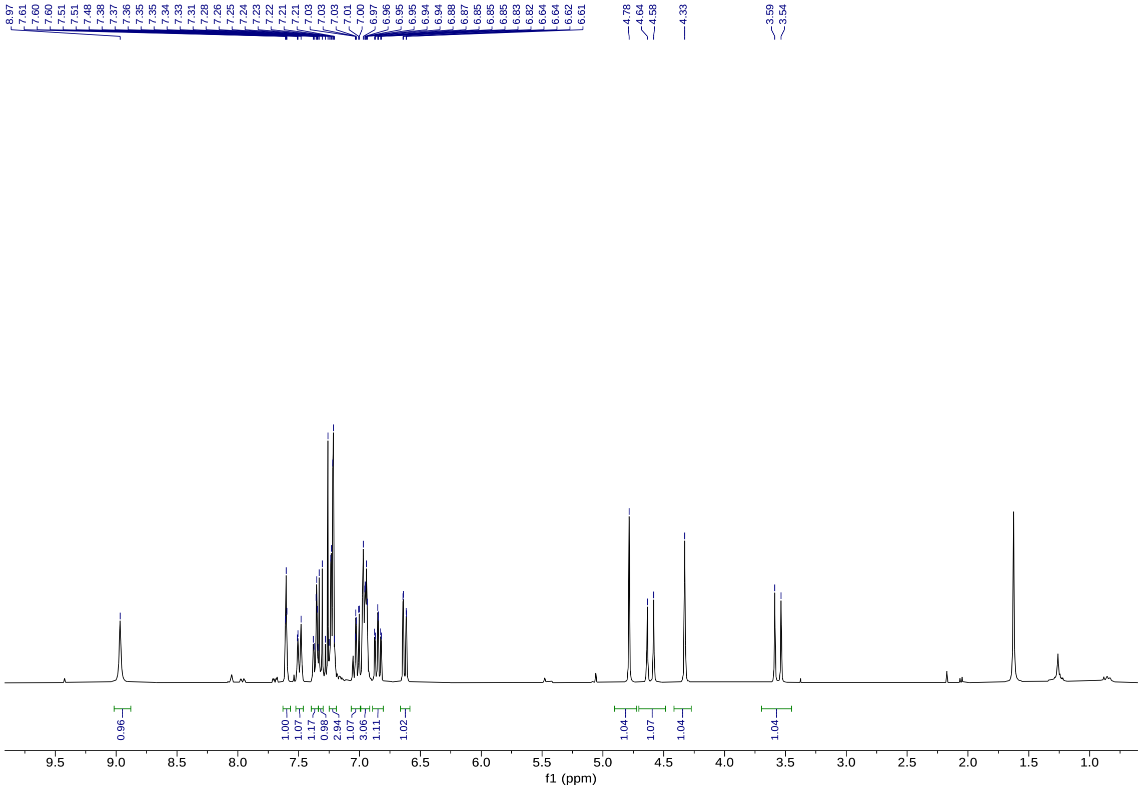

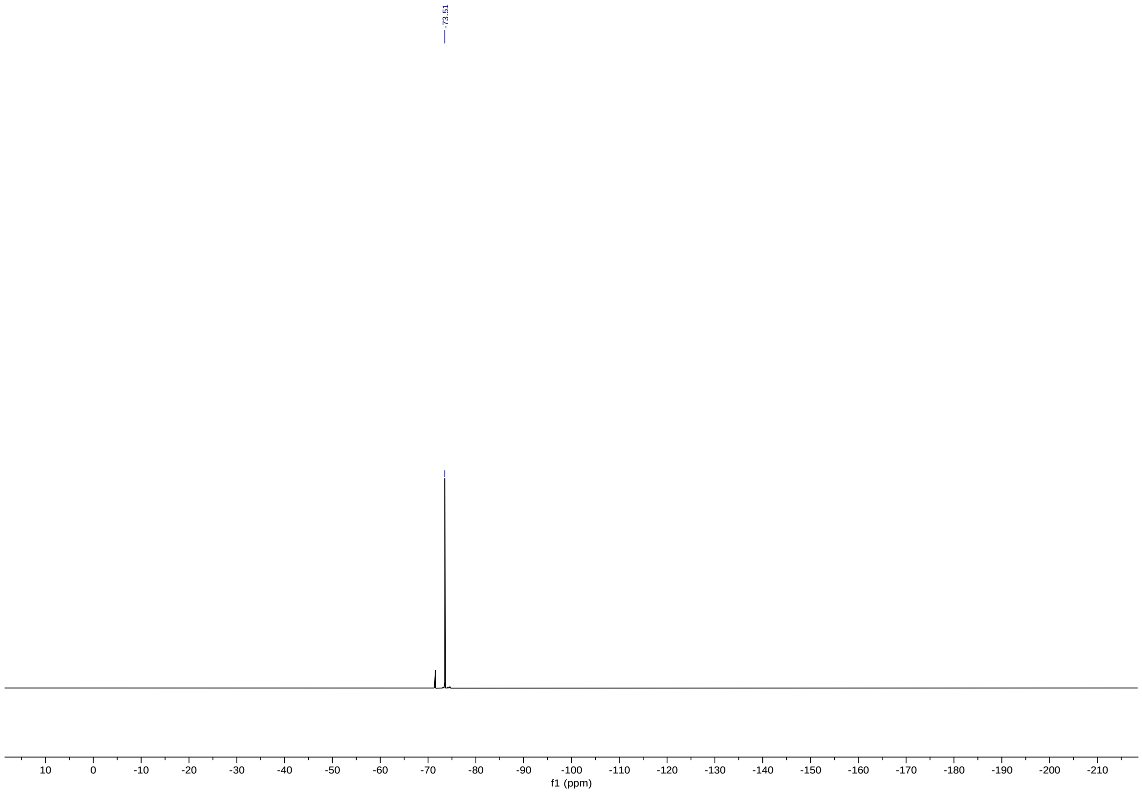

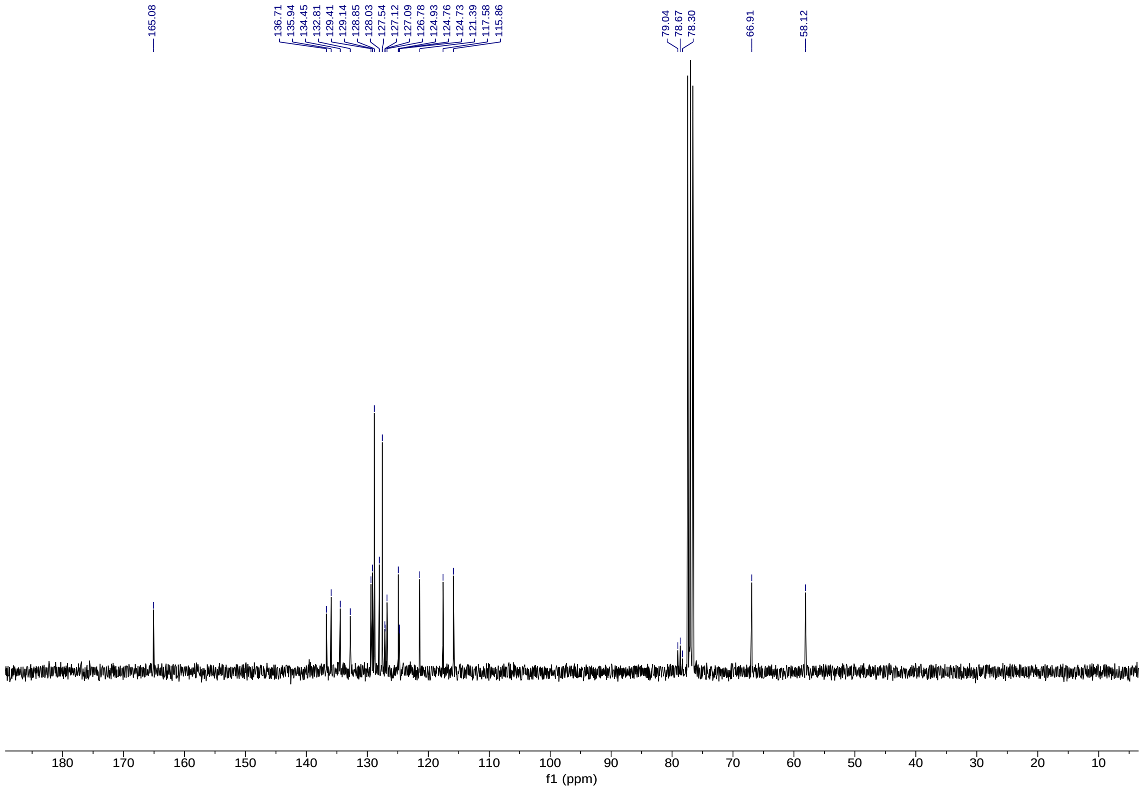
165.08
136.71
135.94
134.45
132.81
129.41
129.14
128.85
128.03
127.54
127.12
127.09
126.78
124.93
124.76
124.73
121.39
117.58
115.86
79.04
78.67
78.30
66.91
58.12
180 170 160 150 140 130 120 110 100 90 80 70 60 50 40 30 20 10
f1 (ppm)

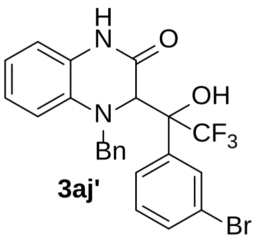

3aj'

^{1}H-NMR (CDCl$_3$, 300 MHz)
^{19}F{^{1}H}-NMR (CDCl$_3$, 282 MHz)
^{13}C{^{1}H}-NMR (CDCl$_3$, 75 MHz)

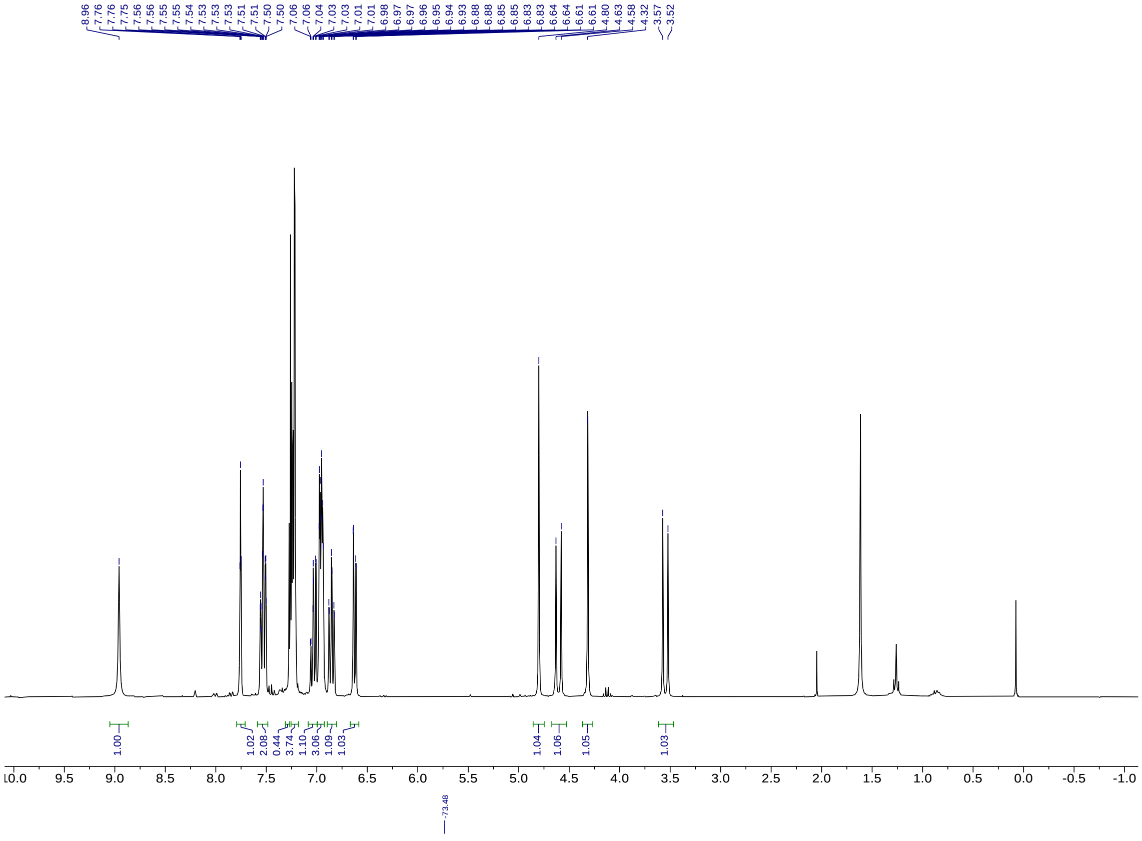

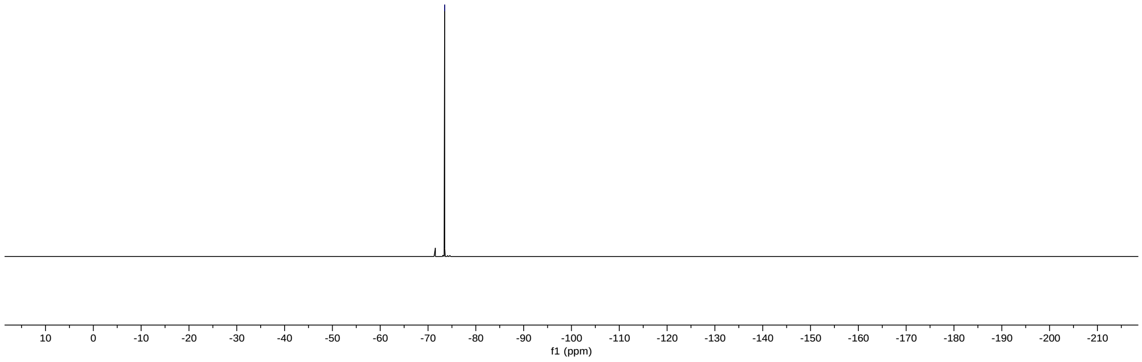

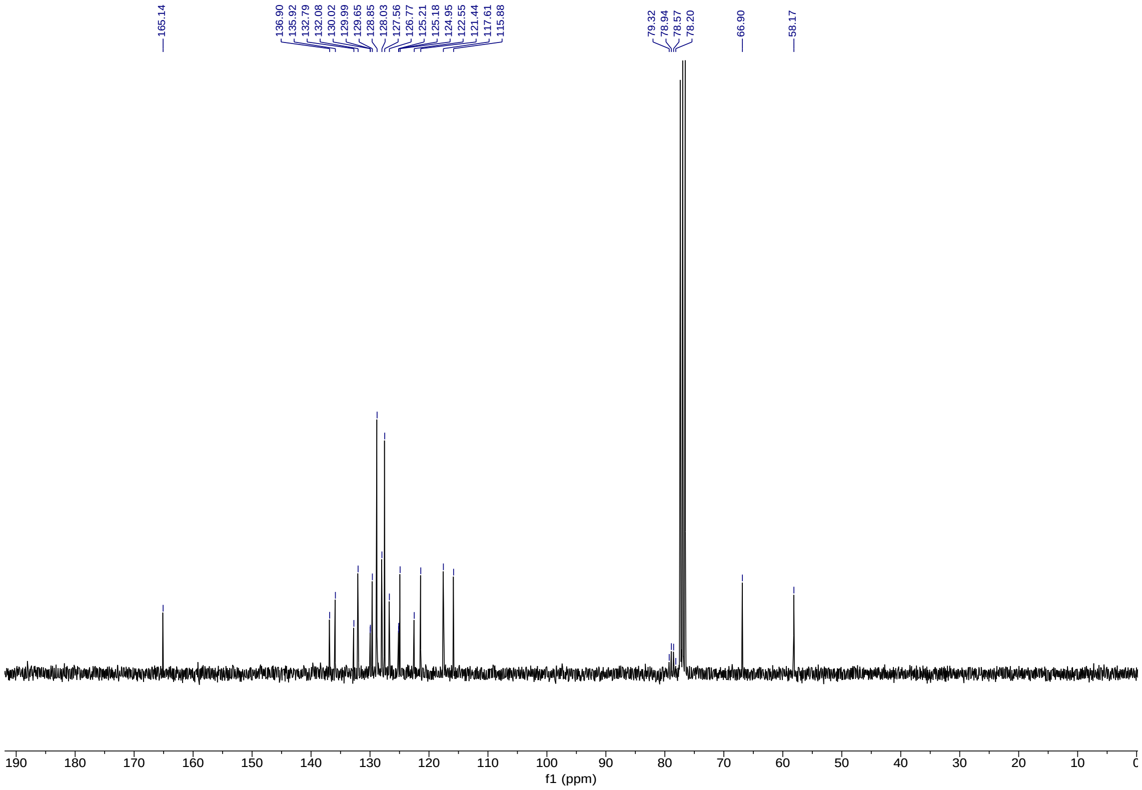
165.14
136.90
135.92
132.79
132.08
130.02
129.99
129.65
128.85
128.03
127.56
126.77
125.21
125.18
124.95
122.55
121.44
117.61
115.88
79.32
78.94
78.57
78.20
66.90
58.17
190 180 170 160 150 140 130 120 110 100 90 80 70 60 50 40 30 20 10 0
f1 (ppm)

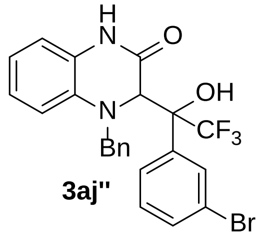

3aj''

^{1}H-NMR ($CDCl_3$, 300 MHz)
$^{19}F\{^{1}H\}$-NMR ($CDCl_3$, 282 MHz)
$^{13}C\{^{1}H\}$-NMR ($CDCl_3$, 75 MHz)

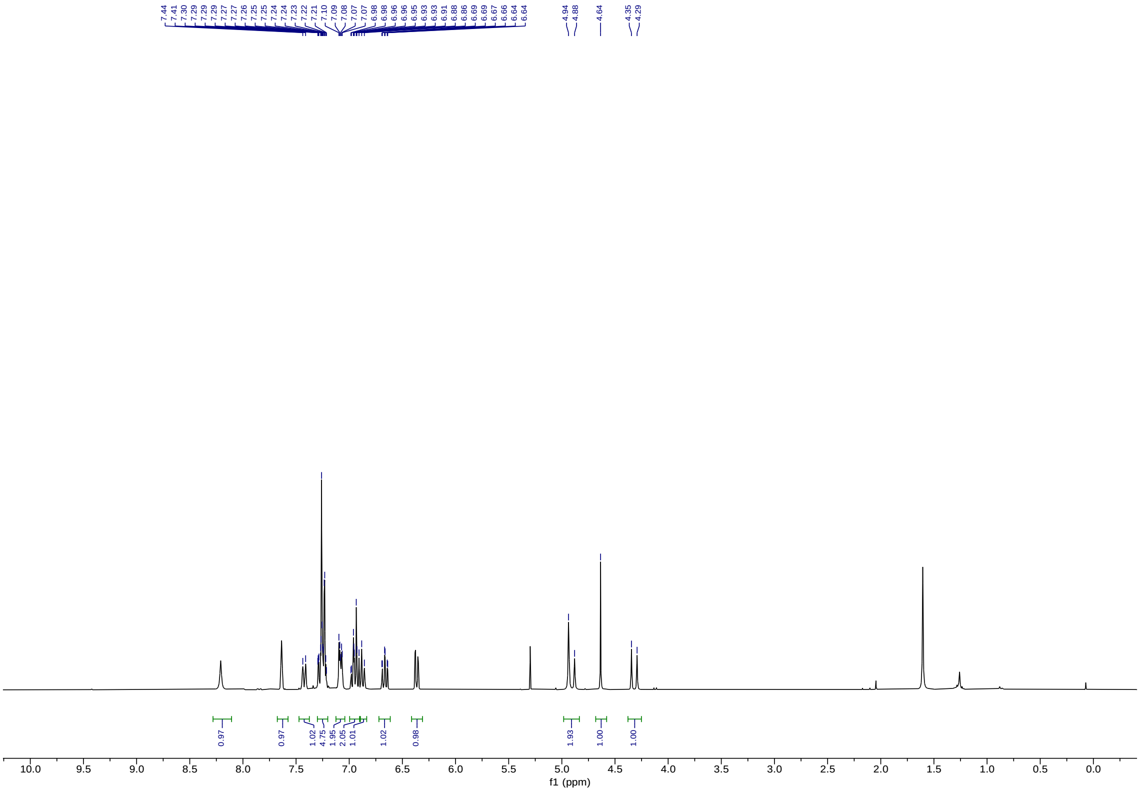

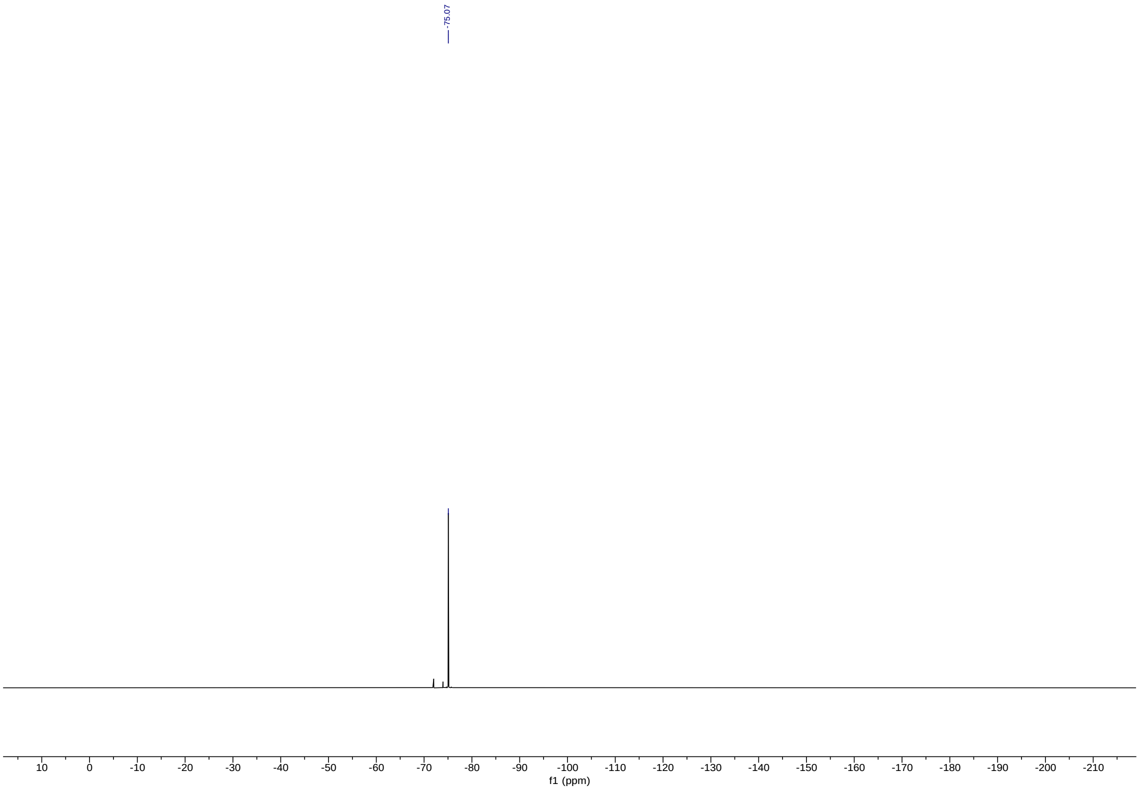

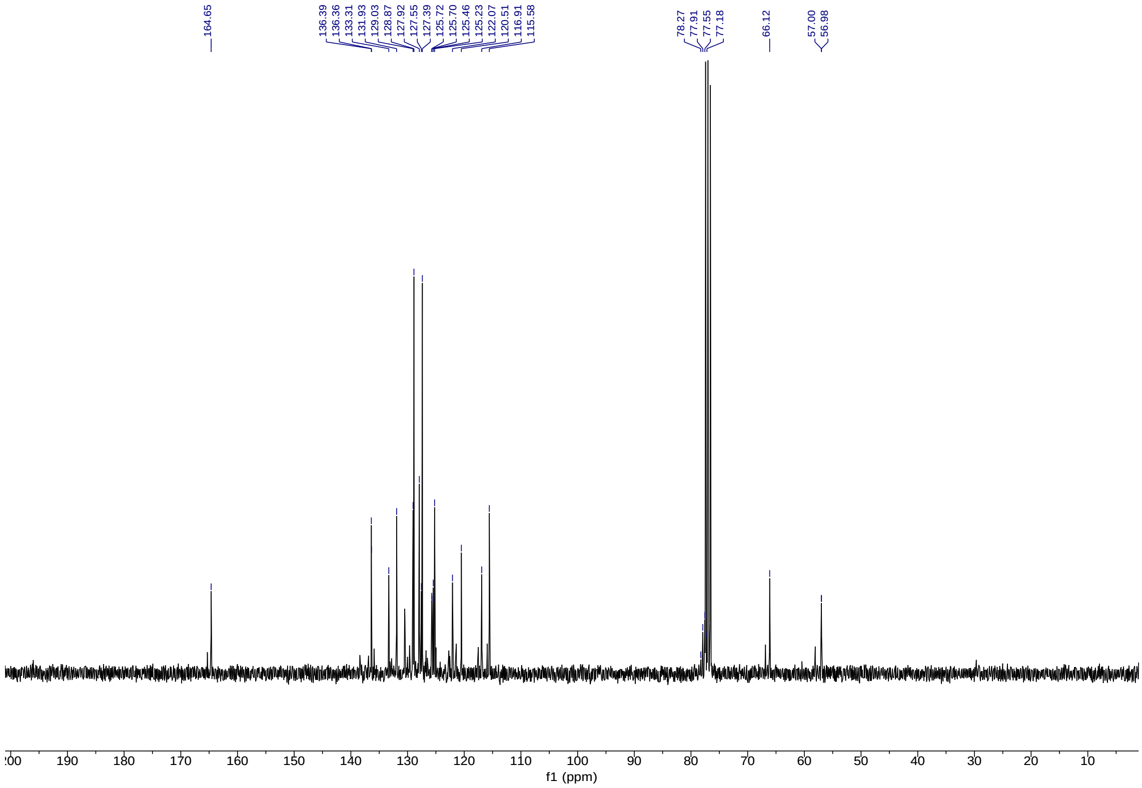
164.65
136.39
136.36
133.31
131.93
129.03
128.87
127.92
127.55
127.39
125.72
125.70
125.46
125.23
122.07
120.51
116.91
115.58
78.27
77.91
77.55
77.18
66.12
57.00
56.98
f1 (ppm)

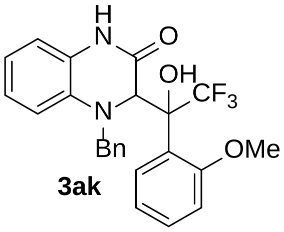

3ak

^{1}H-NMR ($CDCl_3$, 300 MHz)
^{19}F{^{1}H}-NMR ($CDCl_3$, 282 MHz)
^{13}C{^{1}H}-NMR ($CDCl_3$, 75 MHz)

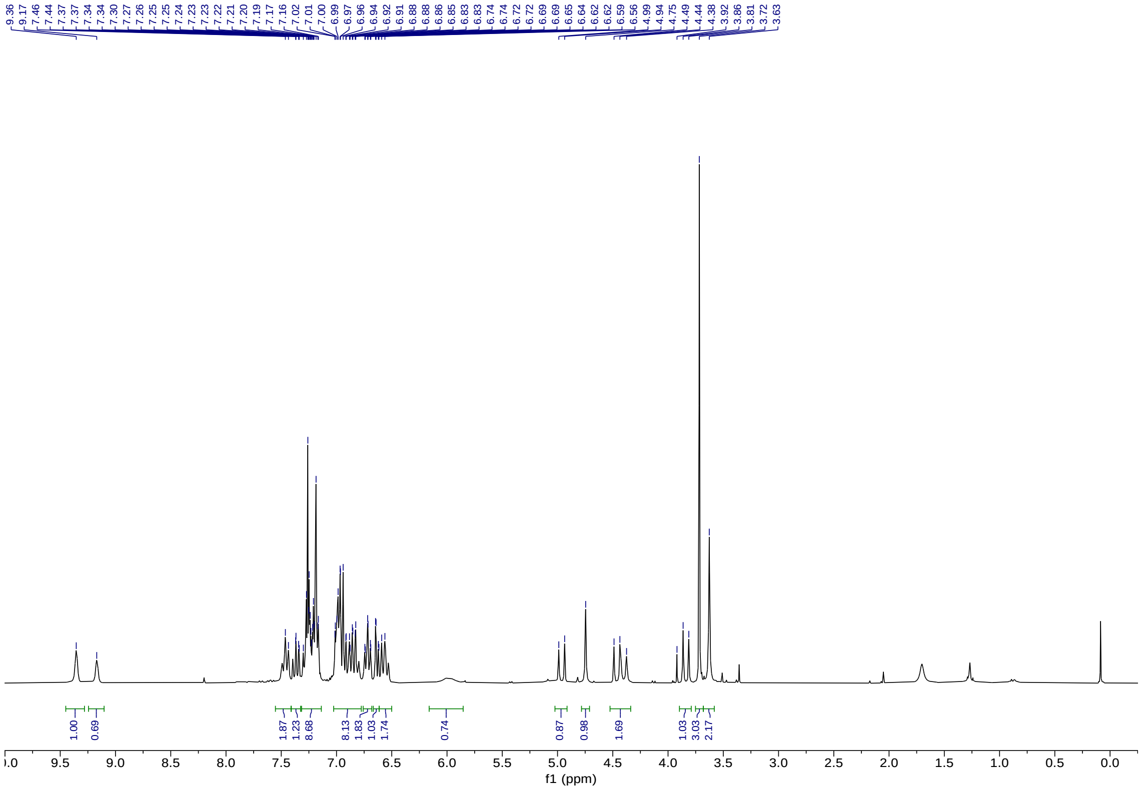

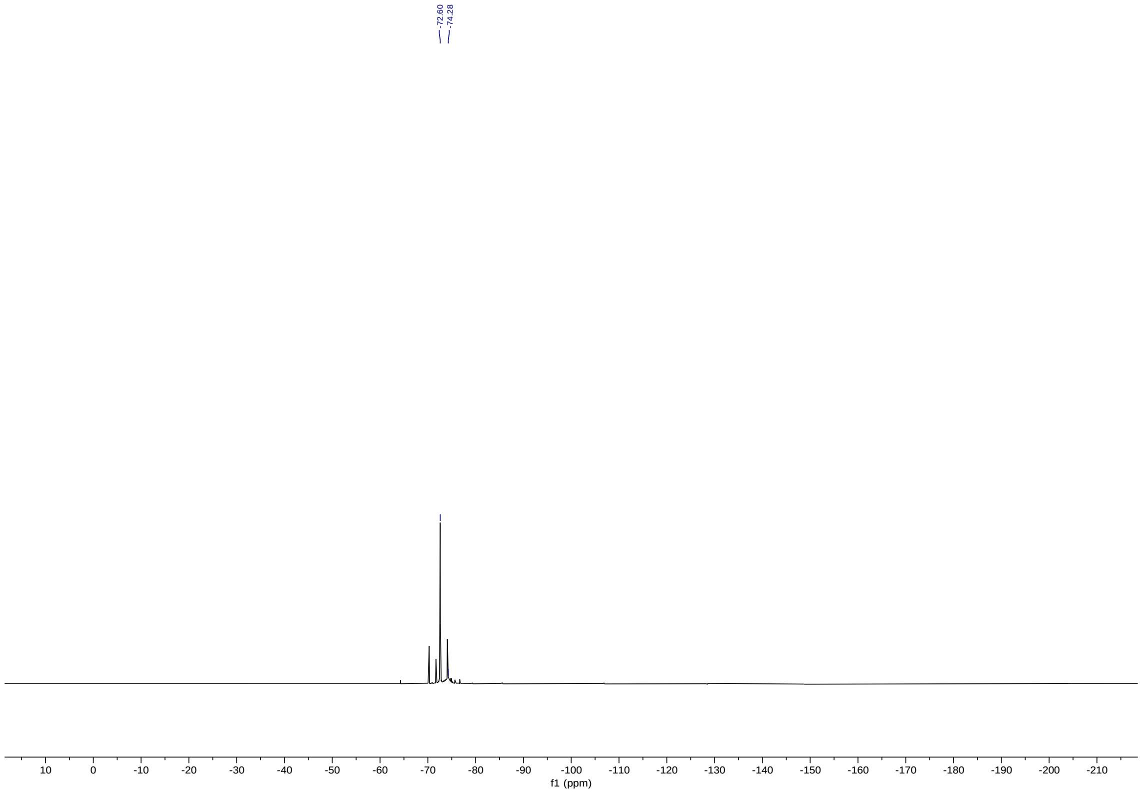

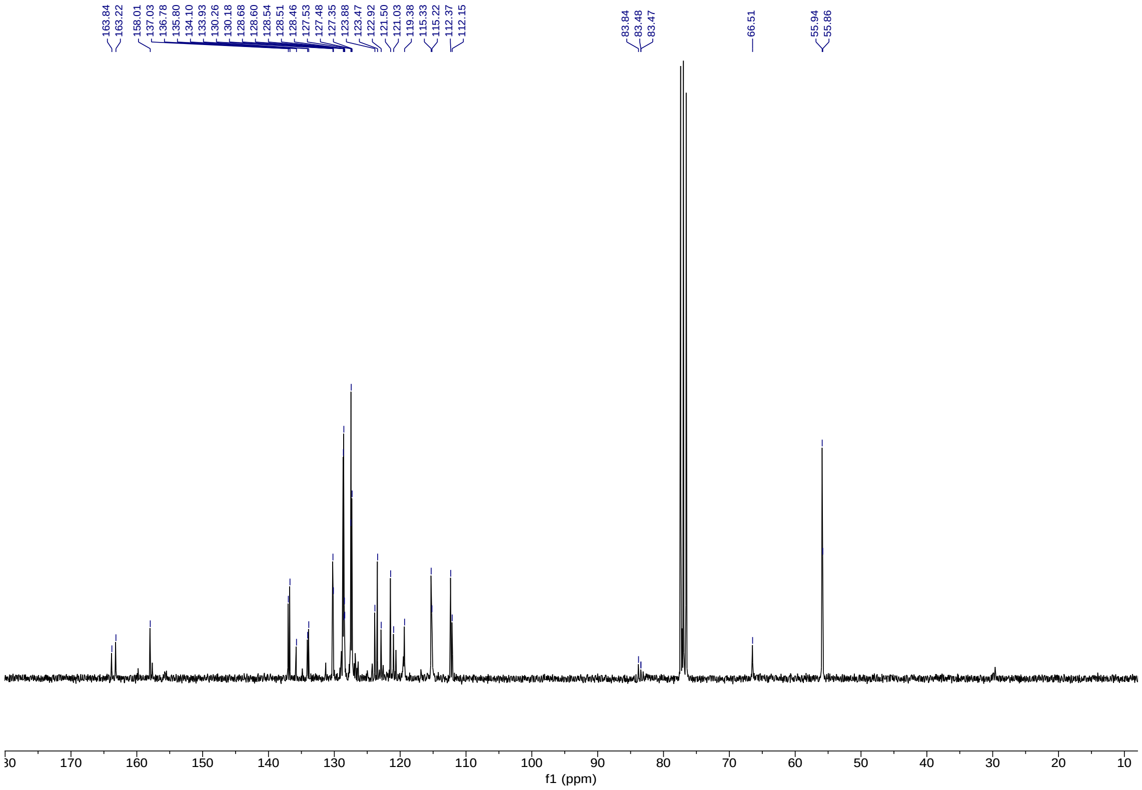

163.84
163.22
158.01
137.03
136.78
135.80
134.10
133.93
130.26
130.18
128.68
128.60
128.54
128.51
128.46
127.53
127.48
127.35
123.88
123.47
122.92
121.50
121.03
119.38
115.33
115.22
112.37
112.15
83.84
83.48
83.47
66.51
55.94
55.86
170
160
150
140
130
120
110
100
90
80
70
60
50
40
30
20
10
f1 (ppm)

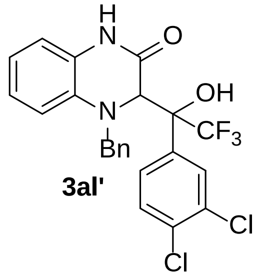

3al'

^{1}H-NMR ($CDCl_3$, 300 MHz)
^{19}F{^{1}H}-NMR ($CDCl_3$, 282 MHz)
^{13}C{^{1}H}-NMR ($CDCl_3$, 75 MHz)

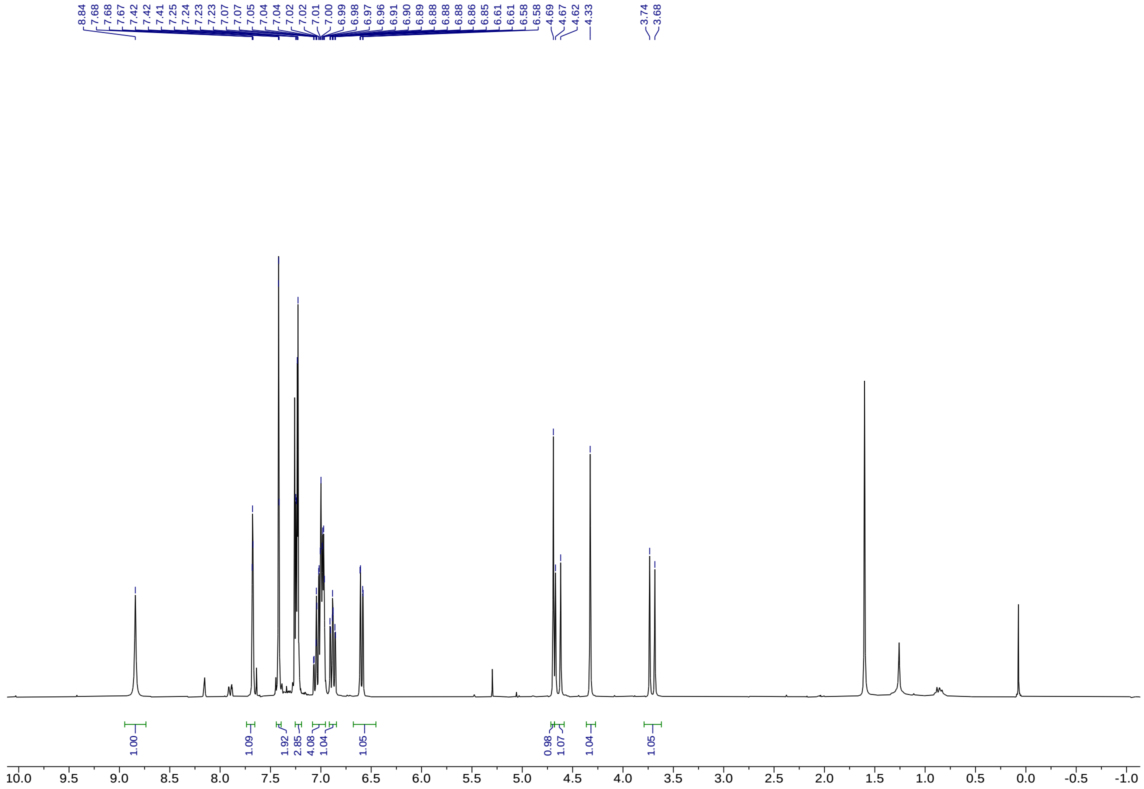

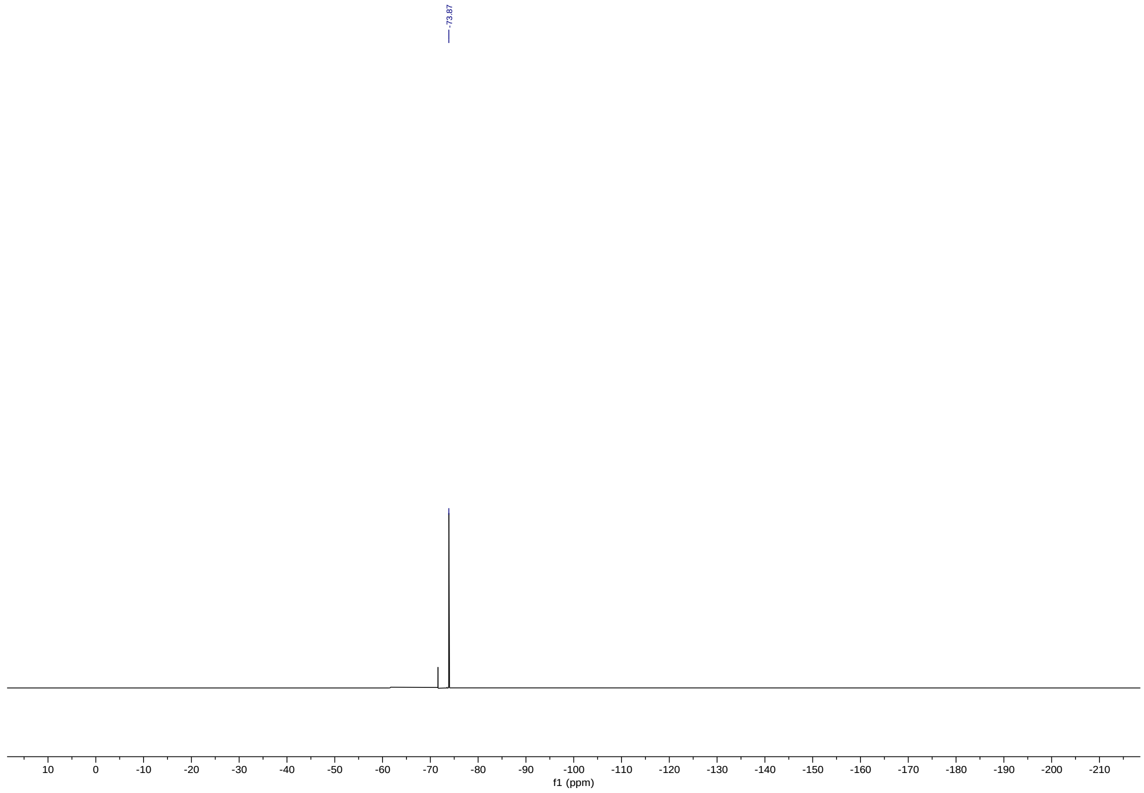

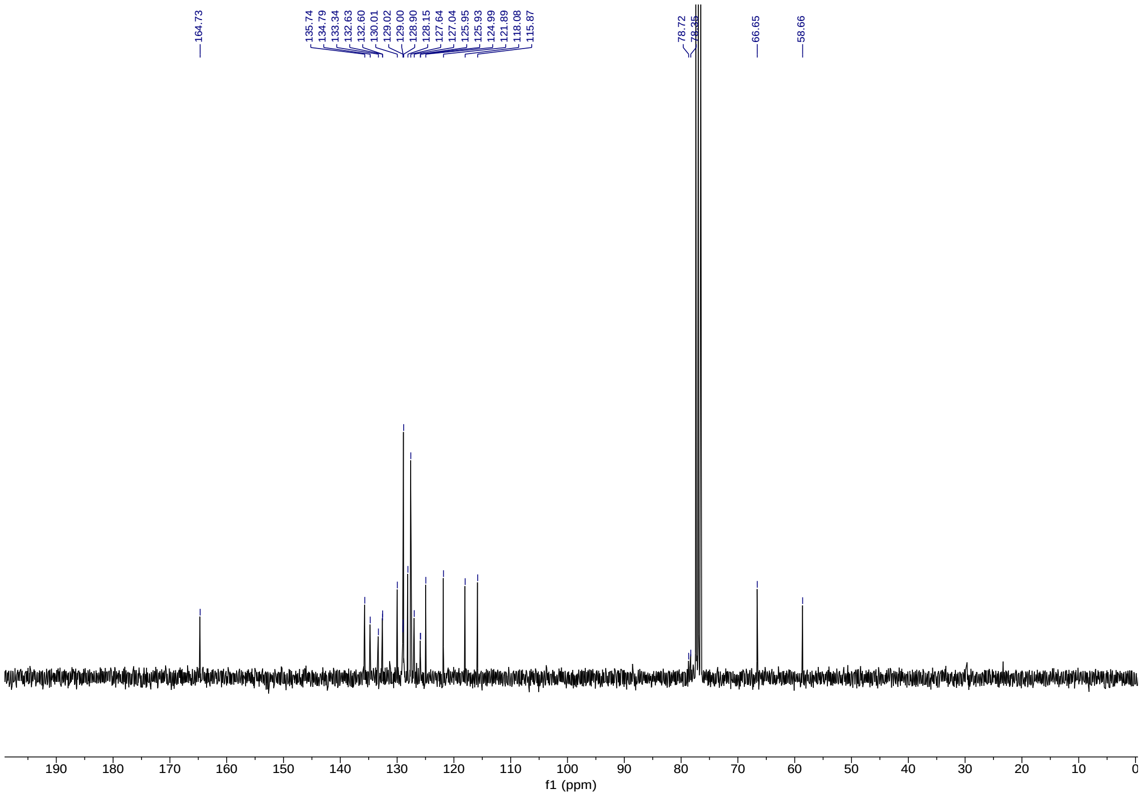
164.73
135.74
134.79
133.34
132.63
132.60
130.01
129.02
129.00
128.90
128.15
127.64
127.04
125.95
125.93
124.99
121.89
118.08
115.87
78.72
78.35
66.65
58.66
190
180
170
160
150
140
130
120
110
100
90
80
70
60
50
40
30
20
10
0
f1 (ppm)

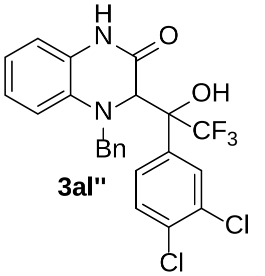

3al''

^{1}H-NMR ($CDCl_3$, 300 MHz)
^{19}F{^{1}H}-NMR ($CDCl_3$, 282 MHz)
^{13}C{^{1}H}-NMR ($CDCl_3$, 75 MHz)

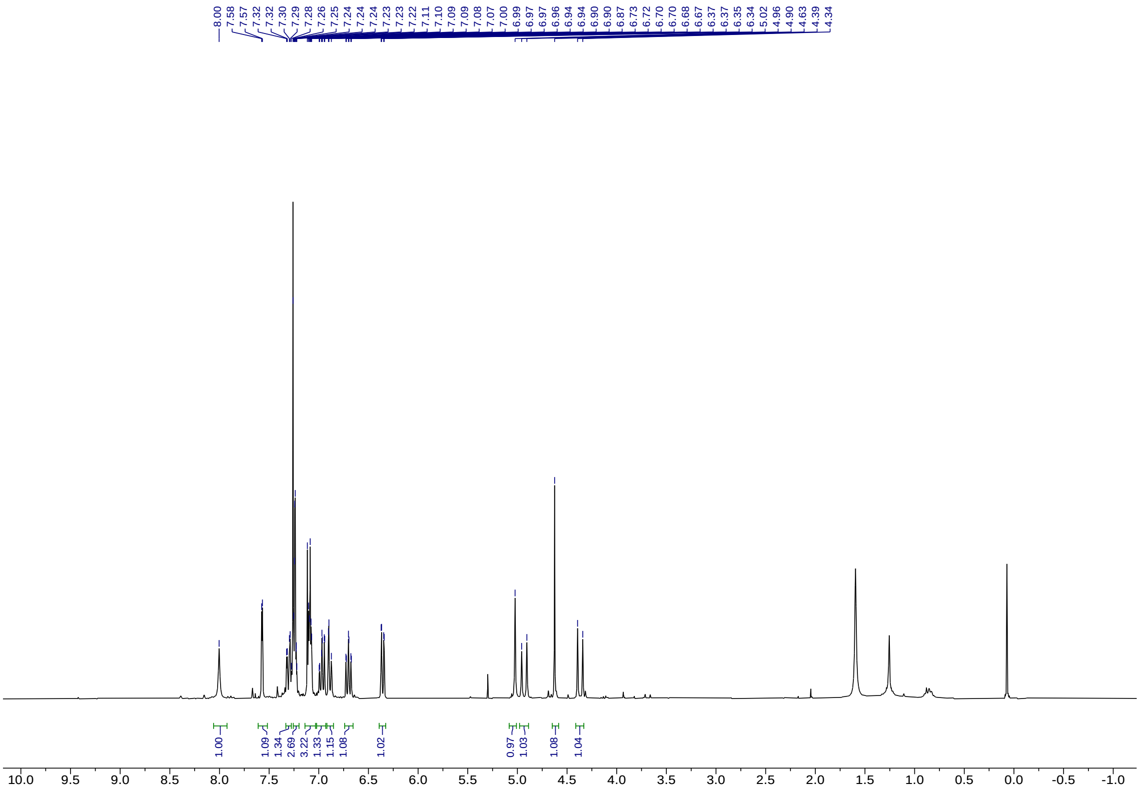

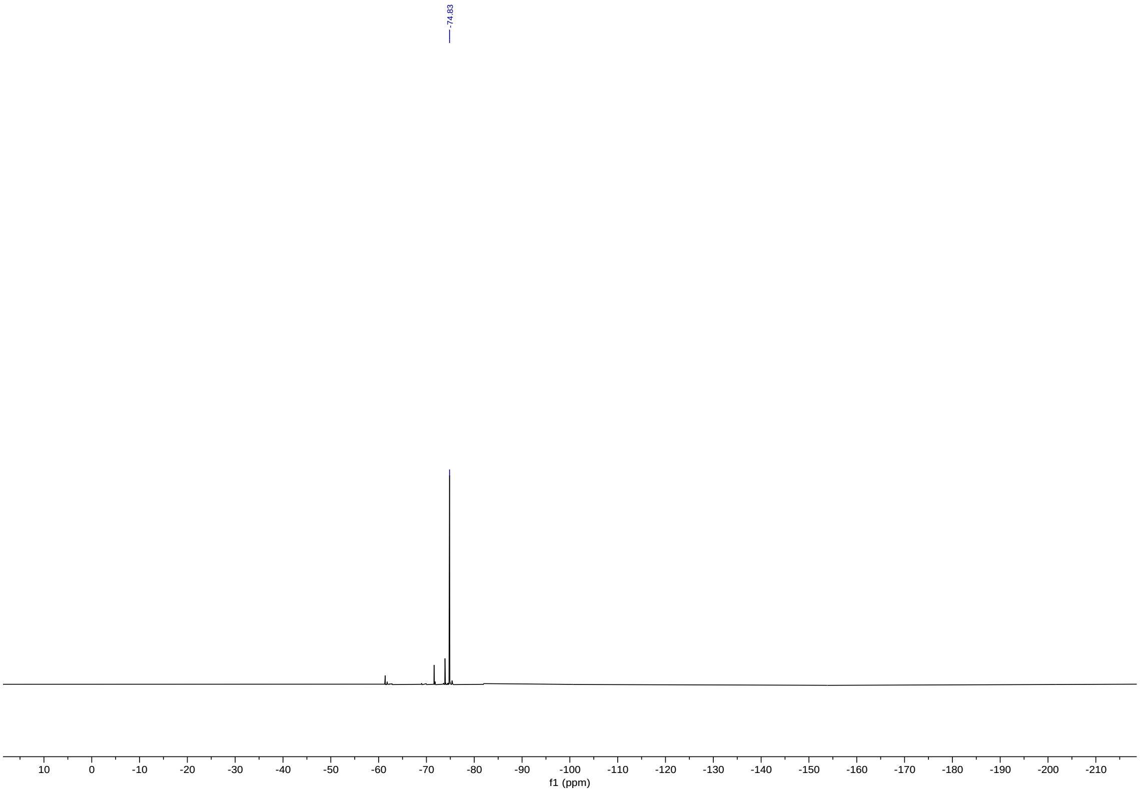

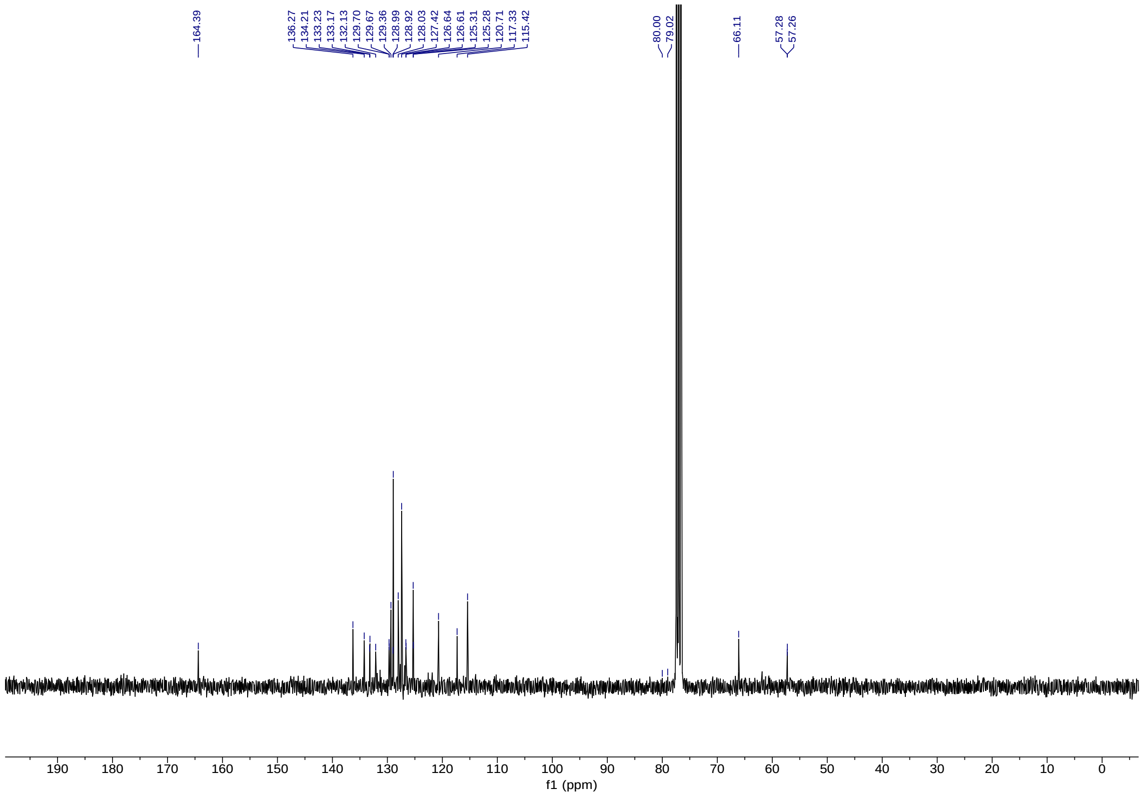
164.39
136.27
134.21
133.23
133.17
132.13
129.70
129.67
129.36
128.99
128.92
128.03
127.42
126.64
126.61
125.31
125.28
120.71
117.33
115.42
80.00
79.02
66.11
57.28
57.26
190 180 170 160 150 140 130 120 110 100 90 80 70 60 50 40 30 20 10 0
f1 (ppm)

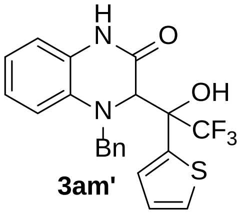

3am'

^{1}H-NMR (CDCl$_3$, 300 MHz)
^{19}F{^{1}H}-NMR (CDCl$_3$, 282 MHz)
^{13}C{^{1}H}-NMR (CDCl$_3$, 75 MHz)

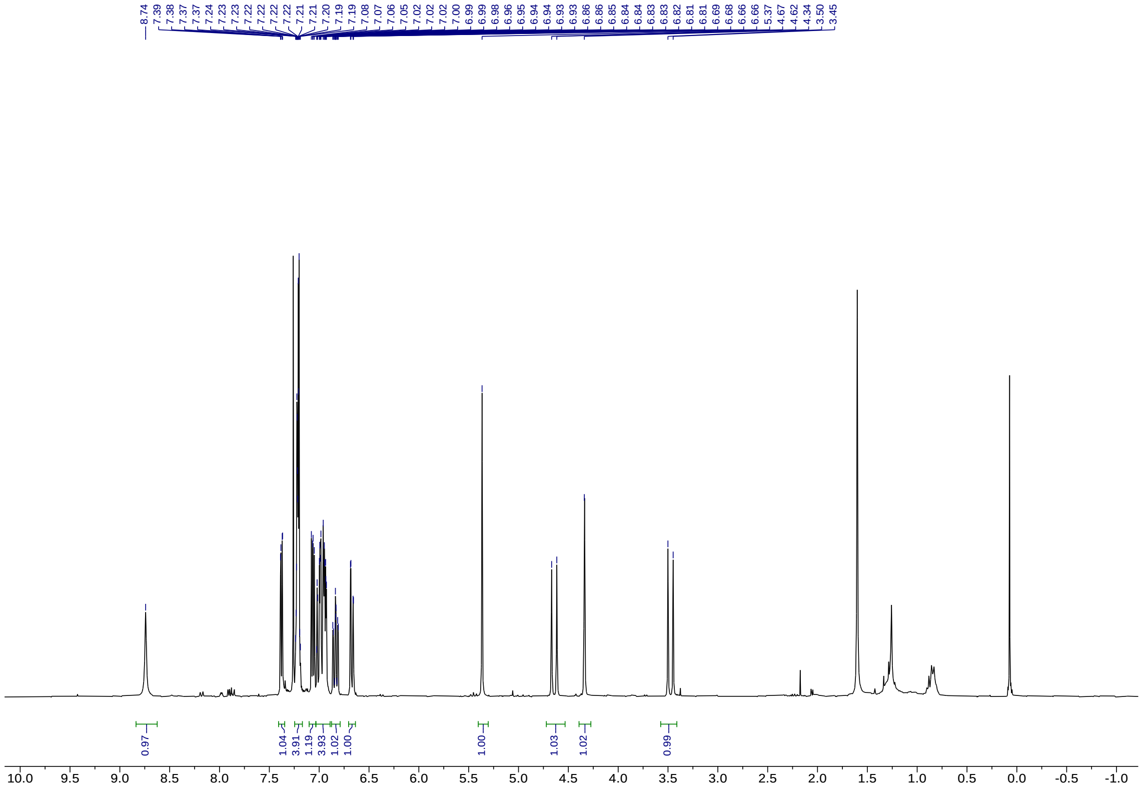

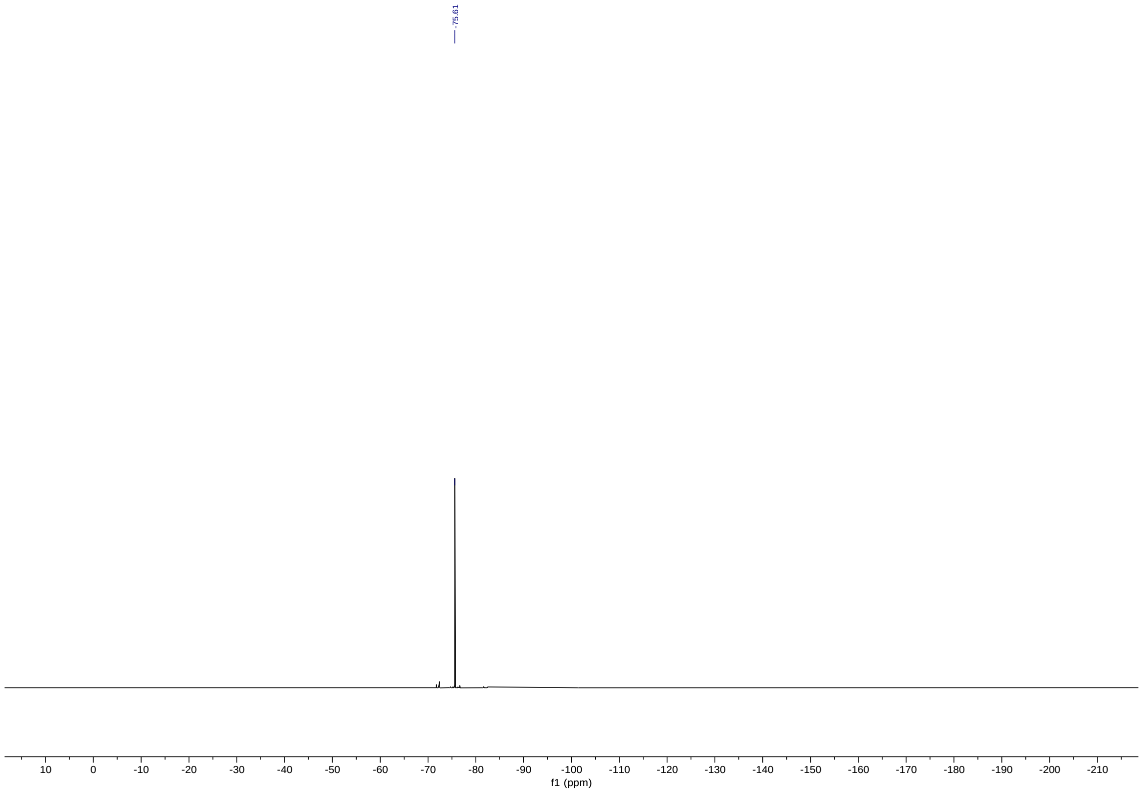

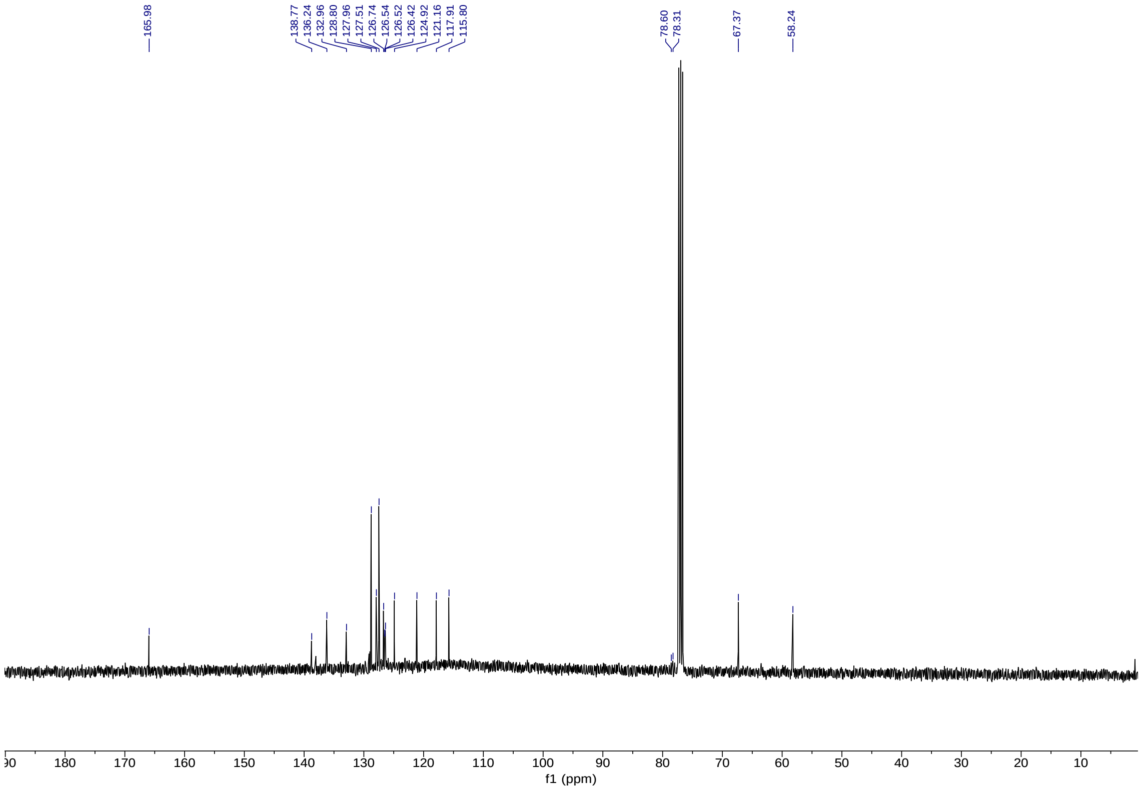
165.98
138.77
136.24
132.96
128.80
127.96
127.51
126.74
126.54
126.52
126.42
124.92
121.16
117.91
115.80
78.60
78.31
67.37
58.24
180
170
160
150
140
130
120
110
100
90
80
70
60
50
40
30
20
10
f1 (ppm)

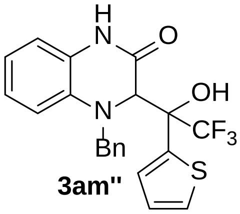

3am''

^{1}H-NMR ($CDCl_3$, 300 MHz)
^{19}F{^{1}H}-NMR ($CDCl_3$, 282 MHz)
^{13}C{^{1}H}-NMR ($CDCl_3$, 75 MHz)

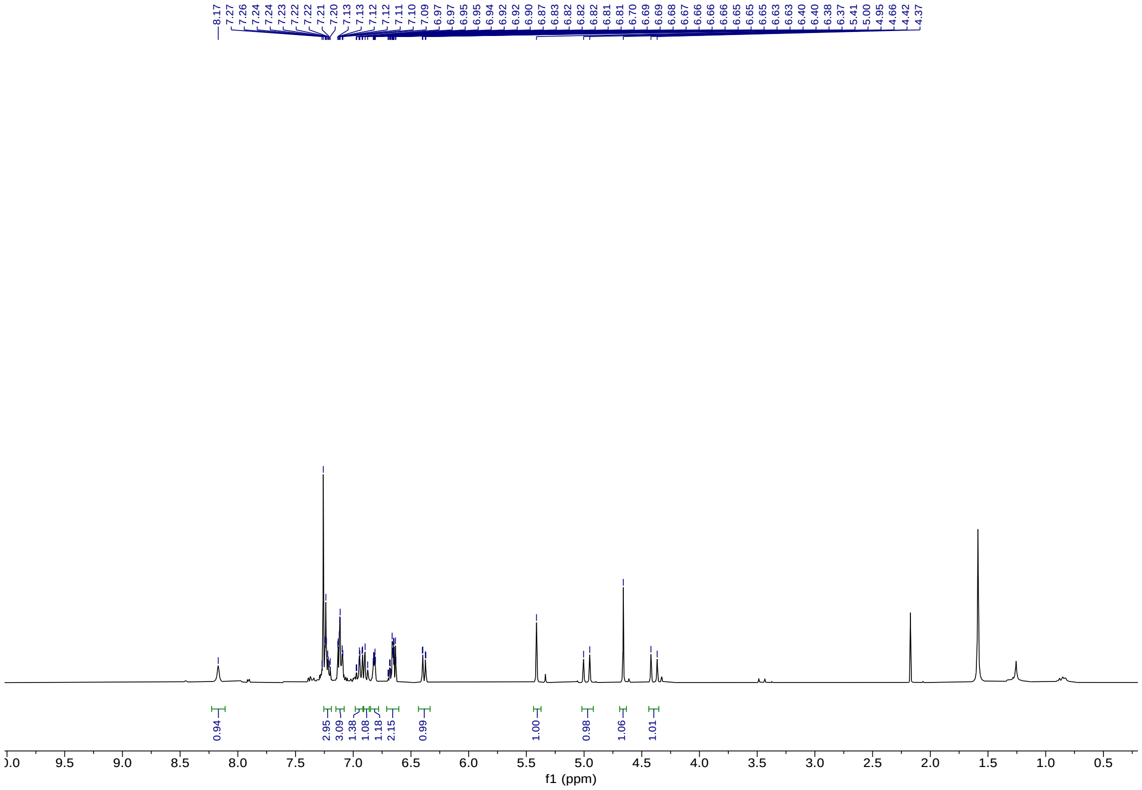

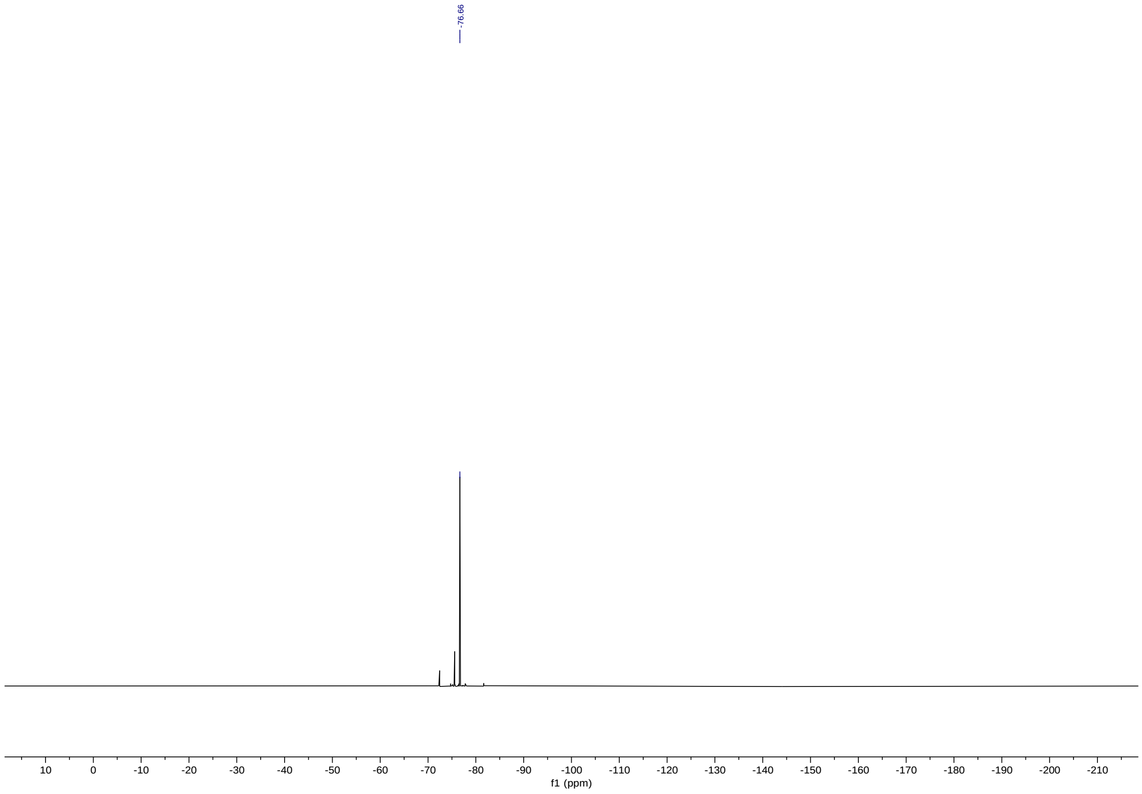

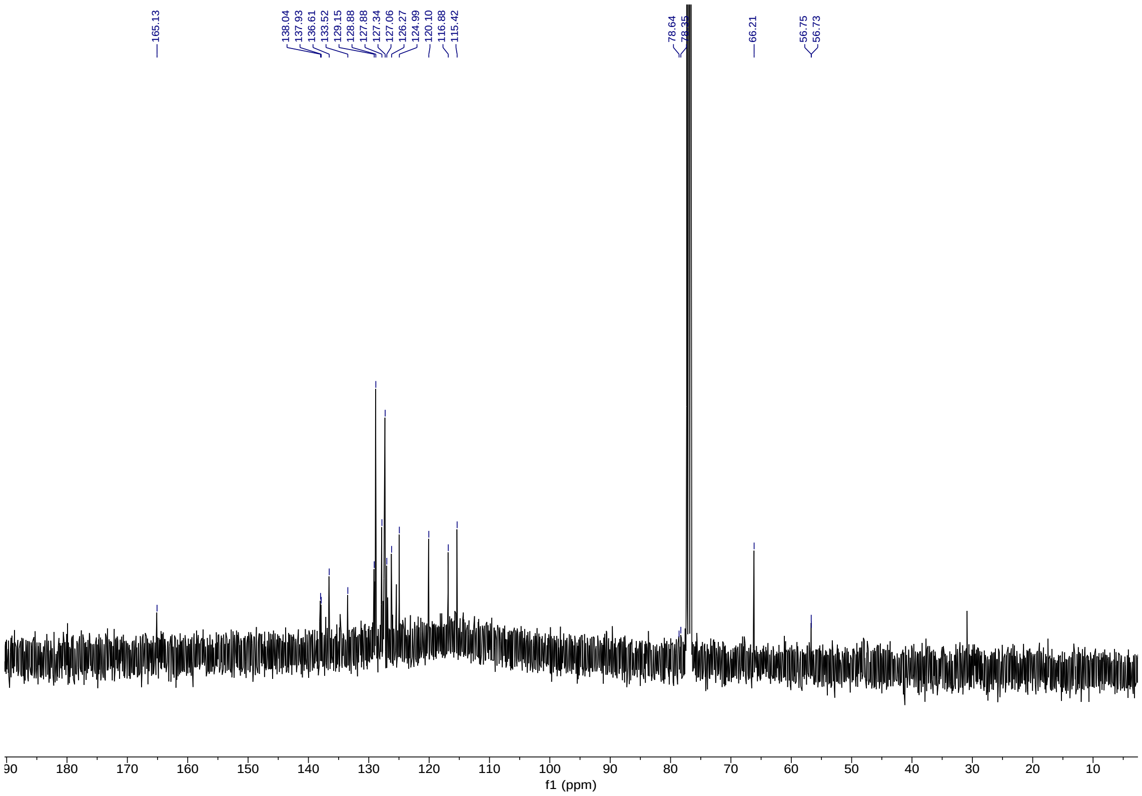
165.13
138.04
137.93
136.61
133.52
129.15
128.88
127.88
127.34
127.06
126.27
124.99
120.10
116.88
115.42
78.64
78.35
66.21
56.75
56.73
90
180
170
160
150
140
130
120
110
100
90
80
70
60
50
40
30
20
10
f1 (ppm)

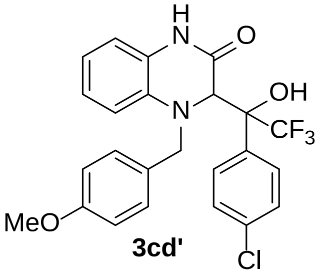

3cd'

^{1}H-NMR ($CDCl_3$, 300 MHz)
^{19}F{^{1}H}-NMR ($CDCl_3$, 282 MHz)
^{13}C{^{1}H}-NMR ($CDCl_3$, 75 MHz)

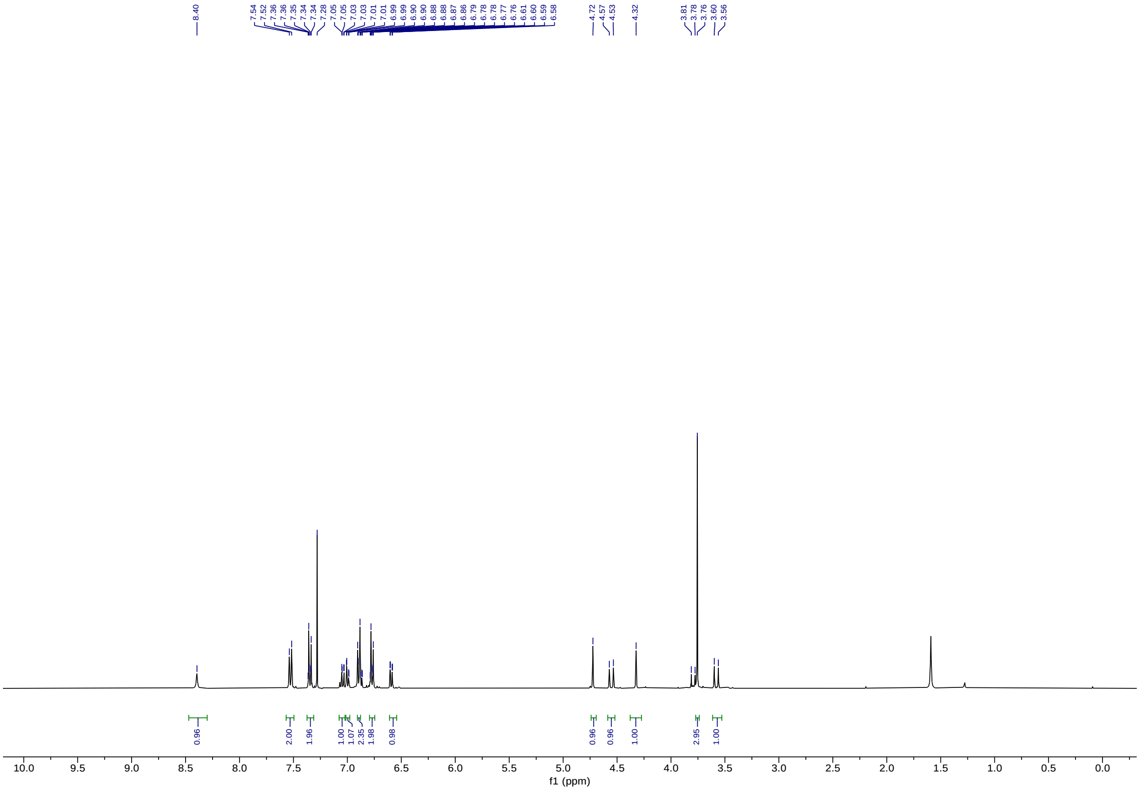

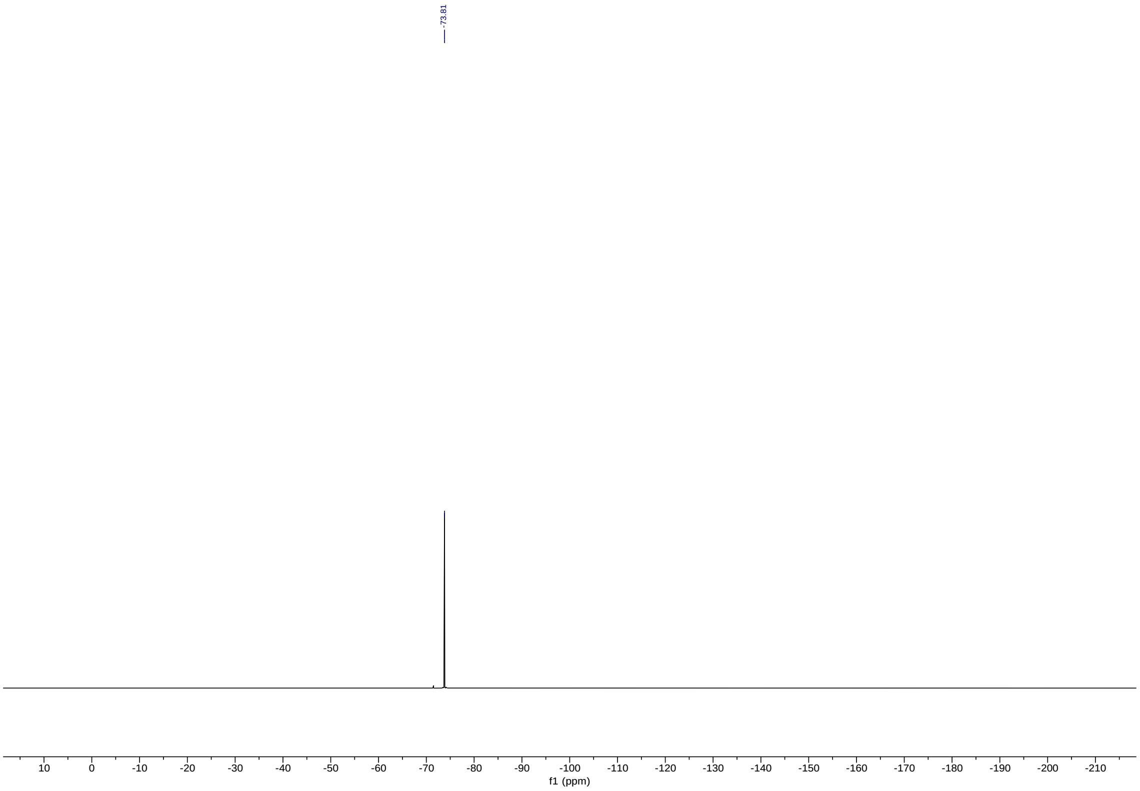

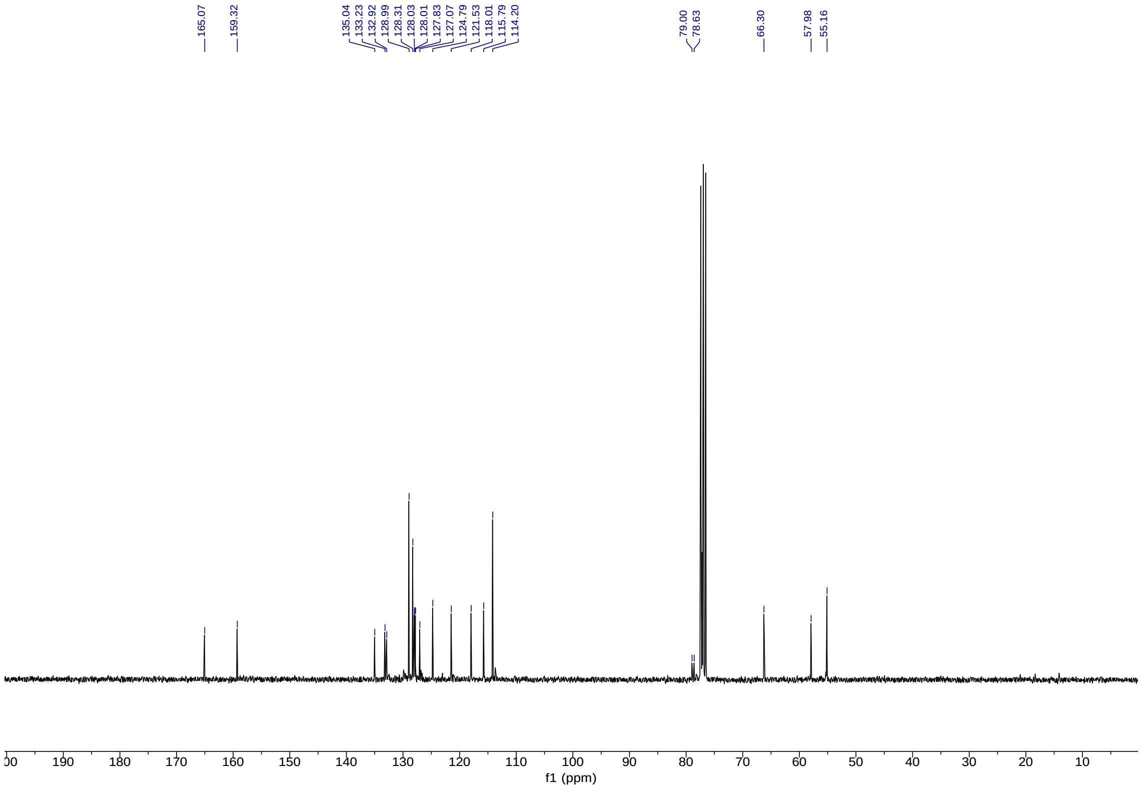
165.07
159.32
135.04
133.23
132.92
128.99
128.31
128.03
128.01
127.83
127.07
124.79
121.53
118.01
115.79
114.20
79.00
78.63
66.30
57.98
55.16
f1 (ppm)

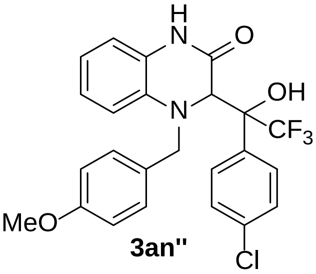

3an''

^{1}H-NMR ($CDCl_3$, 300 MHz)
^{19}F{^{1}H}-NMR ($CDCl_3$, 282 MHz)
^{13}C{^{1}H}-NMR ($CDCl_3$, 75 MHz)

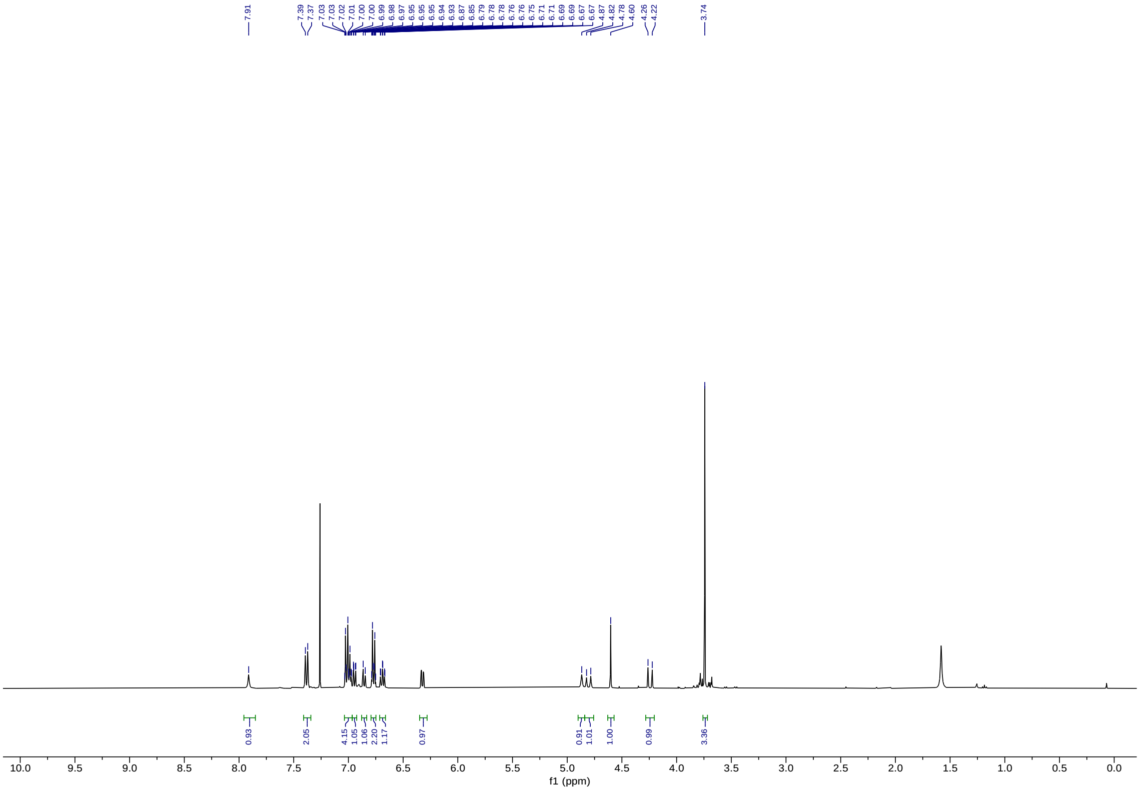

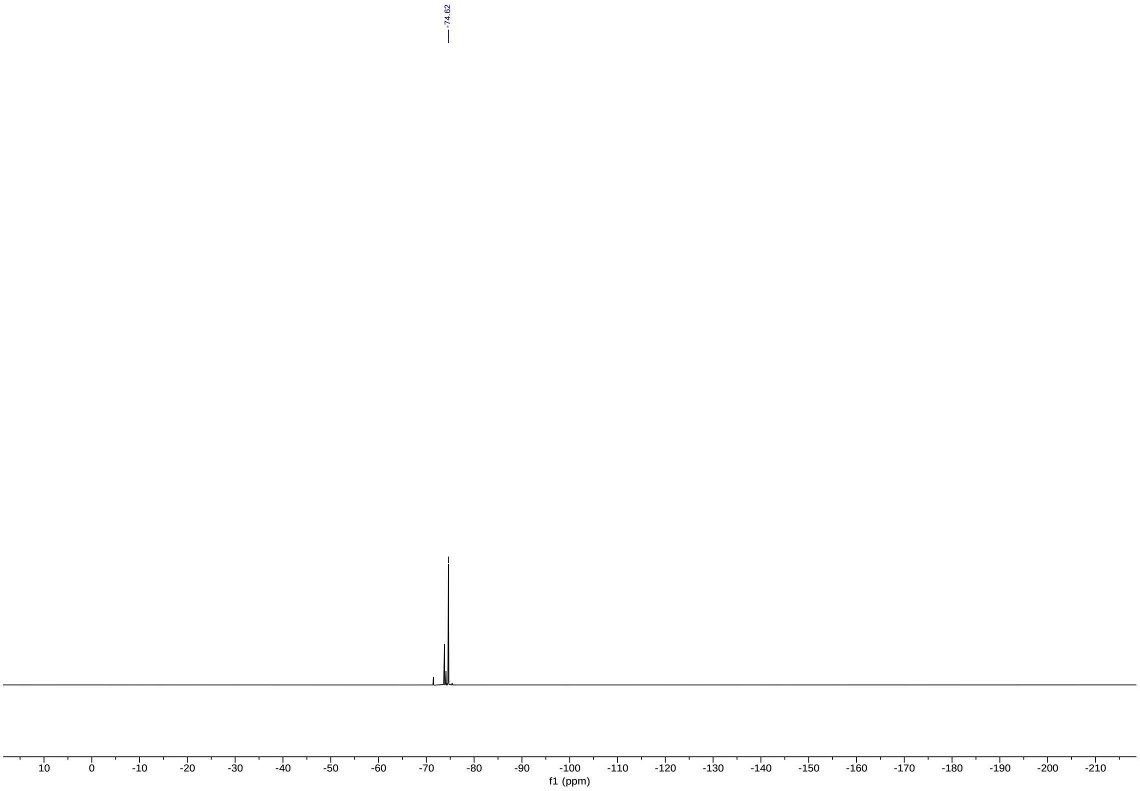

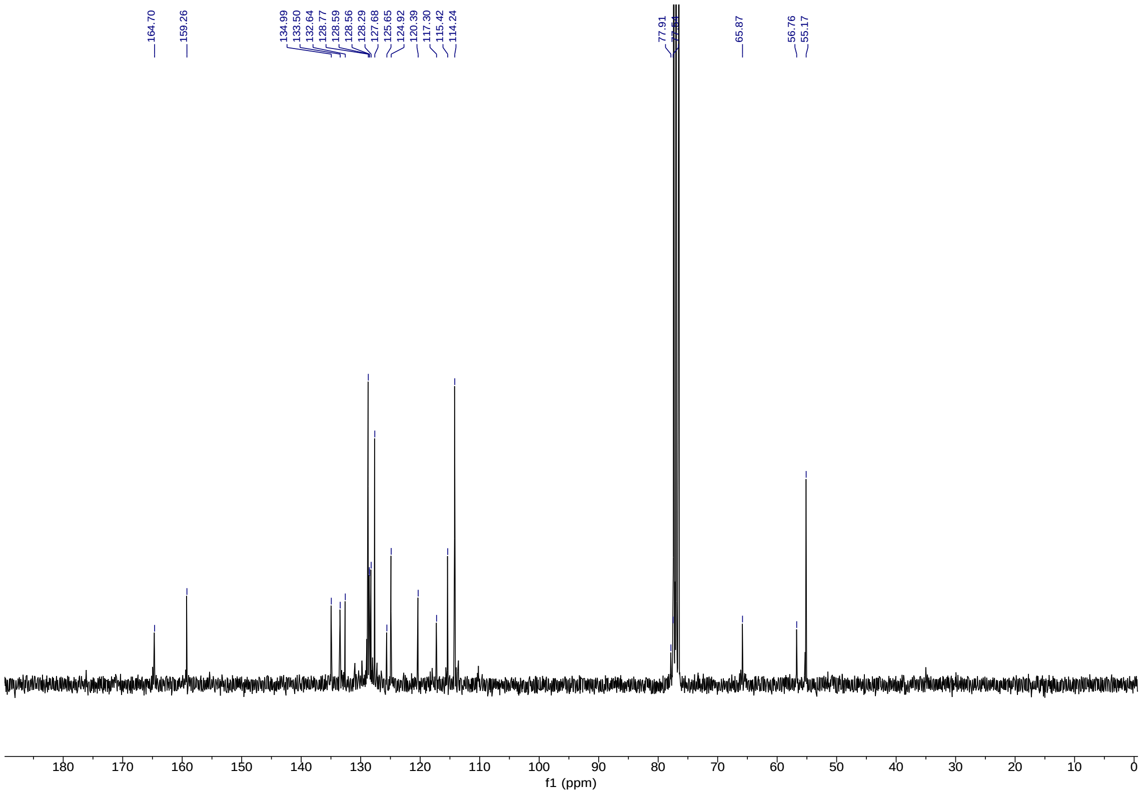

164.70
159.26
134.99
133.50
132.64
128.77
128.59
128.56
128.29
127.68
125.65
124.92
120.39
117.30
115.42
114.24
77.91
65.87
56.76
55.17
180
170
160
150
140
130
120
110
100
90
80
70
60
50
40
30
20
10
0
f1 (ppm)

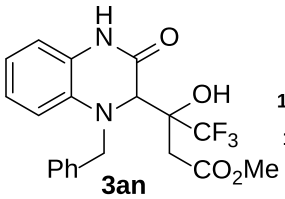

3an

^{1}H-NMR ($CDCl_3$, 300 MHz)
^{19}F{^{1}H}-NMR ($CDCl_3$, 282 MHz)
^{13}C{^{1}H}-NMR ($CDCl_3$, 75 MHz)

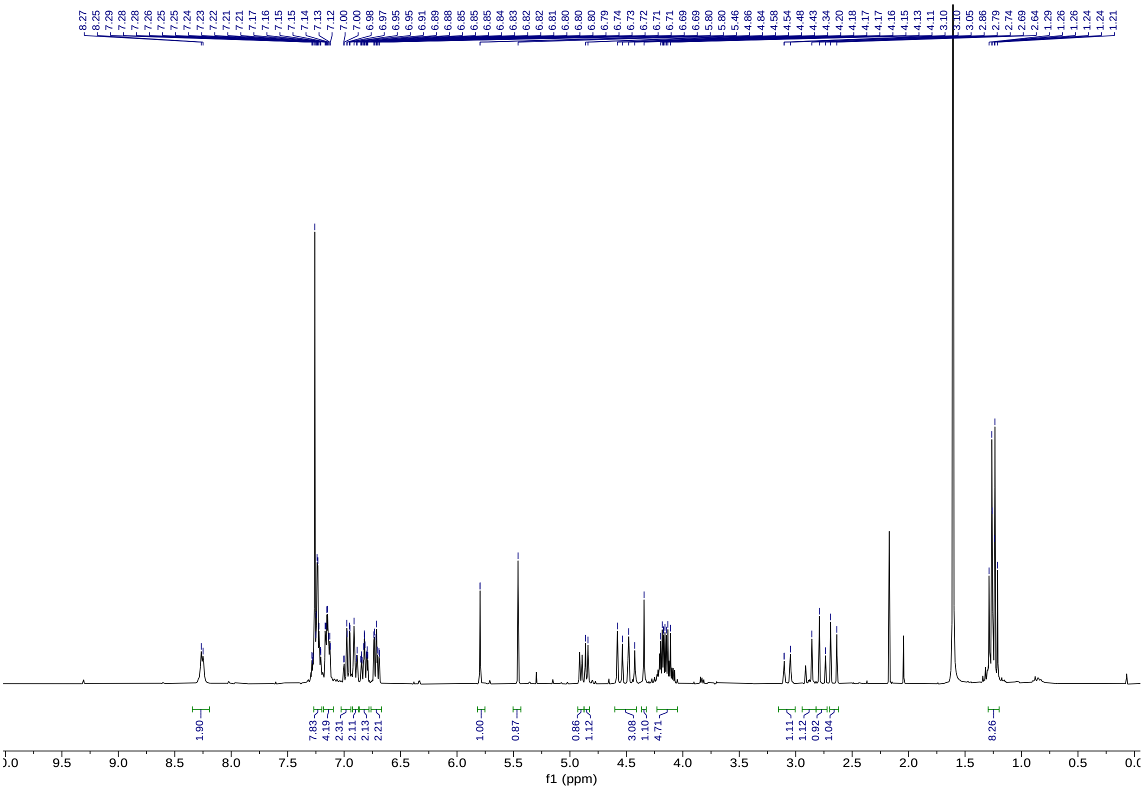

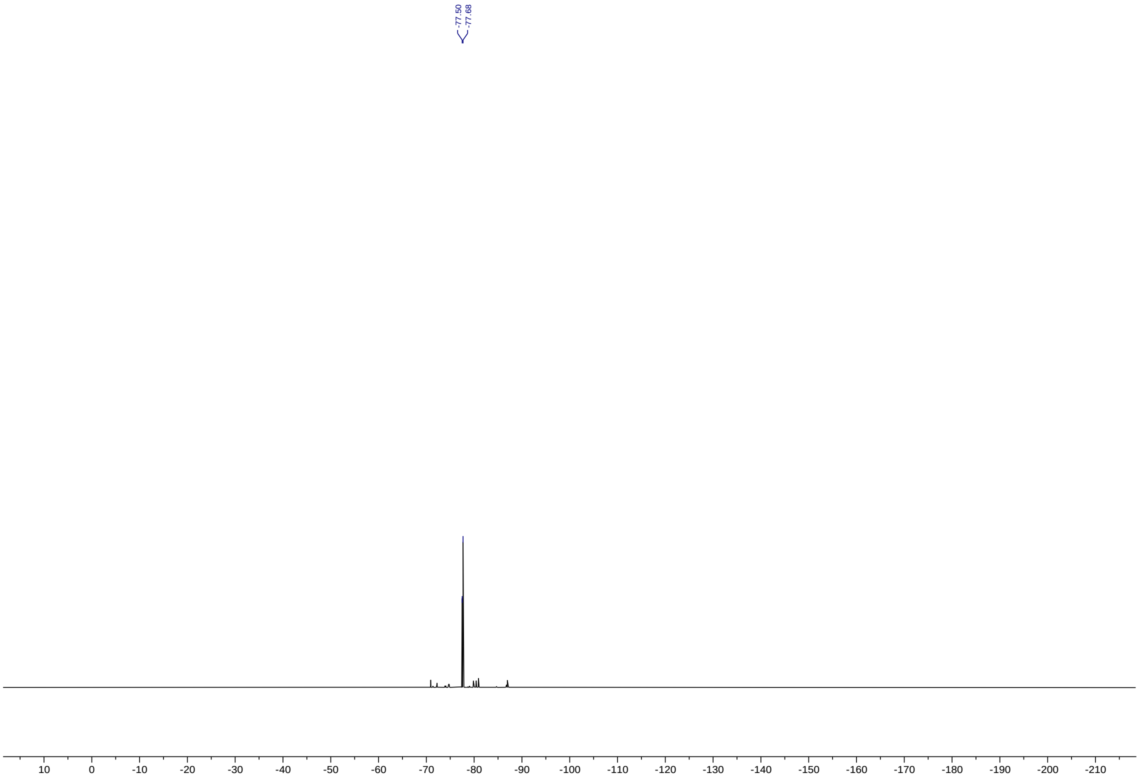

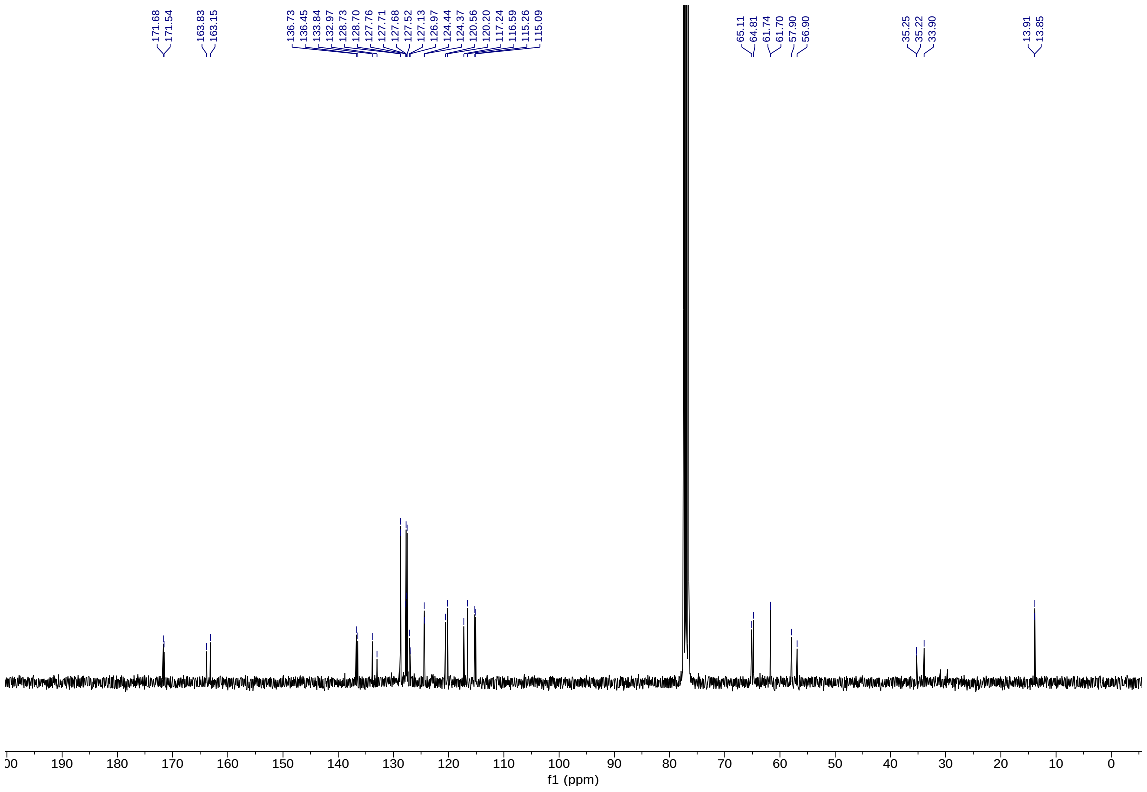
171.68
171.54
163.83
163.15
136.73
136.45
133.84
132.97
128.73
128.70
127.76
127.71
127.68
127.52
127.13
126.97
124.44
124.37
120.56
120.20
117.24
116.59
115.26
115.09
65.11
64.81
61.74
61.70
57.90
56.90
35.25
35.22
33.90
13.91
13.85
f1 (ppm)

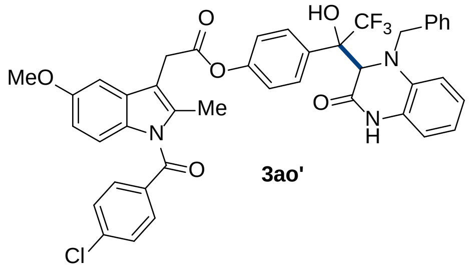

3ao'

^{1}H-NMR ($CDCl_3$, 300 MHz)
$^{19}F\{^{1}H\}$-NMR ($CDCl_3$, 282 MHz)
$^{13}C\{^{1}H\}$-NMR ($CDCl_3$, 75 MHz)

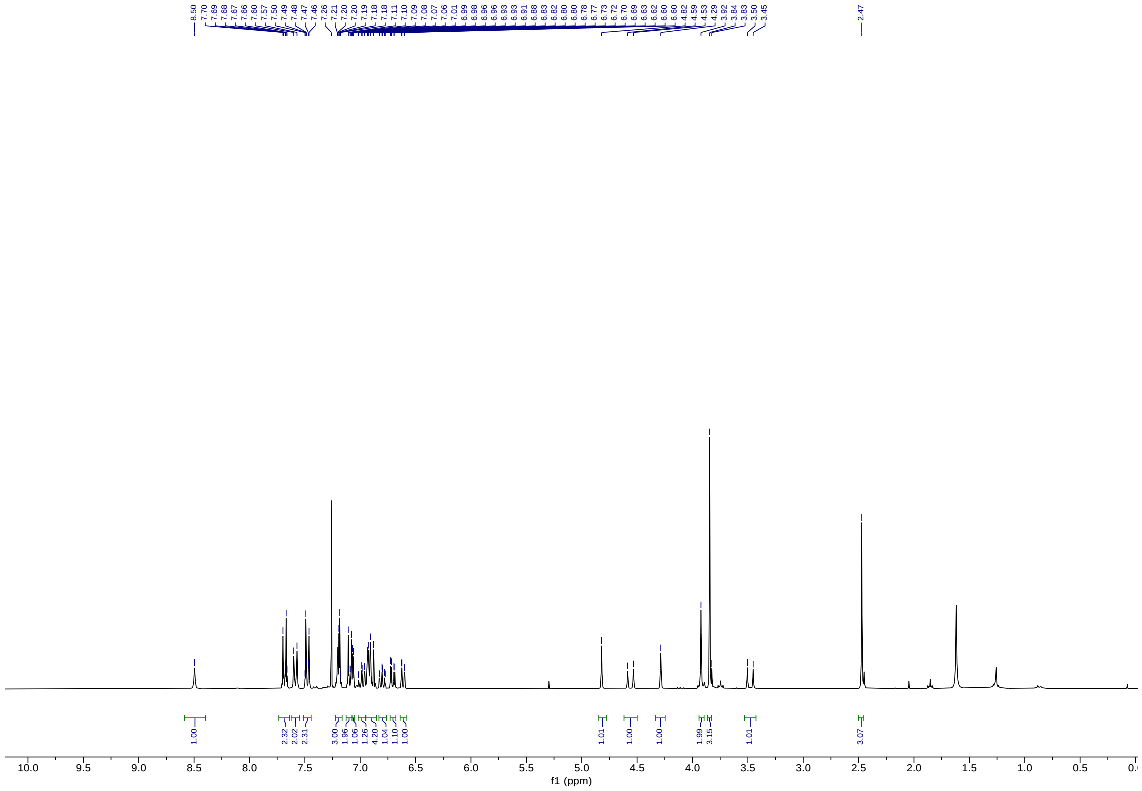

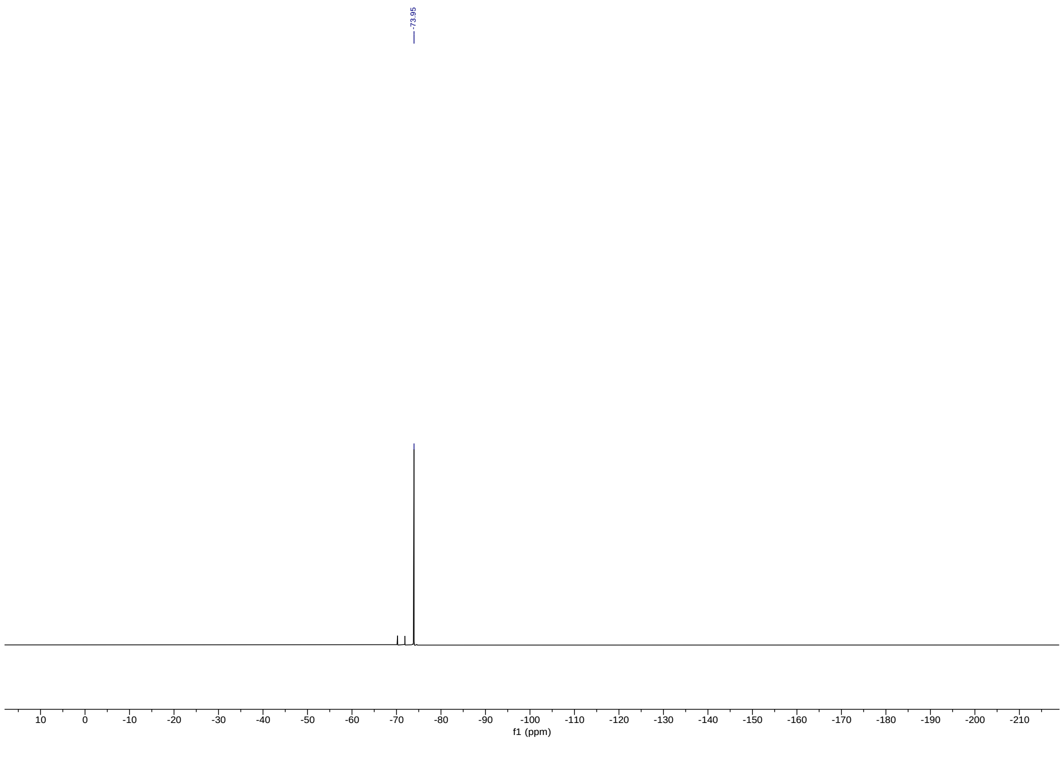

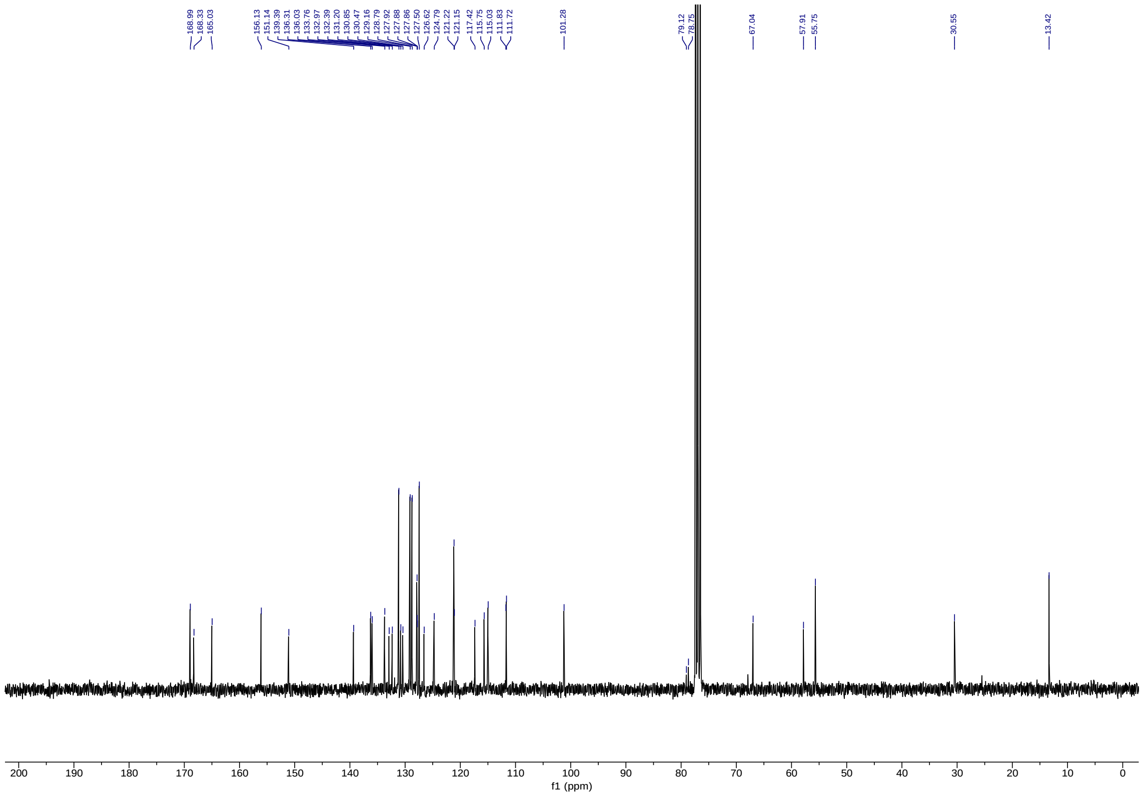

168.99
168.33
165.03
156.13
151.14
139.39
136.31
136.03
133.76
132.97
132.39
131.20
130.85
130.47
129.16
128.79
127.92
127.88
127.86
127.50
126.62
124.79
121.22
121.15
117.42
115.75
115.03
111.83
111.72
101.28
79.12
78.75
67.04
57.91
55.75
30.55
13.42
f1 (ppm)

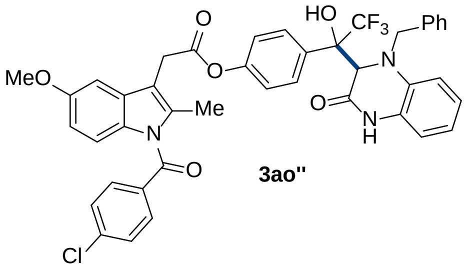

3ao''

^{1}H-NMR ($CDCl_3$, 300 MHz)
^{19}F{^{1}H}-NMR ($CDCl_3$, 282 MHz)
^{13}C{^{1}H}-NMR ($CDCl_3$, 75 MHz)

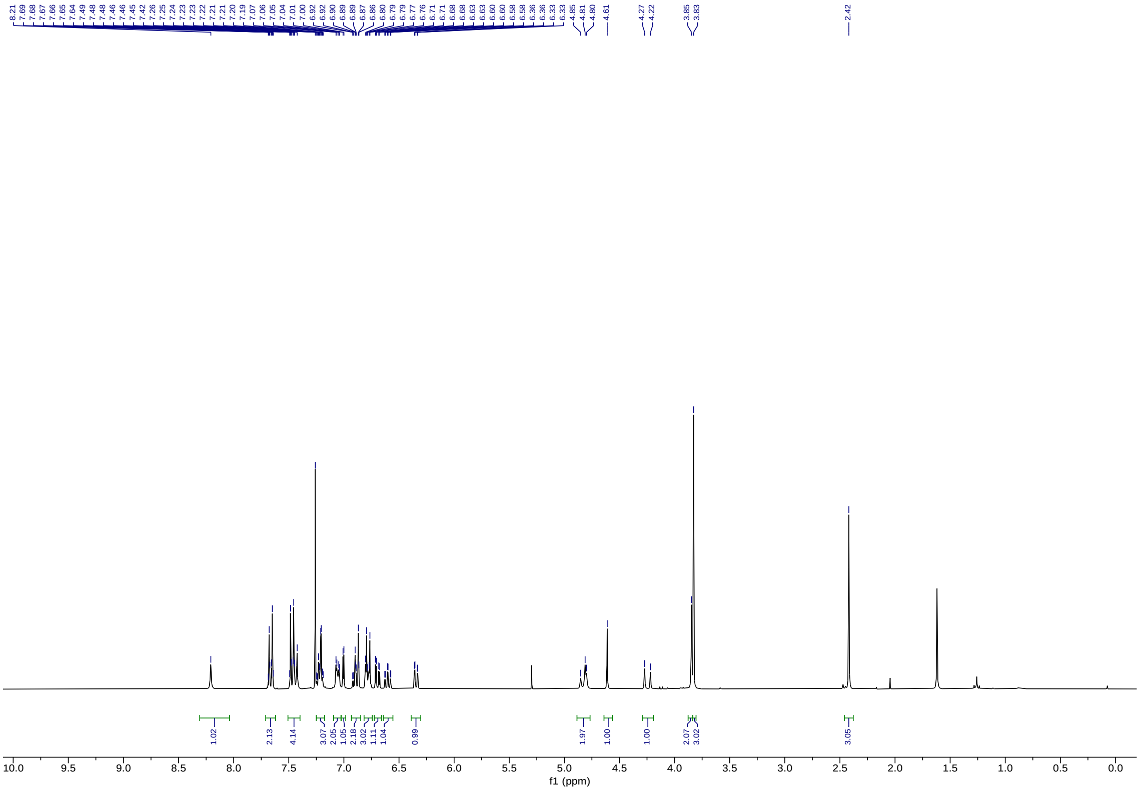

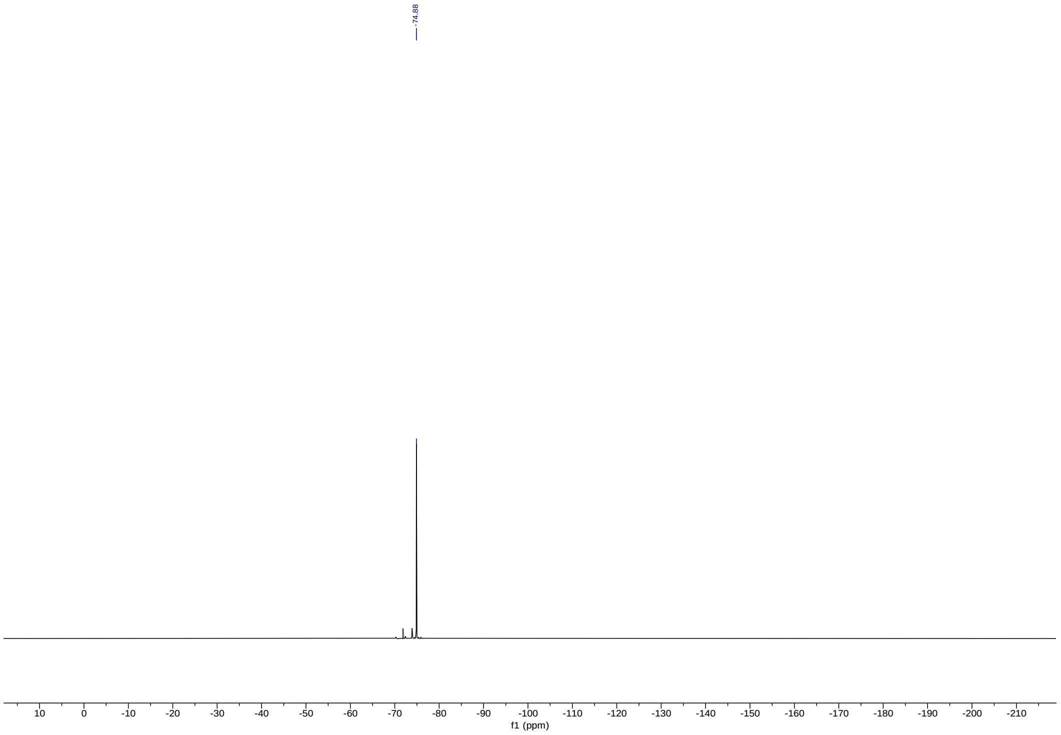

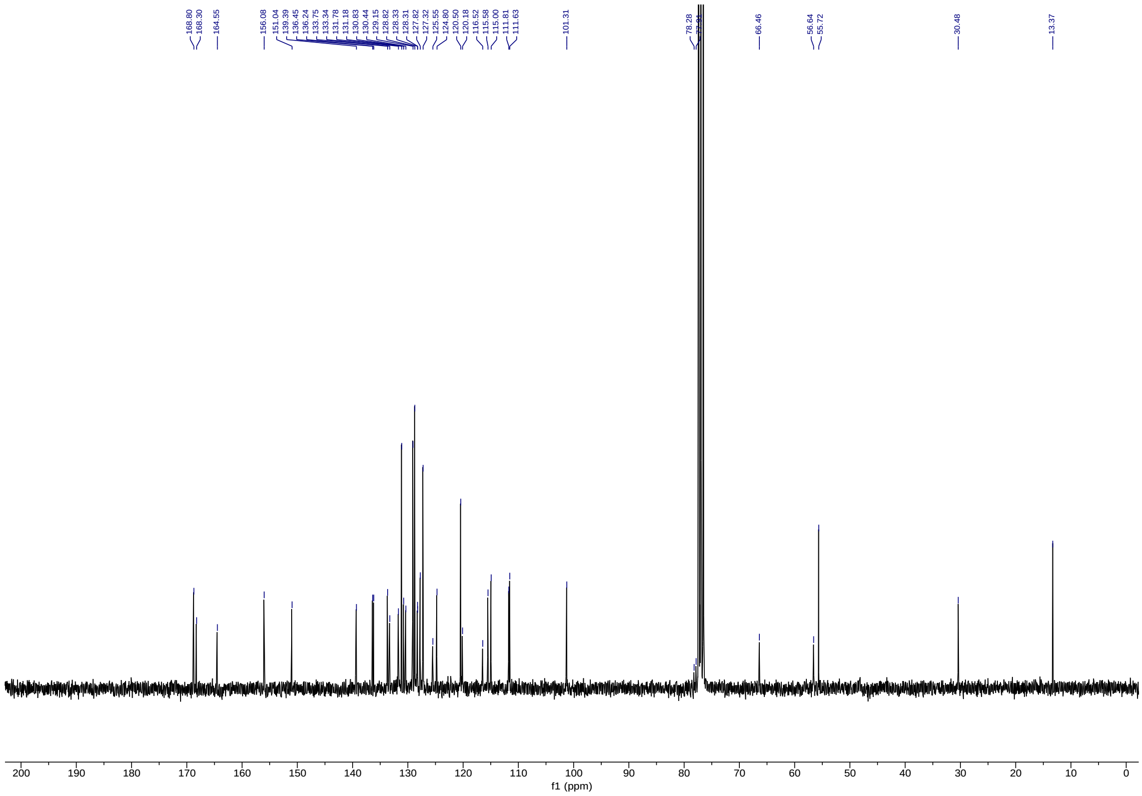
168.80
168.30
164.55
156.08
151.04
139.39
136.45
136.24
133.75
133.34
131.78
131.18
130.83
130.44
129.15
128.82
128.33
128.31
127.82
127.32
125.55
124.80
120.50
120.18
116.52
115.58
115.00
111.81
111.63
101.31
78.28
66.46
56.64
55.72
30.48
13.37
200 190 180 170 160 150 140 130 120 110 100 90 80 70 60 50 40 30 20 10 0
f1 (ppm)

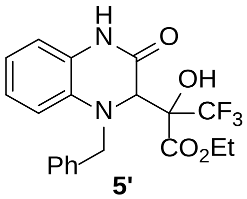

5'

^{1}H-NMR ($CDCl_3$, 300 MHz)
^{19}F{^{1}H}-NMR ($CDCl_3$, 282 MHz)
^{13}C{^{1}H}-NMR ($CDCl_3$, 75 MHz)

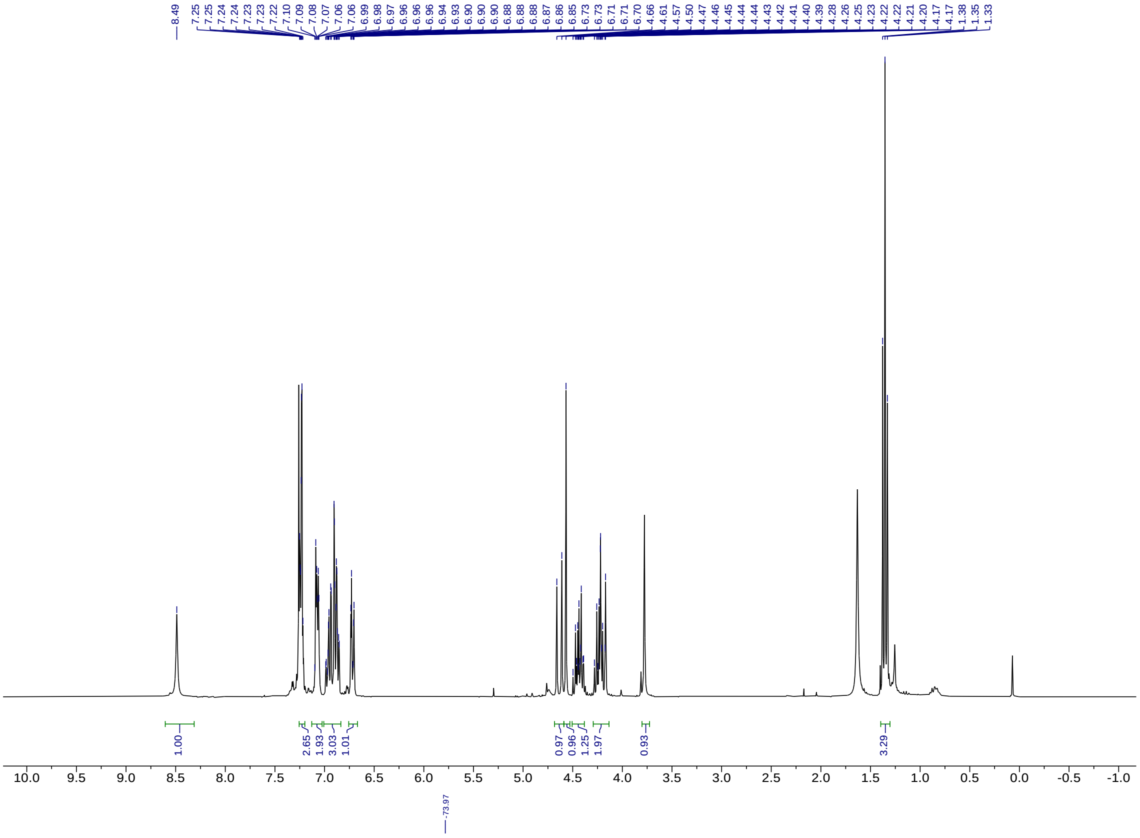

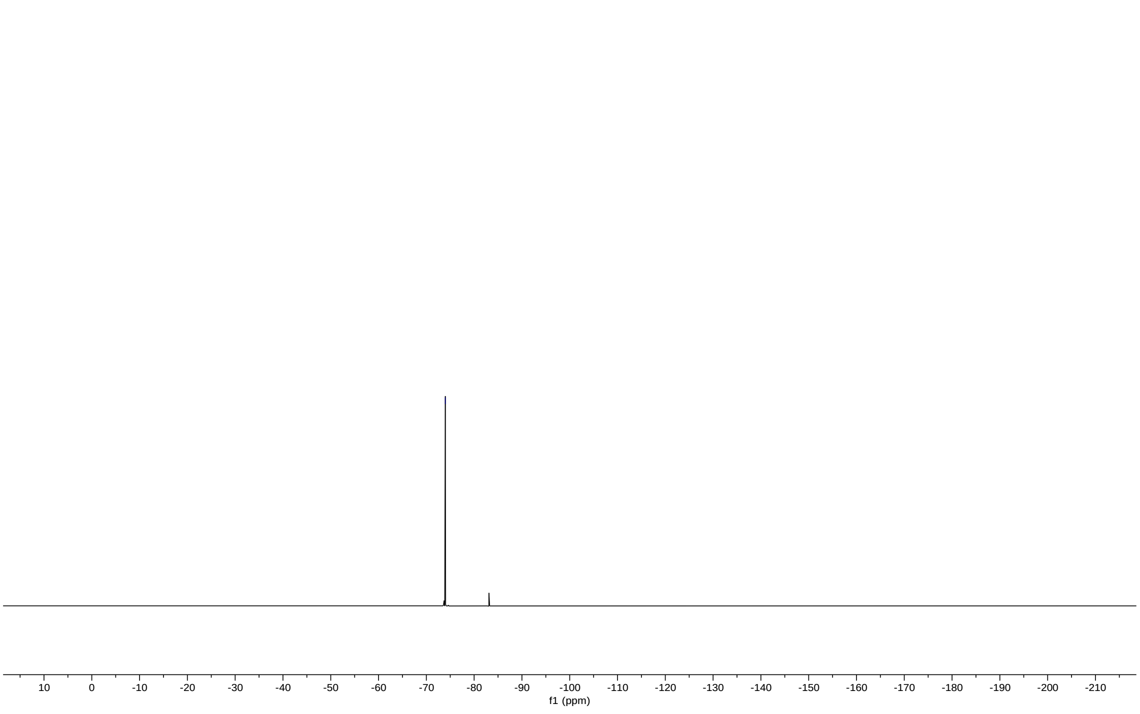

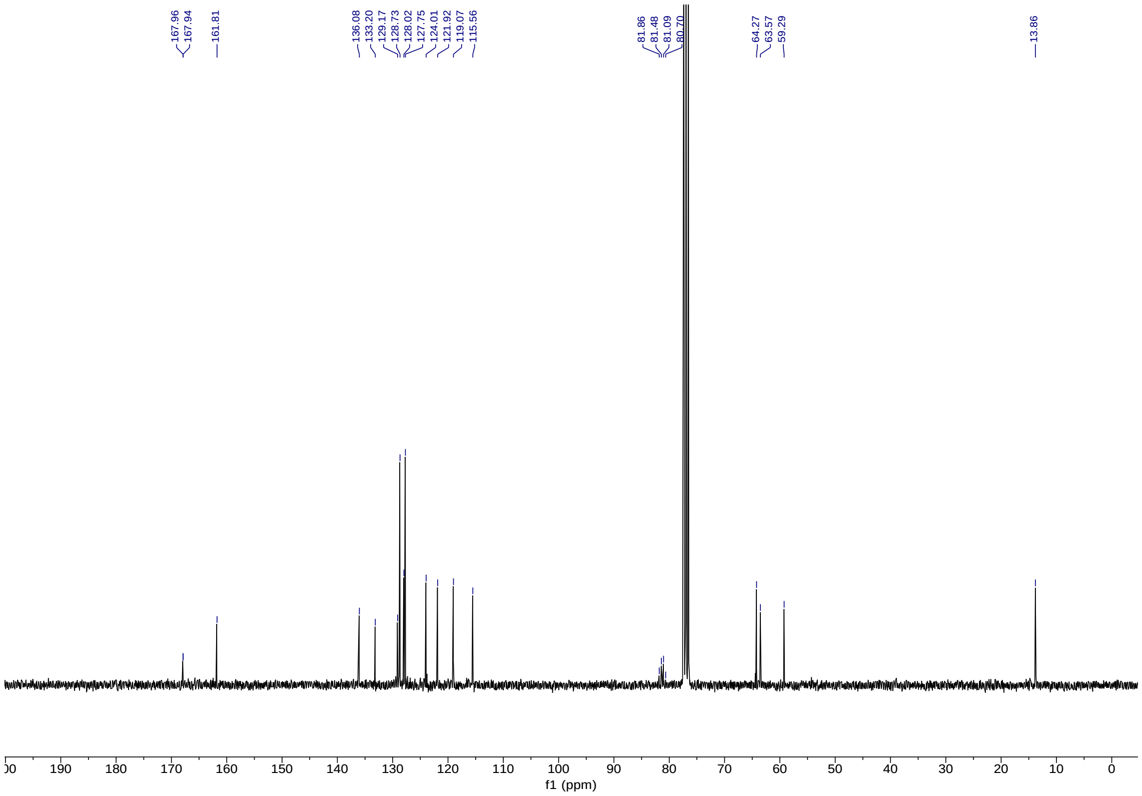
167.96
167.94
161.81
136.08
133.20
129.17
128.73
128.02
127.75
124.01
121.92
119.07
115.56
81.86
81.48
81.09
80.70
64.27
63.57
59.29
13.86
190 180 170 160 150 140 130 120 110 100 90 80 70 60 50 40 30 20 10 0
f1 (ppm)

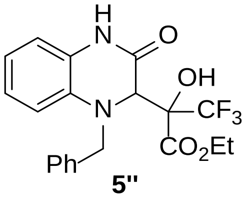

5''

^{1}H-NMR ($CDCl_3$, 300 MHz)
$^{19}F\{^{1}H\}$-NMR ($CDCl_3$, 282 MHz)
$^{13}C\{^{1}H\}$-NMR ($CDCl_3$, 75 MHz)

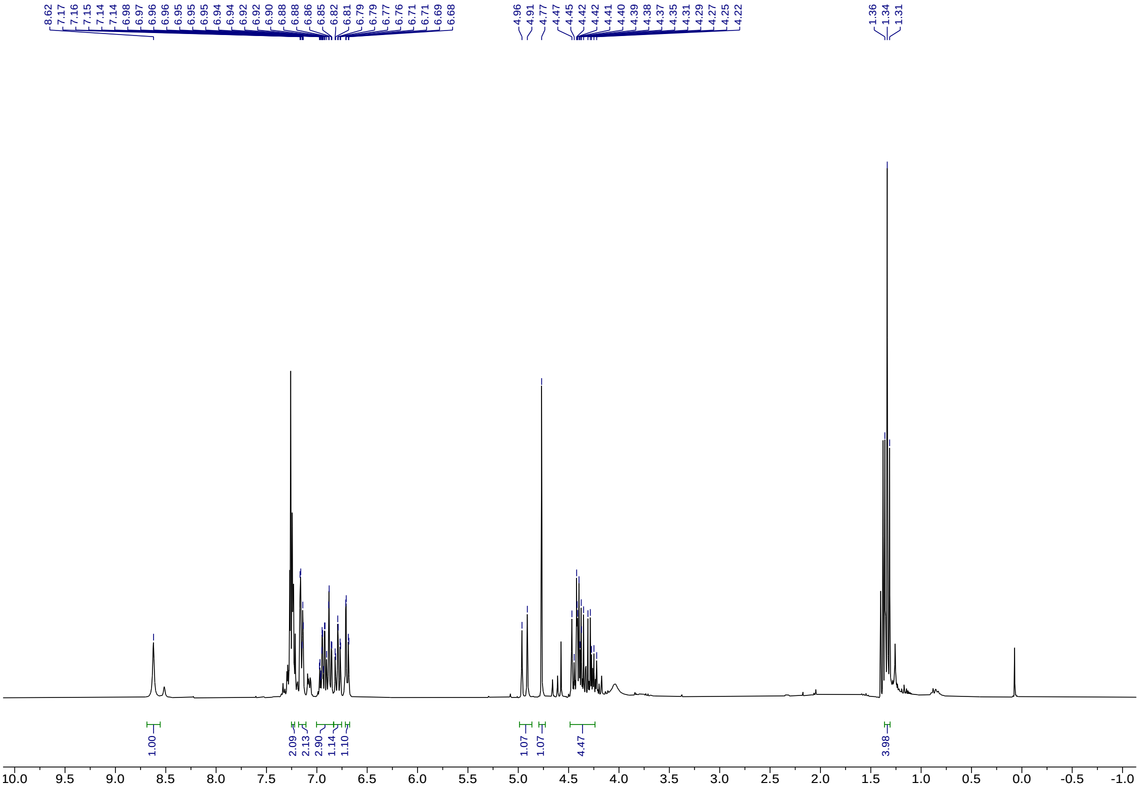

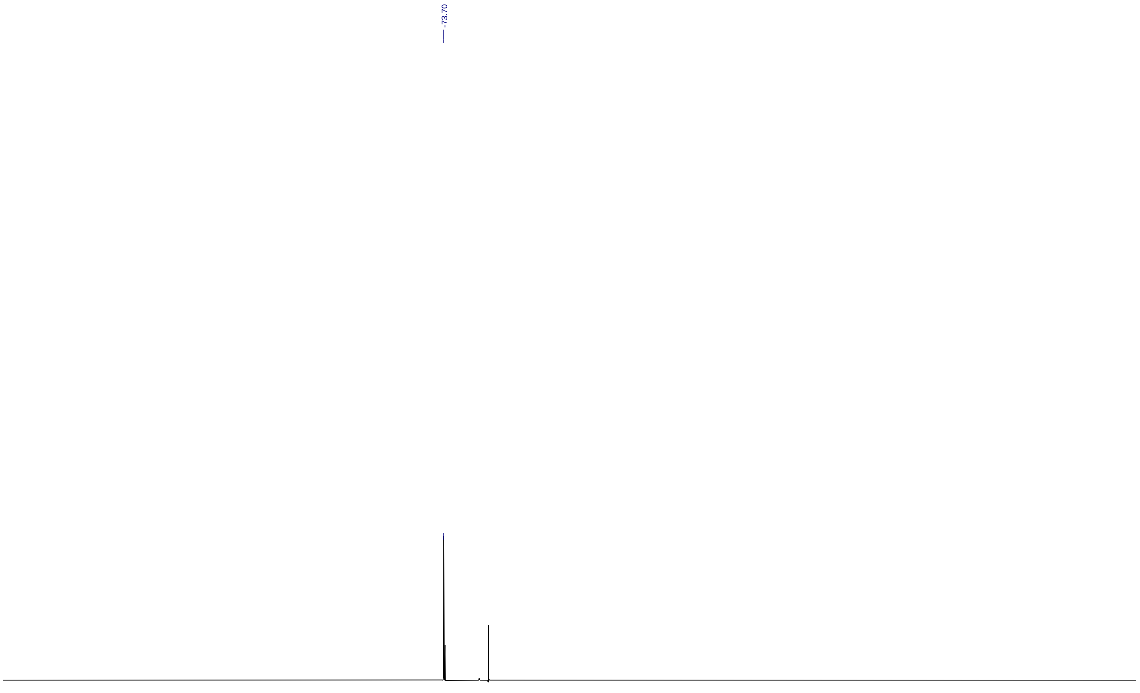

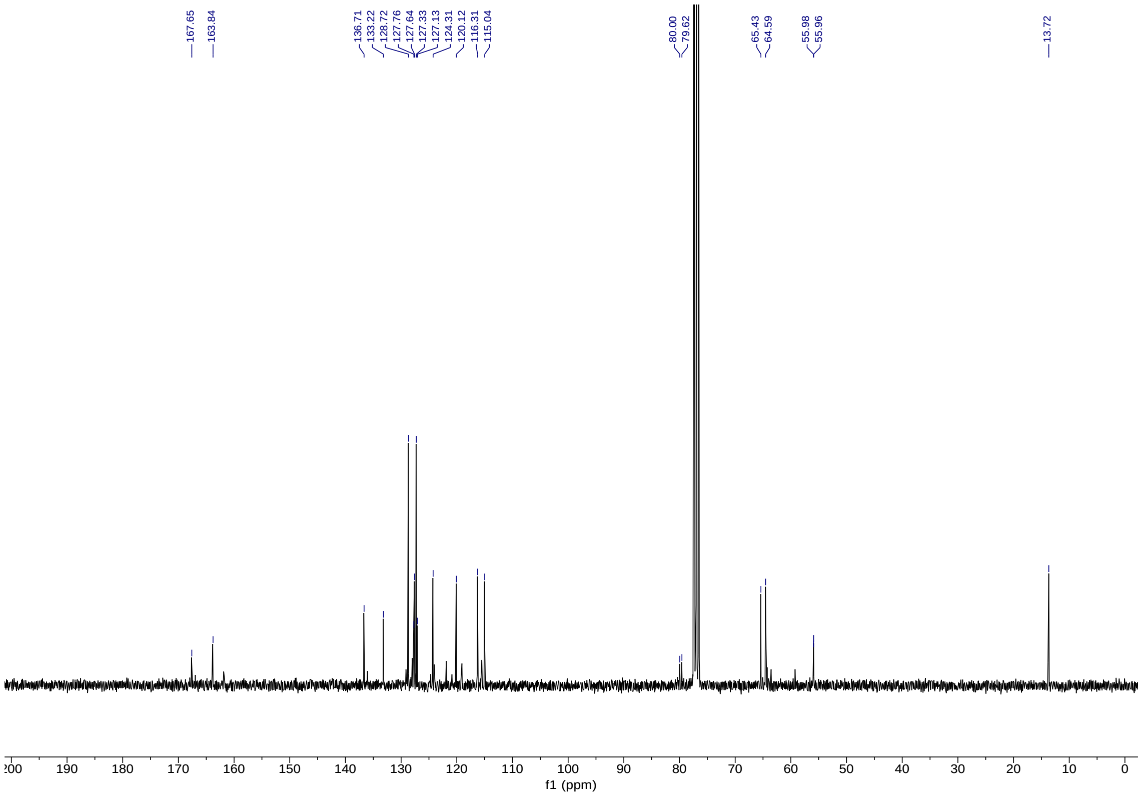
167.65
163.84
136.71
133.22
128.72
127.76
127.64
127.33
127.13
124.31
120.12
116.31
115.04
80.00
79.62
65.43
64.59
55.98
55.96
13.72
f1 (ppm)

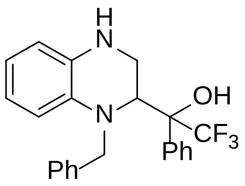

6'

^{1}H-NMR ($CDCl_3$, 300 MHz)
^{19}F{^{1}H}-NMR ($CDCl_3$, 282 MHz)
^{13}C{^{1}H}-NMR ($CDCl_3$, 75 MHz)

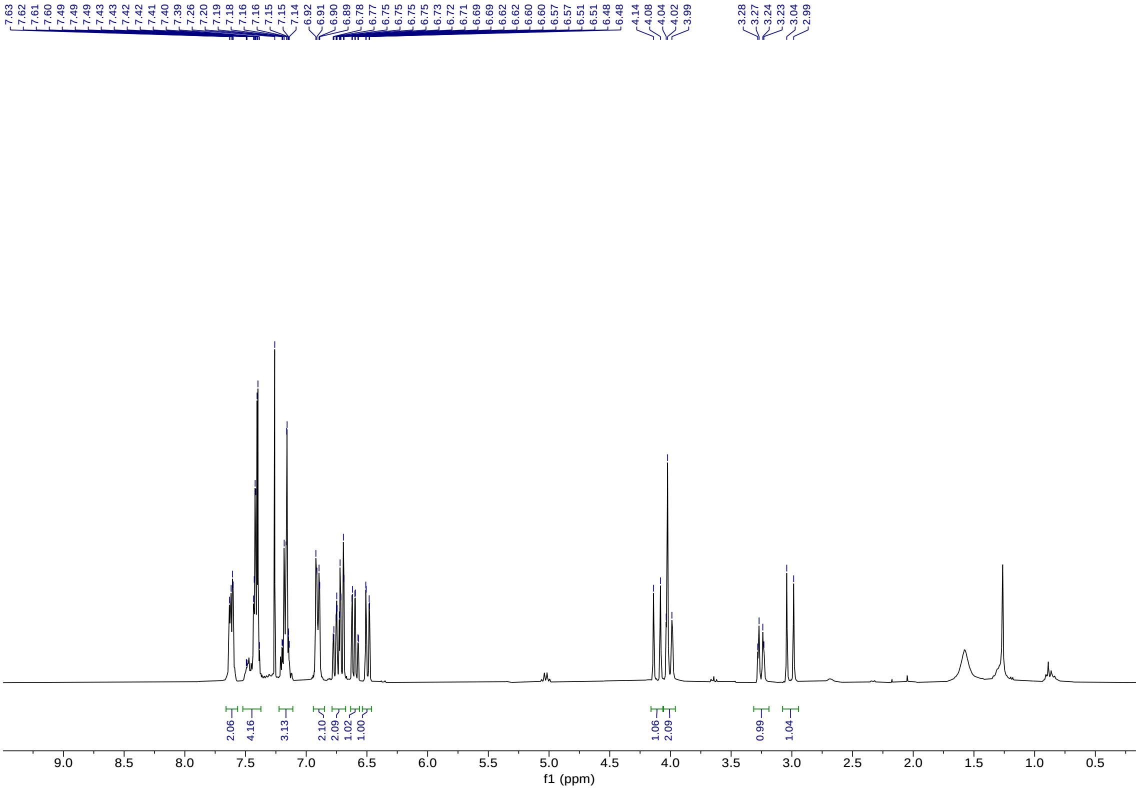

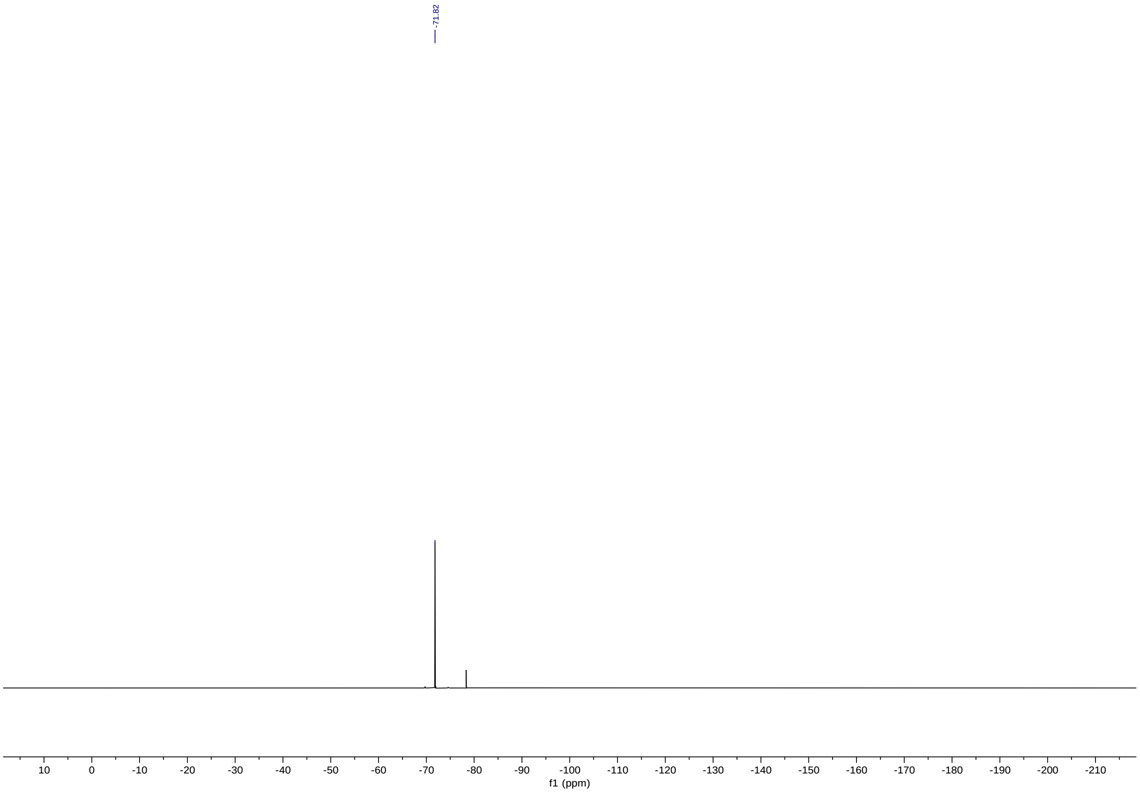

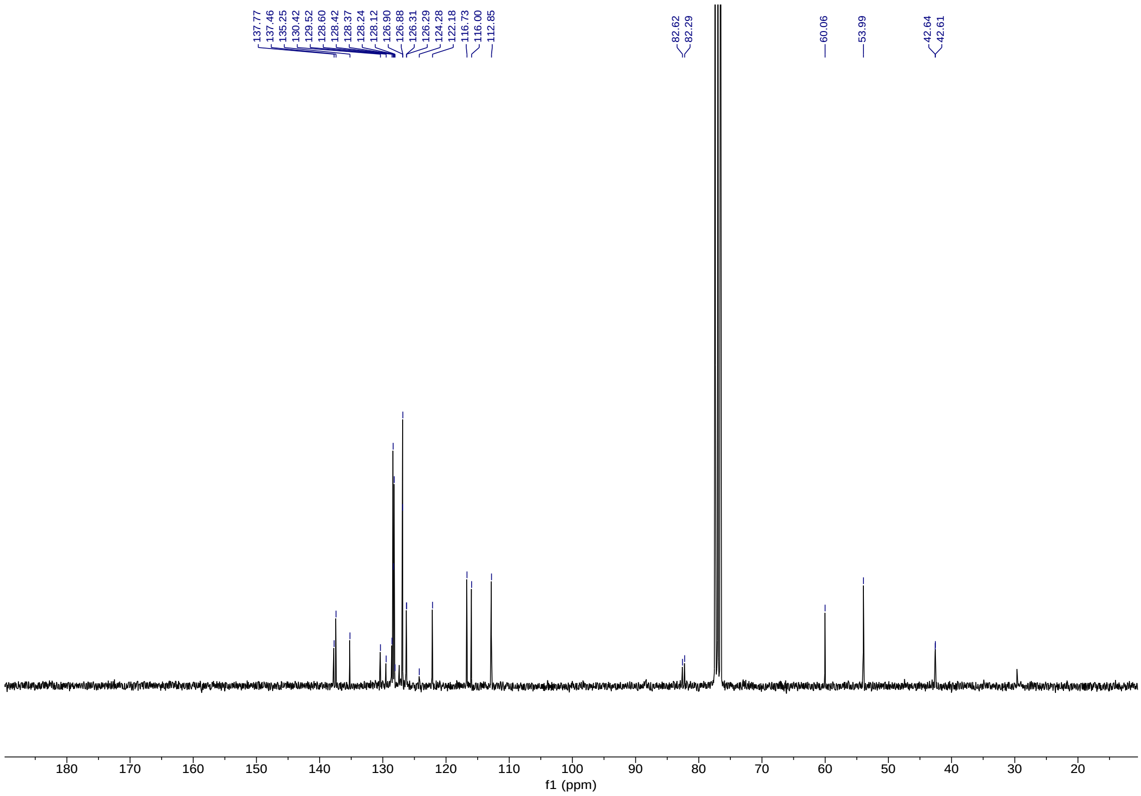
137.77
137.46
135.25
130.42
129.52
128.60
128.42
128.37
128.24
128.12
126.90
126.88
126.31
126.29
124.28
122.18
116.73
116.00
112.85
82.62
82.29
60.06
53.99
42.64
42.61
180 170 160 150 140 130 120 110 100 90 80 70 60 50 40 30 20
f1 (ppm)

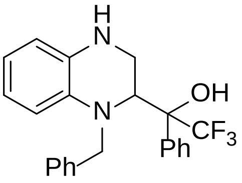

6''

^{1}H-NMR ($CDCl_3$, 300 MHz)
^{19}F{^{1}H}-NMR ($CDCl_3$, 282 MHz)
^{13}C{^{1}H}-NMR ($CDCl_3$, 75 MHz)

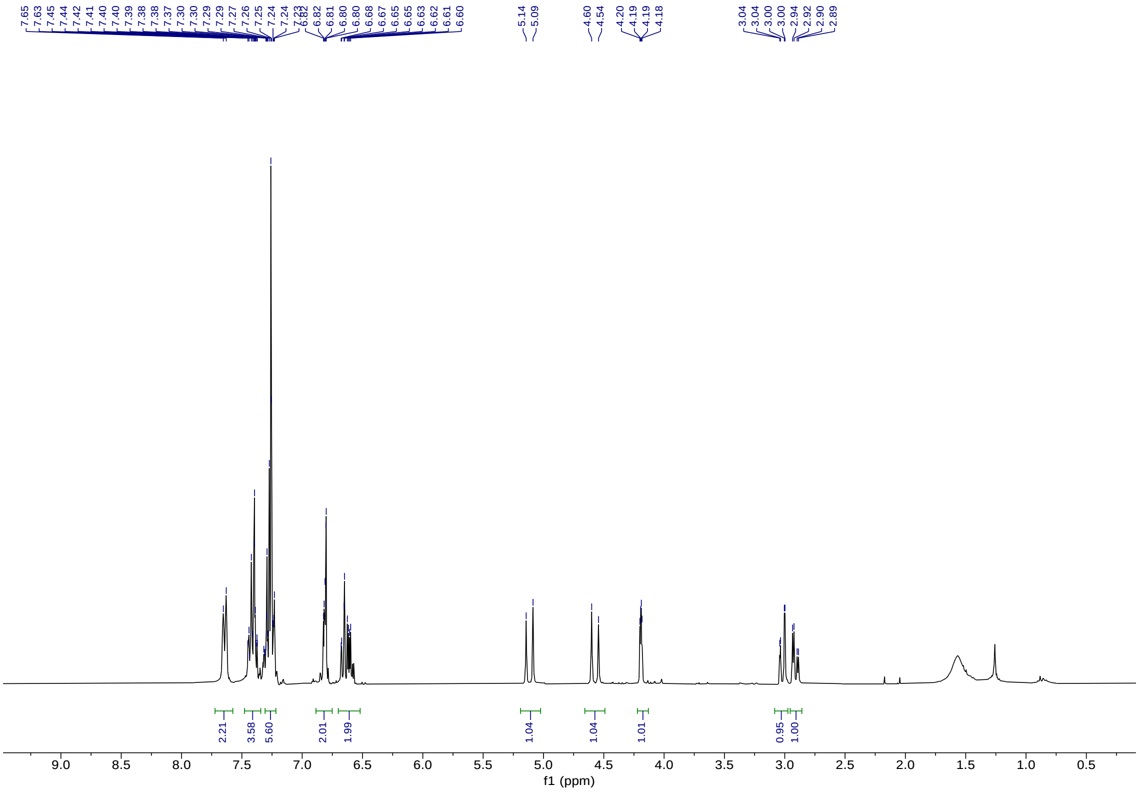

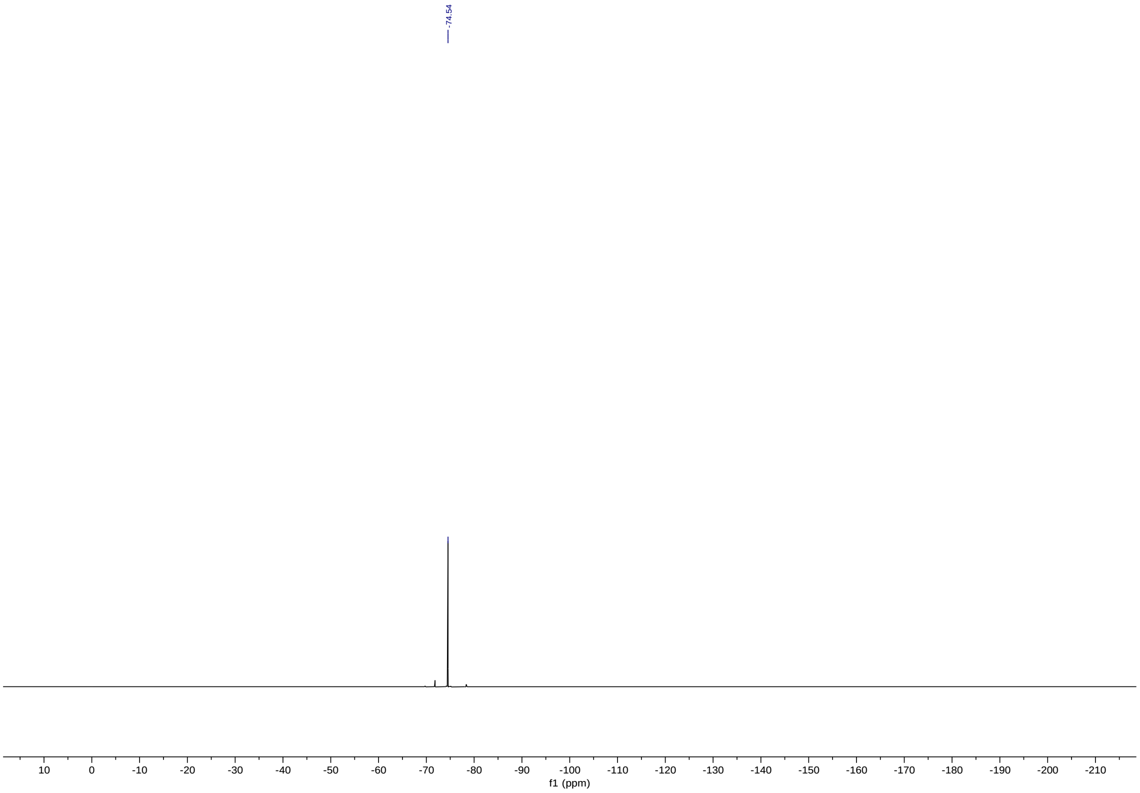

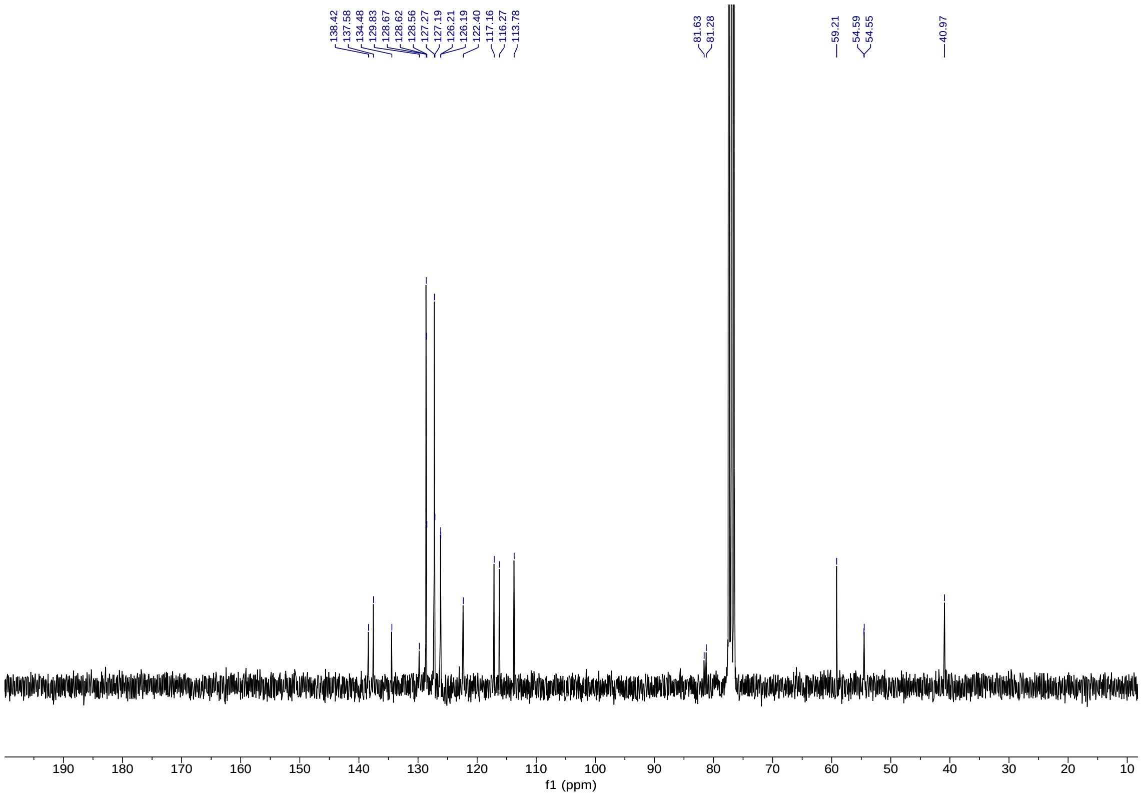
138.42
137.58
134.48
129.83
128.67
128.62
128.56
127.27
127.19
126.21
126.19
122.40
117.16
116.27
113.78
81.63
81.28
59.21
54.59
54.55
40.97
190
180
170
160
150
140
130
120
110
100
90
80
70
60
50
40
30
20
10
f1 (ppm)

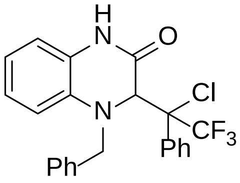

7'

^{1}H-NMR ($CDCl_3$, 300 MHz)
^{19}F{^{1}H}-NMR ($CDCl_3$, 282 MHz)
^{13}C{^{1}H}-NMR ($CDCl_3$, 75 MHz)

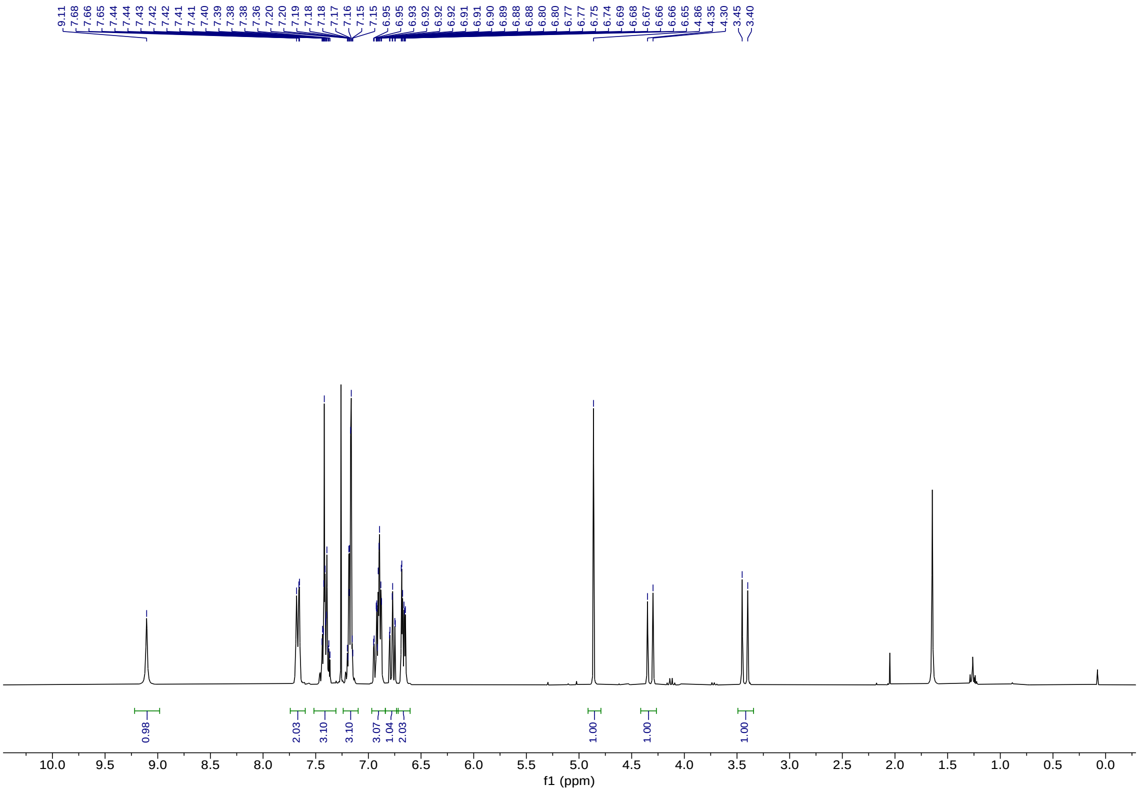

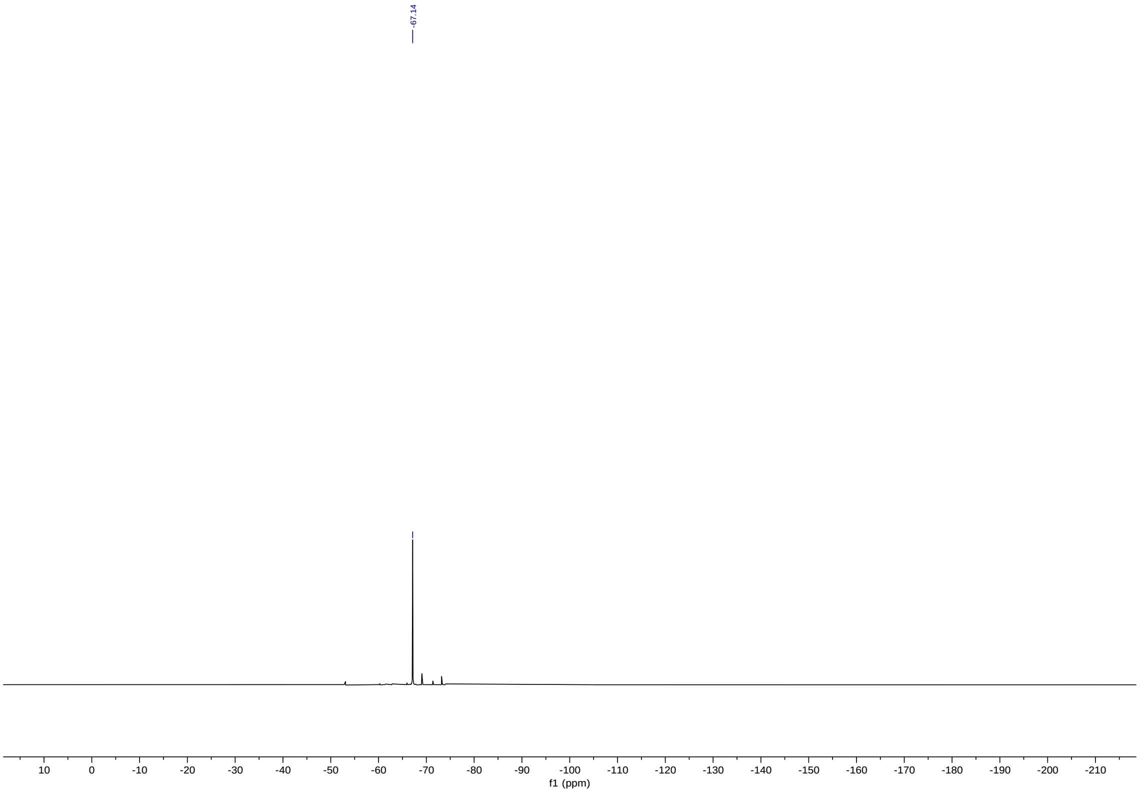

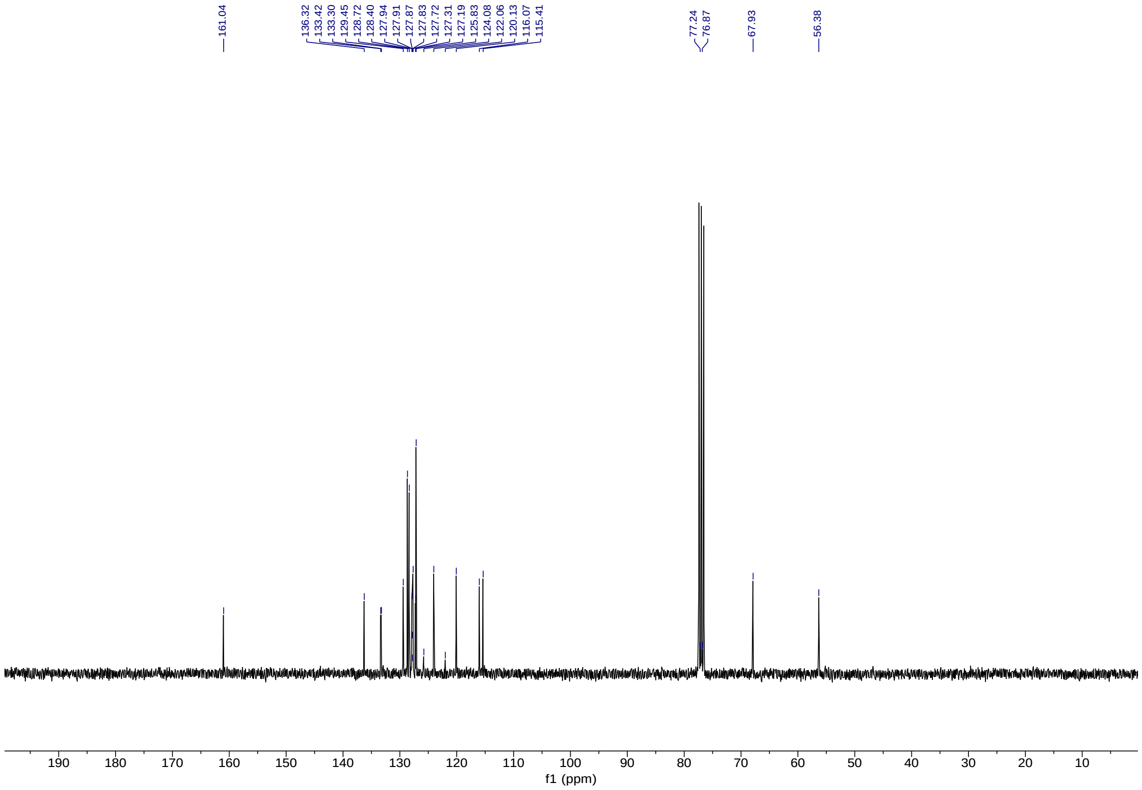
161.04
136.32
133.42
133.30
129.45
128.72
128.40
127.94
127.91
127.87
127.83
127.72
127.31
127.19
125.83
124.08
122.06
120.13
116.07
115.41
77.24
76.87
67.93
56.38
190 180 170 160 150 140 130 120 110 100 90 80 70 60 50 40 30 20 10
f1 (ppm)

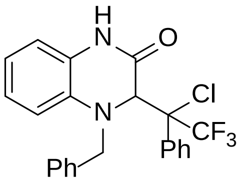

7''

^{1}H-NMR ($CDCl_3$, 300 MHz)
^{19}F{^{1}H}-NMR ($CDCl_3$, 282 MHz)
^{13}C{^{1}H}-NMR ($CDCl_3$, 75 MHz)

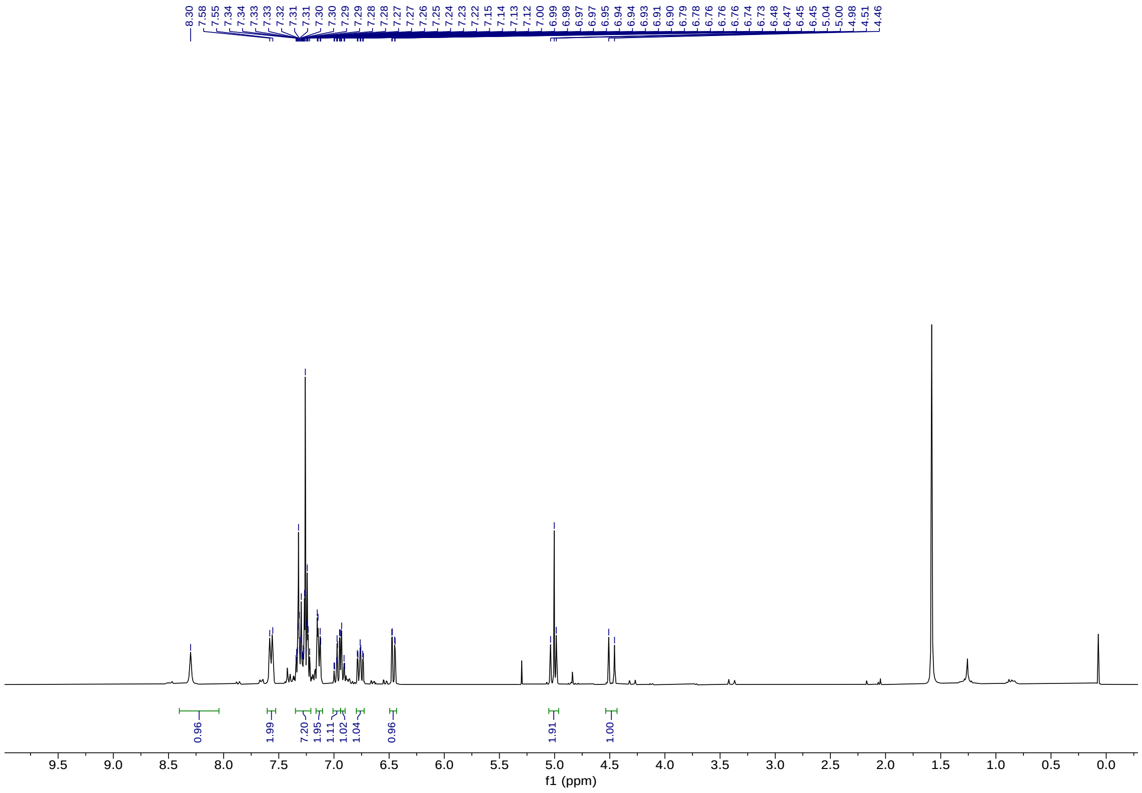

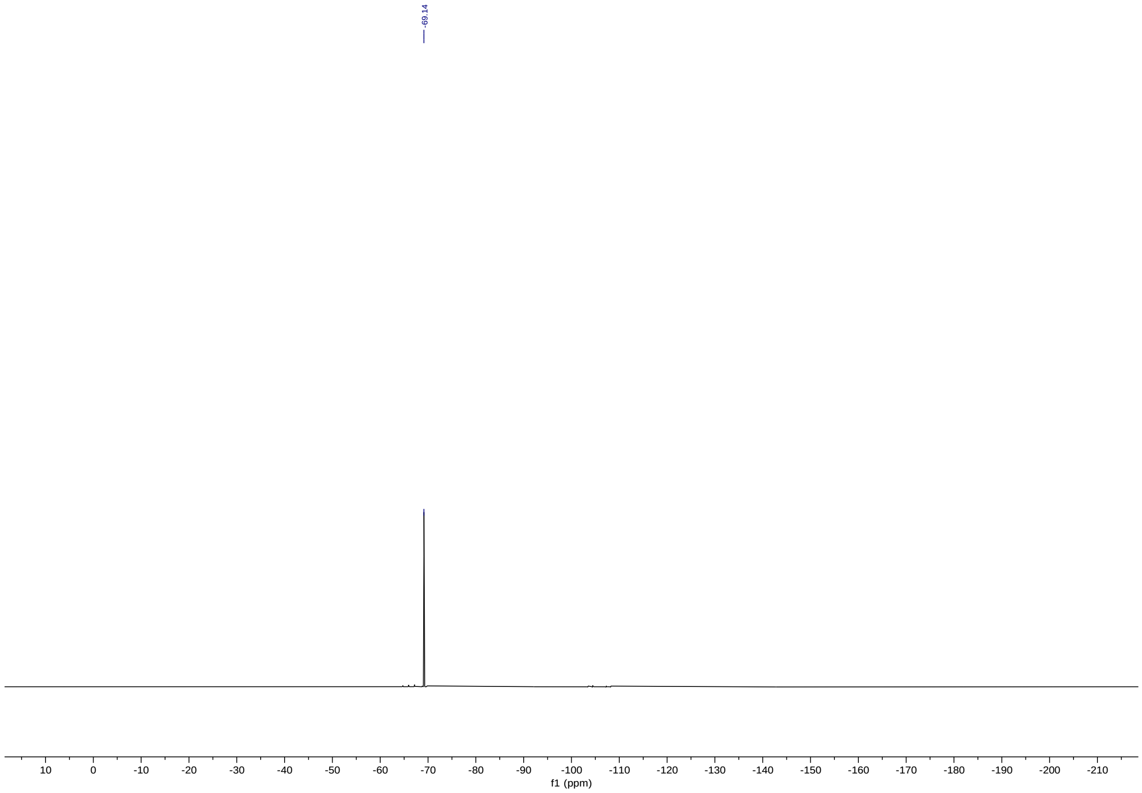

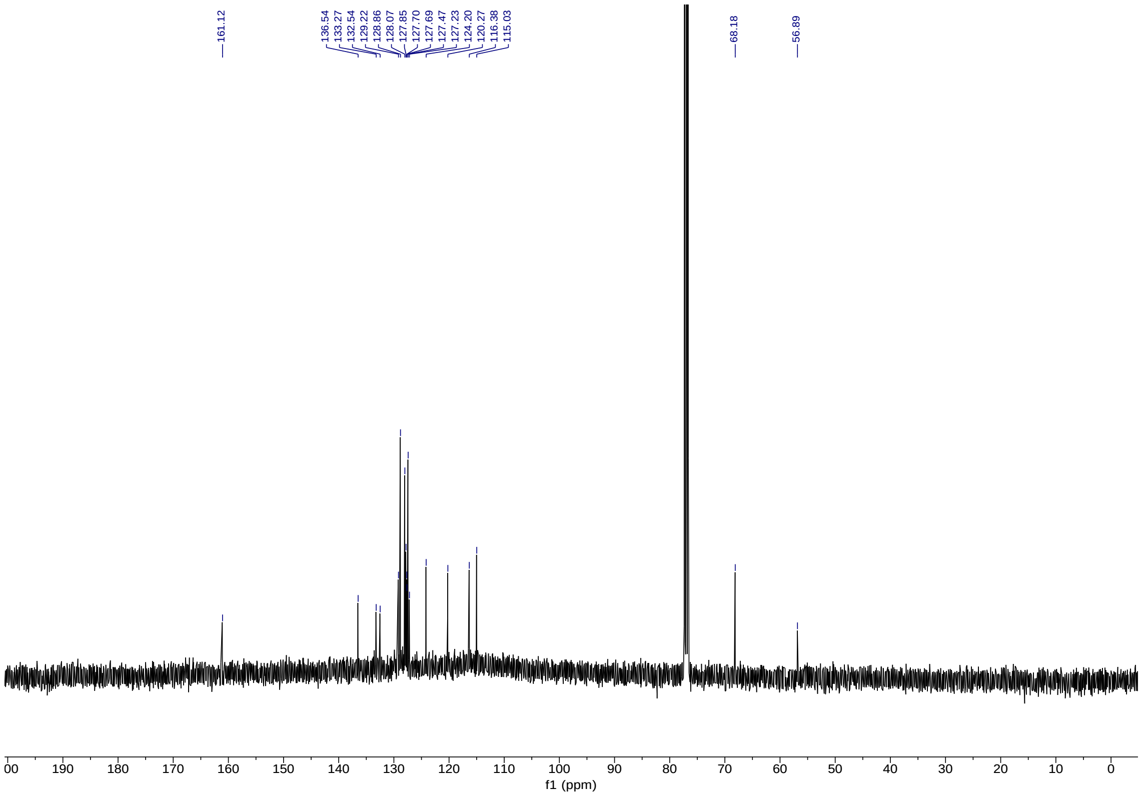
161.12
136.54
133.27
132.54
129.22
128.86
128.07
127.85
127.70
127.69
127.47
127.23
124.20
120.27
116.38
115.03
68.18
56.89
00
190
180
170
160
150
140
130
120
110
100
90
80
70
60
50
40
30
20
10
0
f1 (ppm)

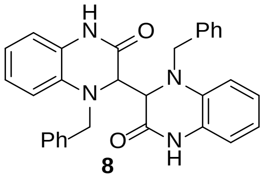

8

^{1}H NMR (300 MHz, DMSO-d^{6})

^{13}C {^{1}H} NMR (75 MHz, DMSO-d^{6})

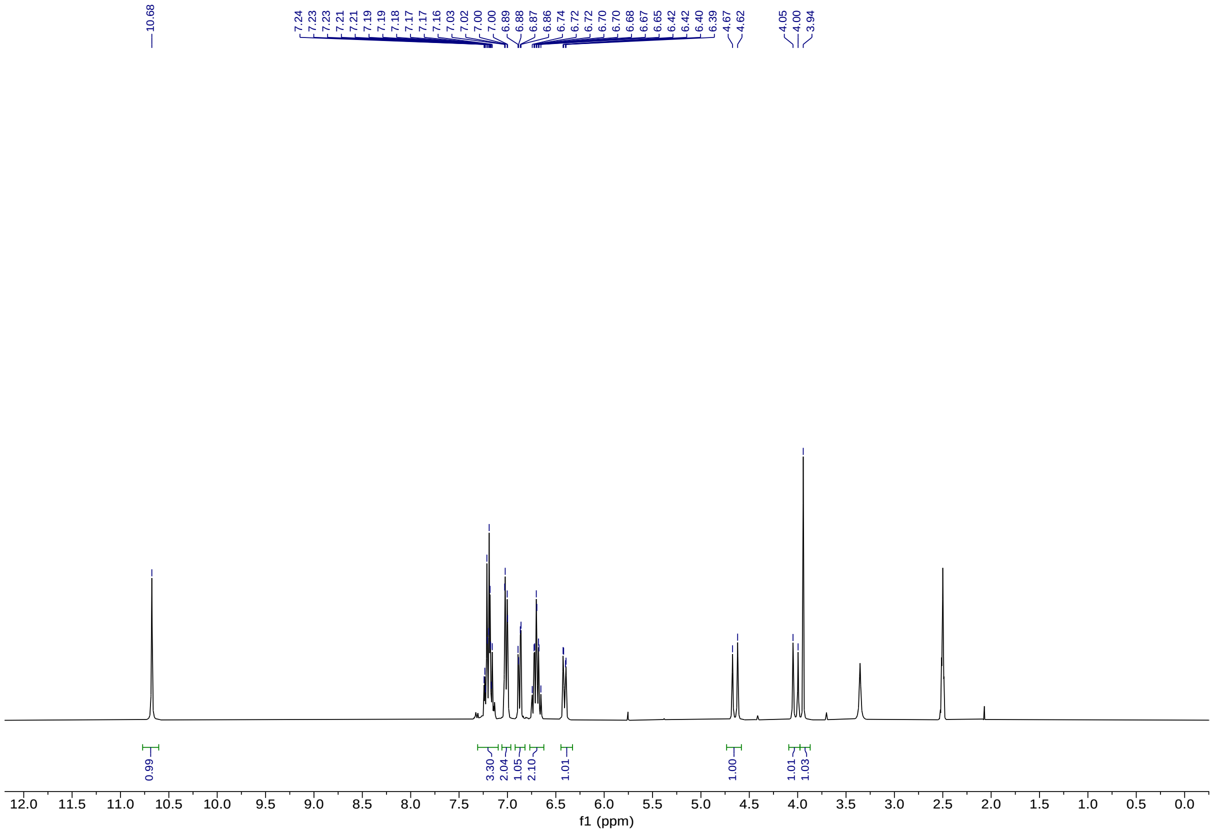

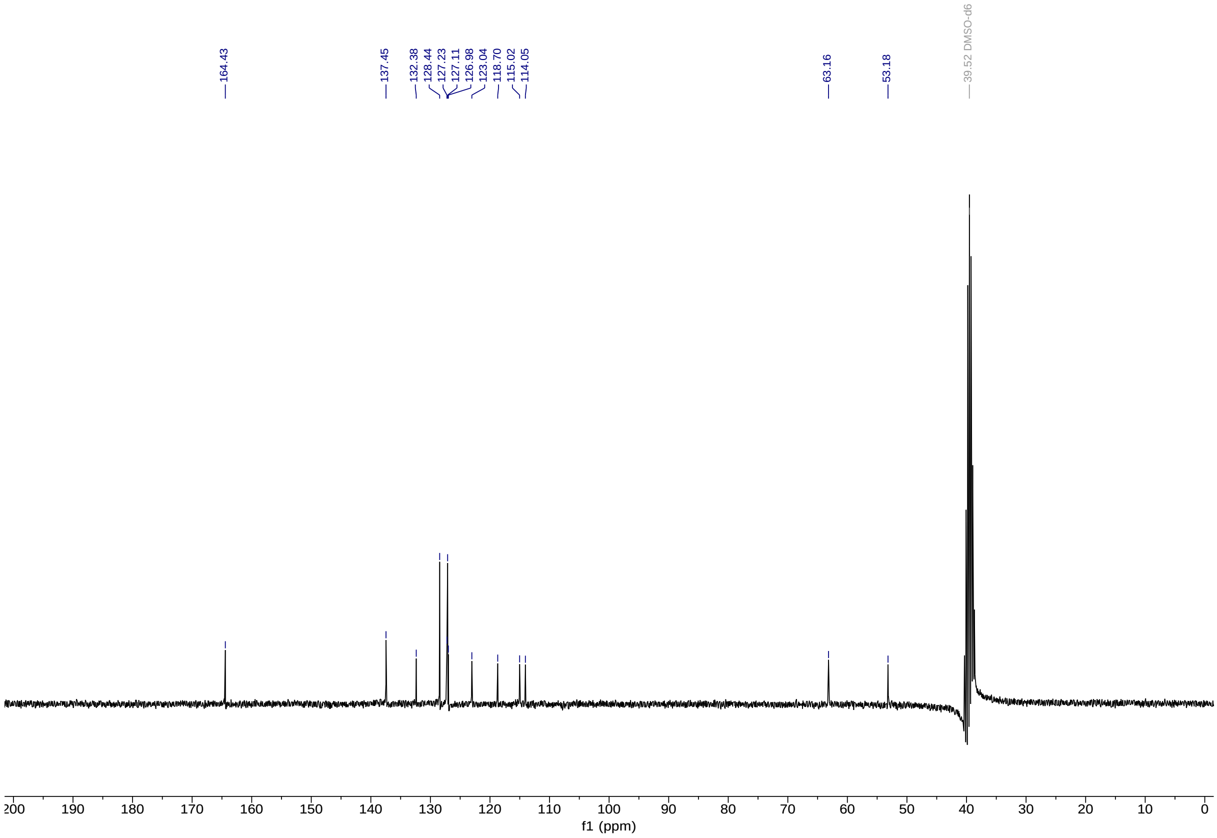

Supplement: Supplementary file 1 — jo2c01139_si_001.pdf [file jo2c01139_si_001.pdf]
